# Construction and Investigation of a lncRNA-Associated ceRNA Regulatory Network in Cholangiocarcinoma

**DOI:** 10.3389/fonc.2019.00649

**Published:** 2019-08-09

**Authors:** Junyu Long, Jianping Xiong, Yi Bai, Jinzhu Mao, Jianzhen Lin, Weiyu Xu, Hui Zhang, Shuguang Chen, Haitao Zhao

**Affiliations:** ^1^Department of Liver Surgery, Peking Union Medical College Hospital, Chinese Academy of Medical Sciences & Peking Union Medical College, Beijing, China; ^2^Interventional Radiology, Beijing Friendship Hospital, Capital Medical University, Beijing, China; ^3^Department of General Surgery, Peking Union Medical College Hospital, Chinese Academy of Medical Sciences & Peking Union Medical College, Beijing, China

**Keywords:** competing endogenous RNA network, long non-coding RNA, cholangiocarcinoma, prognosis, microRNA

## Abstract

**Background/Aims:** As a type of malignant tumor commonly found in the bile duct, cholangiocarcinoma (CCA) has a poor prognosis. Long non-coding RNA (lncRNA) has recently drawn increasing attention because it functions as a competing endogenous RNA (ceRNA) to hinder miRNA functions that participate in posttranscriptional regulatory networks in tumors. Therefore, to investigate the mechanisms of CCA carcinogenesis and to enhance treatment efficiency, the expression profiles, including lncRNA, miRNA, and mRNA data, were comprehensively integrated and analyzed in this study.

**Methods:** A comprehensive comparison was performed on the RNA-sequencing and miRNA profiles data of 36 CCA samples and 9 normal samples from The Cancer Genome Atlas (TCGA) database. Then, a dysregulated lncRNA-related ceRNA network was established by using four public databases.

**Results:** In summary, 1,410 lncRNAs, 64 miRNAs, and 3,494 mRNAs appeared as genes that were aberrantly expressed in CCA. Then, a dysregulated ceRNA network related to the lncRNAs was constructed. The network included 116 lncRNAs, 13 miRNAs and 60 mRNAs specific to CCA. The survival analysis showed that, among them, 26 lncRNAs, 3 miRNAs, and 13 mRNAs were prognostic biomarkers for patients with CCA. Finally, three mRNAs were selected for validation of their expression levels in the Gene Expression Omnibus (GEO) database. The results indicated that the expression of those genes was highly consistent between the TCGA and GEO databases.

**Conclusions:** The findings in this study provide a better understanding of the ceRNA network involved in CCA biology and lay a solid foundation for improving CCA diagnosis and prognosis.

## Introduction

Cholangiocarcinoma (CCA) is a malignancy most commonly observed in the bile duct and the second most common primary hepatocellular carcinoma (HCC) (1). CAA can be divided into three subtypes, namely, intrahepatic distal (dCCA), perihilar (pCCA), and intrahepatic (iCCA) cholangiocarcinoma. Although such malignancy usually occurs in Asia, an obvious increase in incidence has recently been observed in North America and Europe (2). In addition, CCA has a dismal prognosis (3). Curative treatment for patients with CCA may depend on surgery; however, most patients are diagnosed at an advanced stage of disease, and recurrence can occur frequently after resection (4). Patients with an advanced tumor stage have a dismal 5-year survival rate of below 5% (4). Given the lack of effective treatment options at an advanced stage of disease, the early detection of CCA is essential for providing patients a curative therapeutic approach and to maximize clinical outcomes (4). Therefore, prognostic biomarkers might help guide treatment decisions with respect to patient life expectancy and develop personalized treatments for individual CCA patients.

Long non-coding RNAs (lncRNAs), which are longer than 200 nucleotides, act as non-protein-coding transcripts (5). Because they do not encode proteins, they were originally thought to be “junk DNA.” However, increasing attention has been recently paid to the application of lncRNAs in the biology of cancers because lncRNAs can participate in carcinogenesis and cancer metastasis, as revealed by several studies (6). Nevertheless, it is unclear whether lncRNAs regulate gene expression. The regulatory function of lncRNAs on gene expression at the transcription, post-transcription and translation levels has been reported in the form of both theoretical and experimental results, but the complete influencing mechanism of lncRNAs on cancer has not been elucidated (6).

Salmena et al. put forth a hypothesis about competing endogenous RNA (ceRNA) in 2011, revealing that lncRNA, mRNA, and other types of RNAs can hinder miRNA function by virtue of shared microRNA response elements (MREs) as natural miRNA sponges (7). Many studies have proven this hypothesis via experiments, showing that lncRNA can compete for miRNA as a type of ceRNA to communicate with mRNA (8). Due to similar sequences with targeted miRNA, lncRNA is able to separate miRNA from mRNA. For example, under the upregulation of oxidative stress, H19 and HULC can target IL-6 and CXCR4 by virtue of ceRNA patterns of sponging let-7a/let-7b and miR-372/miR-373, respectively, to moderate the invasion and migration of CCA cells (9). The lncRNA TUG1 can competitively bind itself to miR-335-5p to regulate ROCK1, followed by inducing the migration and invasion of osteosarcoma cells (10). It is believed that exploring RNA interactions is of vital significance for the treatment of cancer. Specific lncRNA-miRNA-mRNA ceRNA networks have been constructed for esophageal squamous cell carcinoma, cutaneous melanoma and human lung squamous cell carcinoma on the basis of the abovementioned theory (11–13). Nevertheless, there remain insufficient comprehensive analyses on the lncRNAs and miRNAs related to CCA at the genome-wide scale, particularly on the basis of high-through detection.

The Cancer Genome Atlas (TCGA) database, which is available to the public, provides abundant resources for biological discovery and data mining because it collects data from approximately 10,000 patients and clinicopathological information related to over 30 types of human cancers (14, 15). Recently, the study with TCGA in CCA was published and this integrated analysis of DNA methylation, copy number, RNA expression, and somatic mutations led to a molecular classification and identified an IDH mutant-enriched subtype with distinct molecular characteristics (14). In the present study, we extracted expression data from TCGA including data from 36 tumor samples and 9 adjacent non-tumor samples (14). Here, we used differentially expressed genes (DEGs) between CCA and the adjacent liver to construct a ceRNA network. In total, 1,410 lncRNAs, 64 miRNAs, and 3,494 mRNAs were identified to be aberrantly expressed in CCA and were used to construct the aberrant ceRNA network related to the lncRNAs. The ceRNA network consisted of 116 lncRNAs, 13 miRNAs, and 60 mRNAs. Survival analyses showed that, among them, 26 lncRNAs, 3 miRNAs, and 13 mRNAs were prognostic biomarkers for patients with CCA. In conclusion, novel lncRNAs, miRNAs, and mRNAs were identified as potential prognostic biomarkers and therapeutic targets specific to CCA. More importantly, the proposed ceRNA network may allow a better understanding of the regulatory mechanism of ceRNA mediated by lncRNA in the pathogenesis of CCA.

## Materials and Methods

### Data Collection and Pre-processing

The RNA expression profiles (level 3) and the clinical information of patients with CCA were downloaded from TCGA. Specifically, the study included all samples with expression data (*n* = 45) of patients with CCA in the TCGA database, including 36 CCA tumor samples and 9 non-tumor samples. LncRNAs and mRNAs were quantified on the basis of the Genome Research Project of ENCyclopedia of DNA Elements (GENCODE) (GRCh38) catalog (http://www.gencodegenes.org/). The transformed data (sense_overlapping, lincRNA, 3prime_overlapping_ncrna, processed_transcript, antisense and sense_intronic) were considered lncRNAs. In summary, 19,660 mRNAs and 1,4254 lncRNAs derived from RNA sequencing (RNA-Seq) as well as 1,881 miRNAs derived from miRNA-Seq were retrieved. The sequencing data were derived using the Illumina HiSeq RNA-Seq and Illumina HiSeq miRNA-Seq platforms. This study was carried out strictly following TCGA publication guidelines. As the data came from TCGA, there was no need for ethics committee approval. Another CCA patient cohort that included 163 samples (104 CCA samples and 59 surrounding liver samples) was downloaded from the GEO database under accession number GSE26566 and was applied to examine the expression of mRNA biomarkers as the validation cohort (16). In addition, the gene expression profiles from GSE89749 (including 118 CCA samples) and GSE76297 (including 91 CCA samples) also were obtained from the GEO database to validate the relationships between lncRNAs and mRNAs.

### Identification of Significantly DEGs

Differential expression analyses were performed for the identification of differentially expressed lncRNAs, mRNAs and miRNAs (hereafter referred to as “DElncRNAs,” “DEmRNAs” and “DEmiRNAs,” respectively) between the non-cancerous samples and the CCA samples using the “edgeR” packages in R (17). DElncRNAs, DEmiRNAs, and DEmRNAs were selected based on the same cut-off value corresponding to the false discovery rate (FDR) adjusted by the Benjamini-Hochberg method. FDR <0.05 and |log fold change (FC)| >1 were taken as the cut-off criteria. Heatmaps were generated by hierarchical clustering analysis and utilized to identify the gene expression differences between the normal samples and the CCA samples.

### Construction of the ceRNA Network

The miRcode database was used to predict which DElncRNAs and DEmiRNAs could interact with each other (18). DEmRNAs targeted by DEmiRNAs were retrieved based on the TargetScan, miRDB, and miRTarBase databases, and only those recognized by these three mentioned databases served as candidate DEmRNAs (19–21). The next step was to establish the lncRNA-miRNA-mRNA ceRNA network on the foundation of the interactions between DElncRNAs and DEmiRNAs and between DEmiRNAs and DEmRNAs. The established network was visualized by “ggalluvial” R package.

### Functional and Pathway Enrichment Analyses

To clarify the potential biological process of DEmRNAs associated with the ceRNA network, we used the Database for Annotation, Visualization, and Integrated Discovery (DAVID) online tool (https://david.ncifcrf.gov/) to analyze the Gene Ontology (GO) functional enrichment of DEmRNAs (22). Default parameters were set for the analysis. The whole human genome was set as the background, and functional categories with *P* < 0.05 were considered significant. To understand the potential pathways of the DEmRNAs participating in the ceRNA network, Kyoto Encyclopedia of Genes and Genomes (KEGG) pathway enrichment analysis of the DEmRNAs was performed using the KO-Based Annotation System (KOBAS) online tool (http://kobas.cbi.pku.edu.cn/index.php) (23). KEGG pathways were also evaluated for significance (*P* < 0.05).

### Construction of Protein-Protein Interaction (PPI) Network

We adopted the Search Tool for the Retrieval of Interacting Genes (STRING, https://string-db.org/) database to build the PPT network of DEmRNAs, aiming to clarify the interaction among DEmRNAs (24). Additionally, Cytoscape software was used to embody the constructed PPI networks.

### Survival Analysis

As the ceRNA network proposed a large number of potential DElncRNAs, DEmiRNAs, and DEmRNAs, the association among these genes and the prognosis of patients with CCA were estimated to unveil to what extent and in which aspects these genes play a role in the overall survival of patients with CCA. We used the survival package in R to perform the Kaplan–Meier survival analyses of the association between the expression of DElncRNAs, DEmiRNAs, and DEmRNAs in the ceRNA network and the prognosis of patients with CCA.

### Clinical Feature Analysis of ceRNAs Network

We extracted clinical information from TCGA database. Clinical features such as vascular invasion, histologic grade and neoplasm histologic grade were chosen to analyze the correlation between these features and ceRNAs.

### Validation of DEmRNAs

The GSE26566 dataset was adopted for validating the expression of candidate genes (25). The transcriptome profiles in the GSE26566 (Platform: GPL6104) dataset including 104 CCA samples and 59 non-tumor liver samples (16).

### Functional Prediction of the DEmRNAs

To infer the potential functions of the DEmRNAs in the ceRNA network, gene set enrichment analysis (GSEA) of the DEmRNAs was performed. Thirty-five CCA samples in the TCGA database were classified into two groups (high vs. low) according to the median expression level of the DEmRNAs in the RNA sequencing data. The reference gene sets were the annotated gene sets of c2.cp.kegg.v6.1.symbols.gmt in the Molecular Signatures Database (MSigDB). The cut-off criteria was *P* < 0.05.

### RNA Extraction and qPCR Validation

Eleven paired CCA and corresponding normal liver tissue samples were attained from patients with CCA in the Peking Union Medical College Hospital (PUMCH). All the diagnoses were according to pathological assessments. Upon removal of the surgical specimen, each sample was immediately frozen in liquid nitrogen and stored at −80°C prior to RNA isolation and qPCR analysis.

We extracted RNA from tissue samples using TRIzol reagent (Invitrogen, Carlsbad, CA, USA). Complementary DNA (cDNA) was synthesized using the HiScript III RT SuperMix for qPCR (+g DNA wiper) (Vazyme Biotech, NanJing, China). qPCR was performed with a AceQ qPCR SYBR Green Master Mix (Vazyme Biotech, NanJing, China) via a StepOnePlus Real-Time PCR system (Applied Biosystems, Foster City, CA, USA). The sequence of PCR primers of SLC43A1 were GGACGTGGAAGCTCTGTCTC (Forward primer) and GCAGCGTGAGTGAAGTGAAC (Reverse primer). The PCR cycling conditions were as follows: 95°C for 5 min, 40 cycles of 95°C for 10 sec, 60°C for 34 s, dissociation at 95°C for 15 s, 60°C for 1 min and 95°C for 15 s. The results were analyzed using the 2^−ΔΔ*CT*^ method. The qPCR reactions were all repeated three times. The paired *t*-test was used for normally distributed data; otherwise, the Wilcoxon rank test for paired data was used to evaluate whether a gene was differentially expressed between tumor samples and normal samples.

## Results

### Identification of Differentially Expressed lncRNA, miRNA and mRNA

**Figure 1:**
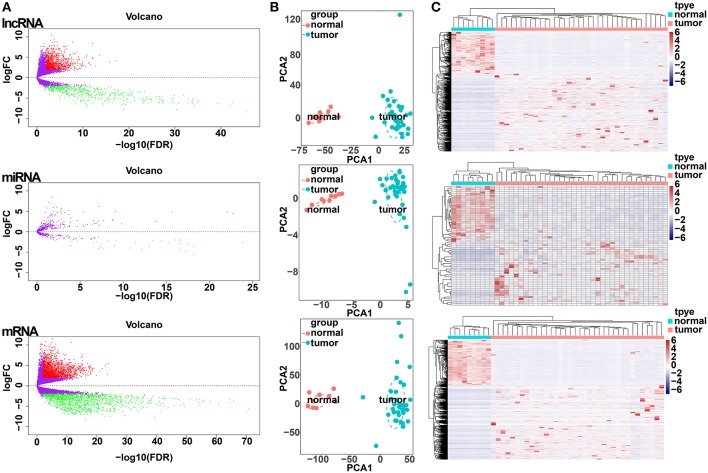
Differentially expressed gene (DEG) analysis. **(A)** Volcano plots of differentially expressed lncRNAs (upper panel), miRNAs (middle panel), and mRNAs (lower panel). The Y-axis represents the negative log (base 10) of the FDR, and the X-axis represents the log (base 2) of FC. Each point represents a gene. The dots in green denote downregulated DEGs, the dots in red denote upregulated DEGs, and the dots in black denote non-DEGs. **(B)** Principal components analysis (PCA) plot of the differentially expressed lncRNAs (upper panel), miRNAs (middle panel), and mRNAs (lower panel). The tumor samples (green, *n* = 36) and normal samples (red, *n* = 9) are plotted along the axis about the first two principal components (PC1 and PC2). **(C)** Heatmap plots of the differentially expressed lncRNAs (upper panel), miRNAs (middle panel), and mRNAs (lower panel). The horizontal axis represents the samples, and the upper horizontal axis represents the sample clusters. The vertical axis represents the DEGs, and the left vertical axis shows the DEG clusters. Red represents upregulated genes, while green represents downregulated genes.

All samples with expression data (*n* = 45) of patients with CCA in the TCGA database, including 36 CCA tumor tissues and 9 adjacent non-cancerous tissues were enrolled in our study (Table S1). Differential expression analysis was performed based on comparisons of the expression of 14,254 lncRNAs, 19,660 miRNAs and 1,881 mRNAs between 36 CCA and 9 adjacent normal liver tissue samples in the TCGA database. Using thresholds of FDR <0.05 and |log FC| >1, 1,410 DElncRNAs (916 upregulated and 494 downregulated), 64 DEmiRNAs (34 upregulated and 30 downregulated), and 3,494 DEmRNAs (2,184 upregulated and 1,310 downregulated) between CCA and adjacent normal liver tissues were identified (Figure 1A; Table S2). Clustering analysis and principal component analysis of DElncRNAs, DEmiRNAs, and DEmRNAs suggested that CCA samples may be distinguished from normal samples based on the expression levels of DElncRNAs, DEmiRNAs, and DEmRNAs (Figures 1B,C).

### Prediction of DelncRNA-DEmiRNA Interactions by miRcode

We used the miRcode database to predict the miRNA-targeted lncRNAs. The constructed ceRNA network only included the interactions between DEmiRNAs and DElncRNAs; thus, 328 interactions between 117 DElncRNAs (AL591845.1, KIAA0087, and H19, etc.) and 14 DEmiRNAs (hsa-mir-96, hsa-mir-182, and hsa-mir-122, etc.) were identified (Table S3).

### Prediction of DEmiRNA-Targeted DEmRNAs

**Figure 2:**
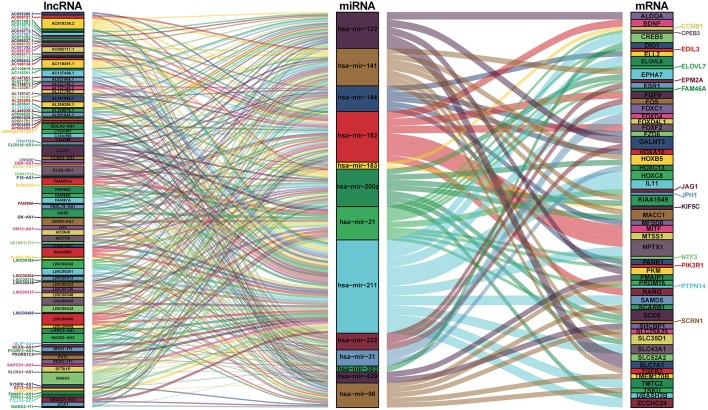
Sankey diagram for the ceRNA network in cholangiocarcinoma. Each rectangle represents a gene, and the connection degree of each gene is displayed based on the size of the rectangle.

We mapped the abovementioned 14 DEmiRNAs into the TargetScan, miRDB, and miRTarBase databases and searched for the target DEmRNAs. Sixty DEmRNAs that interacted with 13 of the 14 DEmiRNAs in all 3 datasets were selected (Table S4). After removing the remaining 1 DEmiRNA and the corresponding lncRNAs, finally 116 DElncRNAs, 13 DEmiRNAs, and 60 DEmRNAs were obtained to build the ceRNA network (Figure 2; Table S4). In addition, we calculated the connection degree of each gene by topology to clarify its importance in the ceRNA network (Figure 2; Table S6). KIAA0087 (connection degree = 8), hsa-mir-211 (connection degree = 50), and KIAA1549 (connection degree = 2) were considered the most important genes among the lncRNAs, miRNAs, and mRNAs, respectively (Figure 2; Table S6). Because hsa-mir-211 was the gene with the highest connection degree in the ceRNA network (connection degree = 50), we concluded that it might exert a strong influence on CCA pathogenesis. The ceRNA network also shows that DElncRNAs interact with DEmRNAs in an indirect manner.

### Functional Enrichment Analysis and Protein-Protein Interaction Network Construction

**Figure 3:**
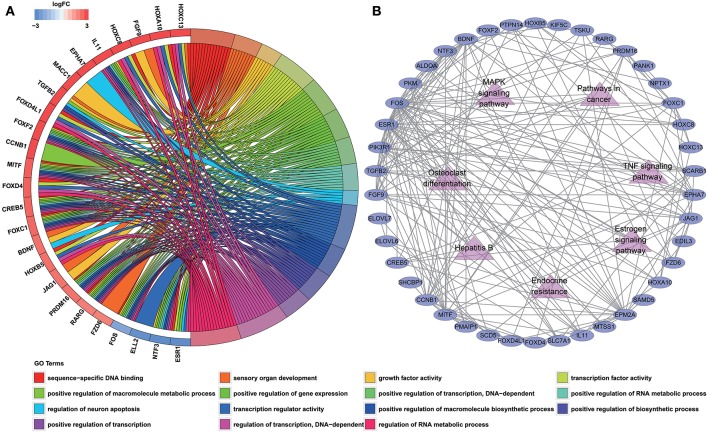
Functional enrichment analysis of 60 DEmRNAs**. (A)** Chord diagram showing enriched GO clusters for the 60 DEmRNAs. In each chord diagram, genes contributing to their respective enrichment are shown on the left, and enriched GO clusters are shown on the right. Downregulated DEmRNAs are displayed in blue, whereas upregulated DEmRNAs are displayed in red. Each GO term is represented by one colored line. **(B)** PPI networks of the DEmRNAs. Ellipse represents DEmRNA, and edge represents the interaction between two proteins. Triangles represent pathways in which 60 DEmRNAs were enriched.

To determine the biological functions and pathways of the 60 DEmRNAs in the constructed ceRNA network, we used the DAVID database to analyze the GO functional enrichment and the KOBAS database to analyze KEGG pathway enrichment. This analysis revealed the enrichment of 15 GO terms and 7 KEGG pathways (with *P* < 0.001 and 0.0001, respectively; Tables S7, S8). The top GO terms were “sequence-specific DNA binding,” “sensory organ development” and “growth factor activity” (Figure 3A; Table S7). According to KEGG pathway analyses, the most significant pathways identified were “endocrine resistance,” “estrogen signaling pathway” and “TNF signaling pathway” (Figure 3B; Table S8). In addition, we constructed PPI networks to explore the relationship among DEGs via the STRING database, which was visualized by Cytoscape software (Figure 3B).

### Association Between Key Genes and Overall Survival

**Figure 4:**
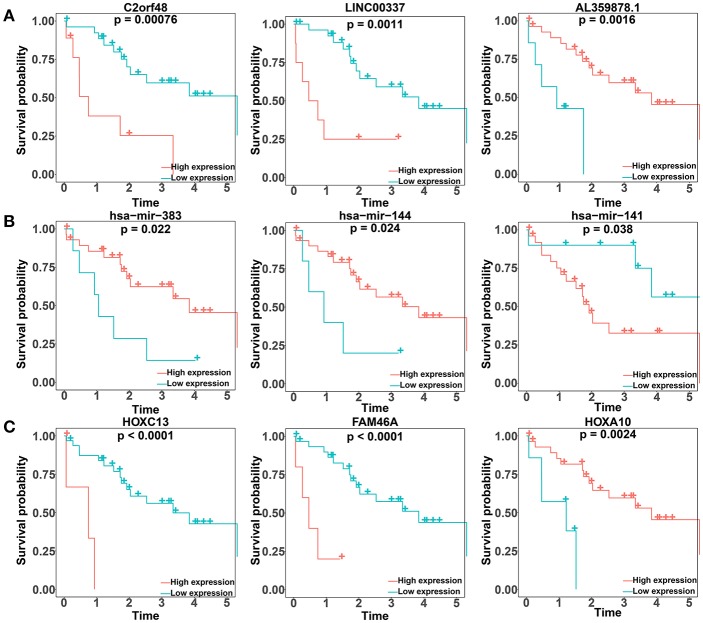
Kaplan–Meier survival curves for the top three lncRNAs, miRNAs, and mRNAs related to overall survival based on their optimal cutoffs in cholangiocarcinoma. The horizontal axis represents the overall survival time in years; the vertical axis represents the survival probability.

We performed a log-rank test and Kaplan-Meier survival analysis for each DElncRNA, DEmiRNA, and DEmRNA in the constructed ceRNA network to obtain RNAs that exhibited a close relation to the prognosis of patients with CCA. Twenty-six DElncRNAs, 3 DEmiRNAs, and 13 DEmRNAs were identified as RNAs associated with overall survival based on their respective optimal cutoffs (Figure 4; Tables S9–S11). Among these, 17 DElncRNAs, 2 DEmiRNAs, and 7 DEmRNAs seemed to exhibit protective function, as the prognosis of patients with higher expression levels of these RNAs was longer than that in patients with lower expression levels. In contrast, the remaining 9 DElncRNAs, 1 DEmiRNA, and 6 DEmRNAs were regarded as risky, as there was a negative relationship between their expression and the prognosis of CCA patients.

### Positive Correlation Among the Expression Levels of ceRNAs

**Figure 5:**
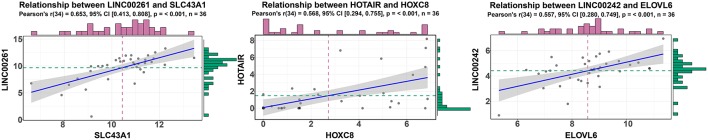
Regression analysis between the expression levels of DElncRNAs and DEmRNAs targeted by hsa-miR-211 in the ceRNA network (showing the top three correlation coefficients). The horizontal axis denotes the expression of the DEmRNAs, and the vertical axis represents the expression of the DElncRNAs. The upper and right edges are histograms of gene expressions.

According to ceRNA theory, lncRNAs can positively regulate mRNAs by competitively compete with miRNAs for the binding sites of miRNAs. To validate this finding, a regression analysis was carried out between DElncRNAs and DEmRNAs targeted by hsa-mir-211, which was the gene with the highest degree of connections in the ceRNA network (connection degree = 50). Strong positive correlations were found between DElncRNAs and DEmRNAs targeted by hsa-mir-211 (minimum requirement of an interaction score >0.4; Table S12). Figure 5 showed the top three correlation coefficients of interactions, in which LINC00261 interacts with SLC43A1, HOTAIR interacts with HOXC8, and LINC00242 interacts with ELOVL6 under the regulation of hsa-mir-211.

### Validation of DEmRNAs in the ceRNA Network

**Figure 6:**
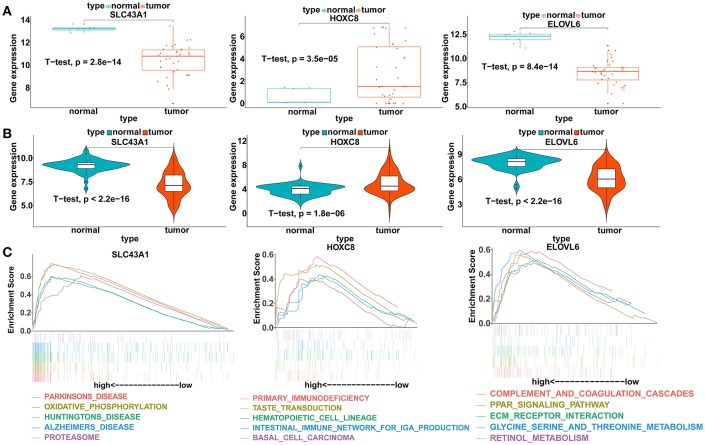
Validation of the expression levels and gene set enrichment analysis (GSEA) of SLC43A1, HOXC8, and ELOVL6. **(A)** Scatter plots of mRNA levels of SLC43A1, HOXC8, and ELOVL6 in the TCGA training dataset. **(B)** Violin plots of mRNA levels of SLC43A1, HOXC8, and ELOVL6 in the GSE26566 validation dataset. **(C)** The top five functional gene sets enriched in the CCA samples are listed, which showed high expression of SLC43A1, HOXC8, and ELOVL6.

To verify our analysis, the expression levels of SLC43A1, HOXC8, and ELOVL6 were assessed in 104 CCA tumor tissues and 59 adjacent non-tumor liver tissues. Consistent with our analysis findings, the mean expression levels of SLC43A1 and ELOVL6 were significantly lower while HOXC8 was significantly higher in CCA samples than in non-tumor liver samples in the GSE26566 dataset (Figures 6A,B). The results proved the reliability of our analysis.

### GSEA of the DEmRNAs

To identify the biological pathways related to SLC43A1, HOXC8, and ELOVL6, which were highly expressed according to the TCGA database, we performed GSEA in the CCA tissues based on the TCGA database (Figure 6C). CCA tissues in the SLC43A1 high expression group were most significantly enriched for “Parkinson's disease” (Table S13); CCA tissues in the HOXC8 high expression group were most significantly enriched for “primary immunodeficiency” (Table S14); and CCA tissues in the ELOVL6 high expression group were most significantly enriched for “complement and coagulation cascades” (Table S15).

### Validation of Positive Correlations Between lncRNAs and mRNAs

**Figure 7:**
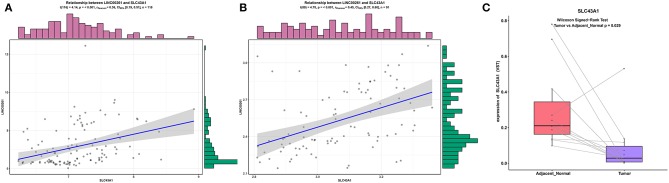
Validation of the relationship between LINC00261 and SLC43A1. Regression analysis between the expression levels of LINC00261 and SLC43A1 in the GSE89749 **(A)** and GSE76297 **(B)** validation datasets. Violin plots of mRNA levels of SLC43A1 in the PUMCH validation dataset by using RT-PCR **(C)**.

To make our results more reliable, we also verified the relationship between lncRNA and mRNA in other independent datasets (GSE89749 and GSE76297). Since among the correlation coefficients between DElncRNAs and DEmRNAs targeted by hsa-mir-211, the relationship between LINC00261 and SLC43A1 have highest correlation coefficient, we only verified the relationship between LINC00261 and SLC43A1. The results of the correlation analysis of these two datasets (GSE89749 and GSE76297) are consistent with the results of TCGA (Figures 5, 7A,B). In addition, to check the credibility of the bioinformatics results, we validated the expression of SLC43A1 by using qPCR. Paired *t*-tests were used to analyze the differences in expression of the SLC43A1 between 11 CCA samples and 11 adjacent non-cancerous samples. The qPCR results from 11 CCA patients were consistent with the bioinformatics results (Figures 6A,B, 7C).

### Clinical Feature Analysis of ceRNAs

**Figure 8:**
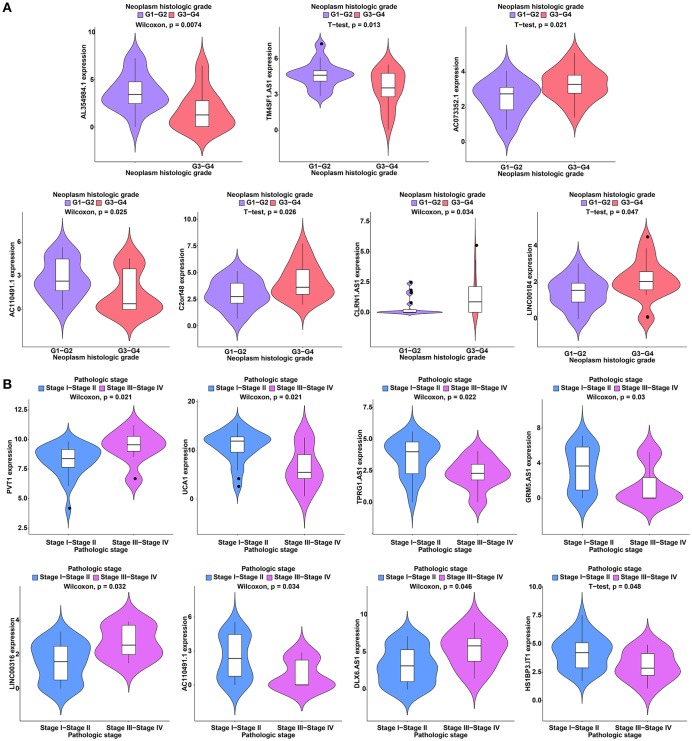
Clinical feature analysis of DElncRNAs in the ceRNA network. The correlation between DElncRNAs involved in the ceRNA network and neoplasm histologic grade **(A)** and pathologic stage **(B)**.

**Figure 9:**
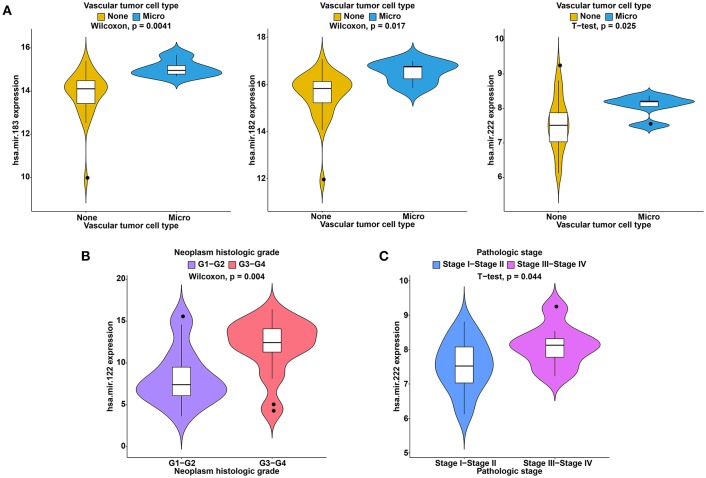
Clinical feature analysis of DEmiRNAs in the ceRNA network. The correlation between DEmiRNAs involved in the ceRNA network and vascular invasion **(A)**, neoplasm histologic grade **(B)** and pathologic stage **(C)**.

**Figure 10:**
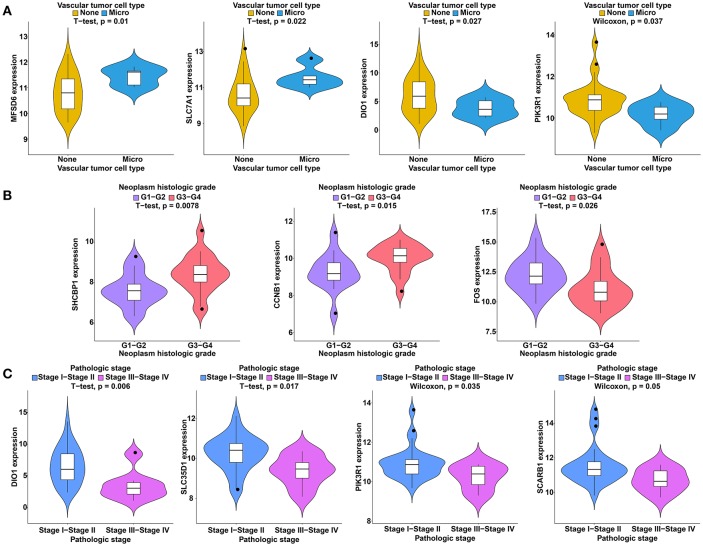
Clinical feature analysis of DEmRNAs in the ceRNA network. The correlation between DEmRNAs involved in the ceRNA network and vascular invasion **(A)**, neoplasm histologic grade **(B)** and pathologic stage **(C)**.

To further study the ceRNAs, the correlation between the ceRNAs involved in the ceRNA network and clinical features including vascular invasion, neoplasm histologic grade, and pathologic stage in TCGA database were analyzed. The results revealed that 3 miRNA, and 4 mRNA were associated with vascular invasion; 7 lncRNA, 1 miRNA, and 3 mRNA were associated with neoplasm histologic grade; 8 lncRNA, 1 miRNA, and 4 mRNA were associated with pathologic stage (*P* < 0.05; Figures 8–10).

## Discussion

CCA is one of the most common cancers worldwide, and its incidence and mortality remain high (26). Although great progress has been made in the treatment, prognosis and diagnosis of CCA in recent studies, CCA mortality remains high, which may be due to the lack of efficient biomarkers and the unclear mechanisms underlying CCA (1). Recent studies have demonstrated the vital role lncRNAs play in tumor progression, and some of these lncRNAs may be potential biomarkers for better prognosis and diagnosis (27). lncRNAs function in many ways. The ceRNA hypothesis postulates that lncRNAs may compete with miRNAs for the binding sites of miRNAs and may further affect mRNA expression through MREs (7). This hypothesis integrates the relationship among lncRNAs, mRNAs, and miRNAs and better explains the interaction among a variety of types of RNAs at the genetic level. We established a lncRNA-miRNA-mRNA ceRNA network mediated by lncRNA at the transcriptome-wide level in this study to lay a useful foundation for studying the regulatory function of ceRNA in CCA.

To our knowledge, few studies have focused on predicting CCA prognosis, and no systematic examination has been performed on the prognostic value of ceRNA in CCA. In addition, rare reliable lncRNAs, miRNAs and mRNAs in relation to CCA can be treated as molecular biomarkers for detecting CCA and stratifying CCA risk. Under the background, the lncRNAs, mRNA, and miRNA expression data of 36 CCA samples and 9 normal samples were attained from the TCGA database. Based on the comprehensive integration of the lncRNA, mRNA and miRNA expression data of 36 CCA tissues and 9 normal tissues, a ceRNA network related to lncRNA was established to study the regulatory mechanism of ceRNA. Although CCA is characterized by extremely complicated biogenesis, DEGs can represent the complicity of tumor development as well as metastasis dissemination to a large extent; therefore, we used DEGs to establish the ceRNA network. In this study, we found 1,410 DElncRNAs in the CCA samples compared with the normal liver samples and 117 DElncRNAs targeting DEmiRNAs in the ceRNA network. Twenty-seven of them presented an obvious relevance to overall survival. There were 64 DEmiRNAs in the CCA tissues compared with the non-cancerous tissues and 14 DEmiRNAs targeted by DElncRNAs in the ceRNA network. Thereinto, 3 DEmiRNAs exhibited remarkable relevance to overall survival. We also found 3,494 DEmRNAs in the CCA tissues compared with the non-cancerous tissues, and 60 DEmRNAs targeted by DEmiRNAs were included in the ceRNA network. Thereinto, 13 DEmiRNAs presented a remarkable relevance to overall survival. The genes associated with overall survival are likely to indicate prognosis for CCA.

We analyzed the functions and pathways of DEmRNAs participating in the ceRNA network by using GO and KEGG. These DEmRNAs were mainly enriched in functions of “sequence-specific DNA binding,” “sensory organ development” and “growth factor activity” and in the pathways of “endocrine resistance,” “estrogen signaling pathway,” and “TNF signaling pathway,” etc., which are closely associated with tumorigenesis.

In the ceRNA network, hsa-mir-211 could interact with the majority of DElncRNAs and DEmRNAs (connection degree = 50), suggesting that hsa-miR-211 has a significant effect on the pathogenesis and prognosis of CCA. Although dysregulation of miR-211 is observed in many cancers, the function of miR-211 in CCA remains unclear. Computational methods were used to predict miR-211-5p by using conservation with mouse and Fugu rubripes sequences (28). The sequence is mapped to human chromosome 15, and it has been shown that miR-211-5p greatly impacts many types of cancers, such as colorectal cancer (CRC), non-small cell lung cancer (NSCLC), gastric cancer (GC), HCC, etc. (29–32). Based on studies conducted in the past, miR-211-5p was downregulated in GC, HCC and ovarian cancer (29, 30, 33), yet it was overexpressed in NSCLC (32). MiR-211 presented a decreasing trend in HCC samples compared with surrounding non-cancerous samples (29). The overexpression of miR-211 subdued the invasion and proliferation in SMMC7721 and HepG2 cells (29). MiR-211 can downregulate SATB2, leading to subdued HCC (29). MiR-211 also showed an obvious downregulation in GC (30). The invasion and proliferation of GC cells *in vitro* could be subdued by the overexpression of miR-211 and could be facilitated by downregulating the expression of miR-211 (30). The proliferation and invasion of GC cells could be partially inhibited by miR-211 by virtue of downregulating SOX4 (30). In contrast, miR-211 expression was upregulated in cell lines and tissues of human NSCLC (32). As miR-211 was overexpressed, the proliferation, colony formation, and invasion of NSCLC cells were enhanced (32). In addition, by targeting SRCIN1, miR-211 played a role in facilitating the proliferation of NSCLC (32). It was found in our study that miR-211 was downregulated in CCA tissues in comparison with normal liver tissues. In addition, a correlation analysis between DElncRNAs and DEmRNAs targeted by miR-211 was performed and revealed that some DElncRNAs and some DEmRNAs targeted by miR-211 have very strong correlations; among them, LINC00261 has the strongest correlation with SLC43A1 (*P* = 1.58E−05).

LncRNA LINC00261 is mapped to 20p11.21 and has been shown to be downregulated in many cancer tissues compared with their surrounding non-cancerous samples (34, 35). There was an obvious downregulation of LINC00261 in GC tissues and a negative relationship between its expression and advanced tumor status, clinical stage and unfavorable prognosis of patients with GC (36). LINC00261 plays a role as a tumor suppressor in GC because it weakens the stability of Slug proteins and subdues the EMT (36). The expression of LINC00261 in HCC tissues appears to be lower than that in the surrounding normal tissues (37). The decrease in LINC00261 expression was correlated with greater tumor size, higher TNM stage (III-IV), and worse survival in patients with HCC (37). Based on the functional assays, as LINC00261 is overexpressed in HCC cells, the proliferation, colony formation and invasion of cells and the EMT process were subdued *in vitro*. Additionally, when LINC00261 was upregulated, the expression levels of Notch1 and Hes-1 in HCC cells were downregulated; thus, Notch signaling was greatly reduced (37). LINC00261 expression levels in NSCLC tissues were suppressed compared with those in surrounding non-cancerous lung tissues (38). Low expression of LINC00261 was found to significantly related to TNM stage, lymph node status, and distant metastasis (38). Based on Kaplan-Meier analysis, low LINC00261 expression levels were largely indicative of poorer overall survival of patients with NSCLC (38). In our findings, LINC00261 was strongly correlated with SLC43A1. Furthermore, LINC00261 and SLC43A1 were both downregulated in CCA. Based on our results, we believe that lncRNA LINC00261 can mediate SLC43A1 expression by competing with miR-211 to participate in the progression of CCA.

Our analysis has great importance in that it reveals a ceRNA network in CCA, and some of the results were confirmed by another database. However, there were still several limitations in our study. First, the number of tumor tissue samples for the survival analysis was not large, and therefore, a larger CCA cohort is needed to find more ceRNAs associated with prognosis. Second, further study is warranted on the functions of key ceRNAs *in vitro* and *in vivo*. Third, Xu et al. reported that lncRNA SPRY4-IT1 functioned as a molecular sponge for miR-101-3p, antagonizing its ability to repress EZH2 protein translation in CCA (39). However, lncRNA SPRY4-IT1 does not exist in the TCGA database, so lncRNA SPRY4-IT1/miR-101-3p does not exist in our ceRNA network. In the future, with the emergence of better sequencing methods, our ceRNA network will be improved. Additionally, Zhang reported that lncRNA LINC01296 promotes cancer growth and progression by sponging miR-5095 in cholangiocarcinoma. However, miR-5095 is not a differentially expressed gene in the TCGA database. In general, gene expression data can be analyzed at three levels: the first level is the single gene level, which is the difference between the two experimental conditions; the second level is the analysis of functionally similar genes and interactions between genes; and the third level is research based on gene and protein networks (40). The most critical step is screening for potential DEGs from the expression profile (41). Our ceRNA network was established based on differentially expressed lncRNAs, miRNAs, and mRNAs; thus, the genes in our ceRNA network were more statistically and biologically significant. In our study, to identify the most significant interactions in the ceRNA of CCA and to cover as many interactions as possible, we set the thresholds for screening DEGs uniformly to FDR <0.05 and |log FC| >1. Although the lncRNA SPRY4-IT1/miR-101-3p and LINC01296/miR-5095 interactions were not included in the current ceRNA network, this deficiency did not impede the reliability of the ceRNA network, because our network was based on rigorous processes. First, we only included cancer-specific lncRNAs, miRNAs, and mRNAs with thresholds of FDR <0.05 and |log FC| >1. Second, the interactions between the DElncRNAs and DEmiRNAs and between the DEmiRNAs and DEmRNAs were predicted by experiment-supported databases, such as miRTarBase. These two methods ensured that the identified interactions would not only occur in computer simulations, but also based on evidence from experimental support. Therefore, we believe that the genes in the current ceRNA network are more reliable. In the future, with the emergence of better algorithms, better databases, and larger sample sizes, a more comprehensive ceRNA network will be established.

In conclusion, we built a ceRNA network mediated by dysregulated lncRNA in CCA on the basis of the “ceRNA hypothesis” through which the gene regulation mediated by lncRNAs in CCA can be comprehensively analyzed at the system level. This study provides a better understanding of the regulatory mechanism of ceRNAs mediated by lncRNA in CCA. Additionally, novel lncRNAs, miRNAs and mRNAs could be useful candidate diagnostic biomarkers or serve as potential therapeutic targets.

## Data Availability

The datasets analyzed in this study are available in The Cancer Genome Atlas (TCGA) and Gene Expression Omnibus (GEO) public repository.

## Ethics Statement

This research project was approved by the Ethics Committee of Peking Union Medical College Hospital. Written consents were obtained from each patient.

## Author Contributions

HZhao and SC were the principal investigators who designed and conceived the study and obtained financial support. JLo, JX, and YB analyzed the data and wrote the manuscript. JM, JLi, WX, and HZhang prepared the dataset. All authors have read, revised, and approved the final manuscript.

### Conflict of Interest Statement

The authors declare that the research was conducted in the absence of any commercial or financial relationships that could be construed as a potential conflict of interest.
