# Supplementary material for: Construction and Investigation of a lncRNA-Associated ceRNA Regulatory Network in Cholangiocarcinoma
Source: Front Oncol. 2019 Aug 9;9:649. doi: 10.3389/fonc.2019.00649 (PMC6696726; doi:10.3389/fonc.2019.00649)
Supplement: Supplementary file 1 [file Data_Sheet_1.PDF]

Table S1. Samples included from the TCGA database.

CCA sample (n=45)	Adjacent non-tumorous sample (n=9)
TCGA-3X-AAVE-01A-11R-A41I-07	
TCGA-W5-AA2G-01A-11R-A41I-07	
TCGA-W5-AA2X-01A-11R-A41I-07	TCGA-W5-AA2X-11A-11R-A41I-07
TCGA-ZH-A8Y6-01A-11R-A41I-07	
TCGA-W5-AA2R-01A-11R-A41I-07	TCGA-W5-AA2R-11A-11R-A41I-07
TCGA-W5-AA2U-01A-11R-A41I-07	TCGA-W5-AA2U-11A-11R-A41I-07
TCGA-3X-AAVA-01A-11R-A41I-07	
TCGA-3X-AAVB-01A-31R-A41I-07	
TCGA-4G-AAZO-01A-12R-A41I-07	
TCGA-W5-AA36-01A-11R-A41I-07	
TCGA-ZH-A8Y1-01A-11R-A41I-07	
TCGA-4G-AAZT-01A-11R-A41I-07	
TCGA-W5-AA30-01A-31R-A41I-07	TCGA-W5-AA30-11A-11R-A41I-07
TCGA-3X-AAV9-01A-72R-A41I-07	
TCGA-W5-AA2W-01A-11R-A41I-07	
TCGA-3X-AAVC-01A-21R-A41I-07	
TCGA-W5-AA33-01A-11R-A41I-07	
TCGA-W5-AA39-01A-11R-A41I-07	
TCGA-W5-AA2H-01A-31R-A41I-07	
TCGA-WD-A7RX-01A-12R-A41I-07	
TCGA-W5-AA2T-01A-12R-A41I-07	
TCGA-W5-AA2Q-01A-11R-A41I-07	TCGA-W5-AA2Q-11A-11R-A41I-07
TCGA-W6-AA0S-01A-11R-A41I-07	
TCGA-YR-A95A-01A-12R-A41I-07	
TCGA-W5-AA34-01A-11R-A41I-07	TCGA-W5-AA34-11A-11R-A41I-07
TCGA-W5-AA2Z-01A-11R-A41I-07	
TCGA-W5-AA38-01A-11R-A41I-07	
TCGA-ZH-A8Y4-01A-11R-A41I-07	
TCGA-ZD-A8I3-01A-11R-A41I-07	
TCGA-ZH-A8Y2-01A-11R-A41I-07	
TCGA-W5-AA31-01A-11R-A41I-07	TCGA-W5-AA31-11A-11R-A41I-07
TCGA-W5-AA2O-01A-11R-A41I-07	
TCGA-ZH-A8Y8-01A-51R-A41I-07	
TCGA-W5-AA2I-01A-32R-A41I-07	TCGA-W5-AA2I-11A-11R-A41I-07
TCGA-ZH-A8Y5-01A-11R-A41I-07	
TCGA-ZU-A8S4-01A-11R-A41I-07	TCGA-ZU-A8S4-11A-11R-A41I-07

Table S2. Differentially expressed gene between cholangiocarcinoma samples and adjacent non-tumorous samples.

Gene	Type	Log fold change	P value	Adjusted P value
CPS1-IT1	lncRNA	-8.366650566	3.31E-50	2.46E-46
AC010280.2	lncRNA	-6.074674061	7.16E-47	2.65E-43
AC107396.1	lncRNA	-6.765607744	4.03E-46	9.97E-43
LINC01863	lncRNA	-6.358154102	2.40E-43	4.44E-40
AC137723.1	lncRNA	-5.71153596	4.66E-39	6.92E-36

HAO2-IT1	lncRNA	-6.127042	1.00E-38	1.24E-35
LINC01818	lncRNA	-6.818685981	5.68E-38	6.02E-35
LINC01780	lncRNA	-5.175946894	1.05E-37	9.71E-35
LINC02153	lncRNA	-6.272979345	1.94E-37	1.52E-34
AC015468.1	lncRNA	-5.588533565	2.04E-37	1.52E-34
LINC02362	lncRNA	-5.899223173	3.14E-37	2.12E-34
AC120042.1	lncRNA	-5.462460382	1.96E-36	1.21E-33
AL592182.2	lncRNA	-6.248190645	2.70E-36	1.54E-33
AC012313.3	lncRNA	-4.232138664	2.80E-35	1.48E-32
AC010280.1	lncRNA	-6.320660768	3.06E-34	1.51E-31
AC079598.1	lncRNA	-7.836403988	9.71E-34	4.50E-31
AC068631.1	lncRNA	-4.389356884	2.70E-33	1.18E-30
CR936218.2	lncRNA	-6.205021676	5.93E-33	2.43E-30
AP001043.1	lncRNA	-4.976245876	6.23E-33	2.43E-30
AP003716.1	lncRNA	-5.811854882	8.38E-33	3.11E-30
AL354872.2	lncRNA	-5.079358554	1.25E-32	4.40E-30
AC008708.1	lncRNA	-6.271632995	1.03E-30	3.49E-28
AC106822.1	lncRNA	-5.621010282	1.12E-30	3.62E-28
AL359915.1	lncRNA	-5.551002852	1.77E-30	5.48E-28
AL356056.3	lncRNA	-4.694405944	2.44E-30	7.24E-28
AC092155.1	lncRNA	-5.89996829	2.60E-30	7.41E-28
AC004160.1	lncRNA	-5.260699053	3.78E-30	1.04E-27
LINC02348	lncRNA	-6.043371912	8.18E-30	2.17E-27
TPRG1-AS1	lncRNA	-4.549545614	9.04E-30	2.31E-27
AL161668.3	lncRNA	-5.376652357	1.23E-29	3.04E-27
AC099508.2	lncRNA	-6.066464627	1.56E-29	3.73E-27
AC020978.4	lncRNA	-4.555912866	5.20E-29	1.21E-26
LINC01767	lncRNA	-5.271935189	4.79E-28	1.08E-25
LINC01625	lncRNA	-5.783138501	5.70E-28	1.24E-25
AC004160.2	lncRNA	-5.2429242	7.48E-28	1.58E-25
AC116025.1	lncRNA	-5.216122486	1.11E-27	2.29E-25
AP006285.2	lncRNA	-6.205443431	3.75E-27	7.52E-25
AC026765.2	lncRNA	-5.189915483	4.25E-27	8.30E-25
AL161740.1	lncRNA	-5.221539588	7.32E-27	1.39E-24
AC244100.3	lncRNA	-4.617554644	7.77E-27	1.44E-24
AC079360.1	lncRNA	-5.513916648	2.08E-26	3.76E-24
AC012313.9	lncRNA	-3.3603471	2.40E-26	4.24E-24
AC007991.3	lncRNA	-5.783778022	2.96E-26	5.10E-24
PCDH9-AS2	lncRNA	-6.166174486	4.01E-26	6.76E-24
LINC00399	lncRNA	-5.314521288	5.16E-26	8.51E-24
MIR4290HG	lncRNA	-5.493766347	5.33E-26	8.60E-24
AC243836.1	lncRNA	-4.899513371	5.94E-26	9.36E-24
AP001783.1	lncRNA	-6.139791398	9.69E-26	1.50E-23
AC004832.5	lncRNA	-3.338136609	1.53E-25	2.32E-23
SPATA41	lncRNA	-3.589784322	3.02E-25	4.47E-23
AP003471.1	lncRNA	-5.112113305	3.78E-25	5.50E-23
AL023581.2	lncRNA	-3.417019701	9.07E-25	1.29E-22

AC062004.1	lncRNA	-4.941214355	3.61E-24	5.05E-22
AL138749.1	lncRNA	-5.620393455	3.75E-24	5.15E-22
AL117382.2	lncRNA	-4.732722658	5.97E-24	8.05E-22
AC007666.1	lncRNA	-3.943918738	1.22E-23	1.62E-21
AC097639.1	lncRNA	-3.355384167	1.73E-23	2.25E-21
LINC01744	lncRNA	-4.430115388	2.29E-23	2.90E-21
AC138356.1	lncRNA	-4.024112219	2.31E-23	2.90E-21
AC022784.6	lncRNA	-6.132835256	4.05E-23	5.01E-21
AC036176.1	lncRNA	-2.803119691	5.44E-23	6.61E-21
AL139385.1	lncRNA	-3.748562864	6.84E-23	8.12E-21
HS1BP3-IT1	lncRNA	-4.285807378	6.90E-23	8.12E-21
AC069294.1	lncRNA	-4.134410742	1.44E-22	1.67E-20
AL355974.2	lncRNA	-4.440579987	1.47E-22	1.67E-20
AC083902.1	lncRNA	-5.436133403	1.50E-22	1.69E-20
AL928921.1	lncRNA	-5.109415932	2.46E-22	2.73E-20
AP006216.2	lncRNA	-5.995576177	2.61E-22	2.84E-20
LY6E-DT	lncRNA	-3.691929499	3.00E-22	3.23E-20
AC021242.2	lncRNA	-5.166834041	3.99E-22	4.23E-20
LINC01831	lncRNA	-6.560335394	4.69E-22	4.89E-20
AC015468.4	lncRNA	-5.515940119	1.23E-21	1.27E-19
AL157832.2	lncRNA	-4.836759295	1.31E-21	1.33E-19
AC119424.1	lncRNA	-5.537021103	1.34E-21	1.33E-19
HNF4A-AS1	lncRNA	-6.417215392	1.35E-21	1.33E-19
AC104809.1	lncRNA	-7.717179308	1.62E-21	1.58E-19
DBH-AS1	lncRNA	-3.662535728	1.68E-21	1.62E-19
AC007406.2	lncRNA	-4.4420887	1.89E-21	1.80E-19
AC012065.3	lncRNA	-3.631445935	2.51E-21	2.35E-19
AC099668.1	lncRNA	-3.966408657	4.44E-21	4.11E-19
LINC01554	lncRNA	-6.685021515	4.49E-21	4.11E-19
AL592494.1	lncRNA	-4.161438878	5.20E-21	4.70E-19
LINC01146	lncRNA	-5.129575168	6.11E-21	5.45E-19
AC087045.2	lncRNA	-3.968459215	6.68E-21	5.89E-19
AC099509.1	lncRNA	-4.262510128	1.41E-20	1.23E-18
AC005304.2	lncRNA	-5.117460518	1.94E-20	1.67E-18
F11-AS1	lncRNA	-4.163933975	2.37E-20	2.02E-18
MIR99AHG	lncRNA	-3.146070417	2.53E-20	2.13E-18
LINC02453	lncRNA	-2.94736173	2.84E-20	2.36E-18
LINC00907	lncRNA	-3.914226259	3.19E-20	2.63E-18
AP001065.1	lncRNA	-3.488079185	3.44E-20	2.80E-18
SPINT1-AS1	lncRNA	4.216114062	8.62E-20	6.87E-18
AC244100.4	lncRNA	-4.452920225	8.68E-20	6.87E-18
AC005180.1	lncRNA	-4.07305554	8.70E-20	6.87E-18
AC073321.1	lncRNA	-4.836827148	1.35E-19	1.05E-17
AP000253.1	lncRNA	-3.00050236	1.67E-19	1.29E-17
AC026469.1	lncRNA	-3.618262358	1.70E-19	1.30E-17
AC008592.4	lncRNA	-3.920598332	2.01E-19	1.52E-17
AL360007.1	lncRNA	-5.445345918	2.07E-19	1.55E-17

AC092384.2	lncRNA	-4.412955815	2.24E-19	1.66E-17
AC079061.1	lncRNA	-4.945917424	2.33E-19	1.71E-17
AC006205.2	lncRNA	-5.406786708	2.76E-19	2.01E-17
AC005180.2	lncRNA	-3.767191742	3.41E-19	2.45E-17
AC007423.1	lncRNA	-6.02554005	5.44E-19	3.88E-17
AC015908.3	lncRNA	-3.73184782	5.77E-19	4.07E-17
OGFR-AS1	lncRNA	-2.369556552	1.03E-18	7.23E-17
TMEM220-AS1	lncRNA	-3.660181818	1.08E-18	7.50E-17
AC007298.2	lncRNA	-5.290497329	1.57E-18	1.07E-16
AL161645.1	lncRNA	-4.202471457	1.57E-18	1.07E-16
AC099684.1	lncRNA	-4.016462955	2.05E-18	1.38E-16
AL023755.1	lncRNA	-4.358337029	2.08E-18	1.39E-16
MAFG-AS1	lncRNA	3.838759518	2.40E-18	1.59E-16
AC026461.2	lncRNA	-5.004760134	3.00E-18	1.97E-16
AL133279.1	lncRNA	-4.53716136	3.46E-18	2.25E-16
LINC01018	lncRNA	-5.931689409	3.61E-18	2.32E-16
LINC00526	lncRNA	-2.207368645	4.22E-18	2.69E-16
LINC02160	lncRNA	-5.460624648	7.34E-18	4.65E-16
LINC01127	lncRNA	-5.007908523	7.68E-18	4.82E-16
PITPNM2-AS1	lncRNA	-5.095155802	8.36E-18	5.21E-16
AL359715.3	lncRNA	-2.866786727	1.09E-17	6.75E-16
AJ009632.2	lncRNA	-5.568966106	1.61E-17	9.84E-16
AP003119.3	lncRNA	-3.540332505	2.19E-17	1.33E-15
U91324.1	lncRNA	-5.794194492	3.22E-17	1.94E-15
AL357055.3	lncRNA	-2.695135249	3.50E-17	2.09E-15
AL121992.3	lncRNA	-4.006132629	3.63E-17	2.14E-15
AC138430.1	lncRNA	-4.055213181	3.64E-17	2.14E-15
FAM83A-AS1	lncRNA	-6.767921819	5.49E-17	3.20E-15
BOK-AS1	lncRNA	-3.757774319	6.78E-17	3.93E-15
AC090877.2	lncRNA	-4.024526579	7.62E-17	4.38E-15
AC087392.1	lncRNA	-4.461798042	7.91E-17	4.51E-15
AL121827.1	lncRNA	-6.292416693	8.18E-17	4.63E-15
BX547991.1	lncRNA	-4.093114882	8.42E-17	4.73E-15
AC005280.1	lncRNA	-3.294851633	9.37E-17	5.22E-15
AC026202.2	lncRNA	-2.70205527	1.54E-16	8.50E-15
LINC01485	lncRNA	-5.21144459	1.56E-16	8.59E-15
AP001065.2	lncRNA	-4.098121101	1.70E-16	9.25E-15
AC091729.2	lncRNA	-4.806206733	2.10E-16	1.14E-14
NDUFA6-AS1	lncRNA	-2.868713158	2.16E-16	1.16E-14
AP001781.1	lncRNA	-4.157149528	2.48E-16	1.32E-14
AC254562.2	lncRNA	-3.633576979	2.55E-16	1.35E-14
AC105105.3	lncRNA	-3.782027371	4.27E-16	2.25E-14
AC063919.1	lncRNA	-4.271408686	4.93E-16	2.57E-14
AL512274.1	lncRNA	5.55330032	5.07E-16	2.63E-14
AC009166.1	lncRNA	-4.255912386	6.35E-16	3.27E-14
AP001198.2	lncRNA	-5.304005556	6.47E-16	3.31E-14
AL590483.2	lncRNA	-4.949731363	8.27E-16	4.20E-14

LINC02028	lncRNA	-3.526381593	8.62E-16	4.35E-14
LINC01348	lncRNA	-4.946370822	9.22E-16	4.62E-14
AC020558.1	lncRNA	-2.953912427	1.03E-15	5.14E-14
AC046158.1	lncRNA	-3.831997534	1.10E-15	5.45E-14
LINC00242	lncRNA	-2.617503596	1.13E-15	5.56E-14
TMC3-AS1	lncRNA	-2.82336815	1.27E-15	6.19E-14
LINC01816	lncRNA	-2.972142333	1.35E-15	6.57E-14
AF127577.4	lncRNA	-2.45195953	1.40E-15	6.76E-14
AC114810.1	lncRNA	-2.492345435	1.67E-15	7.99E-14
KLHL6-AS1	lncRNA	-4.101697006	2.00E-15	9.50E-14
AC007298.1	lncRNA	-4.157630198	2.10E-15	9.94E-14
AL031055.1	lncRNA	-2.748544882	2.46E-15	1.15E-13
AC021744.1	lncRNA	-5.294621647	3.54E-15	1.65E-13
MIR4435-2HG	lncRNA	2.848745489	3.62E-15	1.68E-13
LINC01727	lncRNA	-4.965531065	3.75E-15	1.73E-13
AGAP1-IT1	lncRNA	-3.008628195	4.07E-15	1.86E-13
AC008250.2	lncRNA	-4.340756519	4.66E-15	2.12E-13
MLIP-AS1	lncRNA	-4.782942459	5.34E-15	2.42E-13
MAGI2-AS3	lncRNA	-2.647887695	7.40E-15	3.33E-13
AC009093.5	lncRNA	-3.551216975	7.70E-15	3.44E-13
LRRC3-AS1	lncRNA	-3.591112577	7.83E-15	3.46E-13
AC010336.2	lncRNA	-3.31287994	7.85E-15	3.46E-13
AL031722.1	lncRNA	-2.868207052	9.20E-15	4.03E-13
LINC00511	lncRNA	4.938705442	1.68E-14	7.34E-13
AC011591.1	lncRNA	-4.674322797	1.71E-14	7.42E-13
AC006960.2	lncRNA	-6.204087031	1.87E-14	8.05E-13
AC108860.2	lncRNA	4.67837637	1.98E-14	8.48E-13
MNX1-AS1	lncRNA	8.402358964	2.11E-14	9.01E-13
AC055720.2	lncRNA	-2.475064136	2.21E-14	9.38E-13
LINC02156	lncRNA	-4.100596813	2.89E-14	1.22E-12
AC018467.1	lncRNA	-4.907460281	3.20E-14	1.34E-12
AC022816.1	lncRNA	-4.774852811	4.15E-14	1.73E-12
LDLRAD4-AS1	lncRNA	-4.362670798	4.89E-14	2.02E-12
MELTF-AS1	lncRNA	3.249275867	6.01E-14	2.48E-12
ITIH4-AS1	lncRNA	-3.457956224	7.11E-14	2.91E-12
AC004816.2	lncRNA	-3.388500771	7.76E-14	3.14E-12
AC092071.1	lncRNA	-5.376819859	8.09E-14	3.26E-12
AC083841.1	lncRNA	-4.173591792	8.51E-14	3.41E-12
AC135048.1	lncRNA	-2.350508715	8.67E-14	3.46E-12
AC008592.5	lncRNA	-4.058489239	1.06E-13	4.21E-12
Z92544.2	lncRNA	3.411369213	1.14E-13	4.49E-12
AC103740.1	lncRNA	-3.356644787	1.23E-13	4.84E-12
LOXL1-AS1	lncRNA	3.701782058	1.53E-13	5.96E-12
LINC02275	lncRNA	-4.733380802	1.57E-13	6.11E-12
LINC01702	lncRNA	-5.541986013	1.59E-13	6.14E-12
AC126614.1	lncRNA	-3.92991077	1.68E-13	6.46E-12
LINC01151	lncRNA	-4.277185468	1.73E-13	6.61E-12

LAMA5-AS1	lncRNA	-3.073387693	1.92E-13	7.31E-12
AL354919.1	lncRNA	-3.723862212	2.12E-13	8.04E-12
AL355096.1	lncRNA	-4.382986424	2.22E-13	8.34E-12
AC015917.2	lncRNA	-3.034626435	2.60E-13	9.74E-12
LINC01714	lncRNA	-3.910308221	2.65E-13	9.89E-12
FGF14-AS2	lncRNA	-2.034841669	2.68E-13	9.95E-12
LINC01093	lncRNA	-7.000268998	2.90E-13	1.07E-11
AC096564.1	lncRNA	-3.009813276	3.01E-13	1.10E-11
LINC02027	lncRNA	-5.654352801	3.18E-13	1.16E-11
AC091114.1	lncRNA	-3.219612006	3.29E-13	1.20E-11
AL365181.3	lncRNA	5.562203014	4.28E-13	1.55E-11
AP000439.3	lncRNA	-3.491755564	4.40E-13	1.58E-11
AC008549.1	lncRNA	-6.548619653	4.70E-13	1.68E-11
AL049555.1	lncRNA	5.27316373	4.71E-13	1.68E-11
ALDH1L1-AS1	lncRNA	-3.892565665	4.72E-13	1.68E-11
AC009159.3	lncRNA	-2.268813338	5.36E-13	1.89E-11
AC012409.1	lncRNA	-3.070302164	6.74E-13	2.37E-11
AC137056.1	lncRNA	-3.930867629	6.98E-13	2.44E-11
AC115619.1	lncRNA	-4.705288946	7.01E-13	2.44E-11
AC102953.2	lncRNA	2.743960772	7.40E-13	2.56E-11
LINC01344	lncRNA	-3.562988782	8.47E-13	2.91E-11
AL360181.1	lncRNA	-3.518814659	8.83E-13	3.02E-11
AL121972.1	lncRNA	-2.878456877	8.96E-13	3.05E-11
HM13-AS1	lncRNA	-2.817580493	9.79E-13	3.30E-11
AC021074.3	lncRNA	-6.284126252	1.05E-12	3.53E-11
AC021491.4	lncRNA	-2.830875403	1.10E-12	3.68E-11
TFAP2A-AS1	lncRNA	4.881637713	1.14E-12	3.80E-11
LINC02037	lncRNA	-5.669087467	1.19E-12	3.94E-11
FAM222A-AS1	lncRNA	-3.244756431	1.33E-12	4.39E-11
AC104785.1	lncRNA	-2.729660969	1.36E-12	4.46E-11
AC231981.1	lncRNA	-2.248421535	1.74E-12	5.67E-11
LINC01847	lncRNA	-4.034626919	1.91E-12	6.22E-11
AC020763.1	lncRNA	-2.831986584	1.92E-12	6.22E-11
AC090164.3	lncRNA	-2.844997887	2.14E-12	6.85E-11
AC024651.1	lncRNA	-5.018995457	2.14E-12	6.85E-11
NADK2-AS1	lncRNA	-2.941280722	2.22E-12	7.08E-11
LINC02289	lncRNA	-2.620661699	2.50E-12	7.92E-11
ALDH1L1-AS2	lncRNA	-3.792312229	2.76E-12	8.70E-11
AC239868.1	lncRNA	2.947811607	3.01E-12	9.47E-11
AL390778.2	lncRNA	-5.490968595	3.13E-12	9.80E-11
AC009137.2	lncRNA	-3.093526075	3.25E-12	1.01E-10
AC040970.1	lncRNA	3.151302558	3.78E-12	1.17E-10
LINC01428	lncRNA	-4.289056641	4.06E-12	1.25E-10
LINC02259	lncRNA	-2.752145582	4.15E-12	1.28E-10
AC099684.2	lncRNA	-3.274704845	4.19E-12	1.28E-10
AL358613.2	lncRNA	-3.627070122	4.30E-12	1.31E-10
RNF157-AS1	lncRNA	3.937649539	4.38E-12	1.33E-10

LINC02298	lncRNA	3.43429419	5.17E-12	1.56E-10
LINC00261	lncRNA	-3.349178597	5.37E-12	1.62E-10
LINC02428	lncRNA	-5.460514484	5.44E-12	1.63E-10
AL772337.1	lncRNA	-4.681990211	5.47E-12	1.63E-10
AC078864.1	lncRNA	-3.399860715	6.26E-12	1.86E-10
CYTOR	lncRNA	2.712560025	6.43E-12	1.90E-10
DDX11-AS1	lncRNA	3.097739864	6.48E-12	1.91E-10
FAM99B	lncRNA	-5.974130913	6.64E-12	1.95E-10
JAKMIP2-AS1	lncRNA	-4.139955105	6.85E-12	1.99E-10
THRB-AS1	lncRNA	-2.604992011	8.76E-12	2.53E-10
PRKAG2-AS1	lncRNA	-2.193008821	8.89E-12	2.55E-10
AC004130.1	lncRNA	3.081620065	1.10E-11	3.13E-10
AC015689.1	lncRNA	-3.249344908	1.10E-11	3.13E-10
AP006285.1	lncRNA	-5.535082993	1.11E-11	3.13E-10
AC104170.1	lncRNA	-2.575757315	1.19E-11	3.35E-10
LINC00337	lncRNA	-2.485363386	1.19E-11	3.35E-10
LINC01277	lncRNA	-2.261799812	1.21E-11	3.38E-10
LINC02499	lncRNA	-5.735053754	1.22E-11	3.41E-10
AC105942.1	lncRNA	-2.125652787	1.46E-11	4.05E-10
AL606970.3	lncRNA	-3.410500248	1.47E-11	4.08E-10
AC008741.2	lncRNA	2.255524282	1.51E-11	4.17E-10
LINC01621	lncRNA	-4.747201403	1.53E-11	4.19E-10
LINC01482	lncRNA	-3.198799964	1.55E-11	4.24E-10
AC009974.1	lncRNA	-2.866312276	1.75E-11	4.75E-10
AC008760.1	lncRNA	3.404146949	1.75E-11	4.75E-10
AL162293.1	lncRNA	-4.346003054	1.81E-11	4.89E-10
CAPN10-AS1	lncRNA	2.390120698	1.93E-11	5.21E-10
AC022784.3	lncRNA	-2.793469872	1.94E-11	5.21E-10
AP003721.4	lncRNA	-2.341482859	2.03E-11	5.45E-10
AL590652.1	lncRNA	4.427862567	2.09E-11	5.58E-10
AC092802.3	lncRNA	-3.048781108	2.68E-11	7.11E-10
AC004832.4	lncRNA	-2.652927994	2.82E-11	7.46E-10
AL163636.1	lncRNA	-2.475335669	3.06E-11	8.03E-10
AC128688.2	lncRNA	4.333914048	3.06E-11	8.03E-10
LINC01121	lncRNA	5.008328684	3.07E-11	8.03E-10
RAB11B-AS1	lncRNA	-2.012580821	3.08E-11	8.03E-10
AC008760.2	lncRNA	-3.334477528	3.15E-11	8.19E-10
AC078909.2	lncRNA	5.117823633	3.43E-11	8.86E-10
LINC01900	lncRNA	-3.68672909	3.49E-11	8.98E-10
LINC01011	lncRNA	2.135379235	4.63E-11	1.19E-09
SNHG1	lncRNA	2.033816079	4.74E-11	1.21E-09
AP000851.2	lncRNA	-4.568792214	4.77E-11	1.22E-09
AC016687.3	lncRNA	-3.841712258	4.94E-11	1.25E-09
PVT1	lncRNA	3.539424771	6.51E-11	1.64E-09
FAM66C	lncRNA	2.908511736	6.66E-11	1.67E-09
AL133419.1	lncRNA	-5.10708843	6.66E-11	1.67E-09
AP003419.3	lncRNA	3.865868378	7.27E-11	1.81E-09

AL590666.2	lncRNA	4.453474427	7.42E-11	1.85E-09
AC114947.2	lncRNA	-2.290599847	8.39E-11	2.08E-09
AC009119.3	lncRNA	-2.887706588	8.64E-11	2.13E-09
AC105219.4	lncRNA	6.297808267	8.69E-11	2.14E-09
AC090227.2	lncRNA	-3.966101056	9.30E-11	2.28E-09
AC004812.2	lncRNA	2.136237496	9.49E-11	2.32E-09
LINC02388	lncRNA	-3.225980077	1.31E-10	3.17E-09
AF165147.1	lncRNA	-2.824986312	1.39E-10	3.37E-09
FAM99A	lncRNA	-6.403011706	1.52E-10	3.65E-09
AL359878.1	lncRNA	3.395917349	1.70E-10	4.07E-09
LINC01725	lncRNA	-2.746910867	1.80E-10	4.30E-09
LINC02073	lncRNA	-3.194466307	2.15E-10	5.14E-09
AC090150.1	lncRNA	-3.791024924	2.31E-10	5.48E-09
AC099329.1	lncRNA	-3.000241131	2.33E-10	5.52E-09
LINC00941	lncRNA	5.926834165	2.39E-10	5.63E-09
AC092171.2	lncRNA	2.284157055	2.39E-10	5.63E-09
B4GALT1-AS1	lncRNA	-2.160963264	2.45E-10	5.74E-09
AC012379.1	lncRNA	-4.492196434	2.45E-10	5.74E-09
AC005225.2	lncRNA	-3.005668316	2.55E-10	5.96E-09
RNFT1-DT	lncRNA	-2.038984234	2.60E-10	6.03E-09
LINC01963	lncRNA	2.52825762	2.66E-10	6.16E-09
AC006504.7	lncRNA	-4.663358524	2.80E-10	6.44E-09
AC010205.1	lncRNA	-2.767258859	2.81E-10	6.45E-09
CRNDE	lncRNA	3.639326006	3.20E-10	7.32E-09
AL117379.1	lncRNA	2.702438123	3.38E-10	7.71E-09
AL080248.1	lncRNA	-4.143096344	3.39E-10	7.71E-09
LINC01123	lncRNA	3.387272427	3.54E-10	8.01E-09
LINC02482	lncRNA	-2.481469074	3.60E-10	8.11E-09
AF111167.2	lncRNA	-2.132741727	3.97E-10	8.89E-09
GAS5	lncRNA	2.294206181	4.04E-10	9.02E-09
AC025754.2	lncRNA	-2.25954504	4.09E-10	9.10E-09
AC092378.1	lncRNA	-3.072799809	4.14E-10	9.20E-09
AL157756.1	lncRNA	-2.057126811	4.27E-10	9.46E-09
AL157373.2	lncRNA	-2.576391155	4.42E-10	9.75E-09
AC024600.1	lncRNA	-3.78236085	4.48E-10	9.86E-09
LINC01370	lncRNA	-5.615417675	4.66E-10	1.02E-08
AL365181.2	lncRNA	5.873959341	4.93E-10	1.08E-08
AC016705.2	lncRNA	4.372167012	6.11E-10	1.33E-08
AC008105.1	lncRNA	3.052391774	6.85E-10	1.49E-08
AL117335.1	lncRNA	3.072161499	6.93E-10	1.50E-08
LINC02266	lncRNA	-4.266376848	7.62E-10	1.64E-08
AFAP1-AS1	lncRNA	8.743640467	7.63E-10	1.64E-08
AL109615.3	lncRNA	3.526775064	7.67E-10	1.65E-08
LINC02139	lncRNA	-2.678637504	8.78E-10	1.88E-08
AC116036.2	lncRNA	-2.548247215	9.61E-10	2.05E-08
AC108134.1	lncRNA	3.589237903	1.01E-09	2.15E-08
AL161630.1	lncRNA	-4.084653286	1.09E-09	2.31E-08

AC005083.1	lncRNA	3.162434816	1.09E-09	2.31E-08
ERLNC1	lncRNA	-3.183735459	1.12E-09	2.37E-08
LINC01977	lncRNA	5.005466274	1.31E-09	2.76E-08
NKILA	lncRNA	4.630295333	1.45E-09	3.04E-08
AC008875.1	lncRNA	4.102292027	1.50E-09	3.15E-08
AC083900.1	lncRNA	-2.407985604	1.52E-09	3.17E-08
AC090796.1	lncRNA	-4.229235912	1.52E-09	3.17E-08
AC010969.1	lncRNA	-4.082740538	1.53E-09	3.17E-08
AC008764.8	lncRNA	2.326524212	1.64E-09	3.40E-08
MIR583HG	lncRNA	-2.56523507	1.70E-09	3.51E-08
AL355388.2	lncRNA	3.543638107	1.79E-09	3.69E-08
AL109936.2	lncRNA	-2.161739459	1.98E-09	4.05E-08
AL034374.1	lncRNA	-2.893736904	2.17E-09	4.43E-08
AC009560.1	lncRNA	-2.226655011	2.27E-09	4.62E-08
STAM-AS1	lncRNA	3.055858529	2.38E-09	4.82E-08
AC017104.1	lncRNA	3.439133038	2.46E-09	4.98E-08
OBSCN-AS1	lncRNA	3.162587316	2.58E-09	5.22E-08
TMEM51-AS1	lncRNA	3.654099618	2.68E-09	5.39E-08
LINC02041	lncRNA	5.678326442	2.68E-09	5.39E-08
AC244021.1	lncRNA	-2.921096078	2.71E-09	5.43E-08
COL18A1-AS2	lncRNA	-2.946755514	2.90E-09	5.80E-08
AC023908.3	lncRNA	3.102143202	3.02E-09	6.02E-08
NRAV	lncRNA	2.149290362	3.10E-09	6.16E-08
AC019205.1	lncRNA	2.888479016	3.13E-09	6.21E-08
AC011462.4	lncRNA	2.766870339	3.18E-09	6.29E-08
AC092171.4	lncRNA	2.918510568	3.57E-09	7.05E-08
AC092119.2	lncRNA	2.691009126	3.60E-09	7.09E-08
FBXL19-AS1	lncRNA	2.616385896	3.73E-09	7.32E-08
GK-AS1	lncRNA	-2.261959743	3.80E-09	7.43E-08
AC013275.1	lncRNA	5.529636259	4.23E-09	8.25E-08
AC024559.2	lncRNA	-4.284002065	4.28E-09	8.33E-08
AC254633.1	lncRNA	-2.131041899	4.38E-09	8.50E-08
AL445686.2	lncRNA	-2.823853433	4.43E-09	8.58E-08
AC026368.1	lncRNA	4.560486854	5.13E-09	9.88E-08
AC026356.1	lncRNA	2.972262012	5.24E-09	1.01E-07
AL022322.1	lncRNA	2.525856407	5.58E-09	1.07E-07
DISC1FP1	lncRNA	-3.677726585	5.65E-09	1.08E-07
AC004862.1	lncRNA	-4.119542688	5.99E-09	1.14E-07
AC002550.2	lncRNA	2.646771547	6.14E-09	1.16E-07
KANSL1-AS1	lncRNA	-2.094152715	6.35E-09	1.20E-07
AL139383.1	lncRNA	-2.750665046	6.58E-09	1.24E-07
AP000355.1	lncRNA	-4.66630488	6.60E-09	1.24E-07
AC007406.1	lncRNA	-4.184397084	6.72E-09	1.26E-07
AC141928.1	lncRNA	4.581396581	6.74E-09	1.26E-07
AP006284.1	lncRNA	2.365922298	6.84E-09	1.27E-07
AL390719.2	lncRNA	3.215572893	7.29E-09	1.35E-07
AC104411.1	lncRNA	-2.533022796	7.32E-09	1.36E-07

AC147651.1	lncRNA	3.653023117	7.33E-09	1.36E-07
MNX1-AS2	lncRNA	4.718687684	7.54E-09	1.39E-07
AP001626.1	lncRNA	4.536491843	7.95E-09	1.46E-07
LINC01978	lncRNA	3.604169156	8.42E-09	1.55E-07
AC016773.1	lncRNA	3.248341087	8.54E-09	1.56E-07
AC106886.2	lncRNA	2.990223734	8.68E-09	1.59E-07
AC005393.1	lncRNA	3.208681823	8.72E-09	1.59E-07
AL121839.2	lncRNA	2.530491653	8.89E-09	1.62E-07
AL109976.1	lncRNA	4.259326616	9.06E-09	1.64E-07
AC104123.1	lncRNA	-2.71587377	1.01E-08	1.83E-07
AC002401.3	lncRNA	-2.526671022	1.05E-08	1.90E-07
AC090844.2	lncRNA	-2.464403867	1.10E-08	1.98E-07
AC024361.2	lncRNA	-2.146002206	1.10E-08	1.98E-07
AC010201.2	lncRNA	2.316678837	1.18E-08	2.12E-07
AL133215.2	lncRNA	4.06100557	1.23E-08	2.19E-07
AC027688.1	lncRNA	-3.908834108	1.27E-08	2.25E-07
AC012668.3	lncRNA	-3.153650678	1.28E-08	2.27E-07
DGCR9	lncRNA	3.853098937	1.31E-08	2.31E-07
AC007364.1	lncRNA	-2.000083145	1.34E-08	2.36E-07
AC090515.2	lncRNA	2.614243462	1.34E-08	2.36E-07
AC124319.1	lncRNA	3.726677058	1.35E-08	2.38E-07
AF131215.6	lncRNA	3.020391704	1.38E-08	2.41E-07
SNHG4	lncRNA	3.381571475	1.40E-08	2.44E-07
LINC01559	lncRNA	7.809197414	1.59E-08	2.76E-07
AC080129.1	lncRNA	-4.138681401	1.66E-08	2.87E-07
AL731569.1	lncRNA	2.56984067	1.67E-08	2.88E-07
CASC15	lncRNA	3.917390705	1.67E-08	2.88E-07
AC139149.1	lncRNA	3.145926807	1.68E-08	2.88E-07
LINC01106	lncRNA	2.988642202	1.69E-08	2.89E-07
AL606489.1	lncRNA	3.153213813	1.77E-08	3.02E-07
AC021205.3	lncRNA	-2.02274767	1.80E-08	3.06E-07
AC245060.6	lncRNA	2.071178685	1.81E-08	3.08E-07
AC016395.1	lncRNA	-3.455808937	1.81E-08	3.08E-07
LINC02484	lncRNA	-3.880772679	1.82E-08	3.09E-07
AL359541.1	lncRNA	-2.170176516	1.87E-08	3.16E-07
AC016999.1	lncRNA	-3.224787737	1.97E-08	3.32E-07
AL160408.3	lncRNA	-3.559123036	2.01E-08	3.38E-07
AL033527.3	lncRNA	4.558231503	2.03E-08	3.41E-07
LINC00665	lncRNA	3.496523016	2.10E-08	3.51E-07
AC107959.3	lncRNA	4.443841495	2.17E-08	3.62E-07
CPB2-AS1	lncRNA	-2.156757718	2.21E-08	3.67E-07
AC022028.2	lncRNA	6.95084792	2.26E-08	3.73E-07
AL583722.1	lncRNA	4.629669388	2.26E-08	3.74E-07
AL391095.2	lncRNA	-4.117817233	2.30E-08	3.79E-07
AL355488.1	lncRNA	2.104115595	2.33E-08	3.82E-07
AC002398.1	lncRNA	2.335800476	2.49E-08	4.07E-07
AL591895.1	lncRNA	2.321478545	2.55E-08	4.17E-07

ADORA2A-AS1	lncRNA	-3.745424544	2.59E-08	4.22E-07
AC141273.1	lncRNA	-3.112500583	2.60E-08	4.23E-07
AC044839.1	lncRNA	-2.435765075	2.70E-08	4.38E-07
AL445493.3	lncRNA	-2.568398375	2.79E-08	4.52E-07
AL109741.1	lncRNA	-2.264104126	2.80E-08	4.53E-07
AC138904.1	lncRNA	4.689768568	2.86E-08	4.60E-07
AL133410.1	lncRNA	2.78929122	2.90E-08	4.66E-07
AGAP2-AS1	lncRNA	2.555965018	3.00E-08	4.82E-07
LINC02246	lncRNA	-2.434634308	3.04E-08	4.87E-07
LINC01836	lncRNA	3.474367657	3.13E-08	5.00E-07
AL121772.3	lncRNA	2.596247053	3.30E-08	5.25E-07
SPRY4-AS1	lncRNA	3.6154333	3.69E-08	5.83E-07
AC007344.1	lncRNA	-3.384994385	3.77E-08	5.94E-07
AL022476.1	lncRNA	2.750435348	3.87E-08	6.07E-07
Z97832.2	lncRNA	2.145404503	3.89E-08	6.08E-07
AC092757.2	lncRNA	3.791082193	4.00E-08	6.24E-07
AP003469.2	lncRNA	4.461865904	4.08E-08	6.35E-07
USP30-AS1	lncRNA	-2.447261362	4.08E-08	6.35E-07
AC006538.1	lncRNA	2.787941023	4.58E-08	7.11E-07
SLC12A9-AS1	lncRNA	2.910152331	4.62E-08	7.15E-07
AC003965.2	lncRNA	4.569767448	4.75E-08	7.33E-07
AL451085.1	lncRNA	-2.381460115	4.79E-08	7.37E-07
H19	lncRNA	-3.350766421	4.89E-08	7.51E-07
LINC00992	lncRNA	3.776770608	4.97E-08	7.61E-07
BLACAT1	lncRNA	7.269703712	4.99E-08	7.63E-07
AC009148.1	lncRNA	3.012588584	5.05E-08	7.69E-07
AC105219.1	lncRNA	2.710320607	5.07E-08	7.71E-07
POU6F2-AS1	lncRNA	-3.291476109	5.16E-08	7.81E-07
VIM-AS1	lncRNA	2.84047463	5.21E-08	7.87E-07
LINC02015	lncRNA	-3.127457861	5.28E-08	7.95E-07
LINC00844	lncRNA	-4.815680749	5.30E-08	7.96E-07
AL606763.1	lncRNA	3.864526069	5.68E-08	8.48E-07
AC092117.1	lncRNA	2.648981618	5.87E-08	8.73E-07
LINC01354	lncRNA	-2.646510868	5.89E-08	8.76E-07
AL596223.2	lncRNA	4.725099086	6.38E-08	9.45E-07
AC004231.1	lncRNA	6.188848425	6.52E-08	9.65E-07
AL035563.1	lncRNA	2.251124218	6.72E-08	9.92E-07
AL137784.2	lncRNA	2.793402317	6.76E-08	9.96E-07
LINC01956	lncRNA	7.654719974	6.82E-08	1.00E-06
AC004012.1	lncRNA	-2.684642481	7.01E-08	1.03E-06
AC011481.1	lncRNA	2.662133828	7.19E-08	1.05E-06
AL365203.2	lncRNA	2.835201423	7.46E-08	1.09E-06
AC105105.2	lncRNA	-3.64685943	7.50E-08	1.09E-06
AC105219.2	lncRNA	6.537723191	7.56E-08	1.10E-06
AL359232.1	lncRNA	-2.194074328	7.69E-08	1.12E-06
AC092683.1	lncRNA	3.221713144	7.85E-08	1.13E-06
AC110609.1	lncRNA	-2.314517177	7.89E-08	1.14E-06

AP001453.4	lncRNA	2.193755735	8.20E-08	1.18E-06
AL133367.1	lncRNA	2.554399302	8.31E-08	1.19E-06
AF131215.5	lncRNA	2.917515112	8.51E-08	1.22E-06
SPACA6P-AS	lncRNA	4.252704356	9.51E-08	1.36E-06
AC023302.1	lncRNA	4.36418369	9.88E-08	1.41E-06
AC004540.1	lncRNA	3.404420339	1.01E-07	1.44E-06
AC010931.2	lncRNA	5.013019703	1.02E-07	1.45E-06
AC129507.1	lncRNA	-2.294018531	1.03E-07	1.46E-06
LEF1-AS1	lncRNA	4.552788519	1.04E-07	1.47E-06
AC245100.1	lncRNA	5.647376601	1.09E-07	1.53E-06
AC234582.1	lncRNA	2.269871968	1.09E-07	1.53E-06
AL049629.1	lncRNA	5.184763241	1.11E-07	1.56E-06
AL137060.1	lncRNA	2.727288386	1.12E-07	1.57E-06
TMEM26-AS1	lncRNA	-2.706706758	1.13E-07	1.57E-06
AL445649.1	lncRNA	7.685401644	1.13E-07	1.57E-06
AC027796.4	lncRNA	2.184740977	1.13E-07	1.57E-06
HOXB-AS3	lncRNA	3.829580022	1.13E-07	1.57E-06
AC012613.2	lncRNA	-2.695740807	1.15E-07	1.59E-06
AC012640.4	lncRNA	-2.014686056	1.17E-07	1.61E-06
LINC01311	lncRNA	2.319203563	1.17E-07	1.61E-06
AL451060.1	lncRNA	-2.808369264	1.27E-07	1.74E-06
AC010168.2	lncRNA	2.216429748	1.28E-07	1.75E-06
C15orf56	lncRNA	3.520534687	1.29E-07	1.77E-06
SEMA6A-AS2	lncRNA	5.347427984	1.32E-07	1.80E-06
AL626787.1	lncRNA	-2.579543376	1.35E-07	1.83E-06
RUNDC3A-AS1	lncRNA	4.20699263	1.42E-07	1.93E-06
AC008610.1	lncRNA	2.146959665	1.43E-07	1.94E-06
AC048341.2	lncRNA	2.084137217	1.44E-07	1.95E-06
HORMAD2-AS1	lncRNA	-3.82996323	1.50E-07	2.03E-06
AL021392.1	lncRNA	2.772606799	1.51E-07	2.03E-06
SLCO4A1-AS1	lncRNA	5.066175297	1.56E-07	2.10E-06
AC022150.4	lncRNA	3.191527278	1.62E-07	2.17E-06
AC092757.3	lncRNA	2.956467246	1.65E-07	2.21E-06
AC007495.1	lncRNA	-3.467154233	1.69E-07	2.26E-06
AC073573.1	lncRNA	-2.549359501	1.72E-07	2.29E-06
BAALC-AS1	lncRNA	2.638387433	1.80E-07	2.40E-06
TUSC8	lncRNA	-3.740685321	1.83E-07	2.43E-06
NAV2-AS4	lncRNA	-3.302504343	1.86E-07	2.46E-06
LINC00574	lncRNA	-2.447728979	1.94E-07	2.56E-06
CDKN2B-AS1	lncRNA	3.283775866	1.96E-07	2.58E-06
AC112496.1	lncRNA	-2.088292795	1.97E-07	2.58E-06
LINC02313	lncRNA	5.427187889	2.07E-07	2.72E-06
C2-AS1	lncRNA	-2.823453689	2.15E-07	2.82E-06
AC010487.2	lncRNA	4.545412865	2.16E-07	2.82E-06
FAM3D-AS1	lncRNA	-2.994208842	2.19E-07	2.86E-06
AL445472.1	lncRNA	2.829992138	2.25E-07	2.93E-06
AL035252.3	lncRNA	2.195951035	2.28E-07	2.97E-06

AC026356.2	lncRNA	3.086220704	2.29E-07	2.98E-06
AL078590.3	lncRNA	-2.085447155	2.48E-07	3.21E-06
LINC01474	lncRNA	-2.343619749	2.54E-07	3.28E-06
AP001627.1	lncRNA	-2.413342589	2.55E-07	3.28E-06
AC019193.3	lncRNA	-2.513981679	2.57E-07	3.30E-06
MRPS30-DT	lncRNA	2.71457961	2.78E-07	3.57E-06
LINC01726	lncRNA	-3.545182767	2.80E-07	3.58E-06
LINC01012	lncRNA	4.37476198	2.88E-07	3.68E-06
PRR7-AS1	lncRNA	2.640249869	3.03E-07	3.87E-06
AC084864.1	lncRNA	5.956317419	3.09E-07	3.93E-06
AC112206.2	lncRNA	-4.330055277	3.14E-07	3.99E-06
AC099329.2	lncRNA	-2.962507602	3.15E-07	4.00E-06
AL121983.1	lncRNA	-2.346154574	3.18E-07	4.03E-06
AL355102.4	lncRNA	4.236214296	3.21E-07	4.05E-06
AC026250.1	lncRNA	3.277710998	3.24E-07	4.09E-06
AC099850.3	lncRNA	2.960823189	3.26E-07	4.10E-06
LINC01829	lncRNA	4.767833094	3.31E-07	4.16E-06
AC114488.1	lncRNA	3.94062447	3.36E-07	4.22E-06
MIR181A2HG	lncRNA	2.432373562	3.43E-07	4.30E-06
LINC02198	lncRNA	-2.082386032	3.48E-07	4.35E-06
AL645608.8	lncRNA	3.213665308	3.52E-07	4.40E-06
LINC00570	lncRNA	-3.021004984	3.62E-07	4.51E-06
AL008718.2	lncRNA	3.682464912	3.63E-07	4.52E-06
AL359694.2	lncRNA	6.495630378	3.72E-07	4.61E-06
AC132872.2	lncRNA	2.915325586	3.88E-07	4.80E-06
AL670729.1	lncRNA	3.592668743	3.91E-07	4.84E-06
Z99289.1	lncRNA	-2.669143848	4.00E-07	4.92E-06
AC009005.1	lncRNA	3.030706106	4.03E-07	4.96E-06
AL590235.1	lncRNA	2.322543582	4.17E-07	5.11E-06
AL109741.3	lncRNA	-2.040753778	4.25E-07	5.19E-06
AC004076.2	lncRNA	2.159286865	4.35E-07	5.31E-06
AL357079.1	lncRNA	2.248446224	4.46E-07	5.44E-06
AC020661.1	lncRNA	3.273376728	4.50E-07	5.47E-06
AL021707.2	lncRNA	2.203151572	4.69E-07	5.67E-06
AC009414.2	lncRNA	2.9379112	4.74E-07	5.72E-06
AL606807.1	lncRNA	-2.336019271	5.01E-07	6.00E-06
AC069431.1	lncRNA	-2.554305991	5.02E-07	6.00E-06
AC012629.2	lncRNA	-2.624874112	5.03E-07	6.00E-06
COL18A1-AS1	lncRNA	5.428064452	5.03E-07	6.00E-06
AC016866.1	lncRNA	2.781301504	5.18E-07	6.14E-06
FTCD-AS1	lncRNA	-3.307812115	5.38E-07	6.38E-06
AL731533.2	lncRNA	3.580703496	5.41E-07	6.40E-06
AC020916.1	lncRNA	2.115886657	5.63E-07	6.63E-06
AC145423.2	lncRNA	3.12206941	5.77E-07	6.79E-06
AC026992.2	lncRNA	4.105064299	5.91E-07	6.93E-06
AL731571.1	lncRNA	2.155820648	5.96E-07	6.98E-06
AC027228.2	lncRNA	3.457141216	6.24E-07	7.29E-06

AL008721.2	lncRNA	2.458961088	6.73E-07	7.82E-06
AC011815.1	lncRNA	2.159718508	6.82E-07	7.91E-06
AC011365.1	lncRNA	-2.884423668	6.90E-07	7.99E-06
AC116158.1	lncRNA	4.047030072	6.95E-07	8.03E-06
AL591845.1	lncRNA	2.37613752	6.99E-07	8.08E-06
AL024498.1	lncRNA	2.262031015	7.45E-07	8.58E-06
UPP2-IT1	lncRNA	-3.279810913	8.00E-07	9.16E-06
AL355303.1	lncRNA	-2.575790955	8.15E-07	9.31E-06
AC078778.1	lncRNA	2.577336176	8.22E-07	9.37E-06
AC090192.2	lncRNA	7.126723183	8.43E-07	9.60E-06
LINC00870	lncRNA	-2.21480151	8.80E-07	9.99E-06
AC012640.3	lncRNA	-2.943034927	8.94E-07	1.01E-05
AL663023.1	lncRNA	-4.524927554	9.23E-07	1.04E-05
Z69733.1	lncRNA	2.879173781	9.30E-07	1.05E-05
ARHGAP31-AS1	lncRNA	3.549685084	9.46E-07	1.07E-05
FAM201A	lncRNA	2.478046824	9.57E-07	1.08E-05
LINC01615	lncRNA	6.668531078	9.68E-07	1.09E-05
MAFA-AS1	lncRNA	6.688759017	9.75E-07	1.09E-05
AL008582.1	lncRNA	2.611778854	9.77E-07	1.09E-05
AC017002.3	lncRNA	3.393932758	9.81E-07	1.09E-05
RRS1-AS1	lncRNA	-2.306767151	9.81E-07	1.09E-05
AC073842.2	lncRNA	2.267683593	1.01E-06	1.12E-05
LINC01600	lncRNA	3.547405719	1.03E-06	1.14E-05
AL121895.2	lncRNA	3.557759335	1.03E-06	1.14E-05
AL139231.1	lncRNA	-3.24336388	1.07E-06	1.18E-05
AC073174.1	lncRNA	-5.033161408	1.08E-06	1.20E-05
AC006042.1	lncRNA	2.459749994	1.12E-06	1.24E-05
F10-AS1	lncRNA	-2.631878183	1.13E-06	1.25E-05
AC010776.2	lncRNA	-3.077650619	1.14E-06	1.25E-05
AC125807.2	lncRNA	2.160064581	1.18E-06	1.29E-05
AL354707.1	lncRNA	-2.19504188	1.21E-06	1.32E-05
BX470102.1	lncRNA	4.388778119	1.22E-06	1.33E-05
LINC00494	lncRNA	5.949023087	1.23E-06	1.33E-05
LINC00622	lncRNA	2.833877635	1.25E-06	1.36E-05
AL731568.1	lncRNA	-2.567064629	1.26E-06	1.36E-05
LINC00628	lncRNA	3.413287892	1.34E-06	1.44E-05
AC010864.1	lncRNA	2.329958021	1.34E-06	1.44E-05
HOXA-AS3	lncRNA	7.068036731	1.35E-06	1.45E-05
AC091057.3	lncRNA	3.275280716	1.38E-06	1.48E-05
AL022068.1	lncRNA	3.946054879	1.42E-06	1.51E-05
AC027702.1	lncRNA	2.898446577	1.44E-06	1.53E-05
TAT-AS1	lncRNA	-3.014675277	1.45E-06	1.54E-05
LINC01336	lncRNA	3.227598918	1.46E-06	1.55E-05
LINC02575	lncRNA	-3.004740496	1.46E-06	1.55E-05
AC138150.2	lncRNA	2.57230482	1.55E-06	1.63E-05
LINC00327	lncRNA	3.559857716	1.57E-06	1.66E-05
AC116407.2	lncRNA	2.44505446	1.59E-06	1.67E-05

AL139161.1	lncRNA	-2.549186405	1.60E-06	1.68E-05
LHFPL3-AS2	lncRNA	5.281144759	1.64E-06	1.72E-05
AP001922.5	lncRNA	2.855694048	1.65E-06	1.73E-05
AC009720.1	lncRNA	3.582583937	1.66E-06	1.74E-05
LINC00323	lncRNA	4.198401638	1.70E-06	1.77E-05
AC099518.2	lncRNA	4.539747143	1.77E-06	1.83E-05
LINC01833	lncRNA	7.149330491	1.79E-06	1.85E-05
AC131009.2	lncRNA	-2.933063521	1.79E-06	1.85E-05
AC023421.2	lncRNA	5.995378598	1.80E-06	1.86E-05
AC003070.1	lncRNA	2.226776628	1.85E-06	1.90E-05
AC022167.4	lncRNA	-2.45608684	1.86E-06	1.91E-05
AC004520.1	lncRNA	2.546113135	1.90E-06	1.95E-05
LINC02097	lncRNA	3.369888032	1.94E-06	1.98E-05
AL513320.1	lncRNA	2.00966427	1.95E-06	1.98E-05
AP001107.4	lncRNA	2.111133759	1.95E-06	1.98E-05
AC087273.1	lncRNA	-3.240462835	2.01E-06	2.04E-05
AC040160.1	lncRNA	2.324642105	2.02E-06	2.05E-05
AC022784.8	lncRNA	-2.289822854	2.03E-06	2.06E-05
AC147651.2	lncRNA	5.352091105	2.04E-06	2.06E-05
AL121772.1	lncRNA	2.899067635	2.06E-06	2.08E-05
AC092111.1	lncRNA	2.656223949	2.08E-06	2.09E-05
AC080037.1	lncRNA	5.458169523	2.09E-06	2.11E-05
ZMIZ1-AS1	lncRNA	2.503491854	2.21E-06	2.22E-05
LINC01943	lncRNA	2.754150718	2.23E-06	2.24E-05
AC004477.1	lncRNA	2.020294644	2.27E-06	2.26E-05
U73166.1	lncRNA	2.032322644	2.30E-06	2.30E-05
AC148476.1	lncRNA	5.044531363	2.34E-06	2.33E-05
AC005005.3	lncRNA	2.097677707	2.41E-06	2.39E-05
AC105118.1	lncRNA	6.881426449	2.49E-06	2.47E-05
AC027682.6	lncRNA	-2.357512561	2.57E-06	2.53E-05
LINC00923	lncRNA	-2.720083606	2.58E-06	2.54E-05
LINC01537	lncRNA	-2.140117973	2.58E-06	2.54E-05
BX255925.1	lncRNA	2.901456623	2.63E-06	2.57E-05
AP003354.2	lncRNA	3.459166467	2.65E-06	2.59E-05
AC116025.2	lncRNA	5.534047097	2.69E-06	2.62E-05
AC096642.1	lncRNA	2.136408839	2.73E-06	2.66E-05
AC108748.1	lncRNA	-3.254892585	2.75E-06	2.67E-05
FO393415.1	lncRNA	-2.639956986	2.77E-06	2.69E-05
CYP4A22-AS1	lncRNA	3.092826546	2.79E-06	2.70E-05
AC004923.4	lncRNA	2.80232359	2.79E-06	2.70E-05
AC005618.1	lncRNA	3.221960268	2.83E-06	2.73E-05
AL021807.1	lncRNA	4.396510829	2.90E-06	2.80E-05
AP000695.2	lncRNA	3.740099094	2.90E-06	2.80E-05
SIAH2-AS1	lncRNA	-2.087206078	2.96E-06	2.83E-05
AC108676.1	lncRNA	3.106165531	3.05E-06	2.93E-05
U47924.3	lncRNA	2.809674521	3.11E-06	2.97E-05
PTGES2-AS1	lncRNA	3.2961208	3.13E-06	2.98E-05

SLC2A1-AS1	lncRNA	4.144571031	3.14E-06	2.99E-05
AC020907.4	lncRNA	2.540016386	3.16E-06	3.01E-05
COLCA1	lncRNA	2.116617418	3.29E-06	3.13E-05
AP002360.3	lncRNA	2.817467923	3.31E-06	3.13E-05
AC087623.2	lncRNA	3.962652164	3.33E-06	3.14E-05
AC008592.1	lncRNA	-3.370019099	3.36E-06	3.16E-05
AL592211.1	lncRNA	2.897908537	3.43E-06	3.22E-05
AL121832.1	lncRNA	6.009950844	3.44E-06	3.23E-05
CASC8	lncRNA	5.942209196	3.45E-06	3.23E-05
DGCR10	lncRNA	3.32011681	3.46E-06	3.23E-05
AL021068.1	lncRNA	-2.120725924	3.47E-06	3.24E-05
AC084871.2	lncRNA	-2.686892884	3.50E-06	3.26E-05
AC087742.1	lncRNA	2.794986727	3.50E-06	3.26E-05
LINC00113	lncRNA	7.780681434	3.50E-06	3.26E-05
AC096531.2	lncRNA	-2.121446652	3.60E-06	3.35E-05
AC069120.1	lncRNA	4.598694419	3.61E-06	3.35E-05
RNU6ATAC35P	lncRNA	-2.318548316	3.65E-06	3.38E-05
AC091806.1	lncRNA	4.465676079	3.68E-06	3.41E-05
AP000977.1	lncRNA	-2.261534622	3.74E-06	3.45E-05
LINC01342	lncRNA	4.959032412	3.76E-06	3.47E-05
TTC39A-AS1	lncRNA	3.919046733	3.89E-06	3.58E-05
AC079949.2	lncRNA	-2.144649725	3.89E-06	3.58E-05
WARS2-IT1	lncRNA	-2.17841887	3.98E-06	3.65E-05
C17orf82	lncRNA	2.176681275	4.17E-06	3.81E-05
AL121845.4	lncRNA	-2.030108739	4.21E-06	3.85E-05
VPS9D1-AS1	lncRNA	2.04626151	4.27E-06	3.89E-05
LINC00853	lncRNA	2.570188351	4.30E-06	3.91E-05
LINC01948	lncRNA	2.77246891	4.31E-06	3.91E-05
AC010967.1	lncRNA	3.919638574	4.32E-06	3.92E-05
AL162431.2	lncRNA	2.178962013	4.38E-06	3.96E-05
LINC00460	lncRNA	8.73750713	4.46E-06	4.02E-05
AL137145.1	lncRNA	2.584076335	4.52E-06	4.07E-05
AL033384.2	lncRNA	2.732850785	4.55E-06	4.08E-05
AC244034.2	lncRNA	5.612422412	4.67E-06	4.18E-05
LINC02408	lncRNA	5.468511113	4.75E-06	4.24E-05
AC025171.4	lncRNA	2.244774312	5.02E-06	4.47E-05
AC009097.2	lncRNA	4.402438578	5.14E-06	4.57E-05
AC016722.3	lncRNA	2.194094436	5.18E-06	4.60E-05
AL121782.1	lncRNA	3.459801248	5.20E-06	4.60E-05
LINC01186	lncRNA	2.945425568	5.27E-06	4.65E-05
AC002401.2	lncRNA	-2.575602912	5.28E-06	4.66E-05
AL136964.1	lncRNA	5.078314843	5.47E-06	4.82E-05
AC020659.1	lncRNA	3.527026821	5.54E-06	4.88E-05
AC009121.1	lncRNA	3.48128178	5.57E-06	4.90E-05
MLIP-IT1	lncRNA	-3.091846586	5.75E-06	5.04E-05
LINC01116	lncRNA	5.800449686	5.91E-06	5.16E-05
AC011330.2	lncRNA	2.319500994	5.91E-06	5.16E-05

LINC02280	lncRNA	3.488856439	5.92E-06	5.16E-05
AC020922.3	lncRNA	3.61839053	5.94E-06	5.17E-05
AC008514.1	lncRNA	4.935569681	5.95E-06	5.18E-05
LIVAR	lncRNA	-2.730631847	5.98E-06	5.20E-05
AP000911.1	lncRNA	4.055413142	6.26E-06	5.42E-05
AL355472.4	lncRNA	3.606286545	6.30E-06	5.45E-05
AP000864.1	lncRNA	4.576075968	6.39E-06	5.51E-05
IBA57-AS1	lncRNA	3.356504489	6.57E-06	5.66E-05
DUBR	lncRNA	2.017973764	6.64E-06	5.71E-05
UCA1	lncRNA	5.351072236	6.87E-06	5.89E-05
AP000812.1	lncRNA	-2.630355402	6.87E-06	5.89E-05
AC009955.2	lncRNA	6.99649192	6.88E-06	5.89E-05
AC127024.6	lncRNA	2.878019928	6.90E-06	5.90E-05
AC023794.1	lncRNA	3.142281747	6.94E-06	5.93E-05
AC079465.1	lncRNA	-2.524129073	7.06E-06	6.02E-05
FOXD3-AS1	lncRNA	5.447261785	7.79E-06	6.61E-05
AC009133.1	lncRNA	2.031897938	8.13E-06	6.89E-05
AP000757.1	lncRNA	3.591297242	8.16E-06	6.90E-05
AC026369.3	lncRNA	-2.381629041	8.39E-06	7.09E-05
FEZF1-AS1	lncRNA	5.568622046	8.41E-06	7.09E-05
LINC01730	lncRNA	2.9519638	8.43E-06	7.10E-05
AC093690.1	lncRNA	2.354285323	8.48E-06	7.13E-05
AL132765.2	lncRNA	3.970815125	8.50E-06	7.13E-05
AC015712.1	lncRNA	3.57636317	8.61E-06	7.21E-05
AC017083.1	lncRNA	2.355623298	8.81E-06	7.37E-05
LINC00239	lncRNA	2.61956621	9.03E-06	7.54E-05
AP000757.2	lncRNA	4.626448405	9.14E-06	7.61E-05
AC037198.2	lncRNA	-2.183793069	9.19E-06	7.64E-05
LINC00592	lncRNA	4.635397466	9.41E-06	7.79E-05
AL354811.1	lncRNA	-2.294991937	9.71E-06	8.01E-05
AC006557.1	lncRNA	3.225364569	9.72E-06	8.02E-05
LINC01762	lncRNA	3.459977735	9.79E-06	8.06E-05
AC079315.1	lncRNA	2.381175226	9.83E-06	8.08E-05
GACAT2	lncRNA	7.019663736	9.92E-06	8.15E-05
LINC02182	lncRNA	5.247791789	1.02E-05	8.33E-05
LINC02560	lncRNA	6.020272699	1.02E-05	8.33E-05
AP000866.5	lncRNA	2.948829238	1.02E-05	8.33E-05
AL035461.2	lncRNA	2.183103897	1.04E-05	8.48E-05
AC048344.4	lncRNA	3.664249516	1.10E-05	8.93E-05
SNHG28	lncRNA	2.331506285	1.10E-05	8.93E-05
AC016924.1	lncRNA	-2.242331352	1.11E-05	9.00E-05
DPP10-AS1	lncRNA	3.744272834	1.15E-05	9.30E-05
BOLA3-AS1	lncRNA	2.119954696	1.16E-05	9.38E-05
VAC14-AS1	lncRNA	2.971249813	1.17E-05	9.43E-05
ATP2A1-AS1	lncRNA	2.348402559	1.17E-05	9.45E-05
DDN-AS1	lncRNA	2.693188856	1.20E-05	9.63E-05
LINC01619	lncRNA	2.109456245	1.22E-05	9.82E-05

AC092111.2	lncRNA	3.320592146	1.24E-05	9.93E-05
AC016737.1	lncRNA	2.062874851	1.27E-05	0.000102
AC010201.1	lncRNA	2.268246454	1.32E-05	0.000105
AC139887.4	lncRNA	2.408167948	1.36E-05	0.000108
AC015921.1	lncRNA	5.15771181	1.44E-05	0.000114
AC060766.6	lncRNA	4.311704847	1.45E-05	0.000114
PAQR9-AS1	lncRNA	-2.677269029	1.45E-05	0.000114
AC013731.1	lncRNA	2.020812763	1.46E-05	0.000115
HAGLROS	lncRNA	3.516838333	1.47E-05	0.000115
OSTN-AS1	lncRNA	-3.244632744	1.48E-05	0.000117
C9orf163	lncRNA	2.241386745	1.51E-05	0.000119
LINC02154	lncRNA	6.297150698	1.59E-05	0.000125
AC008691.1	lncRNA	5.454870772	1.60E-05	0.000125
AC007319.1	lncRNA	-2.083941049	1.64E-05	0.000127
AC011921.1	lncRNA	2.338463563	1.66E-05	0.000129
AC073842.1	lncRNA	-2.760238981	1.66E-05	0.000129
AC110597.1	lncRNA	2.847093488	1.69E-05	0.000131
THRB-IT1	lncRNA	-2.327689512	1.69E-05	0.000131
AC015908.2	lncRNA	-2.521025325	1.69E-05	0.000131
PLAC4	lncRNA	8.773622275	1.71E-05	0.000132
AC008011.2	lncRNA	5.629741597	1.76E-05	0.000136
AC011773.4	lncRNA	3.558487951	1.77E-05	0.000136
AC022509.3	lncRNA	3.047922482	1.77E-05	0.000136
AL391845.2	lncRNA	2.972646706	1.79E-05	0.000138
AC025154.2	lncRNA	7.906584991	1.83E-05	0.00014
AL117339.4	lncRNA	2.085525536	1.84E-05	0.000141
AC093151.2	lncRNA	2.815458953	1.85E-05	0.000142
SAPCD1-AS1	lncRNA	2.493779248	1.86E-05	0.000142
APCDD1L-AS1	lncRNA	7.795313341	1.88E-05	0.000144
AC126118.1	lncRNA	2.405652686	1.92E-05	0.000146
AC245407.2	lncRNA	3.062757608	1.92E-05	0.000146
AC027117.2	lncRNA	-2.490208979	1.97E-05	0.000149
AL121929.2	lncRNA	3.808035829	2.01E-05	0.000153
EVX1-AS	lncRNA	6.336158971	2.02E-05	0.000153
SPATA3-AS1	lncRNA	3.545537606	2.02E-05	0.000153
AC097478.1	lncRNA	6.260581266	2.05E-05	0.000155
AC006449.2	lncRNA	2.355356446	2.09E-05	0.000158
LINC02038	lncRNA	3.303772342	2.10E-05	0.000158
LINC01182	lncRNA	-2.255818983	2.11E-05	0.000159
AC097358.2	lncRNA	5.130894629	2.11E-05	0.000159
PTPRG-AS1	lncRNA	2.420333302	2.12E-05	0.000159
AL031316.1	lncRNA	-2.055379134	2.17E-05	0.000163
AP001178.3	lncRNA	2.780183399	2.18E-05	0.000163
AL121992.1	lncRNA	3.545254877	2.18E-05	0.000163
AC114488.2	lncRNA	2.514007215	2.20E-05	0.000164
AP003555.3	lncRNA	-2.174190965	2.21E-05	0.000165
AC021086.1	lncRNA	-2.365207596	2.21E-05	0.000165

AC026992.1	lncRNA	3.75886791	2.28E-05	0.00017
LINC00315	lncRNA	2.190834859	2.29E-05	0.000171
AC139100.2	lncRNA	2.285326565	2.31E-05	0.000172
AP001922.6	lncRNA	2.109789667	2.31E-05	0.000172
AL359881.1	lncRNA	3.25585079	2.38E-05	0.000176
ROR1-AS1	lncRNA	9.457654477	2.39E-05	0.000177
AC011313.1	lncRNA	4.84184791	2.40E-05	0.000177
UNC5B-AS1	lncRNA	4.809357856	2.45E-05	0.000181
AC084082.1	lncRNA	-2.268234055	2.51E-05	0.000186
AL392089.1	lncRNA	-2.382939588	2.59E-05	0.00019
AC131159.2	lncRNA	2.194393017	2.60E-05	0.000191
AC023355.1	lncRNA	2.001897898	2.63E-05	0.000193
AP001347.1	lncRNA	2.610620992	2.66E-05	0.000194
AC092611.2	lncRNA	2.962803852	2.72E-05	0.000198
AC060766.7	lncRNA	2.022077677	2.72E-05	0.000198
ABALON	lncRNA	2.198572585	2.73E-05	0.000198
AC004080.1	lncRNA	6.387148237	2.78E-05	0.000202
LINC02441	lncRNA	7.513580761	2.79E-05	0.000202
CR559946.2	lncRNA	2.5696996	2.81E-05	0.000203
HEXA-AS1	lncRNA	2.296516035	2.84E-05	0.000205
AL118511.1	lncRNA	2.505321167	2.94E-05	0.000212
LINC00707	lncRNA	6.097509372	2.94E-05	0.000212
AP003390.1	lncRNA	2.898811765	2.94E-05	0.000212
AC021151.1	lncRNA	7.199386953	3.03E-05	0.000218
AC091544.4	lncRNA	2.716156791	3.04E-05	0.000218
AC084033.3	lncRNA	2.324532199	3.07E-05	0.00022
KRTAP5-AS1	lncRNA	2.960312941	3.09E-05	0.000221
AL136084.2	lncRNA	5.173615404	3.12E-05	0.000223
AC026740.1	lncRNA	3.032863386	3.15E-05	0.000225
AC108463.3	lncRNA	3.441557003	3.19E-05	0.000228
AC087752.4	lncRNA	2.059561079	3.23E-05	0.00023
AC087645.2	lncRNA	2.815693534	3.27E-05	0.000233
AC011611.3	lncRNA	-2.26217463	3.34E-05	0.000237
AC096996.2	lncRNA	-3.287731181	3.35E-05	0.000238
LINC02158	lncRNA	-2.178937389	3.40E-05	0.000241
LINC01589	lncRNA	2.779688295	3.45E-05	0.000244
AL137145.2	lncRNA	2.491096125	3.46E-05	0.000245
KRT7-AS	lncRNA	2.466438473	3.50E-05	0.000247
AC104211.2	lncRNA	-2.06588447	3.54E-05	0.000249
AC078942.1	lncRNA	-2.887566745	3.56E-05	0.00025
AC020915.2	lncRNA	2.182075598	3.60E-05	0.000253
AC009041.2	lncRNA	3.612742014	3.62E-05	0.000254
AC110619.1	lncRNA	3.314212545	3.65E-05	0.000255
AP003119.2	lncRNA	-2.112415236	3.66E-05	0.000256
AL353801.3	lncRNA	2.301341318	3.68E-05	0.000257
AC016735.1	lncRNA	3.241870963	3.69E-05	0.000258
AC007639.1	lncRNA	3.738811129	3.74E-05	0.00026

AL391832.1	lncRNA	-2.271879512	3.77E-05	0.000262
AC129492.1	lncRNA	3.360763797	3.78E-05	0.000263
LINC01234	lncRNA	6.731260983	3.97E-05	0.000275
HAGLR	lncRNA	4.579366886	3.98E-05	0.000275
AC079949.1	lncRNA	-2.039910809	4.00E-05	0.000276
LINC01572	lncRNA	2.576562502	4.00E-05	0.000276
LINC00313	lncRNA	-2.271008135	4.00E-05	0.000276
AC127164.1	lncRNA	2.836712487	4.05E-05	0.000279
LINC00885	lncRNA	-2.285701113	4.11E-05	0.000283
AP001029.2	lncRNA	2.45963609	4.12E-05	0.000283
AC233280.1	lncRNA	2.917279452	4.13E-05	0.000283
AP000424.2	lncRNA	3.459954858	4.13E-05	0.000283
AC245884.1	lncRNA	4.305806072	4.30E-05	0.000293
LINC01687	lncRNA	-3.651809747	4.31E-05	0.000293
LINC02188	lncRNA	6.004152311	4.32E-05	0.000293
LINC01659	lncRNA	4.148806306	4.34E-05	0.000294
AP002957.1	lncRNA	7.06651078	4.36E-05	0.000296
AC023115.1	lncRNA	4.755235552	4.37E-05	0.000296
AC126768.2	lncRNA	6.856088907	4.37E-05	0.000296
BX322234.2	lncRNA	5.328782697	4.39E-05	0.000296
AC105411.1	lncRNA	4.926208005	4.42E-05	0.000298
AC010524.1	lncRNA	3.435977685	4.51E-05	0.000303
AC012499.1	lncRNA	-2.792012586	4.55E-05	0.000306
LINC01214	lncRNA	-3.006156436	4.57E-05	0.000307
DLG3-AS1	lncRNA	4.473023836	4.64E-05	0.000311
AC008637.1	lncRNA	3.27544618	4.92E-05	0.000328
AL161669.1	lncRNA	-2.147566504	4.92E-05	0.000328
AL031717.1	lncRNA	2.421080838	4.95E-05	0.00033
AC243772.2	lncRNA	3.130999614	5.01E-05	0.000333
LINC01436	lncRNA	5.432325618	5.22E-05	0.000346
AC004466.3	lncRNA	2.898132753	5.23E-05	0.000346
LINC01094	lncRNA	2.144153297	5.32E-05	0.000352
AC011747.1	lncRNA	-2.697270686	5.32E-05	0.000352
AC087482.1	lncRNA	-2.533391189	5.37E-05	0.000354
AP001628.1	lncRNA	2.695769609	5.47E-05	0.00036
DRAIC	lncRNA	-2.064759848	5.49E-05	0.000361
AL121895.1	lncRNA	2.636060784	5.53E-05	0.000363
AC006960.3	lncRNA	3.430093049	5.60E-05	0.000367
FZD10-AS1	lncRNA	3.593575642	5.62E-05	0.000369
AL356234.2	lncRNA	4.030088231	5.63E-05	0.000369
AL691482.3	lncRNA	2.732225873	5.64E-05	0.000369
AC110048.2	lncRNA	2.295883628	5.65E-05	0.00037
NCOA7-AS1	lncRNA	-3.193259955	5.71E-05	0.000373
SFTA1P	lncRNA	4.855367167	5.72E-05	0.000373
AP003680.1	lncRNA	3.377517783	5.74E-05	0.000374
AC005821.1	lncRNA	3.884912628	5.84E-05	0.00038
AC134682.1	lncRNA	2.272567306	5.94E-05	0.000386

FOXCUT	lncRNA	5.385259321	6.08E-05	0.000394
LINC01117	lncRNA	6.426646256	6.17E-05	0.000399
LINC00443	lncRNA	8.641260209	6.22E-05	0.000402
LINC02331	lncRNA	3.256558988	6.25E-05	0.000404
AC010999.1	lncRNA	2.750313125	6.26E-05	0.000404
AC036108.4	lncRNA	3.323909981	6.41E-05	0.000414
FAM66B	lncRNA	2.45097124	6.44E-05	0.000415
AC245100.6	lncRNA	4.654648248	6.51E-05	0.000419
ELOVL2-AS1	lncRNA	-2.330754384	6.55E-05	0.000421
AC245884.9	lncRNA	5.108573967	6.58E-05	0.000423
LINC02405	lncRNA	-2.105041391	6.63E-05	0.000426
DNM1P35	lncRNA	2.250546851	6.67E-05	0.000427
AC009275.1	lncRNA	2.615137172	6.70E-05	0.000429
AF131215.4	lncRNA	3.826203556	6.76E-05	0.000431
AC016738.1	lncRNA	2.166715173	6.78E-05	0.000432
AC004466.2	lncRNA	3.172056058	6.81E-05	0.000434
ISPD-AS1	lncRNA	-2.053781848	6.88E-05	0.000438
AL353693.1	lncRNA	-3.151941367	6.93E-05	0.000441
AC011352.3	lncRNA	5.943450585	6.97E-05	0.000442
AC021491.2	lncRNA	3.603755088	7.04E-05	0.000447
AP005233.2	lncRNA	5.462955242	7.13E-05	0.000452
AC006273.1	lncRNA	3.37825457	7.22E-05	0.000457
AL731567.1	lncRNA	2.787505705	7.27E-05	0.00046
AL137847.1	lncRNA	3.058065562	7.55E-05	0.000475
AL138760.1	lncRNA	5.170896732	7.55E-05	0.000475
AL161772.1	lncRNA	2.654065978	7.56E-05	0.000475
AC037487.1	lncRNA	2.861380322	7.57E-05	0.000475
AL031123.1	lncRNA	4.652907396	7.68E-05	0.000481
AC096564.2	lncRNA	-2.146505942	7.71E-05	0.000482
AL021391.1	lncRNA	4.067786365	7.89E-05	0.000493
LINC01778	lncRNA	-2.612004486	7.90E-05	0.000493
HOXC-AS2	lncRNA	4.224524682	7.92E-05	0.000493
LINC02321	lncRNA	-2.152214516	8.12E-05	0.000505
AL162412.1	lncRNA	-2.170741788	8.12E-05	0.000505
AL353593.1	lncRNA	2.217244598	8.16E-05	0.000506
GATA2-AS1	lncRNA	2.803854718	8.18E-05	0.000507
AC239800.2	lncRNA	-2.921975835	8.19E-05	0.000507
AC007750.1	lncRNA	2.791386993	8.26E-05	0.000511
AC034213.1	lncRNA	5.80008037	8.51E-05	0.000524
AC245595.1	lncRNA	2.481270157	8.57E-05	0.000527
AC125616.1	lncRNA	7.886790928	8.70E-05	0.000533
AC124798.1	lncRNA	2.883828117	8.82E-05	0.00054
LINC00942	lncRNA	6.003746831	8.86E-05	0.000541
AC005256.1	lncRNA	5.710344448	8.97E-05	0.000546
FRMD6-AS1	lncRNA	2.390057718	9.00E-05	0.000547
AC116351.1	lncRNA	2.797715915	9.29E-05	0.000564
GRM5-AS1	lncRNA	5.536768295	9.33E-05	0.000566

AC096577.1	lncRNA	-3.158714094	9.46E-05	0.000574
AC004264.1	lncRNA	3.385931072	9.49E-05	0.000574
AL589765.6	lncRNA	4.33680632	9.49E-05	0.000574
AP000696.1	lncRNA	6.901569603	9.55E-05	0.000577
AC099521.1	lncRNA	2.884521227	9.64E-05	0.000582
AC010978.1	lncRNA	3.193888617	9.67E-05	0.000583
TM4SF1-AS1	lncRNA	2.455118155	9.72E-05	0.000585
UBE2Q1-AS1	lncRNA	2.406435721	9.85E-05	0.000592
AC027688.2	lncRNA	-3.448088373	0.0001	0.000602
AC090921.1	lncRNA	4.248006949	0.000102	0.000611
AC084346.2	lncRNA	4.801958197	0.000102	0.000614
AC011676.1	lncRNA	2.913412603	0.000103	0.000614
AC092611.1	lncRNA	2.151427286	0.000104	0.000618
AL442067.1	lncRNA	4.495360949	0.000104	0.000621
AC090260.1	lncRNA	2.839018239	0.000104	0.000623
AL445070.1	lncRNA	6.867373567	0.000105	0.000625
C10orf91	lncRNA	2.974456678	0.000105	0.000627
CLRN1-AS1	lncRNA	-3.039992782	0.000105	0.000627
AC116337.3	lncRNA	3.267237439	0.000106	0.000628
AL590426.2	lncRNA	-3.179885897	0.000107	0.000633
AL451069.1	lncRNA	5.119807056	0.000107	0.000633
AL161431.1	lncRNA	10.37771214	0.000107	0.000634
AC005841.1	lncRNA	4.287091056	0.000107	0.000635
NEBL-AS1	lncRNA	3.205311343	0.000108	0.000638
AC022915.2	lncRNA	-2.108559716	0.000109	0.000641
LINC02551	lncRNA	4.053118283	0.00011	0.000652
AC098614.4	lncRNA	2.769314399	0.000111	0.000653
DPYD-AS1	lncRNA	-2.013999921	0.000112	0.000656
AC103702.2	lncRNA	7.239352074	0.000113	0.000662
AP003031.1	lncRNA	-2.357407018	0.000113	0.000665
AC007221.1	lncRNA	-2.334595441	0.000114	0.000668
LINC02568	lncRNA	5.372223335	0.000114	0.00067
AC127024.4	lncRNA	2.077874609	0.000115	0.000672
AL445228.2	lncRNA	2.72180199	0.000116	0.000679
AL160290.2	lncRNA	3.620696735	0.000118	0.000686
AC010175.1	lncRNA	-2.305288976	0.000121	0.000707
AP000553.2	lncRNA	3.077184752	0.000122	0.00071
DLX6-AS1	lncRNA	4.735047329	0.000124	0.000722
AC022101.1	lncRNA	-2.825554942	0.000129	0.000748
U91328.3	lncRNA	2.323015683	0.000132	0.000761
LINC00930	lncRNA	3.863024789	0.000133	0.000766
AC011352.1	lncRNA	5.084676843	0.000133	0.000768
LINC01913	lncRNA	6.14620634	0.000134	0.00077
AC107464.3	lncRNA	3.200560316	0.000136	0.000781
AL121748.1	lncRNA	4.032445334	0.000136	0.000784
AL133383.1	lncRNA	3.636185715	0.000138	0.000789
AL021154.1	lncRNA	2.604723724	0.000138	0.000789

AC005062.1	lncRNA	2.485757539	0.00014	0.000798
AC017067.1	lncRNA	-2.430053388	0.00014	0.000802
AC073352.1	lncRNA	2.735549659	0.000142	0.000809
LINC02332	lncRNA	4.601189081	0.000143	0.000811
FP325335.1	lncRNA	4.981772218	0.000143	0.000811
LINC01585	lncRNA	3.458780176	0.000143	0.000811
AC090409.1	lncRNA	2.591179583	0.000145	0.000823
AC040174.1	lncRNA	6.092852803	0.000145	0.000824
AC004596.1	lncRNA	2.100964579	0.000147	0.00083
AL355922.2	lncRNA	2.018989726	0.000148	0.000837
AL355997.1	lncRNA	3.119126655	0.000149	0.000841
AC093458.1	lncRNA	4.82566253	0.00015	0.000847
AL137026.2	lncRNA	5.195133644	0.000151	0.000848
AC073111.1	lncRNA	3.065003906	0.000151	0.000851
AL122125.1	lncRNA	2.342074028	0.000152	0.000857
LINC01535	lncRNA	3.103301184	0.000158	0.000885
AC104695.2	lncRNA	2.733280568	0.00016	0.000894
LINC01583	lncRNA	7.145603147	0.000161	0.000898
AL118505.1	lncRNA	3.384671256	0.000162	0.000902
LINC01711	lncRNA	5.350663588	0.000163	0.000907
TGFB2-AS1	lncRNA	3.287975094	0.000164	0.000913
LINC01152	lncRNA	2.800704577	0.000166	0.000923
AP000919.2	lncRNA	-2.276800684	0.000171	0.000946
AC008667.3	lncRNA	4.820154673	0.000172	0.00095
AC004466.1	lncRNA	2.040532358	0.000175	0.000966
AL359397.1	lncRNA	4.532480403	0.000178	0.00098
AL512413.1	lncRNA	2.490883403	0.00018	0.000991
AC073592.1	lncRNA	3.080029578	0.00018	0.000992
AC110285.6	lncRNA	2.044306825	0.000182	0.001002
ZNF528-AS1	lncRNA	2.217418276	0.000184	0.001007
LINC02115	lncRNA	4.653287163	0.000184	0.001008
AC107959.2	lncRNA	4.493688016	0.000185	0.001015
TRPC7-AS1	lncRNA	5.604094548	0.000186	0.00102
LINC00316	lncRNA	3.555714524	0.000186	0.00102
AC025580.1	lncRNA	4.183923547	0.000188	0.001026
AL139220.2	lncRNA	2.103783866	0.000188	0.001026
AL353593.3	lncRNA	3.302033807	0.000188	0.001027
AC244517.7	lncRNA	3.717649935	0.00019	0.001036
AC007953.1	lncRNA	-2.255833151	0.000193	0.001049
AC093620.1	lncRNA	2.015537082	0.000195	0.001062
SSTR5-AS1	lncRNA	5.021946969	0.000197	0.001071
SLC6A1-AS1	lncRNA	-2.085729831	0.000198	0.001072
AC139749.1	lncRNA	2.654783562	0.000201	0.001087
AC092336.1	lncRNA	9.10627381	0.000202	0.001092
AP002360.2	lncRNA	3.697345649	0.000204	0.001101
LINC02048	lncRNA	-2.098590101	0.000207	0.001114
AL591806.1	lncRNA	4.469638023	0.000207	0.001118

AC022390.1	lncRNA	3.587074727	0.00021	0.001131
AL121760.1	lncRNA	2.510147741	0.000211	0.001137
AC090541.1	lncRNA	4.426346059	0.000212	0.001138
AL512328.1	lncRNA	3.467383477	0.000212	0.001138
Z82246.1	lncRNA	-3.176735095	0.000212	0.001138
AC097059.2	lncRNA	3.011331058	0.000213	0.001143
AP000424.1	lncRNA	2.911754715	0.000214	0.001145
AL162431.1	lncRNA	4.310575969	0.000215	0.001152
AC009542.1	lncRNA	2.595462349	0.000216	0.001152
AC106772.1	lncRNA	5.872725367	0.000216	0.001155
AL035706.1	lncRNA	-2.483777021	0.00022	0.001172
AC106052.1	lncRNA	2.965829776	0.00022	0.001172
LINC00887	lncRNA	2.787871518	0.000222	0.001179
AP000808.2	lncRNA	2.202484702	0.000226	0.0012
C12orf80	lncRNA	4.960746959	0.000226	0.001203
AC055822.1	lncRNA	2.129940655	0.000227	0.001205
SYNPR-AS1	lncRNA	5.039822662	0.000227	0.001206
AF233439.1	lncRNA	2.842876564	0.000228	0.001209
AC027117.1	lncRNA	2.649847311	0.000232	0.001226
NEXN-AS1	lncRNA	2.616227688	0.000232	0.001226
AP002989.1	lncRNA	3.396281741	0.000236	0.001247
Z97653.1	lncRNA	2.699983322	0.000236	0.001249
NPSR1-AS1	lncRNA	4.399805462	0.000237	0.001252
AC110285.1	lncRNA	2.02430582	0.000237	0.001252
DNAH17-AS1	lncRNA	3.273450138	0.000239	0.001258
AP002812.3	lncRNA	2.634308311	0.000245	0.001288
AC007207.2	lncRNA	-2.299801722	0.000246	0.001291
Z84723.1	lncRNA	5.065569584	0.000247	0.001299
AC104695.3	lncRNA	2.114532978	0.000249	0.001308
AC092296.3	lncRNA	-2.296814523	0.000251	0.001315
AL607028.1	lncRNA	2.254139792	0.000253	0.001327
AC106869.1	lncRNA	2.018676395	0.000254	0.001331
LINC01819	lncRNA	5.945341594	0.000257	0.001342
KCNK15-AS1	lncRNA	3.199257747	0.00026	0.001359
LINC01929	lncRNA	3.78847876	0.000261	0.00136
AL080312.2	lncRNA	5.799299957	0.000273	0.001414
AC020928.1	lncRNA	2.162184781	0.000274	0.001418
AC023449.2	lncRNA	2.232158605	0.000277	0.001433
PYCARD-AS1	lncRNA	2.441392325	0.000281	0.001451
AC005699.1	lncRNA	4.323809134	0.000285	0.00147
HIF1A-AS1	lncRNA	3.42476604	0.000287	0.001482
AL596223.1	lncRNA	3.381182649	0.000293	0.001508
AC103564.1	lncRNA	6.518050542	0.000296	0.00152
AC127496.1	lncRNA	2.354368624	0.000296	0.001522
PAX8-AS1	lncRNA	2.962460671	0.000298	0.001529
AC099791.2	lncRNA	2.784754534	0.0003	0.001538
LINC02212	lncRNA	7.943738245	0.000315	0.001607

AC108463.2	lncRNA	2.073282344	0.000318	0.001618
AL354984.1	lncRNA	4.759710809	0.000319	0.001624
TMEM72-AS1	lncRNA	3.285595146	0.000324	0.001647
LINC01748	lncRNA	5.902851037	0.000325	0.00165
LINC01612	lncRNA	-2.638989575	0.000329	0.001673
AC055855.2	lncRNA	3.248217176	0.00033	0.001673
SH3PXD2A-AS1	lncRNA	4.014005346	0.000333	0.001691
LINC01649	lncRNA	3.541459825	0.000336	0.001703
AC109454.3	lncRNA	-2.920287158	0.000337	0.001706
AC010536.3	lncRNA	3.431461103	0.00034	0.001718
AL160286.2	lncRNA	3.624689427	0.00034	0.001719
LINC01671	lncRNA	2.387979779	0.000342	0.001725
NRG1-IT1	lncRNA	5.951237965	0.000344	0.001737
LINC00184	lncRNA	3.502313942	0.000345	0.001741
AC027312.1	lncRNA	3.809191166	0.000348	0.001751
C1QTNF1-AS1	lncRNA	3.44274036	0.000358	0.001799
AC009549.1	lncRNA	3.003022543	0.000362	0.001816
LINC01269	lncRNA	5.267010554	0.000362	0.001816
LINC01480	lncRNA	2.682877918	0.000367	0.001834
AL139241.1	lncRNA	3.189106678	0.000368	0.001841
ZFHX2-AS1	lncRNA	3.38617903	0.000369	0.001845
AL163953.1	lncRNA	2.806758702	0.00037	0.001848
AC005696.4	lncRNA	4.688406702	0.00037	0.001848
AL390115.1	lncRNA	4.273067685	0.000371	0.001849
FAM215B	lncRNA	2.80352218	0.000377	0.001878
AP005271.1	lncRNA	2.967546869	0.000379	0.001883
AL356356.1	lncRNA	2.061514555	0.000383	0.001903
LINC01595	lncRNA	-4.101854106	0.000394	0.001947
AC245100.7	lncRNA	2.26411119	0.000394	0.001947
AC239809.3	lncRNA	2.121310552	0.000395	0.00195
AC133644.2	lncRNA	3.299134995	0.000398	0.001965
LINC02421	lncRNA	2.508122332	0.000409	0.002008
AC007182.1	lncRNA	-3.103946485	0.00041	0.002011
AC110285.3	lncRNA	4.747694431	0.000411	0.002016
AC245041.2	lncRNA	2.643058901	0.000413	0.002026
AL049836.1	lncRNA	4.15059239	0.000417	0.002037
AL365226.2	lncRNA	7.732131467	0.000419	0.002046
AC007272.1	lncRNA	2.576532641	0.000424	0.002068
FAM66D	lncRNA	2.428856835	0.000427	0.002078
LINC02345	lncRNA	3.296180432	0.000428	0.002084
AL359091.5	lncRNA	2.487268893	0.000434	0.00211
AC015961.2	lncRNA	2.15355122	0.000435	0.002115
AC106037.1	lncRNA	2.143316376	0.000436	0.002116
AL590666.1	lncRNA	2.801803224	0.000436	0.002116
AP001271.1	lncRNA	2.84610672	0.00044	0.002128
AC087623.1	lncRNA	3.111249459	0.000441	0.002132
AP003721.1	lncRNA	4.900999347	0.000446	0.002151

AC008667.1	lncRNA	2.940951119	0.00045	0.002167
AL161725.2	lncRNA	2.864595439	0.000454	0.002184
C2orf48	lncRNA	2.649266886	0.000466	0.002239
AL158151.4	lncRNA	2.718260794	0.000466	0.002239
LINC00322	lncRNA	3.445652174	0.000469	0.002247
LINC00704	lncRNA	3.635308189	0.000469	0.002247
AC079834.2	lncRNA	2.059712796	0.000475	0.002273
AC109361.2	lncRNA	2.817582175	0.000476	0.002276
AC092171.1	lncRNA	2.126825172	0.000479	0.002288
STAU2-AS1	lncRNA	2.980063849	0.000481	0.002298
LINC01658	lncRNA	4.629186284	0.000482	0.002303
LINC02519	lncRNA	2.743671467	0.000484	0.00231
LINC02080	lncRNA	3.287282306	0.000493	0.002349
CYP4F26P	lncRNA	3.191183433	0.000501	0.00238
AL133467.1	lncRNA	-2.025942522	0.000501	0.00238
SLC7A11-AS1	lncRNA	5.458708612	0.000509	0.002416
AC090241.3	lncRNA	2.629940451	0.000515	0.00244
AL449403.2	lncRNA	3.165422967	0.000517	0.002446
AC110491.1	lncRNA	4.608617214	0.000518	0.002449
AC098936.1	lncRNA	5.060901234	0.000524	0.002474
AL137244.1	lncRNA	2.096719688	0.000524	0.002474
AC078850.1	lncRNA	3.421827048	0.000525	0.002477
AL137798.1	lncRNA	-2.735207875	0.000528	0.002491
AC007285.1	lncRNA	2.781114281	0.000529	0.002493
AC092118.1	lncRNA	2.007380986	0.000538	0.002531
AC079779.2	lncRNA	3.204334484	0.00054	0.002537
AC000403.1	lncRNA	2.063800529	0.000547	0.002566
LINC02137	lncRNA	5.478120104	0.000547	0.002566
LINC02014	lncRNA	4.348236763	0.000549	0.002569
LINC00390	lncRNA	-2.585059116	0.000549	0.002572
AC022424.1	lncRNA	4.731889222	0.000552	0.002579
LINC02081	lncRNA	3.905377583	0.000552	0.002579
SLC9A3-AS1	lncRNA	2.172906121	0.000555	0.002589
LINC00348	lncRNA	-2.863070927	0.000562	0.00262
AC115522.1	lncRNA	2.312300517	0.000565	0.002629
AC133794.1	lncRNA	4.074807913	0.000566	0.002634
AC106712.1	lncRNA	-2.217913653	0.000569	0.002644
SOX21-AS1	lncRNA	5.365485285	0.000577	0.002677
AL391361.2	lncRNA	-2.208313407	0.000578	0.00268
HOXC-AS1	lncRNA	4.940598734	0.000579	0.002683
AP001207.3	lncRNA	4.304716696	0.000585	0.002699
LINP1	lncRNA	4.783142852	0.000586	0.002703
Z97200.1	lncRNA	4.054677476	0.000586	0.002703
AC008750.3	lncRNA	2.729352931	0.000587	0.002703
AP003063.1	lncRNA	4.127432116	0.000596	0.002742
LINC02119	lncRNA	6.527120332	0.000598	0.002745
AC009955.1	lncRNA	4.904029605	0.0006	0.002752

MIR3150BHG	lncRNA	3.607594718	0.000601	0.002754
AL359881.3	lncRNA	4.074831268	0.000601	0.002754
HDAC11-AS1	lncRNA	3.85225158	0.000605	0.002765
AL359881.2	lncRNA	2.828637917	0.000609	0.002784
KIF26B-AS1	lncRNA	5.192378522	0.000615	0.002806
AC244517.1	lncRNA	3.255510523	0.000616	0.002809
AC124312.3	lncRNA	2.530627273	0.000624	0.002841
AC120036.4	lncRNA	2.582731339	0.000627	0.002847
AC000067.1	lncRNA	3.976491246	0.000631	0.002866
AL031663.3	lncRNA	4.674493366	0.000632	0.002869
PIK3CD-AS2	lncRNA	2.051044872	0.000633	0.00287
AC025166.1	lncRNA	4.117066624	0.000638	0.002886
MYOSLID	lncRNA	3.919194734	0.000641	0.002898
FAM87A	lncRNA	3.484758997	0.000643	0.002907
AP001063.1	lncRNA	-2.519488463	0.000648	0.002924
AC096637.2	lncRNA	2.704551375	0.000653	0.002946
AC068196.1	lncRNA	3.179878424	0.000655	0.002952
AC144833.1	lncRNA	7.098322685	0.000661	0.002977
AC007497.1	lncRNA	2.54312681	0.000669	0.00301
AP004609.1	lncRNA	4.986512828	0.000671	0.003017
LINC02043	lncRNA	3.424635189	0.000678	0.003041
AC138305.1	lncRNA	4.420153667	0.000682	0.003056
AC087761.1	lncRNA	-2.035333406	0.000687	0.003077
LINC01411	lncRNA	3.721633012	0.0007	0.003125
AL365356.5	lncRNA	4.864795738	0.000701	0.003127
LHFPL3-AS1	lncRNA	5.117117775	0.000713	0.003175
AL355472.3	lncRNA	3.257382736	0.000714	0.003181
AC107464.1	lncRNA	4.311437015	0.000717	0.003192
AC073323.1	lncRNA	6.197509733	0.00072	0.0032
AP000943.1	lncRNA	3.720421958	0.000724	0.003216
AC004988.1	lncRNA	2.543341235	0.000724	0.003216
LINC02086	lncRNA	5.267294253	0.000731	0.003239
LINC01136	lncRNA	2.46616972	0.000735	0.003254
AC135050.4	lncRNA	2.480351572	0.000735	0.003254
AL353801.2	lncRNA	3.304879551	0.00075	0.003313
AC073283.1	lncRNA	4.060098684	0.000751	0.003313
AC023509.1	lncRNA	2.154028826	0.000752	0.003313
LINC02261	lncRNA	5.793539237	0.000752	0.003313
LINC00565	lncRNA	2.483012733	0.000754	0.003317
AC093462.1	lncRNA	2.634074167	0.00076	0.003342
FIRRE	lncRNA	2.837893168	0.000761	0.003346
HOXB-AS4	lncRNA	7.083762638	0.000766	0.003361
AC012531.1	lncRNA	5.961872944	0.000771	0.003379
LINC01159	lncRNA	4.487193823	0.000772	0.003382
PCAT14	lncRNA	4.10438111	0.000774	0.00339
AL353801.1	lncRNA	3.734753426	0.000782	0.003418
LINC01914	lncRNA	3.230480111	0.000783	0.00342

AC012358.1	lncRNA	2.296515634	0.000797	0.003471
AL391056.1	lncRNA	3.992552417	0.000801	0.003479
AC067817.2	lncRNA	2.003874353	0.000805	0.003496
AC123595.1	lncRNA	-2.075366604	0.000813	0.003524
AL139339.2	lncRNA	3.937795431	0.000814	0.003525
AC099792.1	lncRNA	4.515765872	0.000821	0.003557
AL109804.1	lncRNA	2.293570766	0.000831	0.003591
AC015660.2	lncRNA	4.110996048	0.000839	0.003614
AC245041.1	lncRNA	2.448248077	0.00084	0.003614
AC013553.3	lncRNA	2.284545139	0.000852	0.003653
AC004906.1	lncRNA	4.186675858	0.000859	0.003683
AL513211.1	lncRNA	2.313148068	0.000884	0.003778
PLCE1-AS1	lncRNA	4.019186911	0.000885	0.00378
RNF144A-AS1	lncRNA	2.706466721	0.0009	0.003832
AC034236.3	lncRNA	2.928799627	0.000903	0.003843
SZT2-AS1	lncRNA	3.075759476	0.00091	0.003871
AL365259.1	lncRNA	-2.366042505	0.000913	0.003879
AC004066.2	lncRNA	3.831181725	0.000914	0.003882
AC112721.1	lncRNA	6.461854687	0.000917	0.003893
AC092667.1	lncRNA	2.8912329	0.00093	0.003942
AC009118.2	lncRNA	2.220594268	0.000933	0.003954
AC011479.2	lncRNA	2.445681292	0.000944	0.003991
AC006270.1	lncRNA	4.300360296	0.000951	0.004015
MYHAS	lncRNA	4.31383663	0.000966	0.004067
AC093833.1	lncRNA	3.077585341	0.000966	0.004067
LINC00519	lncRNA	3.819290915	0.000966	0.004067
AL133523.1	lncRNA	2.336257993	0.000983	0.004125
AB015752.1	lncRNA	-2.208821361	0.000991	0.004156
HOTAIR	lncRNA	7.300588786	0.001004	0.004198
AC009269.4	lncRNA	3.306526274	0.001008	0.004213
AC068870.1	lncRNA	2.244025071	0.00101	0.004219
AC113383.1	lncRNA	2.630398809	0.001015	0.004239
AC015712.6	lncRNA	3.385806511	0.001017	0.004242
AC011405.1	lncRNA	2.112162978	0.001032	0.004302
LINC01315	lncRNA	2.168327253	0.001053	0.004377
LINC01169	lncRNA	-2.108638159	0.001055	0.004382
LINC00922	lncRNA	6.107118667	0.001067	0.004429
AC012508.2	lncRNA	3.795297214	0.00109	0.004514
AL022344.2	lncRNA	3.355208412	0.001095	0.004529
PSORS1C3	lncRNA	2.28682912	0.001102	0.00455
AC022182.1	lncRNA	3.610152271	0.001113	0.004594
AC011944.1	lncRNA	2.967084575	0.001128	0.004645
NFIA-AS2	lncRNA	2.867941564	0.00113	0.00465
AC004253.2	lncRNA	4.015380661	0.001133	0.004657
AC100827.4	lncRNA	2.942677503	0.001143	0.004696
AL512363.1	lncRNA	3.718005152	0.001144	0.004696
AL445490.1	lncRNA	2.777195624	0.00115	0.004719

AC023301.1	lncRNA	5.018315923	0.001161	0.004756
LINC00865	lncRNA	2.334604884	0.001171	0.004782
STXBP5-AS1	lncRNA	2.645202472	0.001175	0.004796
AC005392.3	lncRNA	4.571977075	0.001197	0.004882
AC004241.4	lncRNA	3.202600028	0.001202	0.004895
AL513217.1	lncRNA	3.084898866	0.001203	0.004897
EHMT2-AS1	lncRNA	2.512209933	0.001206	0.004904
LINC00702	lncRNA	2.067824998	0.00121	0.004914
LINC00466	lncRNA	5.378467829	0.001221	0.004953
AC091153.2	lncRNA	3.813835865	0.001222	0.004955
LINC02099	lncRNA	2.936320156	0.001227	0.00497
AC234782.2	lncRNA	2.052647831	0.001237	0.005003
AL445647.1	lncRNA	4.883742289	0.00124	0.005014
AL133319.1	lncRNA	-2.373445938	0.001263	0.00509
AC007570.1	lncRNA	2.674583696	0.001263	0.00509
AC091946.2	lncRNA	4.329951011	0.001264	0.00509
BBOX1-AS1	lncRNA	5.453517599	0.001268	0.005104
MIR200CHG	lncRNA	7.177196885	0.00127	0.005108
LINC01133	lncRNA	4.853266494	0.001275	0.005126
AP000525.1	lncRNA	2.926038656	0.001293	0.005181
AC018553.1	lncRNA	3.534511839	0.001296	0.005184
TTLL10-AS1	lncRNA	4.397001708	0.001302	0.005204
LINC02152	lncRNA	4.429832677	0.001307	0.005219
AL356272.1	lncRNA	-2.432062909	0.001308	0.00522
LINC00926	lncRNA	2.880886847	0.001311	0.005228
AC012074.1	lncRNA	2.019083671	0.001354	0.005379
AC073517.1	lncRNA	2.149610678	0.001356	0.00538
AC121757.1	lncRNA	2.186599636	0.001393	0.005504
AC138625.1	lncRNA	3.063281143	0.001399	0.005522
AP000924.1	lncRNA	-2.201207679	0.001408	0.005551
AP003481.1	lncRNA	4.48203089	0.001408	0.005551
AC023090.1	lncRNA	3.735767666	0.00144	0.005672
KIAA0087	lncRNA	3.472964822	0.001454	0.005717
AC009955.3	lncRNA	2.723677529	0.001461	0.005744
LINC00304	lncRNA	2.502887154	0.001495	0.005853
LINC02253	lncRNA	6.875464809	0.001508	0.0059
AC093627.7	lncRNA	3.000208157	0.001509	0.005901
SATB2-AS1	lncRNA	2.097175594	0.00152	0.005937
AF131215.7	lncRNA	2.309725304	0.001527	0.00596
AC007128.1	lncRNA	6.138296172	0.001528	0.005962
AC138466.1	lncRNA	2.727981194	0.001535	0.005985
ARHGEF38-IT1	lncRNA	2.601152742	0.001542	0.006
AC068722.2	lncRNA	2.097104452	0.001542	0.006
AC011498.3	lncRNA	3.93023927	0.001572	0.006104
AC023794.7	lncRNA	2.49312128	0.001579	0.006124
AC007368.1	lncRNA	4.164483543	0.001587	0.006154
AC013400.1	lncRNA	2.743514311	0.001596	0.006182

AL929236.1	lncRNA	2.492311288	0.001601	0.006195
AC015712.7	lncRNA	3.15393685	0.001605	0.006209
AC078922.1	lncRNA	3.146821538	0.00161	0.006221
AC098864.1	lncRNA	5.362516351	0.001618	0.006248
AL592430.1	lncRNA	2.512979503	0.001627	0.006279
LINC00970	lncRNA	5.182550248	0.001628	0.006282
LINC02454	lncRNA	4.774813993	0.001646	0.006336
CLLU1	lncRNA	7.110313142	0.001646	0.006336
AC015987.1	lncRNA	2.611901114	0.001649	0.006344
AC005954.1	lncRNA	2.069041126	0.001659	0.006376
HIF1A-AS2	lncRNA	2.582526557	0.001663	0.00639
RERG-IT1	lncRNA	3.871526517	0.001672	0.006419
AC010615.2	lncRNA	2.40871347	0.001682	0.006449
LINC02159	lncRNA	4.220546183	0.001683	0.006449
LINC00336	lncRNA	3.672181282	0.001686	0.006458
LINC00866	lncRNA	2.825128056	0.001716	0.006558
CLDN10-AS1	lncRNA	5.608183217	0.001748	0.006664
AC120498.3	lncRNA	3.193742426	0.001783	0.006786
AC091544.2	lncRNA	3.447604841	0.001788	0.006803
AL135999.1	lncRNA	3.111812231	0.001813	0.006884
AC010442.1	lncRNA	2.235439025	0.00182	0.006903
IL12A-AS1	lncRNA	2.984219149	0.001823	0.006908
AL034346.1	lncRNA	4.171328195	0.001827	0.006922
AP001803.2	lncRNA	6.811132352	0.001845	0.006984
AC127496.2	lncRNA	2.661036704	0.001891	0.007134
AC138655.1	lncRNA	3.95480982	0.001899	0.007155
LINC01844	lncRNA	3.522719142	0.001905	0.007172
AC010478.1	lncRNA	5.214858429	0.001911	0.007188
AL356417.2	lncRNA	2.348044605	0.001928	0.00724
LINC00402	lncRNA	-2.446864139	0.001971	0.007387
AC006064.3	lncRNA	2.018678798	0.001973	0.00739
AC114811.2	lncRNA	3.09558135	0.001983	0.007425
AC010641.1	lncRNA	5.11575553	0.001991	0.007451
AC025171.3	lncRNA	2.553297654	0.002023	0.007557
SERTAD4-AS1	lncRNA	2.345376912	0.002028	0.007572
AL135787.1	lncRNA	-2.114613257	0.002045	0.00763
AL592071.1	lncRNA	2.423018737	0.002048	0.007638
AC025035.1	lncRNA	4.004257101	0.002088	0.007762
AC114284.1	lncRNA	2.147025009	0.002098	0.007785
AC015849.1	lncRNA	2.792671029	0.002152	0.007972
AL513327.2	lncRNA	3.744353589	0.002153	0.007972
AC015961.1	lncRNA	2.425857647	0.002169	0.008025
CR559946.1	lncRNA	2.742285761	0.002174	0.00804
AL354919.2	lncRNA	5.233274053	0.002177	0.008046
AC092620.1	lncRNA	2.699160243	0.002191	0.008089
LINC01775	lncRNA	3.253077732	0.002198	0.008098
LINC02389	lncRNA	3.472880657	0.002204	0.008117

AC016723.1	lncRNA	-2.020126085	0.00223	0.008198
LINC01060	lncRNA	5.405644531	0.002267	0.008313
AL355810.1	lncRNA	2.837274733	0.002287	0.008374
AL022316.1	lncRNA	2.082774797	0.002301	0.008408
AL139275.2	lncRNA	6.82014365	0.002328	0.008495
AC008870.3	lncRNA	3.77893624	0.002378	0.008652
AL035448.1	lncRNA	2.284453648	0.002406	0.00874
AC121338.1	lncRNA	3.652395306	0.002429	0.008816
LINC01481	lncRNA	2.20796907	0.002436	0.008836
AC122710.2	lncRNA	2.51890951	0.002447	0.008872
AL353751.1	lncRNA	2.217943843	0.002448	0.008872
AC099552.1	lncRNA	5.794274724	0.002479	0.00897
AL079303.1	lncRNA	5.510948127	0.002494	0.009015
AC105446.1	lncRNA	2.347248768	0.002495	0.009016
AC021092.1	lncRNA	2.119086236	0.002496	0.009016
ITGB1-DT	lncRNA	2.640839586	0.002505	0.009038
CASC11	lncRNA	2.72773819	0.002511	0.00905
AL355312.3	lncRNA	5.234145973	0.002513	0.009053
GAPLINC	lncRNA	2.366384933	0.002532	0.009108
AC006237.1	lncRNA	4.853166137	0.002536	0.009119
AL590644.1	lncRNA	5.060610511	0.002586	0.009275
LINC01970	lncRNA	2.120018443	0.00259	0.009285
TESC-AS1	lncRNA	2.505545146	0.002606	0.00934
HOTTIP	lncRNA	4.193954741	0.002614	0.009364
AC100791.2	lncRNA	4.927788004	0.002662	0.009507
AC011294.1	lncRNA	2.390189612	0.00269	0.009591
AC093734.1	lncRNA	2.733565697	0.002697	0.009612
AC092794.2	lncRNA	2.170884402	0.002706	0.009641
AC005790.1	lncRNA	3.749399014	0.002708	0.009643
AC120498.10	lncRNA	2.615921623	0.00272	0.009671
AC087623.4	lncRNA	2.552836536	0.002725	0.009685
AC097634.3	lncRNA	3.948880706	0.002742	0.009732
LINC01524	lncRNA	4.491071935	0.002742	0.009732
AC016705.1	lncRNA	3.028108133	0.002744	0.009733
LINC01812	lncRNA	5.607955089	0.002753	0.009755
AL358473.1	lncRNA	3.077258154	0.002762	0.009781
CCND2-AS1	lncRNA	3.75187269	0.002765	0.009783
AC025539.1	lncRNA	2.380050534	0.002771	0.0098
AL139147.1	lncRNA	3.966221065	0.002775	0.009806
AC002076.1	lncRNA	4.394333663	0.002775	0.009806
AC239803.3	lncRNA	3.973531541	0.002799	0.009888
AL096803.3	lncRNA	3.958236873	0.002802	0.009894
hsa-mir-148a	miRNA	-2.67846	2.49E-27	1.40E-24
hsa-mir-182	miRNA	4.492192	6.72E-26	1.88E-23
hsa-mir-183	miRNA	4.825852	4.55E-25	8.52E-23
hsa-mir-5589	miRNA	-5.37808	4.07E-21	5.71E-19
hsa-mir-5588	miRNA	-3.55713	5.69E-21	6.39E-19

hsa-mir-4662a	miRNA	-3.03315	3.46E-20	3.23E-18
hsa-mir-1258	miRNA	-4.73121	6.44E-20	5.16E-18
hsa-mir-101-1	miRNA	-2.00915	3.80E-19	2.33E-17
hsa-mir-4686	miRNA	-4.50064	4.00E-19	2.33E-17
hsa-mir-378a	miRNA	-2.89994	4.16E-19	2.33E-17
hsa-mir-101-2	miRNA	-2.01315	4.89E-19	2.49E-17
hsa-mir-505	miRNA	-2.00679	2.01E-18	9.39E-17
hsa-mir-96	miRNA	4.784818	5.41E-18	2.34E-16
hsa-mir-139	miRNA	-2.74117	1.13E-17	4.53E-16
hsa-let-7c	miRNA	-2.10674	1.05E-16	3.94E-15
hsa-mir-21	miRNA	2.502849	4.81E-16	1.69E-14
hsa-mir-99a	miRNA	-2.22495	1.06E-15	3.51E-14
hsa-mir-4524a	miRNA	-3.64294	3.06E-15	9.53E-14
hsa-mir-490	miRNA	-4.28443	2.77E-14	8.19E-13
hsa-mir-378c	miRNA	-2.62909	1.22E-13	3.41E-12
hsa-mir-675	miRNA	-3.99871	5.96E-13	1.59E-11
hsa-mir-1468	miRNA	-2.18834	2.80E-11	6.83E-10
hsa-mir-34c	miRNA	4.975575	3.42E-11	8.00E-10
hsa-mir-483	miRNA	-3.37145	4.95E-11	1.11E-09
hsa-mir-92b	miRNA	2.953656	7.18E-11	1.55E-09
hsa-mir-200b	miRNA	2.831124	8.82E-11	1.83E-09
hsa-mir-6761	miRNA	-2.17754	2.50E-10	5.01E-09
hsa-mir-200a	miRNA	2.683969	9.63E-10	1.86E-08
hsa-mir-1295b	miRNA	-4.2242	1.12E-09	2.09E-08
hsa-mir-378d-2	miRNA	-2.99777	2.04E-09	3.70E-08
hsa-mir-885	miRNA	-3.7846	2.37E-09	4.16E-08
hsa-mir-34b	miRNA	6.216486	3.71E-09	6.15E-08
hsa-mir-122	miRNA	-4.04868	4.64E-09	7.25E-08
hsa-mir-181d	miRNA	2.604536	5.23E-09	7.93E-08
hsa-mir-181b-2	miRNA	2.140482	8.79E-09	1.30E-07
hsa-mir-551b	miRNA	-2.47243	9.63E-09	1.39E-07
hsa-mir-181b-1	miRNA	2.078805	1.37E-08	1.90E-07
hsa-mir-222	miRNA	2.0266	1.42E-08	1.90E-07
hsa-mir-429	miRNA	2.768164	2.12E-08	2.71E-07
hsa-mir-383	miRNA	-2.73171	2.36E-08	2.94E-07
hsa-mir-135b	miRNA	4.842512	3.78E-07	4.00E-06
hsa-mir-708	miRNA	2.714289	2.12E-06	2.05E-05
hsa-mir-144	miRNA	-2.21835	2.25E-06	2.14E-05
hsa-mir-548b	miRNA	-2.40284	1.04E-05	9.08E-05
hsa-mir-561	miRNA	3.372769	1.39E-05	0.000118
hsa-mir-6715a	miRNA	-3.41851	1.39E-05	0.000118
hsa-mir-1266	miRNA	2.214034	1.64E-05	0.000137
hsa-mir-196b	miRNA	4.282367	2.57E-05	0.0002
hsa-mir-200c	miRNA	5.204526	3.18E-05	0.000241
hsa-mir-141	miRNA	4.761694	8.74E-05	0.000621
hsa-mir-10b	miRNA	2.429236	0.000114	0.000769
hsa-mir-3934	miRNA	2.118709	0.000178	0.001137

hsa-mir-615	miRNA	5.712798	0.000218	0.001345
hsa-mir-6720	miRNA	3.123045	0.000223	0.001357
hsa-mir-1224	miRNA	4.50066	0.00027	0.00163
hsa-mir-196a-1	miRNA	5.120034	0.000283	0.001682
hsa-mir-203b	miRNA	5.063323	0.000293	0.00171
hsa-mir-187	miRNA	2.697406	0.00055	0.002857
hsa-mir-196a-2	miRNA	4.750498	0.000879	0.004327
hsa-mir-526b	miRNA	6.041833	0.001032	0.005031
hsa-mir-4473	miRNA	2.371743	0.001177	0.005598
hsa-mir-211	miRNA	-2.17008	0.001418	0.00652
hsa-mir-31	miRNA	4.181871	0.001692	0.007656
hsa-mir-577	miRNA	3.912126	0.002046	0.008827
KCNN2	mRNA	-5.54357	1.58E-75	2.79E-71
USH2A	mRNA	-6.3925	1.00E-74	8.89E-71
MSMO1	mRNA	-3.70744	3.13E-70	1.85E-66
RCL1	mRNA	-3.76571	5.00E-70	2.21E-66
SC5D	mRNA	-4.11784	8.48E-69	3.00E-65
GCDH	mRNA	-3.23893	1.28E-68	3.78E-65
LCAT	mRNA	-4.67143	8.45E-65	2.14E-61
ALAS1	mRNA	-3.76165	5.79E-62	1.28E-58
ACAA1	mRNA	-3.704	9.36E-60	1.73E-56
ADI1	mRNA	-3.47058	9.78E-60	1.73E-56
CAT	mRNA	-3.92948	3.68E-59	5.91E-56
PEMT	mRNA	-3.44062	3.49E-58	5.14E-55
KDM8	mRNA	-4.18056	5.67E-58	7.71E-55
ACADSB	mRNA	-4.84086	6.71E-58	8.48E-55
DHODH	mRNA	-3.43308	4.71E-57	5.55E-54
GLUD1	mRNA	-3.23404	5.06E-55	5.59E-52
DCXR	mRNA	-5.13775	4.92E-53	5.12E-50
AADAT	mRNA	-4.44121	1.25E-52	1.23E-49
PCK2	mRNA	-4.75951	1.46E-51	1.35E-48
SORD	mRNA	-4.76608	1.53E-51	1.35E-48
HMGCL	mRNA	-3.39374	6.25E-51	5.27E-48
MTHFD1	mRNA	-3.34384	7.50E-51	6.03E-48
ACOT2	mRNA	-3.17691	5.33E-50	4.10E-47
FBP1	mRNA	-4.91383	1.78E-49	1.31E-46
DHRS1	mRNA	-3.13334	2.64E-49	1.86E-46
DNAJC25	mRNA	-2.94133	3.20E-48	2.18E-45
ESR1	mRNA	-5.37537	2.41E-47	1.58E-44
CFL2	mRNA	-3.14975	2.05E-46	1.29E-43
MUT	mRNA	-3.11995	3.57E-46	2.18E-43
KHK	mRNA	-4.95881	7.24E-46	4.27E-43
QDPR	mRNA	-3.12626	1.05E-45	5.99E-43
ABCG2	mRNA	-4.28951	1.62E-45	8.77E-43
AGL	mRNA	-2.5578	1.64E-45	8.77E-43
ADAMTS13	mRNA	-3.22111	3.14E-45	1.63E-42
FXYD1	mRNA	-6.19151	6.92E-45	3.50E-42

MST1	mRNA	-4.96101	7.89E-45	3.88E-42
TCTEX1D1	mRNA	-4.46628	1.28E-44	6.14E-42
SLC25A13	mRNA	-2.85916	2.19E-44	1.02E-41
TMEM56	mRNA	-3.88786	2.79E-44	1.27E-41
INSIG1	mRNA	-4.75388	4.44E-44	1.96E-41
CD302	mRNA	-4.46513	2.48E-43	1.07E-40
MEP1B	mRNA	-7.69172	1.06E-42	4.46E-40
ETFRF1	mRNA	-2.55622	4.43E-42	1.82E-39
TRIB1	mRNA	-3.17377	1.52E-41	6.10E-39
DHTKD1	mRNA	-3.28145	1.89E-41	7.44E-39
SLC16A2	mRNA	-3.6374	1.97E-41	7.58E-39
MFAP3L	mRNA	-4.3797	2.03E-41	7.64E-39
SLCO2B1	mRNA	-4.07269	4.00E-41	1.47E-38
GSTZ1	mRNA	-3.70301	5.83E-41	2.10E-38
KCNJ8	mRNA	-4.32431	1.17E-40	4.12E-38
PANK1	mRNA	-3.6617	1.19E-40	4.12E-38
GLYCTK	mRNA	-4.20737	3.82E-40	1.30E-37
HADH	mRNA	-3.01482	5.05E-40	1.68E-37
KMO	mRNA	-5.32571	9.92E-40	3.25E-37
CDC37L1	mRNA	-2.40769	3.75E-39	1.21E-36
TCAIM	mRNA	-2.16173	4.59E-39	1.45E-36
ISOC1	mRNA	-2.42442	5.87E-39	1.82E-36
PHYH	mRNA	-3.82162	9.43E-39	2.86E-36
RMDN2	mRNA	-2.82053	9.54E-39	2.86E-36
SARDH	mRNA	-5.30644	1.16E-38	3.41E-36
MT1X	mRNA	-5.15922	1.23E-38	3.57E-36
EBP	mRNA	-3.03455	1.99E-38	5.67E-36
SOD1	mRNA	-2.79397	2.02E-38	5.67E-36
CD14	mRNA	-4.05793	4.08E-38	1.13E-35
SLC25A20	mRNA	-3.4646	4.42E-38	1.20E-35
ABCA6	mRNA	-5.46488	4.69E-38	1.26E-35
ALDH6A1	mRNA	-4.43003	5.79E-38	1.53E-35
CTH	mRNA	-4.92599	1.49E-37	3.88E-35
RCAN1	mRNA	-3.82925	1.72E-37	4.40E-35
SLC27A5	mRNA	-6.35098	3.55E-37	8.97E-35
CYP27A1	mRNA	-4.45507	4.57E-37	1.14E-34
GRHPR	mRNA	-3.05408	5.21E-37	1.28E-34
SPRYD4	mRNA	-2.8135	6.72E-37	1.63E-34
ALDH5A1	mRNA	-3.17358	1.36E-36	3.25E-34
AIG1	mRNA	-2.56905	1.76E-36	4.14E-34
ETFDH	mRNA	-3.50391	2.56E-36	5.96E-34
ACOX2	mRNA	-5.83207	3.02E-36	6.93E-34
ATP11C	mRNA	-2.86234	3.35E-36	7.61E-34
GCH1	mRNA	-3.53958	3.90E-36	8.74E-34
TMEM220	mRNA	-4.23784	4.39E-36	9.71E-34
ACSL1	mRNA	-5.24338	4.84E-36	1.06E-33
SEC14L2	mRNA	-4.19807	7.84E-36	1.69E-33

ACAT2	mRNA	-3.23221	1.25E-35	2.66E-33
ABCA8	mRNA	-5.02427	1.33E-35	2.80E-33
RANBP3L	mRNA	-5.27388	2.04E-35	4.26E-33
HAGH	mRNA	-2.78969	2.12E-35	4.37E-33
TIGD2	mRNA	-2.97549	3.85E-35	7.82E-33
IVD	mRNA	-2.52195	5.68E-35	1.14E-32
SHMT1	mRNA	-4.06401	7.34E-35	1.46E-32
IL17RC	mRNA	-2.33886	1.43E-34	2.81E-32
ECHS1	mRNA	-3.30903	1.68E-34	3.26E-32
HSDL2	mRNA	-2.89017	1.84E-34	3.52E-32
SLC2A9	mRNA	-4.19208	1.85E-34	3.52E-32
NR1I3	mRNA	-5.89475	2.27E-34	4.27E-32
DBH	mRNA	-5.69884	3.00E-34	5.58E-32
NADK2	mRNA	-3.64499	3.35E-34	6.17E-32
ERLIN1	mRNA	-2.4635	3.53E-34	6.44E-32
EPB41L4B	mRNA	-3.78594	3.96E-34	7.14E-32
VWCE	mRNA	-4.35857	1.17E-33	2.10E-31
HIBCH	mRNA	-2.51534	1.25E-33	2.20E-31
ACOX1	mRNA	-2.73343	1.26E-33	2.20E-31
TPPP2	mRNA	-5.37368	1.36E-33	2.35E-31
DHRS4	mRNA	-2.53393	1.50E-33	2.58E-31
ALDH2	mRNA	-4.04794	1.53E-33	2.61E-31
SOX5	mRNA	-4.49499	2.65E-33	4.47E-31
NSUN6	mRNA	-2.45039	2.97E-33	4.95E-31
GPHN	mRNA	-2.4684	3.53E-33	5.84E-31
SCP2	mRNA	-3.08116	6.02E-33	9.86E-31
MPDZ	mRNA	-2.94121	7.26E-33	1.18E-30
PLIN1	mRNA	-5.10219	1.09E-32	1.75E-30
FUOM	mRNA	-4.03769	1.47E-32	2.34E-30
SUCLG2	mRNA	-2.52438	1.83E-32	2.90E-30
RIDA	mRNA	-3.91994	2.09E-32	3.27E-30
ANG	mRNA	-4.93491	2.50E-32	3.88E-30
PGRMC1	mRNA	-2.73598	2.71E-32	4.18E-30
SRD5A2	mRNA	-7.3143	3.37E-32	5.13E-30
SLC7A2	mRNA	-4.66189	5.40E-32	8.17E-30
SLC25A42	mRNA	-3.61658	6.01E-32	9.01E-30
ASPDH	mRNA	-6.30825	1.06E-31	1.58E-29
ITIH1	mRNA	-6.3708	1.54E-31	2.26E-29
TM7SF2	mRNA	-3.43064	2.34E-31	3.42E-29
ABAT	mRNA	-4.81033	2.54E-31	3.68E-29
ETNK2	mRNA	-4.07469	2.64E-31	3.80E-29
DGAT2	mRNA	-4.69945	2.67E-31	3.81E-29
PHYHD1	mRNA	-4.38476	3.75E-31	5.30E-29
HDAC6	mRNA	-2.03827	3.79E-31	5.32E-29
GADD45G	mRNA	-4.44307	4.47E-31	6.22E-29
GOT2	mRNA	-2.73612	4.65E-31	6.43E-29
BCKDHB	mRNA	-3.09599	6.08E-31	8.33E-29

PPP1R1A	mRNA	-5.26392	6.15E-31	8.33E-29
ACAD11	mRNA	-3.51993	6.17E-31	8.33E-29
IQGAP2	mRNA	-3.10392	6.45E-31	8.64E-29
A2M	mRNA	-3.80544	6.74E-31	8.97E-29
CPED1	mRNA	-3.82755	7.75E-31	1.02E-28
HSD17B4	mRNA	-2.59338	7.95E-31	1.04E-28
TSKU	mRNA	-4.02965	8.11E-31	1.06E-28
SLC35D1	mRNA	-3.10453	8.38E-31	1.08E-28
CYP2J2	mRNA	-3.78395	1.11E-30	1.42E-28
MAN1A1	mRNA	-3.2235	1.44E-30	1.84E-28
BTD	mRNA	-2.66294	1.65E-30	2.09E-28
SUOX	mRNA	-2.21098	2.45E-30	3.08E-28
HAAO	mRNA	-4.08871	3.75E-30	4.67E-28
N4BP2L1	mRNA	-2.72433	4.72E-30	5.83E-28
FAM151A	mRNA	-5.87991	4.90E-30	6.02E-28
CYB5A	mRNA	-3.5466	5.93E-30	7.23E-28
APOE	mRNA	-4.68448	6.23E-30	7.55E-28
XYLB	mRNA	-3.47129	6.69E-30	8.05E-28
PLIN2	mRNA	-4.06675	8.14E-30	9.73E-28
CRYL1	mRNA	-3.24614	1.00E-29	1.19E-27
PEBP1	mRNA	-2.70665	1.02E-29	1.21E-27
PDE3B	mRNA	-4.095	1.42E-29	1.66E-27
ACSS2	mRNA	-2.42937	2.03E-29	2.36E-27
EPHX1	mRNA	-3.82311	2.11E-29	2.44E-27
CYP4V2	mRNA	-2.74189	2.61E-29	3.00E-27
KLKB1	mRNA	-4.97847	4.11E-29	4.69E-27
RGN	mRNA	-4.44997	4.16E-29	4.72E-27
TKFC	mRNA	-2.92745	4.45E-29	5.02E-27
DEPDC7	mRNA	-4.53314	4.59E-29	5.14E-27
SFXN1	mRNA	-2.20973	5.38E-29	5.99E-27
HIBADH	mRNA	-2.4927	7.32E-29	8.04E-27
SLC16A13	mRNA	-2.80854	7.32E-29	8.04E-27
ETS2	mRNA	-2.57402	7.45E-29	8.13E-27
ZNF385B	mRNA	-5.46148	7.57E-29	8.21E-27
C1R	mRNA	-3.48368	8.15E-29	8.79E-27
ARID3C	mRNA	-5.96394	9.30E-29	9.98E-27
SLC6A13	mRNA	-6.32145	1.07E-28	1.14E-26
ALDH1L1	mRNA	-5.57683	1.34E-28	1.42E-26
CYP2C9	mRNA	-5.77091	1.45E-28	1.53E-26
MPC1	mRNA	-2.94338	1.72E-28	1.80E-26
ASS1	mRNA	-3.96648	1.89E-28	1.96E-26
EHHADH	mRNA	-4.82345	1.97E-28	2.04E-26
ZFP1	mRNA	-2.24819	2.28E-28	2.34E-26
GNE	mRNA	-2.99596	2.51E-28	2.57E-26
CYP2D6	mRNA	-5.4954	3.01E-28	3.06E-26
GNMT	mRNA	-6.27482	3.14E-28	3.17E-26
ACOT1	mRNA	-3.63591	3.31E-28	3.33E-26

MRO	mRNA	-4.01273	3.86E-28	3.86E-26
SLC47A1	mRNA	-5.03955	4.93E-28	4.90E-26
ADHFE1	mRNA	-3.47681	5.57E-28	5.50E-26
ACACB	mRNA	-3.1141	6.31E-28	6.21E-26
SORL1	mRNA	-2.99184	6.42E-28	6.27E-26
GLS2	mRNA	-5.08165	6.92E-28	6.73E-26
RASGEF1B	mRNA	-3.1112	1.09E-27	1.05E-25
ENO3	mRNA	-3.85928	1.10E-27	1.06E-25
GLTPD2	mRNA	-4.54155	1.25E-27	1.20E-25
MTHFS	mRNA	-2.57226	1.27E-27	1.21E-25
TUBE1	mRNA	-2.57411	2.06E-27	1.95E-25
MYRIP	mRNA	-4.0257	2.36E-27	2.22E-25
ACSM5	mRNA	-6.16184	2.40E-27	2.25E-25
ACAA2	mRNA	-3.02087	2.74E-27	2.55E-25
ACAT1	mRNA	-3.48713	3.00E-27	2.78E-25
POR	mRNA	-2.7376	3.08E-27	2.84E-25
IL27	mRNA	-5.77446	3.34E-27	3.06E-25
SLC6A12	mRNA	-5.61583	3.74E-27	3.41E-25
PIGV	mRNA	-2.01397	3.76E-27	3.41E-25
SCD	mRNA	-3.97193	4.01E-27	3.62E-25
TSLP	mRNA	-4.41061	4.03E-27	3.62E-25
AK3	mRNA	-2.22163	4.05E-27	3.62E-25
CTNNA3	mRNA	-4.57776	4.08E-27	3.63E-25
AL121845.2	mRNA	-6.06695	4.54E-27	4.01E-25
ENPEP	mRNA	-3.47013	4.56E-27	4.01E-25
TTPAL	mRNA	-2.38639	4.68E-27	4.10E-25
HEPACAM	mRNA	-7.53768	4.87E-27	4.24E-25
CMTM8	mRNA	-2.53534	4.91E-27	4.26E-25
SPACA7	mRNA	-6.25107	5.39E-27	4.65E-25
ACADM	mRNA	-2.60116	6.09E-27	5.23E-25
METTL7A	mRNA	-3.27248	7.00E-27	5.98E-25
SELENOP	mRNA	-3.98414	8.81E-27	7.49E-25
AC006254.1	mRNA	-4.41129	1.02E-26	8.67E-25
PRSS16	mRNA	5.440844	1.30E-26	1.10E-24
F11	mRNA	-4.61766	1.40E-26	1.18E-24
CBS	mRNA	-4.25604	1.45E-26	1.21E-24
DHRS3	mRNA	-2.55394	1.54E-26	1.28E-24
PBLD	mRNA	-4.12072	2.04E-26	1.69E-24
PPARA	mRNA	-2.6027	2.06E-26	1.69E-24
CASC10	mRNA	-3.52284	2.92E-26	2.39E-24
C1S	mRNA	-3.58447	3.02E-26	2.46E-24
IQCE	mRNA	2.804402	3.52E-26	2.85E-24
GCK	mRNA	-5.61645	3.65E-26	2.95E-24
NAT2	mRNA	-6.24447	3.98E-26	3.20E-24
FAHD2A	mRNA	-2.20367	4.33E-26	3.47E-24
ECM2	mRNA	-3.26712	5.06E-26	4.03E-24
PTGR1	mRNA	-4.20039	7.09E-26	5.62E-24

EPHX2	mRNA	-3.54899	7.77E-26	6.13E-24
ACADS	mRNA	-2.95875	8.10E-26	6.37E-24
ZFAND5	mRNA	-2.36399	8.38E-26	6.54E-24
MSRA	mRNA	-2.41833	8.39E-26	6.54E-24
DMGDH	mRNA	-6.11826	8.59E-26	6.66E-24
TMEM192	mRNA	-2.11021	8.71E-26	6.73E-24
SMOC1	mRNA	-3.94682	9.29E-26	7.15E-24
GCAT	mRNA	-3.06375	9.35E-26	7.16E-24
RNF152	mRNA	-3.06762	9.85E-26	7.51E-24
RBP5	mRNA	-4.5985	1.29E-25	9.79E-24
PAIP2B	mRNA	-3.12957	1.34E-25	1.01E-23
HACL1	mRNA	-2.3685	1.34E-25	1.01E-23
ESRP1	mRNA	5.274749	1.40E-25	1.05E-23
NTF3	mRNA	-4.21694	1.49E-25	1.12E-23
CNDP1	mRNA	-6.18955	2.01E-25	1.50E-23
PPP1R3B	mRNA	-3.23512	2.07E-25	1.53E-23
SLC30A1	mRNA	-2.5665	2.25E-25	1.66E-23
SPINT1	mRNA	4.629738	2.28E-25	1.67E-23
UROC1	mRNA	-7.45607	2.53E-25	1.85E-23
PGM1	mRNA	-2.75792	2.57E-25	1.87E-23
COLEC10	mRNA	-4.33205	2.66E-25	1.93E-23
MT2A	mRNA	-4.33292	2.89E-25	2.08E-23
GGCX	mRNA	-2.30153	3.06E-25	2.20E-23
ECI2	mRNA	-2.6082	3.51E-25	2.51E-23
ACOT13	mRNA	-2.0605	4.22E-25	3.01E-23
DDT	mRNA	-2.98015	4.61E-25	3.28E-23
APOM	mRNA	-4.54493	6.24E-25	4.42E-23
CYP39A1	mRNA	-5.33237	6.35E-25	4.47E-23
ALDH9A1	mRNA	-2.15356	6.70E-25	4.70E-23
DHRS4L2	mRNA	-2.42848	7.79E-25	5.44E-23
HSD17B8	mRNA	-2.15678	8.29E-25	5.77E-23
CPEB3	mRNA	-3.0179	8.39E-25	5.82E-23
UGT2A1	mRNA	-6.10064	9.54E-25	6.59E-23
MLYCD	mRNA	-2.3147	1.12E-24	7.73E-23
ADK	mRNA	-2.38686	1.42E-24	9.76E-23
FNDC5	mRNA	-5.19449	1.43E-24	9.76E-23
LRRC3	mRNA	-3.43867	1.54E-24	1.05E-22
AQP11	mRNA	-3.16002	1.55E-24	1.05E-22
LYVE1	mRNA	-3.73845	1.59E-24	1.07E-22
MT-ND6	mRNA	-3.15113	1.92E-24	1.29E-22
BCHE	mRNA	-5.71843	1.98E-24	1.33E-22
CFI	mRNA	-3.33519	2.09E-24	1.40E-22
AFMID	mRNA	-2.10907	2.33E-24	1.55E-22
DNMT3L	mRNA	-7.07268	2.33E-24	1.55E-22
PKM	mRNA	4.201314	2.51E-24	1.65E-22
IDI1	mRNA	-2.24192	2.61E-24	1.71E-22
SLC46A3	mRNA	-3.44491	2.81E-24	1.84E-22

RDH5	mRNA	-3.5004	2.86E-24	1.85E-22
CCDC196	mRNA	-5.38474	2.95E-24	1.90E-22
LDHD	mRNA	-4.36742	3.38E-24	2.18E-22
CYP3A43	mRNA	-6.15865	3.64E-24	2.33E-22
GPR146	mRNA	-3.12266	4.38E-24	2.80E-22
MMAB	mRNA	-2.52682	4.70E-24	2.99E-22
ADGRA3	mRNA	-2.25371	5.75E-24	3.64E-22
AGMAT	mRNA	-3.9983	5.77E-24	3.65E-22
SERPINA4	mRNA	-4.19905	6.51E-24	4.10E-22
CYP4F2	mRNA	-5.67614	7.30E-24	4.58E-22
DECR1	mRNA	-2.35006	8.00E-24	5.00E-22
PCTP	mRNA	-2.3039	1.13E-23	7.05E-22
MGMT	mRNA	-2.44304	1.15E-23	7.17E-22
SLC31A1	mRNA	-2.71915	1.24E-23	7.66E-22
DHCR7	mRNA	-2.7318	1.25E-23	7.69E-22
SLC6A1	mRNA	-4.67527	1.32E-23	8.11E-22
SLC25A18	mRNA	-4.77516	1.43E-23	8.74E-22
LRRC1	mRNA	3.624768	1.47E-23	8.94E-22
CISH	mRNA	-2.85224	1.48E-23	8.97E-22
TTC38	mRNA	-2.39192	1.54E-23	9.31E-22
SMIM14	mRNA	-2.67482	1.78E-23	1.07E-21
ABCC6	mRNA	-3.63274	2.07E-23	1.25E-21
AKR1C4	mRNA	-5.68365	2.39E-23	1.43E-21
PLGLB1	mRNA	-4.22557	2.43E-23	1.45E-21
ITPR2	mRNA	-2.85188	2.46E-23	1.47E-21
MAN1C1	mRNA	-3.5292	2.64E-23	1.57E-21
TMEM176B	mRNA	-3.4225	3.24E-23	1.92E-21
HGFAC	mRNA	-5.62744	3.66E-23	2.16E-21
AMT	mRNA	-2.45336	3.67E-23	2.16E-21
APOC1	mRNA	-5.47977	3.83E-23	2.25E-21
APOC4	mRNA	-5.10532	3.88E-23	2.27E-21
PEX11A	mRNA	-2.24884	4.08E-23	2.38E-21
PECR	mRNA	-2.76242	4.20E-23	2.43E-21
NOCT	mRNA	-2.86438	4.31E-23	2.49E-21
MSRB1	mRNA	-2.43403	4.94E-23	2.85E-21
SHF	mRNA	-2.86873	5.05E-23	2.90E-21
FERMT2	mRNA	-2.36538	5.10E-23	2.92E-21
AMDHD1	mRNA	-5.3045	5.48E-23	3.12E-21
AZGP1	mRNA	-4.6301	5.68E-23	3.22E-21
UGP2	mRNA	-2.62987	5.71E-23	3.23E-21
C1orf115	mRNA	-2.31024	6.42E-23	3.62E-21
TTC36	mRNA	-6.59182	6.46E-23	3.63E-21
ABCC9	mRNA	-4.36094	7.08E-23	3.97E-21
MASP2	mRNA	-6.02666	9.09E-23	5.06E-21
ITIH4	mRNA	-3.71609	9.42E-23	5.22E-21
SLC25A15	mRNA	-3.51729	9.77E-23	5.40E-21
SLC34A1	mRNA	-4.62662	1.04E-22	5.71E-21

IDO2	mRNA	-5.66927	1.06E-22	5.83E-21
FCN3	mRNA	-4.42672	1.22E-22	6.69E-21
LEAP2	mRNA	-4.50449	1.33E-22	7.23E-21
PROZ	mRNA	-6.19057	1.34E-22	7.27E-21
G6PC	mRNA	-5.5119	1.42E-22	7.72E-21
F12	mRNA	-5.27203	1.55E-22	8.41E-21
CDC14B	mRNA	-2.15454	1.57E-22	8.45E-21
RHOB	mRNA	-2.79239	1.66E-22	8.93E-21
PCP4L1	mRNA	-4.31485	1.74E-22	9.34E-21
PQLC1	mRNA	-2.61335	1.83E-22	9.78E-21
CNGA1	mRNA	-4.00437	1.98E-22	1.05E-20
SDHB	mRNA	-2.23677	2.41E-22	1.27E-20
APOL5	mRNA	-6.30417	2.45E-22	1.29E-20
FADS2	mRNA	-3.3544	2.49E-22	1.31E-20
MT1E	mRNA	-4.41226	2.63E-22	1.37E-20
SOGA1	mRNA	2.491267	2.77E-22	1.45E-20
SLC9A3R2	mRNA	-2.71033	3.10E-22	1.61E-20
PON3	mRNA	-4.67081	3.16E-22	1.64E-20
FGA	mRNA	-5.38363	4.36E-22	2.24E-20
TCP10L	mRNA	-3.32573	4.42E-22	2.27E-20
HMGCR	mRNA	-2.1616	4.48E-22	2.29E-20
SERPING1	mRNA	-2.71052	5.03E-22	2.56E-20
CSAD	mRNA	-2.12274	5.07E-22	2.57E-20
CYP17A1	mRNA	-4.01776	5.08E-22	2.57E-20
BNIP3	mRNA	-2.53553	5.21E-22	2.63E-20
SLC39A14	mRNA	-2.64548	5.24E-22	2.63E-20
OSGIN1	mRNA	-3.42123	5.33E-22	2.67E-20
PNPO	mRNA	-2.15756	5.39E-22	2.69E-20
ACSS3	mRNA	-4.01153	5.73E-22	2.86E-20
ORMDL3	mRNA	-2.15068	6.75E-22	3.36E-20
ERG28	mRNA	-2.03471	6.85E-22	3.40E-20
GLT1D1	mRNA	-5.15935	9.06E-22	4.47E-20
TTPA	mRNA	-5.14231	9.26E-22	4.55E-20
TM6SF2	mRNA	-4.70428	1.08E-21	5.30E-20
TDRD15	mRNA	-6.00308	1.13E-21	5.54E-20
FMO5	mRNA	-3.8033	1.18E-21	5.77E-20
ALDH8A1	mRNA	-5.1637	1.25E-21	6.05E-20
NKIRAS1	mRNA	-2.37126	1.29E-21	6.23E-20
FTCDNL1	mRNA	-3.43799	1.37E-21	6.64E-20
ADRA2B	mRNA	-3.18783	1.48E-21	7.15E-20
GPAM	mRNA	-4.22222	1.49E-21	7.16E-20
BDH1	mRNA	-4.62796	1.56E-21	7.46E-20
RDH16	mRNA	-6.29629	1.58E-21	7.56E-20
HIGD1A	mRNA	-2.39649	1.74E-21	8.29E-20
FAM13A	mRNA	-3.59969	1.87E-21	8.91E-20
SFXN5	mRNA	-2.95016	1.95E-21	9.26E-20
SH2D3A	mRNA	4.286398	2.00E-21	9.47E-20

4-Sep	mRNA	-2.64364	2.49E-21	1.17E-19
ACY1	mRNA	-2.45648	2.51E-21	1.18E-19
GATM	mRNA	-3.97244	2.69E-21	1.26E-19
NUDT7	mRNA	-2.52635	3.27E-21	1.53E-19
MT-ND5	mRNA	-2.84806	3.32E-21	1.55E-19
ARL4D	mRNA	-3.28017	3.53E-21	1.64E-19
PROS1	mRNA	-3.25462	3.71E-21	1.72E-19
ENHO	mRNA	-4.33284	4.01E-21	1.86E-19
PTMS	mRNA	-2.2124	4.02E-21	1.86E-19
XAGE3	mRNA	-4.74957	4.04E-21	1.86E-19
FDX1	mRNA	-2.20656	4.13E-21	1.90E-19
CHAD	mRNA	-4.2514	4.15E-21	1.90E-19
HEY2	mRNA	-2.44248	4.95E-21	2.26E-19
IGSF3	mRNA	4.284833	4.98E-21	2.27E-19
SPINT2	mRNA	3.784944	5.02E-21	2.28E-19
ABHD15	mRNA	-2.11274	5.28E-21	2.39E-19
FOLH1	mRNA	-3.43398	6.12E-21	2.77E-19
CYP4F12	mRNA	-4.27215	6.42E-21	2.90E-19
GPT	mRNA	-3.98764	6.97E-21	3.14E-19
ALDH7A1	mRNA	-2.33286	7.04E-21	3.16E-19
HSD17B14	mRNA	-2.71022	7.78E-21	3.48E-19
CDT1	mRNA	4.091598	8.14E-21	3.63E-19
GALT	mRNA	-2.09471	8.35E-21	3.72E-19
ADH6	mRNA	-5.2813	8.66E-21	3.85E-19
MAPK13	mRNA	3.338278	8.74E-21	3.88E-19
TENM1	mRNA	-4.84275	9.06E-21	4.01E-19
MT1M	mRNA	-4.90264	9.20E-21	4.05E-19
C4B	mRNA	-3.47128	9.31E-21	4.09E-19
FAH	mRNA	-3.09695	9.99E-21	4.36E-19
STIMATE	mRNA	-2.56041	1.01E-20	4.38E-19
MT1G	mRNA	-6.11795	1.02E-20	4.45E-19
LRP1	mRNA	-2.38309	1.04E-20	4.49E-19
MT1F	mRNA	-3.9219	1.12E-20	4.85E-19
HGF	mRNA	-3.15891	1.14E-20	4.91E-19
SERPINF1	mRNA	-3.89601	1.24E-20	5.35E-19
ENPP1	mRNA	-3.94783	1.27E-20	5.44E-19
PGLYRP2	mRNA	-5.96286	1.31E-20	5.59E-19
TMEM176A	mRNA	-3.57141	1.31E-20	5.61E-19
FETUB	mRNA	-6.50933	1.40E-20	5.97E-19
SLC26A1	mRNA	-2.54209	1.57E-20	6.66E-19
SLFN13	mRNA	3.880486	1.68E-20	7.13E-19
RARRES2	mRNA	-3.13975	1.68E-20	7.13E-19
MGST1	mRNA	-3.3304	1.71E-20	7.23E-19
MTSS1	mRNA	-2.43602	1.94E-20	8.17E-19
EVA1A	mRNA	-4.58942	1.98E-20	8.33E-19
C1RL	mRNA	-2.12335	2.04E-20	8.53E-19
TMEM82	mRNA	-5.64966	2.09E-20	8.74E-19

TTR	mRNA	-6.39429	2.51E-20	1.05E-18
MYEF2	mRNA	4.481993	2.78E-20	1.16E-18
DLG3	mRNA	3.122401	2.78E-20	1.16E-18
MT-ND1	mRNA	-2.2678	2.82E-20	1.17E-18
GC	mRNA	-4.52608	2.88E-20	1.19E-18
CDA	mRNA	-3.73591	2.95E-20	1.21E-18
ADH1B	mRNA	-6.27413	3.01E-20	1.24E-18
COL18A1	mRNA	-2.16853	3.06E-20	1.25E-18
PCCB	mRNA	-2.59492	3.15E-20	1.29E-18
CYP2A13	mRNA	-7.37799	3.16E-20	1.29E-18
ABHD14B	mRNA	-2.07872	3.22E-20	1.31E-18
CLIP2	mRNA	3.086075	3.26E-20	1.33E-18
TMED3	mRNA	2.763615	3.27E-20	1.33E-18
TRPC5	mRNA	-4.84758	3.62E-20	1.46E-18
SEZ6L2	mRNA	5.05462	3.67E-20	1.48E-18
PC	mRNA	-3.66312	3.69E-20	1.49E-18
CYP26A1	mRNA	-5.77192	3.78E-20	1.52E-18
STK39	mRNA	3.465906	3.86E-20	1.55E-18
BHMT2	mRNA	-5.59943	3.93E-20	1.57E-18
LSS	mRNA	-2.18056	4.01E-20	1.60E-18
FCGRT	mRNA	-2.16347	4.35E-20	1.73E-18
CLEC1B	mRNA	-6.22655	4.54E-20	1.80E-18
MT-CYB	mRNA	-2.4001	4.78E-20	1.89E-18
AASS	mRNA	-3.62514	4.87E-20	1.93E-18
MKI67	mRNA	4.057146	5.16E-20	2.04E-18
HSD17B13	mRNA	-6.46046	5.45E-20	2.15E-18
FOXN4	mRNA	-6.08414	5.63E-20	2.21E-18
NUDT6	mRNA	-2.78037	6.54E-20	2.57E-18
CRHBP	mRNA	-5.4458	6.56E-20	2.57E-18
IDNK	mRNA	-2.54245	6.88E-20	2.68E-18
DTL	mRNA	4.228057	7.60E-20	2.96E-18
POF1B	mRNA	4.485876	7.87E-20	3.06E-18
NDST3	mRNA	-4.28786	7.92E-20	3.07E-18
ALAD	mRNA	-2.52674	8.24E-20	3.19E-18
ADRA1A	mRNA	-6.13222	8.51E-20	3.29E-18
EPCAM	mRNA	4.691618	8.88E-20	3.42E-18
GCSH	mRNA	-2.67273	9.38E-20	3.61E-18
NIPAL1	mRNA	-2.93964	1.05E-19	4.03E-18
ITGB4	mRNA	4.805099	1.08E-19	4.13E-18
CDO1	mRNA	-6.05654	1.10E-19	4.22E-18
HIST3H2A	mRNA	4.71412	1.18E-19	4.48E-18
PON1	mRNA	-5.71252	1.39E-19	5.28E-18
NPW	mRNA	-4.79288	1.43E-19	5.45E-18
1-Mar	mRNA	-2.91195	1.47E-19	5.59E-18
RNASE4	mRNA	-3.18635	1.52E-19	5.75E-18
SLC1A2	mRNA	-5.54287	1.58E-19	5.94E-18
CYP2B6	mRNA	-5.91491	1.59E-19	5.98E-18

CLYBL	mRNA	-2.54881	1.64E-19	6.16E-18
STARD4	mRNA	-2.39442	1.68E-19	6.27E-18
MT-ND2	mRNA	-2.25	1.68E-19	6.27E-18
RNF165	mRNA	-3.87974	1.71E-19	6.36E-18
SLC39A8	mRNA	-2.7397	1.73E-19	6.42E-18
NCAM2	mRNA	-4.52061	2.08E-19	7.72E-18
CCDC38	mRNA	-3.9875	2.12E-19	7.83E-18
GCHFR	mRNA	-2.86718	2.13E-19	7.86E-18
ITPR3	mRNA	3.572193	2.17E-19	7.99E-18
ALB	mRNA	-5.96777	2.22E-19	8.18E-18
PLPP2	mRNA	4.67312	2.33E-19	8.57E-18
ABHD6	mRNA	-3.74048	2.36E-19	8.63E-18
ASL	mRNA	-2.60032	2.44E-19	8.92E-18
DCAF11	mRNA	-2.05605	2.49E-19	9.07E-18
ZDHHC13	mRNA	2.536469	2.51E-19	9.14E-18
ADRB2	mRNA	-4.09188	2.70E-19	9.82E-18
GHR	mRNA	-4.34316	2.73E-19	9.88E-18
AK4	mRNA	-3.51584	2.93E-19	1.06E-17
BDH2	mRNA	-2.23907	2.97E-19	1.07E-17
CPS1	mRNA	-6.93178	3.04E-19	1.09E-17
MFSD6	mRNA	2.917053	3.06E-19	1.10E-17
PXDC1	mRNA	-2.38208	3.23E-19	1.16E-17
STARD5	mRNA	-2.9138	3.33E-19	1.19E-17
IDH1	mRNA	-2.23075	3.55E-19	1.27E-17
CYP2C19	mRNA	-5.81691	3.56E-19	1.27E-17
GPER1	mRNA	-3.47649	3.58E-19	1.27E-17
LAMC2	mRNA	5.43081	3.69E-19	1.31E-17
A1BG	mRNA	-6.06474	3.97E-19	1.40E-17
TBX15	mRNA	-4.47452	4.20E-19	1.48E-17
CHRNA4	mRNA	-6.88539	4.64E-19	1.63E-17
MT1A	mRNA	-4.20408	4.80E-19	1.68E-17
GPT2	mRNA	-3.60723	5.81E-19	2.03E-17
HPD	mRNA	-6.25071	5.89E-19	2.05E-17
ELOVL6	mRNA	-3.10243	5.91E-19	2.06E-17
IGFBP1	mRNA	-4.83245	6.17E-19	2.14E-17
SLC38A2	mRNA	-2.25986	6.85E-19	2.37E-17
GOLGA6A	mRNA	-4.58393	6.88E-19	2.38E-17
GCKR	mRNA	-3.71441	7.12E-19	2.46E-17
HLF	mRNA	-3.47556	8.25E-19	2.83E-17
CYP4F11	mRNA	-2.82315	8.35E-19	2.86E-17
RND3	mRNA	-2.62683	8.79E-19	3.01E-17
ECT2	mRNA	3.797073	8.81E-19	3.01E-17
GLYATL1	mRNA	-5.56926	9.06E-19	3.09E-17
CDHR5	mRNA	-4.47836	9.08E-19	3.09E-17
RHPN1	mRNA	3.713983	9.30E-19	3.15E-17
TMEM53	mRNA	-2.18375	9.67E-19	3.27E-17
AVPI1	mRNA	-2.14858	1.00E-18	3.38E-17

PCK1	mRNA	-6.32544	1.00E-18	3.38E-17
LRCOL1	mRNA	-5.14107	1.24E-18	4.16E-17
PIPOX	mRNA	-5.29026	1.26E-18	4.22E-17
BMPER	mRNA	-4.2164	1.26E-18	4.22E-17
STAB2	mRNA	-4.38385	1.39E-18	4.63E-17
CYP4A11	mRNA	-5.82968	1.39E-18	4.64E-17
RAB3D	mRNA	2.790943	1.52E-18	5.03E-17
AQP9	mRNA	-5.66161	1.66E-18	5.50E-17
CTBP2	mRNA	2.651065	1.67E-18	5.52E-17
SHBG	mRNA	-5.65091	1.68E-18	5.52E-17
KLC2	mRNA	2.12263	1.74E-18	5.71E-17
SIGMAR1	mRNA	-2.27464	1.92E-18	6.30E-17
C3orf85	mRNA	-6.6293	1.93E-18	6.33E-17
APOB	mRNA	-4.74303	2.03E-18	6.61E-17
SFXN2	mRNA	-2.1615	2.05E-18	6.68E-17
CYP2A7	mRNA	-8.13783	2.17E-18	7.03E-17
MT-ATP6	mRNA	-2.27494	2.24E-18	7.25E-17
CCNB2	mRNA	4.438325	2.26E-18	7.32E-17
UHRF1	mRNA	4.36499	2.37E-18	7.64E-17
SERPINF2	mRNA	-4.90697	2.41E-18	7.74E-17
SYPL2	mRNA	-3.67049	2.43E-18	7.81E-17
LPAR2	mRNA	3.323772	2.50E-18	8.00E-17
CYP4A22	mRNA	-6.29106	2.51E-18	8.02E-17
RNF125	mRNA	-3.53934	2.57E-18	8.22E-17
MT1H	mRNA	-6.28093	2.77E-18	8.82E-17
PZP	mRNA	-6.27376	2.77E-18	8.82E-17
LRG1	mRNA	-3.62875	2.86E-18	9.09E-17
ELL2	mRNA	-2.90946	3.03E-18	9.59E-17
PEPD	mRNA	-2.14574	3.04E-18	9.63E-17
CNTLN	mRNA	-2.14038	3.22E-18	1.02E-16
SLC16A10	mRNA	-3.76027	3.24E-18	1.02E-16
GADD45B	mRNA	-2.59408	3.40E-18	1.07E-16
GPD1	mRNA	-4.64901	3.48E-18	1.09E-16
CYP2C8	mRNA	-5.31687	3.61E-18	1.13E-16
F8	mRNA	-2.43341	3.78E-18	1.18E-16
CUX2	mRNA	-4.80402	3.88E-18	1.21E-16
SLC25A25	mRNA	-2.95705	4.39E-18	1.37E-16
CDCA5	mRNA	3.788496	4.41E-18	1.37E-16
CES3	mRNA	-4.16124	4.46E-18	1.39E-16
BAAT	mRNA	-4.11202	4.65E-18	1.44E-16
OIT3	mRNA	-5.26368	4.66E-18	1.44E-16
ADGRG1	mRNA	3.569339	4.69E-18	1.45E-16
OXT	mRNA	-5.56479	4.90E-18	1.51E-16
ECHDC2	mRNA	-2.36319	4.91E-18	1.51E-16
PPP2R1B	mRNA	-2.36897	5.40E-18	1.66E-16
GPRIN1	mRNA	4.352143	5.45E-18	1.67E-16
PAH	mRNA	-4.12163	6.15E-18	1.88E-16

MIP	mRNA	-3.64669	6.35E-18	1.94E-16
APOH	mRNA	-5.77732	6.82E-18	2.08E-16
ASGR1	mRNA	-4.61741	6.91E-18	2.11E-16
PXMP2	mRNA	-3.20815	7.75E-18	2.35E-16
FANCI	mRNA	2.876551	9.69E-18	2.94E-16
EXOC3L4	mRNA	-3.1039	1.00E-17	3.03E-16
PRC1	mRNA	4.021862	1.07E-17	3.22E-16
CPT2	mRNA	-2.10105	1.11E-17	3.35E-16
ACADL	mRNA	-3.93875	1.16E-17	3.50E-16
KIFC1	mRNA	3.917448	1.17E-17	3.51E-16
CECR2	mRNA	-4.62454	1.22E-17	3.67E-16
PAQR4	mRNA	3.754518	1.22E-17	3.67E-16
NAT1	mRNA	-2.11099	1.25E-17	3.73E-16
MAT1A	mRNA	-5.84971	1.34E-17	4.00E-16
ANGPTL6	mRNA	-4.39637	1.41E-17	4.19E-16
HAO2	mRNA	-7.0235	1.46E-17	4.33E-16
CENPM	mRNA	4.409606	1.50E-17	4.45E-16
METTL7B	mRNA	-2.85944	1.59E-17	4.69E-16
MFSD2A	mRNA	-4.4686	1.63E-17	4.80E-16
CENPF	mRNA	4.35815	1.70E-17	4.99E-16
NCAPH	mRNA	3.679954	1.74E-17	5.10E-16
ACBD4	mRNA	-2.04004	1.76E-17	5.17E-16
C8B	mRNA	-5.47752	1.84E-17	5.36E-16
FMO4	mRNA	-2.63986	1.89E-17	5.51E-16
BUB1	mRNA	4.24829	1.99E-17	5.78E-16
IGFBP4	mRNA	-2.52192	2.10E-17	6.11E-16
NEK2	mRNA	5.161693	2.26E-17	6.57E-16
B4GALNT4	mRNA	6.195507	2.27E-17	6.58E-16
CRAT	mRNA	-2.38213	2.31E-17	6.67E-16
SDC2	mRNA	-3.15122	2.34E-17	6.75E-16
ASCL1	mRNA	-6.2985	2.52E-17	7.27E-16
PLCD3	mRNA	3.949665	2.72E-17	7.81E-16
SAR1B	mRNA	-2.19222	2.75E-17	7.90E-16
ASPG	mRNA	-5.79	2.78E-17	7.94E-16
CDC42BPG	mRNA	3.083815	2.81E-17	8.02E-16
EPS8L3	mRNA	7.160121	2.84E-17	8.10E-16
RAB25	mRNA	5.323649	2.87E-17	8.16E-16
ANO9	mRNA	3.937915	2.93E-17	8.34E-16
CEP55	mRNA	4.911845	3.03E-17	8.61E-16
RXRA	mRNA	-2.24708	3.08E-17	8.74E-16
MMACHC	mRNA	-2.15126	3.21E-17	9.09E-16
MNX1	mRNA	7.688416	3.23E-17	9.13E-16
CFH	mRNA	-3.80527	3.38E-17	9.54E-16
CXorf66	mRNA	-5.3962	3.55E-17	9.98E-16
PLP2	mRNA	3.206384	3.59E-17	1.01E-15
GRIA3	mRNA	-3.54743	3.59E-17	1.01E-15
MELK	mRNA	4.317365	3.69E-17	1.03E-15

2-Mar	mRNA	-2.38541	3.76E-17	1.05E-15
KLF12	mRNA	-2.23493	3.84E-17	1.07E-15
PALMD	mRNA	-3.10135	3.91E-17	1.09E-15
GOLGA6B	mRNA	-4.15771	4.08E-17	1.13E-15
ALDH4A1	mRNA	-3.3724	4.52E-17	1.25E-15
USH1C	mRNA	5.388141	4.61E-17	1.28E-15
ACY3	mRNA	-2.78168	4.63E-17	1.28E-15
PCSK6	mRNA	-3.50465	4.80E-17	1.33E-15
SPATS2	mRNA	2.270639	4.86E-17	1.34E-15
CEBPD	mRNA	-2.16199	5.09E-17	1.40E-15
PDXP	mRNA	-2.79965	5.19E-17	1.43E-15
FGG	mRNA	-4.88419	5.20E-17	1.43E-15
FOXM1	mRNA	3.977341	5.36E-17	1.47E-15
GAMT	mRNA	-3.29058	5.39E-17	1.47E-15
FAM69A	mRNA	-2.44554	5.43E-17	1.48E-15
RD3L	mRNA	-5.01017	5.56E-17	1.52E-15
BOK	mRNA	-2.24536	5.60E-17	1.52E-15
ETFBKMT	mRNA	-2.04559	6.05E-17	1.64E-15
PKMYT1	mRNA	3.703953	6.39E-17	1.73E-15
MYBL2	mRNA	4.841168	6.39E-17	1.73E-15
AL136295.3	mRNA	-3.56253	6.41E-17	1.74E-15
EPHA10	mRNA	6.880795	6.50E-17	1.76E-15
SLC16A1	mRNA	-2.79074	6.82E-17	1.84E-15
SIPA1L3	mRNA	2.233669	6.86E-17	1.84E-15
HYAL1	mRNA	-2.87494	7.13E-17	1.91E-15
MTTP	mRNA	-6.40781	7.20E-17	1.93E-15
SYT7	mRNA	-4.05339	7.45E-17	1.99E-15
CA5A	mRNA	-5.82372	7.45E-17	1.99E-15
ABCA10	mRNA	-3.26146	7.58E-17	2.02E-15
BPHL	mRNA	-2.24279	8.14E-17	2.17E-15
TOP2A	mRNA	4.393856	8.63E-17	2.30E-15
DBN1	mRNA	3.768877	9.99E-17	2.65E-15
GYS2	mRNA	-6.69521	1.03E-16	2.72E-15
TRO	mRNA	-2.72644	1.04E-16	2.75E-15
FXN	mRNA	-2.05853	1.05E-16	2.76E-15
E2F1	mRNA	3.691109	1.07E-16	2.81E-15
CCNB1	mRNA	3.271275	1.08E-16	2.84E-15
DMD	mRNA	-2.90962	1.10E-16	2.87E-15
AR	mRNA	-4.1517	1.14E-16	2.99E-15
ASNS	mRNA	3.41401	1.16E-16	3.02E-15
TMEM252	mRNA	-4.76484	1.17E-16	3.05E-15
HCN3	mRNA	-2.57102	1.19E-16	3.11E-15
ITIH3	mRNA	-5.10485	1.21E-16	3.14E-15
ADH1A	mRNA	-6.17029	1.21E-16	3.15E-15
UGT2B10	mRNA	-7.11286	1.25E-16	3.23E-15
BAIAP2L2	mRNA	4.673409	1.26E-16	3.26E-15
UGT2B7	mRNA	-5.29534	1.32E-16	3.43E-15

PRG4	mRNA	-5.34928	1.34E-16	3.46E-15
ENTPD5	mRNA	-2.98939	1.44E-16	3.72E-15
TRIM59	mRNA	3.268208	1.52E-16	3.90E-15
DIAPH3	mRNA	4.33951	1.52E-16	3.90E-15
IQGAP3	mRNA	4.883219	1.67E-16	4.27E-15
IGFALS	mRNA	-3.78938	1.77E-16	4.54E-15
FAM198A	mRNA	-3.65574	1.79E-16	4.57E-15
PER3	mRNA	-2.71725	1.86E-16	4.74E-15
MCM2	mRNA	2.526891	1.93E-16	4.92E-15
FGB	mRNA	-5.45583	1.95E-16	4.95E-15
PALM2	mRNA	-3.31046	2.03E-16	5.14E-15
IGFBP2	mRNA	-3.97386	2.06E-16	5.22E-15
MOGAT2	mRNA	-6.62213	2.07E-16	5.25E-15
SPECC1	mRNA	3.671517	2.26E-16	5.71E-15
NR1I2	mRNA	-5.36114	2.27E-16	5.72E-15
UBE2C	mRNA	4.734196	2.31E-16	5.82E-15
SDS	mRNA	-5.25132	2.44E-16	6.14E-15
SLC22A1	mRNA	-6.29762	2.46E-16	6.16E-15
UNC93A	mRNA	-5.06697	2.47E-16	6.18E-15
HAL	mRNA	-5.33646	2.56E-16	6.39E-15
SLC17A2	mRNA	-4.94108	2.74E-16	6.82E-15
PFKFB1	mRNA	-4.80505	2.83E-16	7.04E-15
LGSN	mRNA	-4.96383	3.03E-16	7.52E-15
SLC9B2	mRNA	-3.43789	3.05E-16	7.57E-15
SHCBP1	mRNA	4.198422	3.12E-16	7.72E-15
ANLN	mRNA	4.536993	3.13E-16	7.74E-15
NCAPG	mRNA	4.132466	3.30E-16	8.14E-15
MLXIPL	mRNA	-3.54242	3.37E-16	8.26E-15
NUGGC	mRNA	-4.60856	3.50E-16	8.59E-15
CYP2E1	mRNA	-6.19685	3.66E-16	8.96E-15
KIF2C	mRNA	4.242174	3.97E-16	9.70E-15
C8A	mRNA	-5.62372	4.06E-16	9.92E-15
PDK4	mRNA	-3.70149	4.11E-16	1.00E-14
DPYS	mRNA	-6.36908	5.13E-16	1.24E-14
TSPAN15	mRNA	3.959912	5.16E-16	1.25E-14
KIF18B	mRNA	4.851307	5.18E-16	1.25E-14
CFP	mRNA	-3.51859	5.43E-16	1.31E-14
HMGCS1	mRNA	-2.55947	5.52E-16	1.33E-14
PFKP	mRNA	4.319146	5.99E-16	1.44E-14
DPYD	mRNA	-2.71474	6.11E-16	1.47E-14
PSORS1C1	mRNA	5.396336	6.14E-16	1.47E-14
ABCB11	mRNA	-5.5654	6.14E-16	1.47E-14
REEP6	mRNA	-3.01911	6.48E-16	1.55E-14
GPR182	mRNA	-3.56709	6.92E-16	1.65E-14
MGST2	mRNA	-2.04016	7.03E-16	1.68E-14
AOX1	mRNA	-4.89758	7.45E-16	1.77E-14
FADS1	mRNA	-2.98978	7.51E-16	1.79E-14

INAVA	mRNA	4.879587	7.58E-16	1.80E-14
PSAT1	mRNA	-3.87425	8.25E-16	1.95E-14
TAS2R60	mRNA	-5.27053	8.25E-16	1.95E-14
BUB1B	mRNA	4.273991	8.33E-16	1.96E-14
SAT2	mRNA	-2.0292	8.59E-16	2.02E-14
PROC	mRNA	-4.11333	9.48E-16	2.23E-14
SLC38A3	mRNA	-4.86599	9.72E-16	2.28E-14
KIF23	mRNA	3.920314	9.87E-16	2.31E-14
COBLL1	mRNA	-2.50971	9.98E-16	2.33E-14
AL163636.2	mRNA	-2.86976	1.02E-15	2.37E-14
HSD11B1	mRNA	-4.30176	1.02E-15	2.38E-14
PLGLB2	mRNA	-4.48538	1.03E-15	2.39E-14
CFHR3	mRNA	-6.09655	1.03E-15	2.39E-14
C2	mRNA	-3.97598	1.05E-15	2.45E-14
TBX3	mRNA	-2.94734	1.08E-15	2.51E-14
COL6A6	mRNA	-3.54175	1.09E-15	2.52E-14
CLEC4G	mRNA	-5.91439	1.14E-15	2.64E-14
GCLC	mRNA	-2.46977	1.14E-15	2.64E-14
PCNX2	mRNA	3.890769	1.16E-15	2.67E-14
DNASE1L3	mRNA	-4.45407	1.24E-15	2.86E-14
LIFR	mRNA	-2.81579	1.26E-15	2.89E-14
CDK1	mRNA	3.903759	1.30E-15	2.97E-14
TCEA3	mRNA	-2.54759	1.31E-15	3.00E-14
TEX30	mRNA	-2.05076	1.37E-15	3.12E-14
DLGAP5	mRNA	4.454357	1.38E-15	3.16E-14
ELF4	mRNA	2.680002	1.46E-15	3.32E-14
SERPINA1	mRNA	-3.40312	1.48E-15	3.37E-14
CDH23	mRNA	-3.18274	1.54E-15	3.49E-14
AMBP	mRNA	-4.70317	1.55E-15	3.51E-14
LILRB5	mRNA	-3.54958	1.62E-15	3.67E-14
HRG	mRNA	-6.17594	1.62E-15	3.67E-14
CHAF1B	mRNA	3.078109	1.71E-15	3.85E-14
RIPPLY1	mRNA	-3.92876	1.97E-15	4.42E-14
FCN2	mRNA	-6.45654	2.14E-15	4.79E-14
GCGR	mRNA	-6.04987	2.22E-15	4.97E-14
LEPR	mRNA	-3.4857	2.23E-15	4.98E-14
EFHD1	mRNA	-2.8896	2.28E-15	5.09E-14
LY6E	mRNA	-2.09232	2.32E-15	5.18E-14
DDR1	mRNA	3.7333	2.35E-15	5.23E-14
PEX11G	mRNA	-2.18821	2.41E-15	5.35E-14
MCM3	mRNA	2.040447	2.41E-15	5.35E-14
ASF1B	mRNA	3.68477	2.42E-15	5.38E-14
TFR2	mRNA	-4.30744	2.43E-15	5.39E-14
MARCO	mRNA	-4.25758	2.52E-15	5.58E-14
CYP8B1	mRNA	-6.61827	2.66E-15	5.87E-14
CCDC112	mRNA	2.462025	2.85E-15	6.28E-14
SLC28A1	mRNA	-3.55849	2.87E-15	6.32E-14

RFPL1	mRNA	-5.3636	2.90E-15	6.38E-14
ERRFI1	mRNA	-2.81083	2.92E-15	6.41E-14
CCDC152	mRNA	-3.01563	2.98E-15	6.53E-14
TMEM170B	mRNA	-2.4736	3.01E-15	6.58E-14
OXER1	mRNA	-2.84639	3.06E-15	6.69E-14
ORAI2	mRNA	2.744239	3.08E-15	6.71E-14
GNPNAT1	mRNA	-2.22403	3.11E-15	6.78E-14
GGH	mRNA	-2.59909	3.29E-15	7.14E-14
EPM2A	mRNA	-2.05322	3.81E-15	8.25E-14
CDC6	mRNA	3.507914	4.08E-15	8.83E-14
SCUBE1	mRNA	-4.17131	4.14E-15	8.92E-14
CDC20	mRNA	4.67824	4.15E-15	8.95E-14
CDC45	mRNA	3.993416	4.40E-15	9.45E-14
ANGPTL3	mRNA	-5.75999	4.44E-15	9.53E-14
ABCA3	mRNA	3.099006	4.64E-15	9.93E-14
ARFGEF3	mRNA	4.118637	4.66E-15	9.96E-14
MASP1	mRNA	-4.05128	4.86E-15	1.04E-13
SLC22A10	mRNA	-6.28637	5.06E-15	1.08E-13
EXO1	mRNA	4.141609	5.19E-15	1.10E-13
VNN3	mRNA	-3.99986	5.19E-15	1.10E-13
C6orf132	mRNA	3.805158	5.27E-15	1.12E-13
TCF19	mRNA	2.749206	5.28E-15	1.12E-13
RAB36	mRNA	3.974049	5.33E-15	1.13E-13
PHLDA3	mRNA	2.922705	5.50E-15	1.16E-13
MT-ND4	mRNA	-2.22534	5.57E-15	1.18E-13
KIF18A	mRNA	3.91012	5.60E-15	1.18E-13
ABCA5	mRNA	-2.07938	5.71E-15	1.20E-13
CHPT1	mRNA	-2.21016	5.72E-15	1.20E-13
CDNF	mRNA	-2.1712	5.99E-15	1.26E-13
PLK1	mRNA	4.185812	6.11E-15	1.28E-13
UPP2	mRNA	-4.44236	6.19E-15	1.30E-13
S100A11	mRNA	3.608467	6.32E-15	1.32E-13
GREM2	mRNA	-5.90706	6.36E-15	1.33E-13
MOCS1	mRNA	-2.23096	6.79E-15	1.42E-13
GALK1	mRNA	-2.18149	6.87E-15	1.43E-13
MMP11	mRNA	5.463673	6.93E-15	1.44E-13
AKR1D1	mRNA	-6.75447	6.94E-15	1.44E-13
ABCG5	mRNA	-4.68252	6.94E-15	1.44E-13
ADAMTS17	mRNA	-3.39626	7.55E-15	1.56E-13
MCM10	mRNA	3.690032	7.57E-15	1.57E-13
C4BPA	mRNA	-4.26228	7.69E-15	1.59E-13
ABHD1	mRNA	-2.98931	7.79E-15	1.61E-13
CSRNP1	mRNA	-2.29136	7.93E-15	1.63E-13
PINK1	mRNA	-2.14859	8.00E-15	1.65E-13
MAMDC4	mRNA	-2.8028	8.28E-15	1.70E-13
MGAT5	mRNA	2.939927	8.30E-15	1.71E-13
VIPR1	mRNA	-3.51008	8.61E-15	1.77E-13

HAMP	mRNA	-5.24175	8.72E-15	1.79E-13
PAFAH1B3	mRNA	2.900319	8.80E-15	1.80E-13
SLC38A1	mRNA	3.188225	8.91E-15	1.82E-13
GBP7	mRNA	-5.80057	9.10E-15	1.86E-13
SLITRK3	mRNA	-6.92821	9.13E-15	1.86E-13
ACOT6	mRNA	-3.65343	9.42E-15	1.92E-13
PPP1R3G	mRNA	-3.48079	1.00E-14	2.04E-13
STIL	mRNA	3.281182	1.05E-14	2.13E-13
ACSM2B	mRNA	-5.35074	1.16E-14	2.34E-13
ILDR1	mRNA	3.633744	1.16E-14	2.35E-13
TCTN2	mRNA	3.054612	1.17E-14	2.36E-13
ID2	mRNA	-2.04473	1.24E-14	2.49E-13
FA2H	mRNA	4.965255	1.25E-14	2.52E-13
ABCA1	mRNA	-2.7928	1.35E-14	2.72E-13
RAPGEF4	mRNA	-2.90357	1.39E-14	2.79E-13
RACGAP1	mRNA	2.717575	1.39E-14	2.79E-13
NCAPD2	mRNA	2.138716	1.41E-14	2.82E-13
MMP14	mRNA	2.773891	1.41E-14	2.82E-13
ETNPPL	mRNA	-6.51408	1.42E-14	2.85E-13
SLC52A3	mRNA	5.000201	1.43E-14	2.86E-13
CD24	mRNA	3.56831	1.53E-14	3.06E-13
TERB2	mRNA	-5.34184	1.54E-14	3.07E-13
RAD54L	mRNA	4.586234	1.60E-14	3.18E-13
FTCD	mRNA	-4.78137	1.61E-14	3.19E-13
PLPP3	mRNA	-2.41483	1.71E-14	3.40E-13
KIF4A	mRNA	4.031328	1.85E-14	3.66E-13
FAM111B	mRNA	3.55373	1.97E-14	3.90E-13
TES	mRNA	2.155664	1.99E-14	3.93E-13
UAP1	mRNA	-2.00244	2.01E-14	3.97E-13
SEL1L3	mRNA	3.197898	2.03E-14	4.01E-13
APOF	mRNA	-6.84462	2.14E-14	4.20E-13
OSBP2	mRNA	2.645535	2.16E-14	4.24E-13
BIRC5	mRNA	4.052483	2.17E-14	4.25E-13
TFAP2A	mRNA	6.41981	2.19E-14	4.28E-13
HDAC7	mRNA	2.373045	2.27E-14	4.45E-13
FTL	mRNA	-2.23206	2.45E-14	4.78E-13
FMO3	mRNA	-4.89572	2.49E-14	4.86E-13
WNK3	mRNA	-3.54085	2.52E-14	4.90E-13
ELFN1	mRNA	-3.36983	2.54E-14	4.94E-13
CYP2A6	mRNA	-6.98252	2.56E-14	4.97E-13
C8orf46	mRNA	-3.02625	2.66E-14	5.15E-13
PIK3R1	mRNA	-2.18447	2.89E-14	5.58E-13
CMTM4	mRNA	2.683799	3.01E-14	5.81E-13
DMKN	mRNA	4.68387	3.08E-14	5.93E-13
CYP2C18	mRNA	-3.33673	3.24E-14	6.23E-13
FAM81A	mRNA	4.556	3.25E-14	6.25E-13
ASPA	mRNA	-3.34056	3.40E-14	6.53E-13

UBE2T	mRNA	3.575456	3.40E-14	6.53E-13
PLXNA1	mRNA	2.327384	3.45E-14	6.62E-13
KLF15	mRNA	-3.16017	3.49E-14	6.68E-13
SRD5A1	mRNA	-2.07932	3.61E-14	6.89E-13
ABR	mRNA	2.218938	3.68E-14	7.02E-13
SLCO1B7	mRNA	-4.52948	3.68E-14	7.02E-13
FABP1	mRNA	-6.00146	3.85E-14	7.32E-13
FSCN2	mRNA	4.271736	3.88E-14	7.36E-13
BMP10	mRNA	-5.50263	3.92E-14	7.44E-13
BHMT	mRNA	-6.45511	3.94E-14	7.47E-13
CBR1	mRNA	-2.08466	4.18E-14	7.91E-13
IQANK1	mRNA	4.872284	4.20E-14	7.94E-13
CES1	mRNA	-4.9464	4.43E-14	8.36E-13
PNPLA7	mRNA	-2.01225	4.51E-14	8.51E-13
PAPSS2	mRNA	-2.65641	4.73E-14	8.91E-13
PTPN14	mRNA	3.074722	4.83E-14	9.08E-13
B3GNT3	mRNA	4.531015	4.88E-14	9.16E-13
TTC39A	mRNA	5.032279	4.97E-14	9.32E-13
ACO1	mRNA	-2.22627	5.00E-14	9.37E-13
CYP4F3	mRNA	-4.23275	5.01E-14	9.38E-13
MFSD10	mRNA	2.647797	5.03E-14	9.40E-13
TANC2	mRNA	2.809924	5.03E-14	9.40E-13
E2F8	mRNA	4.454388	5.29E-14	9.88E-13
EPS8L1	mRNA	4.169016	5.31E-14	9.91E-13
PRODH2	mRNA	-5.42694	5.35E-14	9.95E-13
ST3GAL6	mRNA	-3.16757	5.46E-14	1.02E-12
ZG16	mRNA	-6.87128	5.81E-14	1.08E-12
GPR35	mRNA	5.066198	5.91E-14	1.10E-12
ARPP21	mRNA	-4.6581	5.98E-14	1.11E-12
AGMO	mRNA	-3.96907	6.08E-14	1.12E-12
P2RX3	mRNA	-3.54807	6.17E-14	1.14E-12
ORM2	mRNA	-4.90593	6.19E-14	1.14E-12
CENPA	mRNA	4.522304	6.52E-14	1.20E-12
CHRD	mRNA	-2.83876	6.85E-14	1.26E-12
SLC27A2	mRNA	-3.91374	6.99E-14	1.28E-12
ADRA1B	mRNA	-3.02978	7.19E-14	1.31E-12
CYP7A1	mRNA	-6.72731	7.39E-14	1.35E-12
DAO	mRNA	-5.92139	7.53E-14	1.37E-12
SLC25A24	mRNA	2.577561	7.60E-14	1.38E-12
HSPB9	mRNA	-2.64063	7.61E-14	1.38E-12
RGPD3	mRNA	-3.22586	7.90E-14	1.43E-12
LIPG	mRNA	-3.25974	8.03E-14	1.46E-12
KIF20A	mRNA	3.905122	8.05E-14	1.46E-12
CPB2	mRNA	-3.98626	8.08E-14	1.46E-12
TTC39C	mRNA	-2.46438	8.12E-14	1.47E-12
SNTB1	mRNA	-2.37612	8.23E-14	1.49E-12
CUEDC1	mRNA	2.263005	9.06E-14	1.64E-12

FYB2	mRNA	-4.03705	9.09E-14	1.64E-12
SULT1A1	mRNA	-2.24138	9.24E-14	1.66E-12
RUSC1	mRNA	2.111327	9.83E-14	1.77E-12
AC022335.1	mRNA	-3.99019	1.05E-13	1.89E-12
LMNB2	mRNA	2.248563	1.05E-13	1.89E-12
PLXNA3	mRNA	2.313867	1.05E-13	1.89E-12
ALDH1A1	mRNA	-3.08767	1.09E-13	1.94E-12
SERPINA11	mRNA	-6.0876	1.09E-13	1.94E-12
PHLDA1	mRNA	-2.34827	1.14E-13	2.03E-12
TESMIN	mRNA	4.2393	1.15E-13	2.04E-12
MELTF	mRNA	4.36879	1.16E-13	2.07E-12
JADE2	mRNA	2.016044	1.17E-13	2.07E-12
PMEPA1	mRNA	4.525015	1.24E-13	2.20E-12
GLYAT	mRNA	-6.70637	1.25E-13	2.21E-12
SLC39A10	mRNA	2.295786	1.29E-13	2.29E-12
GPLD1	mRNA	-3.92568	1.32E-13	2.32E-12
COQ10A	mRNA	-2.01754	1.35E-13	2.39E-12
SLC22A7	mRNA	-5.86232	1.41E-13	2.48E-12
MT-ND3	mRNA	-2.15956	1.47E-13	2.58E-12
HPX	mRNA	-5.77999	1.56E-13	2.73E-12
UPB1	mRNA	-5.56691	1.59E-13	2.78E-12
PERM1	mRNA	3.456568	1.61E-13	2.82E-12
SCRN1	mRNA	3.335111	1.66E-13	2.90E-12
CFB	mRNA	-2.84833	1.67E-13	2.91E-12
EDNRB	mRNA	-2.39489	1.69E-13	2.94E-12
UGT1A4	mRNA	-6.50422	1.70E-13	2.95E-12
KCNE1	mRNA	-3.27031	1.73E-13	3.00E-12
ARMC9	mRNA	2.606727	1.89E-13	3.28E-12
RRM2	mRNA	3.557049	2.02E-13	3.49E-12
RHOV	mRNA	5.720985	2.02E-13	3.49E-12
ABCA9	mRNA	-3.307	2.04E-13	3.51E-12
FAM9B	mRNA	-4.0282	2.04E-13	3.52E-12
TEAD4	mRNA	2.521257	2.08E-13	3.59E-12
PKLR	mRNA	-4.88069	2.09E-13	3.59E-12
B4GALNT2	mRNA	10.61656	2.10E-13	3.62E-12
PCLAF	mRNA	3.146353	2.16E-13	3.71E-12
FAM155B	mRNA	4.770082	2.17E-13	3.73E-12
ZCCHC24	mRNA	-2.04728	2.19E-13	3.76E-12
STBD1	mRNA	-2.1067	2.23E-13	3.82E-12
C12orf75	mRNA	4.927217	2.25E-13	3.85E-12
PLEKHB1	mRNA	4.054689	2.25E-13	3.85E-12
FAM189B	mRNA	2.054071	2.26E-13	3.85E-12
GALM	mRNA	-2.17531	2.35E-13	4.01E-12
MST1R	mRNA	4.666726	2.38E-13	4.06E-12
OVOL2	mRNA	4.528354	2.40E-13	4.08E-12
GK	mRNA	-2.04414	2.42E-13	4.11E-12
CCL23	mRNA	-3.34866	2.43E-13	4.13E-12

ABCB4	mRNA	-4.65113	2.46E-13	4.17E-12
PLIN4	mRNA	-3.12489	2.62E-13	4.44E-12
C4A	mRNA	-3.3119	2.64E-13	4.47E-12
RAD51	mRNA	3.368011	2.69E-13	4.54E-12
NECAB2	mRNA	-4.27105	2.78E-13	4.68E-12
SKA1	mRNA	4.470545	2.86E-13	4.82E-12
MPPED1	mRNA	-5.42409	2.88E-13	4.84E-12
FMC1	mRNA	-2.03869	2.88E-13	4.84E-12
SLC4A3	mRNA	5.636276	3.00E-13	5.03E-12
CHN2	mRNA	-2.90673	3.00E-13	5.04E-12
TRIP10	mRNA	2.084864	3.13E-13	5.24E-12
F2	mRNA	-5.5769	3.18E-13	5.32E-12
RBP4	mRNA	-5.23413	3.21E-13	5.36E-12
C14orf180	mRNA	-4.58576	3.25E-13	5.42E-12
PTP4A3	mRNA	2.502972	3.25E-13	5.42E-12
NCK2	mRNA	2.358942	3.27E-13	5.44E-12
GOLGA7B	mRNA	4.08743	3.29E-13	5.47E-12
FLVCR2	mRNA	-2.37438	3.29E-13	5.47E-12
ANKS6	mRNA	2.021808	3.30E-13	5.48E-12
LAMB3	mRNA	3.868889	3.40E-13	5.63E-12
C3orf52	mRNA	3.913279	3.53E-13	5.84E-12
SAA4	mRNA	-5.61201	3.67E-13	6.06E-12
SKA3	mRNA	4.102758	3.76E-13	6.20E-12
CDR2L	mRNA	3.201454	3.95E-13	6.51E-12
CTHRC1	mRNA	4.705829	4.01E-13	6.60E-12
LAMA5	mRNA	2.296453	4.12E-13	6.77E-12
HSD17B6	mRNA	-5.59493	4.12E-13	6.77E-12
ESPN	mRNA	-2.95696	4.19E-13	6.87E-12
ALDOC	mRNA	-2.6774	4.29E-13	7.04E-12
SLC51A	mRNA	-3.53599	4.35E-13	7.13E-12
SPC25	mRNA	3.680145	4.37E-13	7.15E-12
C11orf80	mRNA	2.64155	4.40E-13	7.19E-12
SINHCAF	mRNA	2.280784	4.43E-13	7.23E-12
COPZ2	mRNA	-2.16444	4.49E-13	7.34E-12
PPP1R9A	mRNA	2.792368	4.51E-13	7.36E-12
P3H4	mRNA	2.453203	4.71E-13	7.67E-12
RHBDF1	mRNA	2.319111	4.75E-13	7.74E-12
KIF11	mRNA	2.79592	4.77E-13	7.75E-12
INHBC	mRNA	-4.83477	4.84E-13	7.86E-12
AHSG	mRNA	-6.20215	4.87E-13	7.91E-12
SLC38A4	mRNA	-5.96142	4.90E-13	7.95E-12
GINS1	mRNA	2.795034	5.01E-13	8.10E-12
PEX3	mRNA	-2.04999	5.16E-13	8.34E-12
SMLR1	mRNA	-4.03478	5.19E-13	8.39E-12
ENPP5	mRNA	3.190037	5.21E-13	8.42E-12
GABBR1	mRNA	2.894754	5.29E-13	8.52E-12
SLC13A3	mRNA	-3.58054	5.33E-13	8.58E-12

SAPCD2	mRNA	3.893894	5.41E-13	8.71E-12
VSNL1	mRNA	-4.40803	5.63E-13	9.06E-12
RMI2	mRNA	2.954015	5.64E-13	9.07E-12
LIMK1	mRNA	2.441355	5.76E-13	9.24E-12
CD4	mRNA	-2.3141	5.79E-13	9.29E-12
N4BP3	mRNA	3.516029	5.96E-13	9.56E-12
SLC41A2	mRNA	-2.13125	6.08E-13	9.74E-12
CDCA2	mRNA	4.101298	6.10E-13	9.75E-12
TEAD2	mRNA	2.789964	6.19E-13	9.89E-12
NNMT	mRNA	-3.16435	6.20E-13	9.90E-12
SRC	mRNA	2.105557	6.21E-13	9.91E-12
AL645922.1	mRNA	-3.96878	6.32E-13	1.01E-11
AACS	mRNA	2.141955	6.53E-13	1.04E-11
CES2	mRNA	-3.0269	6.67E-13	1.06E-11
EHF	mRNA	4.164951	6.77E-13	1.07E-11
NDRG2	mRNA	-2.26338	6.80E-13	1.08E-11
THRB	mRNA	-2.73098	6.92E-13	1.09E-11
SGPP2	mRNA	4.918213	6.99E-13	1.10E-11
ADRB1	mRNA	-3.8321	7.02E-13	1.11E-11
SERPINA12	mRNA	-4.31253	7.04E-13	1.11E-11
MLIP	mRNA	-4.72387	7.07E-13	1.11E-11
EZH2	mRNA	2.425068	7.08E-13	1.11E-11
CETP	mRNA	-3.0602	7.09E-13	1.12E-11
MT-CO1	mRNA	-2.14715	7.64E-13	1.20E-11
NRG4	mRNA	-2.41741	7.73E-13	1.21E-11
FBLIM1	mRNA	2.884591	8.22E-13	1.29E-11
CPN1	mRNA	-4.52296	8.33E-13	1.30E-11
NAMPT	mRNA	-2.16717	8.33E-13	1.30E-11
ST14	mRNA	2.632756	8.35E-13	1.30E-11
S100A6	mRNA	4.62072	8.49E-13	1.32E-11
TMEM163	mRNA	4.675452	8.51E-13	1.32E-11
SLC13A5	mRNA	-5.47927	8.55E-13	1.33E-11
ANXA3	mRNA	3.543845	8.56E-13	1.33E-11
PRR36	mRNA	5.681911	8.57E-13	1.33E-11
HDGFL3	mRNA	2.834203	8.65E-13	1.34E-11
RNF144B	mRNA	-2.01385	8.69E-13	1.35E-11
CDCP1	mRNA	4.317797	9.47E-13	1.46E-11
CHD3	mRNA	2.772173	9.48E-13	1.46E-11
TNFAIP6	mRNA	6.381124	9.57E-13	1.48E-11
DDC	mRNA	-3.90326	9.70E-13	1.49E-11
SIGLEC11	mRNA	-3.2271	9.73E-13	1.50E-11
KIAA1522	mRNA	2.749907	9.89E-13	1.52E-11
A1CF	mRNA	-3.23586	9.92E-13	1.52E-11
G0S2	mRNA	-3.42091	1.01E-12	1.55E-11
ASPHD1	mRNA	4.813456	1.04E-12	1.59E-11
SERPINA6	mRNA	-3.15099	1.05E-12	1.61E-11
SELENBP1	mRNA	-3.09658	1.05E-12	1.61E-11

TMC4	mRNA	3.625591	1.06E-12	1.62E-11
SERPIND1	mRNA	-5.48127	1.10E-12	1.68E-11
UGT1A3	mRNA	-4.63573	1.11E-12	1.69E-11
SLC25A33	mRNA	-2.15048	1.13E-12	1.73E-11
ITIH5	mRNA	5.736121	1.17E-12	1.78E-11
RORA	mRNA	-2.23337	1.18E-12	1.79E-11
CDCA8	mRNA	3.559326	1.20E-12	1.83E-11
CLEC4M	mRNA	-6.1986	1.24E-12	1.88E-11
TROAP	mRNA	4.003076	1.25E-12	1.89E-11
ADAM9	mRNA	2.618666	1.27E-12	1.92E-11
ARG1	mRNA	-5.767	1.30E-12	1.96E-11
TDO2	mRNA	-4.73423	1.31E-12	1.98E-11
ABCC1	mRNA	2.623175	1.32E-12	1.99E-11
MISP	mRNA	6.452116	1.33E-12	2.01E-11
SLC10A1	mRNA	-5.99168	1.34E-12	2.02E-11
OSBPL3	mRNA	2.97483	1.38E-12	2.07E-11
SLC17A8	mRNA	-3.83542	1.40E-12	2.11E-11
SLC52A2	mRNA	2.004754	1.40E-12	2.11E-11
DLX4	mRNA	5.353829	1.45E-12	2.17E-11
RIBC2	mRNA	3.983565	1.45E-12	2.17E-11
ALLC	mRNA	-2.91637	1.49E-12	2.22E-11
COL26A1	mRNA	-3.18875	1.52E-12	2.26E-11
ACVR1C	mRNA	-3.05382	1.53E-12	2.29E-11
FRRS1	mRNA	-2.64681	1.54E-12	2.30E-11
RHBDF2	mRNA	2.533357	1.55E-12	2.30E-11
COX6A2	mRNA	-4.25709	1.55E-12	2.31E-11
FASN	mRNA	-2.16071	1.60E-12	2.38E-11
GTSE1	mRNA	3.972557	1.61E-12	2.39E-11
GLB1L2	mRNA	3.215186	1.65E-12	2.44E-11
SPEG	mRNA	4.607929	1.67E-12	2.47E-11
DDO	mRNA	-2.23058	1.71E-12	2.53E-11
RTL4	mRNA	-4.90446	1.72E-12	2.54E-11
UGT3A1	mRNA	-5.34151	1.74E-12	2.56E-11
DEPDC1	mRNA	4.557344	1.80E-12	2.65E-11
AFM	mRNA	-6.08488	1.82E-12	2.68E-11
PIMREG	mRNA	4.353914	1.84E-12	2.70E-11
GULP1	mRNA	4.473129	1.87E-12	2.74E-11
AURKB	mRNA	4.051533	1.88E-12	2.75E-11
SLC4A1	mRNA	-4.20576	1.90E-12	2.79E-11
PACS1	mRNA	2.018019	1.92E-12	2.81E-11
FBXL2	mRNA	2.690971	2.00E-12	2.93E-11
KAT2B	mRNA	-2.22625	2.01E-12	2.94E-11
ADH4	mRNA	-6.80126	2.05E-12	2.99E-11
LPCAT4	mRNA	2.588016	2.08E-12	3.04E-11
PTTG1	mRNA	3.860496	2.09E-12	3.04E-11
TST	mRNA	-2.20627	2.14E-12	3.12E-11
HMGN5	mRNA	-2.17801	2.17E-12	3.14E-11

NAT8	mRNA	-3.63981	2.17E-12	3.15E-11
PLEKHN1	mRNA	3.503265	2.31E-12	3.34E-11
VTN	mRNA	-3.33701	2.40E-12	3.47E-11
SFN	mRNA	5.062217	2.47E-12	3.58E-11
ORC1	mRNA	3.455092	2.48E-12	3.58E-11
ACOT4	mRNA	-2.42263	2.50E-12	3.62E-11
CKAP2L	mRNA	3.649717	2.52E-12	3.63E-11
SERPINA10	mRNA	-4.44527	2.56E-12	3.69E-11
HNF4A	mRNA	-2.34676	2.77E-12	3.99E-11
BCAN	mRNA	5.369878	2.96E-12	4.25E-11
LHX2	mRNA	-2.65474	3.17E-12	4.54E-11
LAD1	mRNA	2.980468	3.17E-12	4.54E-11
OTC	mRNA	-5.3918	3.25E-12	4.65E-11
C2orf50	mRNA	5.255172	3.27E-12	4.68E-11
CCL16	mRNA	-5.55203	3.29E-12	4.69E-11
CCNA2	mRNA	3.352352	3.30E-12	4.70E-11
DENND6B	mRNA	2.591882	3.35E-12	4.77E-11
MT-ND4L	mRNA	-2.45487	3.37E-12	4.79E-11
MT-ATP8	mRNA	-2.2988	3.39E-12	4.83E-11
SLC44A2	mRNA	2.316126	3.43E-12	4.87E-11
XRCC2	mRNA	3.301283	3.48E-12	4.94E-11
TLDC2	mRNA	4.044004	3.54E-12	5.02E-11
AVPR1A	mRNA	-4.76764	3.54E-12	5.02E-11
SHD	mRNA	-4.76514	3.68E-12	5.21E-11
ST6GAL1	mRNA	-2.70082	3.71E-12	5.25E-11
MDFI	mRNA	4.307678	3.73E-12	5.27E-11
TESC	mRNA	5.149371	3.87E-12	5.47E-11
CHEK1	mRNA	2.347802	3.97E-12	5.60E-11
BEX2	mRNA	3.26377	4.04E-12	5.68E-11
FAM57A	mRNA	2.575955	4.10E-12	5.77E-11
CDC25C	mRNA	4.294876	4.33E-12	6.08E-11
ZWINT	mRNA	2.822778	4.40E-12	6.17E-11
MBOAT2	mRNA	2.659047	4.54E-12	6.35E-11
GPR162	mRNA	-2.15669	4.66E-12	6.50E-11
MYOF	mRNA	3.37967	4.81E-12	6.71E-11
NFE2L3	mRNA	2.823514	4.83E-12	6.72E-11
NXNL2	mRNA	4.034516	4.92E-12	6.85E-11
F10	mRNA	-2.93273	4.96E-12	6.89E-11
UNC119B	mRNA	2.15672	5.17E-12	7.18E-11
INS-IGF2	mRNA	-6.91224	5.35E-12	7.41E-11
HBB	mRNA	-3.00939	5.35E-12	7.41E-11
PDGFA	mRNA	2.857465	5.35E-12	7.41E-11
TUBB3	mRNA	5.061517	5.41E-12	7.48E-11
SFXN3	mRNA	2.452891	5.53E-12	7.64E-11
ITGA2	mRNA	4.410105	5.54E-12	7.64E-11
RNF24	mRNA	2.170945	5.55E-12	7.64E-11
SLCO1B3	mRNA	-5.68032	5.55E-12	7.64E-11

XDH	mRNA	-3.32003	5.55E-12	7.64E-11
CPNE7	mRNA	5.276206	5.76E-12	7.91E-11
RAP1GAP2	mRNA	2.909645	5.82E-12	7.99E-11
CTSV	mRNA	4.779023	5.85E-12	8.03E-11
FAM241B	mRNA	2.333349	5.96E-12	8.18E-11
TAX1BP3	mRNA	2.410066	6.11E-12	8.36E-11
KCTD17	mRNA	2.863946	6.18E-12	8.45E-11
PDE11A	mRNA	-2.97741	6.24E-12	8.53E-11
GPC5	mRNA	-3.31934	6.41E-12	8.75E-11
HPR	mRNA	-5.75345	6.45E-12	8.79E-11
C5orf30	mRNA	3.706076	6.51E-12	8.88E-11
TMSB10	mRNA	2.84751	6.59E-12	8.97E-11
OBSCN	mRNA	3.721108	6.64E-12	9.04E-11
CCDC74B	mRNA	4.743577	6.75E-12	9.18E-11
ARHGEF10	mRNA	2.157401	6.79E-12	9.23E-11
SLC22A25	mRNA	-5.54401	6.85E-12	9.30E-11
SAMD5	mRNA	-2.90105	6.91E-12	9.37E-11
TRIM55	mRNA	-4.69167	7.19E-12	9.74E-11
NUF2	mRNA	4.444412	7.24E-12	9.80E-11
FAM227A	mRNA	4.327225	7.25E-12	9.81E-11
NEK11	mRNA	2.540386	7.35E-12	9.92E-11
SRGAP1	mRNA	2.831647	7.46E-12	1.01E-10
CP	mRNA	-4.16775	7.48E-12	1.01E-10
STARD10	mRNA	-2.13845	7.58E-12	1.02E-10
SLC25A10	mRNA	-2.18997	7.62E-12	1.03E-10
CYP3A4	mRNA	-6.08362	7.71E-12	1.04E-10
APOC4-APOC2	mRNA	-4.38947	7.81E-12	1.05E-10
GPRIN2	mRNA	4.038996	7.81E-12	1.05E-10
ECHDC3	mRNA	-3.39156	7.92E-12	1.06E-10
MERTK	mRNA	-2.01827	8.15E-12	1.09E-10
PNMA1	mRNA	2.041677	8.35E-12	1.12E-10
UCK2	mRNA	2.079175	8.47E-12	1.13E-10
TMEM61	mRNA	5.261102	8.52E-12	1.14E-10
MPP3	mRNA	2.58957	8.55E-12	1.14E-10
SLC25A52	mRNA	-3.60568	9.07E-12	1.21E-10
ZDHHC1	mRNA	2.752944	9.18E-12	1.22E-10
PYGL	mRNA	-2.87539	9.46E-12	1.26E-10
SOWAHB	mRNA	-2.41437	9.98E-12	1.32E-10
C9	mRNA	-6.25118	1.05E-11	1.39E-10
CCNE1	mRNA	3.371375	1.08E-11	1.42E-10
PKD1L3	mRNA	-2.56397	1.12E-11	1.47E-10
QPRT	mRNA	-2.84365	1.14E-11	1.50E-10
UGT1A1	mRNA	-5.37032	1.18E-11	1.56E-10
C16orf96	mRNA	-3.18015	1.20E-11	1.58E-10
RORC	mRNA	-2.71878	1.22E-11	1.60E-10
TBC1D16	mRNA	2.057481	1.24E-11	1.63E-10
STEAP4	mRNA	-3.16915	1.26E-11	1.66E-10

BACE2	mRNA	3.390606	1.29E-11	1.69E-10
C3	mRNA	-2.51545	1.30E-11	1.70E-10
TRIM45	mRNA	3.515435	1.32E-11	1.73E-10
ATF5	mRNA	-3.71902	1.32E-11	1.73E-10
KNTC1	mRNA	2.034388	1.34E-11	1.76E-10
NDC80	mRNA	3.516527	1.41E-11	1.84E-10
FGGY	mRNA	-2.0099	1.41E-11	1.85E-10
CENPE	mRNA	3.379868	1.47E-11	1.91E-10
HJURP	mRNA	3.922672	1.49E-11	1.94E-10
FANCD2	mRNA	2.427291	1.50E-11	1.95E-10
GAS2	mRNA	-2.89775	1.55E-11	2.01E-10
TNNT1	mRNA	7.680755	1.59E-11	2.06E-10
TMEM132A	mRNA	5.602157	1.60E-11	2.08E-10
PRH2	mRNA	-2.97676	1.61E-11	2.08E-10
SLC6A8	mRNA	3.475357	1.64E-11	2.12E-10
PLXDC1	mRNA	2.771938	1.64E-11	2.12E-10
PEG3	mRNA	-2.81269	1.65E-11	2.13E-10
MBL2	mRNA	-5.9895	1.66E-11	2.14E-10
PLEKHG2	mRNA	2.285085	1.68E-11	2.17E-10
SH3PXD2B	mRNA	2.539054	1.69E-11	2.18E-10
TLR4	mRNA	-2.01066	1.70E-11	2.19E-10
NUSAP1	mRNA	2.435832	1.71E-11	2.20E-10
COL25A1	mRNA	-2.91052	1.75E-11	2.24E-10
PDE4A	mRNA	2.109763	1.78E-11	2.29E-10
CYP1A1	mRNA	-6.28118	1.79E-11	2.29E-10
ANXA2	mRNA	2.363849	1.82E-11	2.33E-10
OAF	mRNA	-2.60872	1.84E-11	2.35E-10
GRID2IP	mRNA	3.804232	1.84E-11	2.35E-10
SMIM1	mRNA	-2.04642	1.89E-11	2.41E-10
GBE1	mRNA	-2.19689	1.91E-11	2.44E-10
C6	mRNA	-4.28589	1.95E-11	2.49E-10
HELLS	mRNA	2.640879	1.96E-11	2.50E-10
MICU3	mRNA	-2.48887	2.01E-11	2.56E-10
NTM	mRNA	4.822371	2.08E-11	2.64E-10
GYG2	mRNA	-2.59858	2.13E-11	2.71E-10
COLEC11	mRNA	-2.18156	2.14E-11	2.71E-10
MAPK15	mRNA	4.849897	2.15E-11	2.72E-10
SLC2A2	mRNA	-5.15935	2.18E-11	2.76E-10
ALDOB	mRNA	-5.33152	2.21E-11	2.79E-10
RGS17	mRNA	4.486086	2.23E-11	2.81E-10
CFHR5	mRNA	-5.90915	2.34E-11	2.94E-10
NRSN2	mRNA	2.581494	2.40E-11	3.02E-10
CYB561D1	mRNA	2.202012	2.42E-11	3.04E-10
AMHR2	mRNA	-3.29029	2.42E-11	3.04E-10
CACNB3	mRNA	3.237666	2.47E-11	3.10E-10
LYZL1	mRNA	-4.22437	2.49E-11	3.12E-10
ATP1B2	mRNA	-2.24461	2.55E-11	3.20E-10

PLA2G12B	mRNA	-5.10007	2.58E-11	3.23E-10
PYCR1	mRNA	3.30513	2.64E-11	3.30E-10
NQO1	mRNA	5.059851	2.65E-11	3.31E-10
FHDC1	mRNA	3.856134	2.70E-11	3.37E-10
PYGB	mRNA	2.389994	2.72E-11	3.39E-10
C15orf39	mRNA	2.126723	2.83E-11	3.52E-10
SLCO1B1	mRNA	-5.3647	2.85E-11	3.55E-10
BBOX1	mRNA	-3.38608	2.90E-11	3.60E-10
TOR4A	mRNA	2.230203	2.98E-11	3.69E-10
ORC6	mRNA	3.215967	3.16E-11	3.91E-10
ZNF320	mRNA	2.520276	3.17E-11	3.92E-10
SCARB1	mRNA	-2.36564	3.18E-11	3.94E-10
CASTOR3	mRNA	2.44074	3.24E-11	4.00E-10
NYNRIN	mRNA	-2.33399	3.28E-11	4.05E-10
CCDC74A	mRNA	3.674613	3.31E-11	4.09E-10
CENPI	mRNA	3.122936	3.35E-11	4.14E-10
PWWP2B	mRNA	2.11521	3.57E-11	4.40E-10
BICD1	mRNA	2.620132	3.66E-11	4.50E-10
PPP1R14C	mRNA	7.443404	3.75E-11	4.60E-10
APCS	mRNA	-3.10413	3.75E-11	4.61E-10
CFHR1	mRNA	-5.36181	3.75E-11	4.61E-10
JRK	mRNA	2.116368	3.77E-11	4.62E-10
TICRR	mRNA	3.876315	3.84E-11	4.70E-10
FUT4	mRNA	2.301176	3.88E-11	4.76E-10
NECTIN4	mRNA	6.694469	3.99E-11	4.88E-10
RPGRIP1L	mRNA	2.120111	4.01E-11	4.90E-10
KIAA2012	mRNA	-3.3672	4.02E-11	4.90E-10
RAVER2	mRNA	2.611047	4.04E-11	4.93E-10
CENPL	mRNA	2.325023	4.07E-11	4.96E-10
GPSM2	mRNA	2.237013	4.17E-11	5.07E-10
LGALS3	mRNA	2.860841	4.35E-11	5.28E-10
SERPINA7	mRNA	-4.50784	4.36E-11	5.29E-10
APOA1	mRNA	-6.03003	4.45E-11	5.41E-10
HID1	mRNA	2.920438	4.48E-11	5.43E-10
INPP5J	mRNA	3.270672	4.58E-11	5.56E-10
KLB	mRNA	-4.19686	4.77E-11	5.78E-10
ATP1B3	mRNA	2.450873	4.81E-11	5.82E-10
SYT9	mRNA	-4.26301	4.81E-11	5.82E-10
ACOT12	mRNA	-5.7994	4.87E-11	5.89E-10
PRR18	mRNA	-3.32718	4.98E-11	6.02E-10
GLDC	mRNA	-3.1228	4.99E-11	6.02E-10
NCEH1	mRNA	2.930443	5.00E-11	6.03E-10
SOX4	mRNA	2.826203	5.04E-11	6.07E-10
TTK	mRNA	3.783863	5.07E-11	6.09E-10
PDLIM7	mRNA	2.554774	5.10E-11	6.13E-10
WDHD1	mRNA	2.383036	5.19E-11	6.23E-10
PSMC3IP	mRNA	2.398135	5.21E-11	6.25E-10

CLU	mRNA	-2.32965	5.27E-11	6.32E-10
CDKN3	mRNA	3.436768	5.35E-11	6.41E-10
CRMP1	mRNA	2.776366	5.35E-11	6.41E-10
GLUD2	mRNA	-2.20496	5.45E-11	6.53E-10
CYP1A2	mRNA	-6.91189	5.46E-11	6.53E-10
ARHGEF38	mRNA	4.285961	5.57E-11	6.65E-10
OTX1	mRNA	4.474912	5.63E-11	6.72E-10
PRKX	mRNA	2.07155	5.66E-11	6.75E-10
ELOVL7	mRNA	3.995428	5.69E-11	6.79E-10
THRSP	mRNA	-6.14683	5.72E-11	6.81E-10
SYT10	mRNA	-3.75322	5.79E-11	6.89E-10
GMNC	mRNA	-4.68019	5.82E-11	6.92E-10
COL5A3	mRNA	-2.48541	5.87E-11	6.98E-10
HAO1	mRNA	-5.78119	6.02E-11	7.14E-10
C12orf49	mRNA	2.537782	6.25E-11	7.42E-10
ARNT2	mRNA	3.815975	6.31E-11	7.48E-10
ARSF	mRNA	-4.53512	6.34E-11	7.51E-10
SPTLC3	mRNA	-2.49617	6.39E-11	7.56E-10
CCDC177	mRNA	-4.66908	6.40E-11	7.57E-10
WNT7B	mRNA	4.98947	6.48E-11	7.66E-10
HRASLS2	mRNA	-3.64774	6.66E-11	7.86E-10
MTCL1	mRNA	3.855642	6.71E-11	7.92E-10
SMIM22	mRNA	6.372438	6.78E-11	7.99E-10
RASEF	mRNA	2.820874	6.98E-11	8.22E-10
ASIP	mRNA	-2.60516	7.35E-11	8.64E-10
AL139011.2	mRNA	-2.52355	7.48E-11	8.79E-10
ACKR2	mRNA	-3.19227	7.57E-11	8.89E-10
ADGRD1	mRNA	-2.30161	7.95E-11	9.33E-10
AC011604.2	mRNA	-4.80234	8.01E-11	9.39E-10
LIPC	mRNA	-3.58218	8.16E-11	9.54E-10
HOMER3	mRNA	2.922476	8.21E-11	9.58E-10
ALDOA	mRNA	2.28921	8.29E-11	9.67E-10
TPRG1	mRNA	-2.47866	8.42E-11	9.82E-10
THPO	mRNA	-3.357	8.53E-11	9.93E-10
EPN3	mRNA	5.304609	8.75E-11	1.02E-09
ORM1	mRNA	-4.59274	9.13E-11	1.06E-09
HAGHL	mRNA	4.043867	9.45E-11	1.10E-09
DNAAF4	mRNA	3.929699	9.75E-11	1.13E-09
SLC7A1	mRNA	3.025837	9.82E-11	1.14E-09
KIAA1841	mRNA	2.291147	9.83E-11	1.14E-09
SPATA17	mRNA	4.552346	1.01E-10	1.17E-09
TEDC2	mRNA	3.021413	1.01E-10	1.17E-09
SEC14L3	mRNA	-4.43045	1.03E-10	1.19E-09
ZMYND12	mRNA	-2.38545	1.03E-10	1.19E-09
DNAH7	mRNA	3.381532	1.04E-10	1.20E-09
RIMKLA	mRNA	3.76874	1.10E-10	1.27E-09
NAGS	mRNA	-3.42731	1.13E-10	1.30E-09

GADD45A	mRNA	-2.1615	1.15E-10	1.33E-09
IGSF23	mRNA	-3.35987	1.18E-10	1.35E-09
BDKRB1	mRNA	4.146619	1.18E-10	1.35E-09
PRSS22	mRNA	4.214206	1.20E-10	1.38E-09
GMNN	mRNA	3.210746	1.21E-10	1.38E-09
CA14	mRNA	-2.17992	1.22E-10	1.39E-09
CD5L	mRNA	-5.40586	1.22E-10	1.40E-09
BCAS4	mRNA	3.261112	1.23E-10	1.40E-09
CYP4F22	mRNA	-3.91554	1.24E-10	1.42E-09
MCIDAS	mRNA	7.821824	1.25E-10	1.42E-09
TMPRSS9	mRNA	-2.41607	1.27E-10	1.45E-09
PRR11	mRNA	3.244214	1.29E-10	1.46E-09
IGF2	mRNA	-3.37124	1.30E-10	1.48E-09
ASAH2	mRNA	-2.10628	1.31E-10	1.48E-09
SLC45A4	mRNA	2.694824	1.33E-10	1.51E-09
FITM1	mRNA	-3.33904	1.35E-10	1.53E-09
TMEM125	mRNA	4.166627	1.36E-10	1.54E-09
BICDL2	mRNA	4.099885	1.39E-10	1.57E-09
KANK4	mRNA	-4.06988	1.39E-10	1.57E-09
GLIS2	mRNA	2.464819	1.41E-10	1.59E-09
TPM4	mRNA	2.185673	1.41E-10	1.59E-09
KRT19	mRNA	4.855361	1.41E-10	1.59E-09
SLC4A11	mRNA	4.739479	1.48E-10	1.67E-09
GRAMD1B	mRNA	3.496515	1.52E-10	1.71E-09
MBNL3	mRNA	-2.21936	1.54E-10	1.73E-09
ZNF532	mRNA	2.383137	1.60E-10	1.80E-09
PBK	mRNA	3.996757	1.65E-10	1.85E-09
LECT2	mRNA	-6.03393	1.65E-10	1.85E-09
AP1M2	mRNA	4.601246	1.65E-10	1.85E-09
LRFN4	mRNA	3.421498	1.67E-10	1.86E-09
HMGA1	mRNA	2.885401	1.69E-10	1.89E-09
CDCA3	mRNA	3.30769	1.70E-10	1.89E-09
SOX9	mRNA	2.778032	1.71E-10	1.90E-09
IGFBP3	mRNA	-2.47743	1.75E-10	1.95E-09
MTFR2	mRNA	3.613855	1.76E-10	1.96E-09
FLNA	mRNA	2.482191	1.77E-10	1.97E-09
ELMO3	mRNA	2.338735	1.82E-10	2.02E-09
NPTXR	mRNA	3.027866	1.85E-10	2.05E-09
UBASH3B	mRNA	3.963769	1.88E-10	2.09E-09
RUNDC3B	mRNA	-3.16301	1.92E-10	2.12E-09
NCS1	mRNA	2.555192	1.96E-10	2.17E-09
DBNDD2	mRNA	2.806207	2.01E-10	2.22E-09
RAB26	mRNA	-2.9331	2.02E-10	2.23E-09
RHCE	mRNA	-3.13906	2.05E-10	2.26E-09
DEPDC1B	mRNA	5.284988	2.05E-10	2.26E-09
TTLL2	mRNA	-3.23099	2.13E-10	2.35E-09
UGT2B4	mRNA	-4.68992	2.14E-10	2.36E-09

NLRP14	mRNA	-3.20121	2.15E-10	2.37E-09
RAD51AP1	mRNA	2.898135	2.17E-10	2.39E-09
RBMS2	mRNA	2.030782	2.18E-10	2.40E-09
C2orf15	mRNA	3.145452	2.21E-10	2.43E-09
HKDC1	mRNA	3.787613	2.21E-10	2.43E-09
NR0B2	mRNA	-3.20396	2.25E-10	2.47E-09
F9	mRNA	-6.59061	2.27E-10	2.48E-09
HORMAD2	mRNA	-4.58477	2.32E-10	2.54E-09
CRACR2B	mRNA	2.716386	2.35E-10	2.56E-09
ADH1C	mRNA	-4.49691	2.42E-10	2.64E-09
FGL1	mRNA	-3.93926	2.44E-10	2.65E-09
DPPA4	mRNA	-3.79645	2.45E-10	2.66E-09
EXPH5	mRNA	-2.02021	2.48E-10	2.69E-09
PTK7	mRNA	3.445197	2.52E-10	2.74E-09
ZNF431	mRNA	2.54501	2.57E-10	2.79E-09
E2F2	mRNA	3.135122	2.67E-10	2.88E-09
ABCC4	mRNA	2.668088	2.72E-10	2.93E-09
SPATA12	mRNA	4.462181	2.73E-10	2.95E-09
SLC25A47	mRNA	-5.68031	2.76E-10	2.97E-09
PID1	mRNA	-2.42258	2.82E-10	3.03E-09
MOGAT3	mRNA	-3.63682	2.85E-10	3.06E-09
HUNK	mRNA	3.741324	2.86E-10	3.06E-09
ASB4	mRNA	-3.40954	2.86E-10	3.06E-09
VNN1	mRNA	-2.81113	2.97E-10	3.18E-09
CHRM2	mRNA	-4.74941	2.98E-10	3.18E-09
LYPD6B	mRNA	5.814275	2.98E-10	3.18E-09
SULT1A2	mRNA	-2.25812	2.98E-10	3.19E-09
IQCD	mRNA	3.378061	3.04E-10	3.24E-09
KRT80	mRNA	4.352188	3.07E-10	3.28E-09
NOL4	mRNA	-4.21628	3.10E-10	3.30E-09
SLC43A1	mRNA	-2.2307	3.13E-10	3.33E-09
FRMD7	mRNA	-3.82704	3.15E-10	3.35E-09
PSORS1C2	mRNA	6.114046	3.15E-10	3.35E-09
ARRDC4	mRNA	-2.19123	3.20E-10	3.40E-09
SPP2	mRNA	-5.81801	3.24E-10	3.43E-09
ADCY10	mRNA	-2.65158	3.28E-10	3.48E-09
SKIDA1	mRNA	-3.00296	3.30E-10	3.49E-09
PCLO	mRNA	3.003305	3.34E-10	3.54E-09
APOA2	mRNA	-5.31154	3.39E-10	3.58E-09
PTHLH	mRNA	5.411067	3.68E-10	3.88E-09
F13B	mRNA	-5.42023	3.82E-10	4.03E-09
BICC1	mRNA	3.817552	3.89E-10	4.09E-09
C1orf116	mRNA	4.344407	3.93E-10	4.13E-09
CDS1	mRNA	2.839667	3.98E-10	4.18E-09
PPP1R13L	mRNA	2.777428	4.00E-10	4.20E-09
KRBA1	mRNA	2.072332	4.02E-10	4.22E-09
ADCY1	mRNA	-2.67166	4.06E-10	4.26E-09

GALP	mRNA	-4.4191	4.13E-10	4.32E-09
WDR54	mRNA	2.363873	4.18E-10	4.38E-09
IL11	mRNA	5.043879	4.21E-10	4.40E-09
NLRP6	mRNA	-2.89883	4.40E-10	4.60E-09
AC135586.2	mRNA	-3.1819	4.50E-10	4.69E-09
SLC35F3	mRNA	5.45191	4.56E-10	4.75E-09
ADRA2C	mRNA	4.074197	4.57E-10	4.76E-09
FAM20A	mRNA	-2.50935	4.69E-10	4.88E-09
AGXT2	mRNA	-4.78125	4.71E-10	4.90E-09
MACC1	mRNA	3.615969	4.72E-10	4.90E-09
SPC24	mRNA	3.177574	4.79E-10	4.97E-09
ITIH2	mRNA	-3.45578	4.80E-10	4.98E-09
CLDN7	mRNA	2.868441	4.84E-10	5.01E-09
CDCA7	mRNA	5.649788	4.87E-10	5.05E-09
CCDC78	mRNA	3.701247	4.89E-10	5.06E-09
HIST3H2BB	mRNA	5.989398	4.93E-10	5.10E-09
NT5DC2	mRNA	2.190918	4.99E-10	5.15E-09
CEBPA	mRNA	-2.85576	5.06E-10	5.22E-09
E2F7	mRNA	4.624285	5.19E-10	5.35E-09
CENPO	mRNA	2.006163	5.21E-10	5.36E-09
CLDN4	mRNA	4.255173	5.47E-10	5.63E-09
CAPG	mRNA	2.70487	5.47E-10	5.63E-09
TNFSF15	mRNA	4.161376	5.48E-10	5.63E-09
GRIP1	mRNA	3.402039	5.56E-10	5.71E-09
ABCG8	mRNA	-4.69205	5.62E-10	5.77E-09
SPDYC	mRNA	-3.89	5.63E-10	5.78E-09
BSPRY	mRNA	3.011857	5.73E-10	5.87E-09
EME1	mRNA	2.998092	5.89E-10	6.03E-09
COL22A1	mRNA	5.550504	5.98E-10	6.12E-09
TACC3	mRNA	2.202956	6.00E-10	6.13E-09
DUSP1	mRNA	-2.1488	6.08E-10	6.21E-09
MAFB	mRNA	-2.02828	6.13E-10	6.26E-09
ATP1A1	mRNA	2.721941	6.14E-10	6.27E-09
LIN7A	mRNA	-2.2693	6.34E-10	6.46E-09
GNA14	mRNA	-2.26201	6.38E-10	6.50E-09
FKBP10	mRNA	2.741947	6.40E-10	6.52E-09
ZNF185	mRNA	2.987705	6.41E-10	6.52E-09
SPP1	mRNA	6.197316	6.48E-10	6.59E-09
LPIN2	mRNA	-2.16878	6.51E-10	6.61E-09
ALDH1B1	mRNA	-2.45369	6.53E-10	6.63E-09
SYT13	mRNA	5.710688	6.55E-10	6.64E-09
3-Sep	mRNA	5.367179	6.63E-10	6.72E-09
HP	mRNA	-5.57381	6.68E-10	6.76E-09
SH3YL1	mRNA	2.330979	6.74E-10	6.81E-09
MTHFD1L	mRNA	2.078399	6.77E-10	6.85E-09
FAXC	mRNA	5.16734	6.89E-10	6.96E-09
NR1H4	mRNA	-2.39232	6.97E-10	7.03E-09

PAQR5	mRNA	3.634924	7.09E-10	7.15E-09
FAM171A2	mRNA	3.416676	7.11E-10	7.16E-09
GPM6A	mRNA	-3.3602	7.27E-10	7.32E-09
STC2	mRNA	3.424596	7.31E-10	7.35E-09
CCBE1	mRNA	-3.15662	7.38E-10	7.42E-09
DAND5	mRNA	5.55923	7.52E-10	7.55E-09
CCL14	mRNA	-3.01336	7.57E-10	7.59E-09
BARD1	mRNA	2.020756	7.61E-10	7.63E-09
HBD	mRNA	-4.38322	7.73E-10	7.74E-09
CCDC191	mRNA	2.013363	7.80E-10	7.80E-09
SCML1	mRNA	-2.00593	7.80E-10	7.80E-09
CD58	mRNA	2.288965	7.82E-10	7.81E-09
GPSM1	mRNA	2.190675	7.82E-10	7.81E-09
ENPP3	mRNA	-3.80513	7.86E-10	7.85E-09
KIF14	mRNA	3.207261	7.94E-10	7.92E-09
CTXN1	mRNA	5.239037	7.96E-10	7.93E-09
FRAS1	mRNA	4.316032	8.03E-10	7.99E-09
DUSP4	mRNA	3.208949	8.11E-10	8.07E-09
AFAP1	mRNA	2.323908	8.38E-10	8.31E-09
JAG2	mRNA	2.047648	8.44E-10	8.38E-09
OLFML2A	mRNA	2.350296	8.52E-10	8.45E-09
TMEM51	mRNA	2.724079	8.58E-10	8.50E-09
KCNF1	mRNA	4.73336	8.66E-10	8.58E-09
ACSL5	mRNA	-2.78067	8.83E-10	8.74E-09
KLF9	mRNA	-2.15158	8.99E-10	8.88E-09
SLC25A36	mRNA	2.014441	8.99E-10	8.89E-09
ZNF711	mRNA	2.383299	9.03E-10	8.91E-09
FBXO40	mRNA	-3.88577	9.05E-10	8.92E-09
WNK2	mRNA	3.982393	9.29E-10	9.15E-09
GNAZ	mRNA	3.261753	9.91E-10	9.74E-09
MTMR11	mRNA	3.364048	9.94E-10	9.77E-09
AHSP	mRNA	-3.98945	1.01E-09	9.93E-09
TRIP13	mRNA	4.46972	1.02E-09	9.99E-09
ANKRD13B	mRNA	2.866337	1.02E-09	1.00E-08
NUDT10	mRNA	-2.96207	1.03E-09	1.01E-08
HGD	mRNA	-2.53619	1.03E-09	1.01E-08
FXYD3	mRNA	6.237677	1.05E-09	1.03E-08
GOT1	mRNA	-2.09418	1.06E-09	1.04E-08
RTP3	mRNA	-5.79487	1.09E-09	1.06E-08
TPBG	mRNA	2.888411	1.10E-09	1.07E-08
SLC34A2	mRNA	4.837645	1.12E-09	1.09E-08
RXRG	mRNA	-3.88611	1.12E-09	1.09E-08
CSGALNACT1	mRNA	3.39532	1.13E-09	1.10E-08
HES4	mRNA	2.446176	1.17E-09	1.13E-08
SMKR1	mRNA	4.285173	1.19E-09	1.16E-08
CCDC9B	mRNA	2.734519	1.20E-09	1.17E-08
CLSPN	mRNA	3.603725	1.22E-09	1.18E-08

WDR62	mRNA	2.668438	1.23E-09	1.19E-08
ITGA3	mRNA	3.987844	1.26E-09	1.22E-08
CERCAM	mRNA	2.452398	1.28E-09	1.24E-08
DNAH6	mRNA	-2.25535	1.33E-09	1.28E-08
CD36	mRNA	-2.93853	1.34E-09	1.29E-08
NEU4	mRNA	-4.31698	1.35E-09	1.29E-08
SRRM3	mRNA	5.15865	1.37E-09	1.31E-08
DNAJC12	mRNA	-2.79883	1.37E-09	1.32E-08
CRYBG2	mRNA	4.524965	1.43E-09	1.37E-08
PRELID3A	mRNA	3.359709	1.51E-09	1.45E-08
ETV4	mRNA	4.025033	1.51E-09	1.45E-08
TK1	mRNA	2.514482	1.52E-09	1.46E-08
ZNF296	mRNA	3.313871	1.56E-09	1.49E-08
FAXDC2	mRNA	-2.21776	1.56E-09	1.49E-08
SULT1B1	mRNA	-3.3266	1.61E-09	1.54E-08
ADH7	mRNA	-4.32239	1.62E-09	1.55E-08
KIF25	mRNA	-2.75273	1.66E-09	1.58E-08
FANCB	mRNA	2.836587	1.66E-09	1.58E-08
WDR76	mRNA	2.38551	1.67E-09	1.58E-08
FANK1	mRNA	3.537139	1.67E-09	1.59E-08
PLEKHG4	mRNA	3.173175	1.68E-09	1.60E-08
WNT9A	mRNA	3.478119	1.71E-09	1.62E-08
ACSM2A	mRNA	-4.74544	1.73E-09	1.64E-08
COLCA2	mRNA	3.333558	1.74E-09	1.65E-08
GLRX	mRNA	-2.04148	1.76E-09	1.66E-08
B3GALT4	mRNA	2.178119	1.78E-09	1.68E-08
POLQ	mRNA	3.694467	1.78E-09	1.68E-08
PRAP1	mRNA	-3.97115	1.79E-09	1.69E-08
KNG1	mRNA	-4.91779	1.81E-09	1.71E-08
COL4A2	mRNA	2.346944	1.81E-09	1.71E-08
CTNND2	mRNA	4.047752	2.00E-09	1.88E-08
CES5A	mRNA	-4.46842	2.01E-09	1.89E-08
BCO2	mRNA	-2.61362	2.09E-09	1.96E-08
CAMK2B	mRNA	-3.0411	2.10E-09	1.97E-08
CENPK	mRNA	2.92254	2.14E-09	2.00E-08
SLC22A24	mRNA	-3.85036	2.18E-09	2.04E-08
ABCC2	mRNA	-3.3667	2.20E-09	2.06E-08
CERS4	mRNA	-2.08163	2.22E-09	2.07E-08
CENPU	mRNA	2.228142	2.28E-09	2.12E-08
GRB7	mRNA	2.527552	2.28E-09	2.13E-08
ZSWIM4	mRNA	2.173647	2.32E-09	2.17E-08
PLEKHG4B	mRNA	6.13923	2.34E-09	2.18E-08
BMP8B	mRNA	3.096552	2.35E-09	2.19E-08
DCDC2	mRNA	4.31048	2.36E-09	2.19E-08
PIWIL4	mRNA	3.187941	2.36E-09	2.20E-08
CYP3A7	mRNA	-3.47256	2.38E-09	2.21E-08
CMTM1	mRNA	2.945027	2.40E-09	2.23E-08

CFHR4	mRNA	-5.65862	2.50E-09	2.32E-08
SAA2-SAA4	mRNA	-4.91624	2.56E-09	2.38E-08
F7	mRNA	-3.79658	2.64E-09	2.45E-08
SLC16A3	mRNA	3.165442	2.66E-09	2.46E-08
OLFML2B	mRNA	2.522219	2.67E-09	2.46E-08
THY1	mRNA	2.199856	2.74E-09	2.54E-08
ERCC6L	mRNA	3.028897	2.84E-09	2.62E-08
TIGD1	mRNA	2.249466	2.84E-09	2.62E-08
LMTK3	mRNA	3.277981	2.84E-09	2.62E-08
PKP3	mRNA	5.227998	2.86E-09	2.63E-08
HES2	mRNA	4.195324	2.87E-09	2.64E-08
SAA1	mRNA	-4.57509	2.99E-09	2.75E-08
FCHSD1	mRNA	2.045632	3.09E-09	2.83E-08
SOCS7	mRNA	2.044921	3.09E-09	2.83E-08
TMEM159	mRNA	2.865217	3.15E-09	2.89E-08
ULBP1	mRNA	4.42662	3.17E-09	2.90E-08
TYRO3	mRNA	3.238531	3.27E-09	2.99E-08
ZC3HAV1L	mRNA	2.923132	3.28E-09	2.99E-08
KIF15	mRNA	2.968003	3.38E-09	3.08E-08
KCNQ1	mRNA	3.261933	3.39E-09	3.09E-08
KRTCAP3	mRNA	2.484237	3.40E-09	3.10E-08
SLC7A11	mRNA	5.615114	3.43E-09	3.12E-08
ZNF83	mRNA	2.114944	3.51E-09	3.19E-08
DMBX1	mRNA	7.86708	3.59E-09	3.26E-08
SLC39A6	mRNA	2.171088	3.76E-09	3.41E-08
C4BPB	mRNA	-3.35218	3.80E-09	3.44E-08
DSTYK	mRNA	2.098664	4.08E-09	3.68E-08
LEF1	mRNA	3.439752	4.11E-09	3.70E-08
MOB3B	mRNA	2.316296	4.13E-09	3.72E-08
PLK4	mRNA	2.472781	4.21E-09	3.79E-08
RARG	mRNA	2.115182	4.34E-09	3.90E-08
FAM83B	mRNA	4.973695	4.35E-09	3.91E-08
MYRF	mRNA	2.802564	4.36E-09	3.92E-08
PDZK1IP1	mRNA	5.58905	4.37E-09	3.92E-08
SLC35F2	mRNA	3.544125	4.38E-09	3.93E-08
CDH24	mRNA	2.450455	4.38E-09	3.93E-08
LRRC56	mRNA	2.275286	4.49E-09	4.02E-08
NRM	mRNA	2.127491	4.55E-09	4.06E-08
JAG1	mRNA	2.347479	4.61E-09	4.12E-08
FBXO41	mRNA	3.077154	4.62E-09	4.12E-08
BICDL1	mRNA	2.453821	4.64E-09	4.14E-08
CALML3	mRNA	-5.25265	4.67E-09	4.17E-08
LYPD1	mRNA	5.1974	4.68E-09	4.17E-08
ABHD17C	mRNA	2.206864	4.71E-09	4.19E-08
PLLP	mRNA	2.412693	4.76E-09	4.23E-08
CMBL	mRNA	-3.07005	4.87E-09	4.32E-08
GPR88	mRNA	-4.435	4.91E-09	4.36E-08

HPGD	mRNA	-4.01762	4.93E-09	4.37E-08
IL18R1	mRNA	-2.09038	4.94E-09	4.38E-08
XKR6	mRNA	3.140546	4.98E-09	4.41E-08
MAD2L1	mRNA	2.418088	5.02E-09	4.44E-08
DPCD	mRNA	2.222502	5.18E-09	4.58E-08
TMEM97	mRNA	-2.10695	5.20E-09	4.59E-08
ADAP1	mRNA	2.907151	5.30E-09	4.67E-08
KIF3C	mRNA	3.530683	5.51E-09	4.84E-08
SUGCT	mRNA	-2.21452	5.60E-09	4.92E-08
MLLT3	mRNA	2.840197	5.61E-09	4.93E-08
RASAL2	mRNA	2.389794	5.62E-09	4.93E-08
KLC3	mRNA	4.438663	5.82E-09	5.10E-08
HFE2	mRNA	-5.21978	5.85E-09	5.12E-08
CREB3L3	mRNA	-3.91149	5.87E-09	5.14E-08
PRR15L	mRNA	3.763805	5.88E-09	5.14E-08
SLC17A1	mRNA	-2.73455	6.00E-09	5.24E-08
TMEM136	mRNA	2.360779	6.06E-09	5.29E-08
TRNP1	mRNA	2.758472	6.12E-09	5.34E-08
NMB	mRNA	2.840098	6.20E-09	5.40E-08
SCTR	mRNA	5.505758	6.21E-09	5.41E-08
ADORA2A	mRNA	-2.61003	6.33E-09	5.50E-08
LOXL4	mRNA	3.555865	6.34E-09	5.50E-08
TNFRSF11A	mRNA	3.234233	6.41E-09	5.56E-08
GPR27	mRNA	4.15393	6.41E-09	5.56E-08
HASPIN	mRNA	2.876136	6.53E-09	5.65E-08
TRPM8	mRNA	-3.26487	6.54E-09	5.66E-08
KIF24	mRNA	2.419588	6.57E-09	5.69E-08
SUMO4	mRNA	-2.47683	6.63E-09	5.73E-08
TRIM17	mRNA	4.86152	6.68E-09	5.77E-08
PNPLA3	mRNA	-2.80063	6.72E-09	5.80E-08
UGT2B15	mRNA	-3.22278	6.82E-09	5.88E-08
DRP2	mRNA	5.203315	6.83E-09	5.89E-08
ZG16B	mRNA	4.68728	6.85E-09	5.90E-08
AHNAK2	mRNA	5.318146	6.88E-09	5.92E-08
AP3B2	mRNA	5.758259	6.92E-09	5.95E-08
PLPPR1	mRNA	-2.92165	7.23E-09	6.20E-08
KIF26B	mRNA	4.193004	7.24E-09	6.20E-08
DENND2C	mRNA	-2.16811	7.26E-09	6.22E-08
DCDC1	mRNA	-2.61769	7.30E-09	6.25E-08
SLC17A3	mRNA	-3.06571	7.43E-09	6.35E-08
SLC6A6	mRNA	2.590892	7.43E-09	6.35E-08
CHI3L1	mRNA	-3.21499	7.74E-09	6.61E-08
AS3MT	mRNA	-2.45055	7.81E-09	6.66E-08
LIPH	mRNA	4.5034	8.19E-09	6.97E-08
STRA6	mRNA	8.268378	8.25E-09	7.02E-08
GOLM1	mRNA	2.184492	8.37E-09	7.12E-08
PDE5A	mRNA	2.667324	8.39E-09	7.13E-08

KRTAP5-1	mRNA	3.955042	8.44E-09	7.17E-08
C4orf48	mRNA	3.398417	8.46E-09	7.18E-08
DAB1	mRNA	-3.00289	8.53E-09	7.23E-08
PQLC2L	mRNA	5.673299	8.60E-09	7.29E-08
GALNT10	mRNA	2.295307	8.78E-09	7.43E-08
ASPM	mRNA	2.937032	8.84E-09	7.48E-08
CFAP57	mRNA	-2.1312	8.87E-09	7.50E-08
OGDHL	mRNA	-2.88215	8.91E-09	7.53E-08
TAT	mRNA	-5.00995	9.38E-09	7.93E-08
SLC2A4	mRNA	-2.82664	9.60E-09	8.11E-08
GJC1	mRNA	2.487943	9.74E-09	8.21E-08
G6PD	mRNA	2.961117	9.82E-09	8.27E-08
GPX3	mRNA	-2.33733	9.82E-09	8.27E-08
UNC13D	mRNA	3.448816	1.01E-08	8.46E-08
GALNT12	mRNA	3.293907	1.01E-08	8.49E-08
FLVCR1	mRNA	2.180867	1.03E-08	8.62E-08
LAMC1	mRNA	2.228044	1.05E-08	8.82E-08
ARHGEF16	mRNA	2.526012	1.09E-08	9.09E-08
KIAA1549L	mRNA	4.26622	1.09E-08	9.12E-08
C11orf97	mRNA	-3.01239	1.10E-08	9.22E-08
ADGRB2	mRNA	3.581409	1.10E-08	9.24E-08
COL15A1	mRNA	3.171568	1.11E-08	9.25E-08
MYBPH	mRNA	-3.371	1.12E-08	9.36E-08
KCNJ11	mRNA	2.977319	1.12E-08	9.36E-08
B4GALNT3	mRNA	4.184383	1.16E-08	9.64E-08
PDGFD	mRNA	3.31952	1.17E-08	9.72E-08
DMRTA1	mRNA	-2.40638	1.17E-08	9.76E-08
KRT23	mRNA	6.093897	1.18E-08	9.80E-08
LYPD6	mRNA	4.815539	1.18E-08	9.83E-08
FOXJ1	mRNA	5.694061	1.21E-08	1.00E-07
FGD6	mRNA	2.341614	1.21E-08	1.00E-07
RTKN2	mRNA	4.189593	1.22E-08	1.01E-07
ZNF469	mRNA	3.227133	1.25E-08	1.03E-07
HBA2	mRNA	-2.84468	1.28E-08	1.06E-07
AADAC	mRNA	-3.46485	1.30E-08	1.07E-07
SBK1	mRNA	3.194167	1.32E-08	1.09E-07
ITGB8	mRNA	3.220165	1.36E-08	1.12E-07
SLC39A5	mRNA	-3.28384	1.36E-08	1.12E-07
RETREG1	mRNA	-2.27379	1.37E-08	1.13E-07
AC025594.2	mRNA	3.237843	1.40E-08	1.15E-07
S100A2	mRNA	5.081237	1.40E-08	1.15E-07
RAB6B	mRNA	3.613586	1.40E-08	1.15E-07
NEURL3	mRNA	4.587479	1.44E-08	1.19E-07
RS1	mRNA	-3.9343	1.45E-08	1.19E-07
NOTCH3	mRNA	2.486408	1.47E-08	1.20E-07
BLM	mRNA	2.679024	1.57E-08	1.29E-07
HOXB9	mRNA	8.676266	1.60E-08	1.31E-07

AC093155.3	mRNA	-3.10259	1.60E-08	1.31E-07
ADAM28	mRNA	3.592997	1.62E-08	1.32E-07
IKBKE	mRNA	2.200438	1.64E-08	1.34E-07
TMC5	mRNA	5.368202	1.67E-08	1.36E-07
FAM46A	mRNA	-2.14424	1.67E-08	1.36E-07
AGRN	mRNA	2.141544	1.67E-08	1.37E-07
ACR	mRNA	-2.2403	1.68E-08	1.37E-07
NR3C2	mRNA	-2.0945	1.69E-08	1.38E-07
APOA4	mRNA	-4.69612	1.73E-08	1.41E-07
PPP2R2C	mRNA	6.607457	1.74E-08	1.42E-07
SULT2A1	mRNA	-5.35747	1.77E-08	1.44E-07
IBSP	mRNA	8.034139	1.78E-08	1.44E-07
GSTA1	mRNA	-4.9765	1.80E-08	1.46E-07
ANO1	mRNA	-2.23115	1.84E-08	1.49E-07
SNTG1	mRNA	-3.43717	1.85E-08	1.50E-07
5-Sep	mRNA	2.423333	1.93E-08	1.56E-07
ZYG11A	mRNA	-3.40632	1.94E-08	1.57E-07
TMEM100	mRNA	-2.32536	1.95E-08	1.58E-07
CXCL12	mRNA	-2.10526	1.97E-08	1.59E-07
PARPBP	mRNA	2.485361	1.98E-08	1.60E-07
RECQL4	mRNA	2.135189	2.00E-08	1.61E-07
PIFO	mRNA	4.876162	2.00E-08	1.61E-07
CPN2	mRNA	-5.33823	2.04E-08	1.64E-07
TFCP2L1	mRNA	4.505864	2.07E-08	1.67E-07
C5orf34	mRNA	2.417561	2.12E-08	1.70E-07
DIO1	mRNA	-4.05284	2.15E-08	1.73E-07
MATN3	mRNA	5.554697	2.15E-08	1.73E-07
ST8SIA6	mRNA	-2.4934	2.23E-08	1.79E-07
HIST1H4H	mRNA	3.996443	2.25E-08	1.80E-07
MROH2A	mRNA	-4.1966	2.25E-08	1.81E-07
RGSL1	mRNA	-3.21238	2.27E-08	1.82E-07
TRAF5	mRNA	3.029948	2.27E-08	1.82E-07
UBD	mRNA	4.00514	2.31E-08	1.85E-07
S100A12	mRNA	-2.8843	2.33E-08	1.86E-07
HCN4	mRNA	4.904628	2.35E-08	1.88E-07
SPA17	mRNA	2.277551	2.37E-08	1.89E-07
SAA2	mRNA	-4.46147	2.45E-08	1.95E-07
VEPH1	mRNA	3.797774	2.46E-08	1.96E-07
NXPH4	mRNA	5.690457	2.56E-08	2.04E-07
ACTL10	mRNA	2.559317	2.63E-08	2.09E-07
GALNT3	mRNA	3.311486	2.66E-08	2.12E-07
GFRA1	mRNA	-3.65302	2.68E-08	2.13E-07
ZBTB12	mRNA	2.257531	2.70E-08	2.14E-07
RASGEF1A	mRNA	4.307384	2.73E-08	2.16E-07
CIP2A	mRNA	2.640354	2.76E-08	2.19E-07
ZNF239	mRNA	2.643377	2.80E-08	2.22E-07
TAP1	mRNA	2.069656	2.81E-08	2.23E-07

STMN1	mRNA	2.140575	2.85E-08	2.25E-07
ARHGAP20	mRNA	-2.64187	2.86E-08	2.26E-07
FAM163B	mRNA	-2.75421	2.86E-08	2.26E-07
GRAMD1A	mRNA	2.033289	2.90E-08	2.29E-07
IP6K3	mRNA	-3.3832	2.97E-08	2.34E-07
HIST2H2BF	mRNA	4.460585	3.03E-08	2.38E-07
IL6R	mRNA	-2.07454	3.03E-08	2.38E-07
ARMC3	mRNA	8.060683	3.05E-08	2.40E-07
GAL3ST1	mRNA	3.559294	3.07E-08	2.42E-07
HK1	mRNA	2.136639	3.10E-08	2.44E-07
DAGLA	mRNA	2.537282	3.12E-08	2.45E-07
DUOX1	mRNA	4.25122	3.15E-08	2.47E-07
HMMR	mRNA	3.063783	3.18E-08	2.49E-07
PLXNB3	mRNA	4.117424	3.19E-08	2.50E-07
C19orf33	mRNA	6.085168	3.29E-08	2.57E-07
MDK	mRNA	3.633974	3.31E-08	2.58E-07
SGO2	mRNA	2.359048	3.33E-08	2.59E-07
NRSN1	mRNA	5.434358	3.33E-08	2.60E-07
IGF2BP2	mRNA	2.603093	3.39E-08	2.64E-07
SULF1	mRNA	3.060435	3.46E-08	2.69E-07
TRIM47	mRNA	2.002881	3.48E-08	2.70E-07
SMOX	mRNA	2.175426	3.50E-08	2.72E-07
GCNT3	mRNA	5.171967	3.51E-08	2.72E-07
HS3ST1	mRNA	3.66272	3.52E-08	2.73E-07
AK8	mRNA	3.043265	3.57E-08	2.76E-07
SYNGR3	mRNA	4.437428	3.61E-08	2.79E-07
SLC44A3	mRNA	2.742611	3.64E-08	2.81E-07
ADGRF4	mRNA	7.444379	3.71E-08	2.86E-07
AC005041.1	mRNA	2.614157	3.71E-08	2.87E-07
ZNF714	mRNA	2.640528	3.73E-08	2.88E-07
TEX9	mRNA	2.419982	3.78E-08	2.92E-07
S100A3	mRNA	3.936571	3.87E-08	2.98E-07
SULT1E1	mRNA	-4.07667	3.89E-08	3.00E-07
FZD6	mRNA	2.07548	3.94E-08	3.03E-07
FGFR1	mRNA	2.471757	3.95E-08	3.03E-07
TFPI	mRNA	-2.50349	3.95E-08	3.03E-07
LINC00672	mRNA	3.046615	3.96E-08	3.05E-07
C1QL4	mRNA	7.217209	3.97E-08	3.05E-07
DSG2	mRNA	2.2263	3.99E-08	3.06E-07
GDF2	mRNA	-5.43363	4.02E-08	3.08E-07
SLC35E4	mRNA	2.248613	4.06E-08	3.11E-07
DTX3	mRNA	2.131424	4.15E-08	3.18E-07
CFAP45	mRNA	3.721078	4.16E-08	3.18E-07
APOC3	mRNA	-5.29896	4.19E-08	3.21E-07
CIB2	mRNA	3.132704	4.26E-08	3.25E-07
CALB2	mRNA	6.74429	4.32E-08	3.29E-07
CNTFR	mRNA	-3.72511	4.47E-08	3.41E-07

CFAP221	mRNA	3.167474	4.59E-08	3.50E-07
VANGL2	mRNA	4.226671	4.73E-08	3.60E-07
IGF1	mRNA	-3.1337	4.74E-08	3.61E-07
GAD1	mRNA	6.230968	4.79E-08	3.64E-07
TMEM45A	mRNA	-2.42552	4.84E-08	3.68E-07
ZNF738	mRNA	2.778063	4.99E-08	3.78E-07
ADAMTS9	mRNA	2.272172	5.09E-08	3.85E-07
LAMA3	mRNA	2.988393	5.10E-08	3.86E-07
LGALS9	mRNA	2.315702	5.20E-08	3.93E-07
SPATC1L	mRNA	2.60243	5.23E-08	3.95E-07
KAAG1	mRNA	4.282903	5.24E-08	3.95E-07
CCNF	mRNA	2.137696	5.35E-08	4.03E-07
GRIN1	mRNA	4.206053	5.39E-08	4.06E-07
CCDC30	mRNA	2.288849	5.41E-08	4.07E-07
SLC26A2	mRNA	2.110147	5.49E-08	4.12E-07
ZNF695	mRNA	5.908294	5.50E-08	4.13E-07
TUBA1A	mRNA	2.321299	5.56E-08	4.17E-07
EDIL3	mRNA	3.152344	5.58E-08	4.18E-07
PHGDH	mRNA	-2.00894	5.65E-08	4.23E-07
CACNB1	mRNA	2.63858	5.67E-08	4.24E-07
SLC1A5	mRNA	2.48142	5.67E-08	4.24E-07
MND1	mRNA	2.838975	5.72E-08	4.28E-07
LRRC2	mRNA	-3.2044	5.75E-08	4.30E-07
HMGCS2	mRNA	-4.58796	5.80E-08	4.33E-07
NPNT	mRNA	3.390916	5.85E-08	4.36E-07
C17orf53	mRNA	2.179881	5.90E-08	4.40E-07
C5	mRNA	-2.65055	5.90E-08	4.40E-07
BDKRB2	mRNA	2.287471	6.05E-08	4.50E-07
ZNF860	mRNA	3.760617	6.18E-08	4.59E-07
MMP24	mRNA	3.813147	6.20E-08	4.60E-07
DEGS2	mRNA	3.530748	6.22E-08	4.61E-07
DSG1	mRNA	-3.74902	6.26E-08	4.64E-07
PLA2G4F	mRNA	6.182154	6.27E-08	4.65E-07
PDZD7	mRNA	3.350992	6.29E-08	4.66E-07
INMT	mRNA	-2.12474	6.30E-08	4.66E-07
COL4A1	mRNA	2.204303	6.36E-08	4.71E-07
LHPP	mRNA	-2.10652	6.40E-08	4.74E-07
RNF227	mRNA	2.1087	6.42E-08	4.75E-07
KREMEN2	mRNA	4.318439	6.57E-08	4.85E-07
NPTX1	mRNA	5.535799	6.60E-08	4.87E-07
LRRC49	mRNA	2.431775	6.63E-08	4.88E-07
AC002094.3	mRNA	-3.19775	6.74E-08	4.96E-07
TMEM178B	mRNA	4.031841	6.80E-08	5.00E-07
STEAP3	mRNA	-2.38316	6.82E-08	5.01E-07
FAP	mRNA	3.437862	6.96E-08	5.11E-07
OTOG	mRNA	6.604347	6.98E-08	5.12E-07
ESCO2	mRNA	2.621712	7.01E-08	5.15E-07

APBA1	mRNA	-2.02679	7.10E-08	5.21E-07
MUC13	mRNA	6.752477	7.11E-08	5.22E-07
NLGN4X	mRNA	-2.10707	7.14E-08	5.23E-07
NOX4	mRNA	3.230107	7.15E-08	5.23E-07
CARD11	mRNA	3.055281	7.23E-08	5.29E-07
COL10A1	mRNA	5.000818	7.46E-08	5.45E-07
XG	mRNA	-3.12918	7.51E-08	5.48E-07
PRTFDC1	mRNA	2.519541	7.51E-08	5.48E-07
LEMD1	mRNA	10.21707	7.56E-08	5.51E-07
SPRED3	mRNA	3.185212	7.81E-08	5.68E-07
PPP4R4	mRNA	-2.98699	7.97E-08	5.79E-07
TGFB2	mRNA	3.563654	8.10E-08	5.88E-07
SULT2B1	mRNA	4.902608	8.44E-08	6.12E-07
PADI2	mRNA	2.524785	8.63E-08	6.25E-07
DPF1	mRNA	3.487727	8.65E-08	6.26E-07
EPO	mRNA	-3.06917	8.70E-08	6.29E-07
GRIN2A	mRNA	4.962468	8.78E-08	6.34E-07
ASCL5	mRNA	4.095255	8.87E-08	6.40E-07
PPEF1	mRNA	3.763863	8.92E-08	6.43E-07
ARHGAP39	mRNA	2.195703	9.05E-08	6.52E-07
CENPH	mRNA	2.007868	9.33E-08	6.70E-07
DNM1	mRNA	3.002831	9.38E-08	6.73E-07
MAP9	mRNA	2.649561	9.40E-08	6.74E-07
ASPHD2	mRNA	2.708056	9.43E-08	6.76E-07
RAB19	mRNA	2.881339	9.48E-08	6.79E-07
CLDN10	mRNA	3.841089	9.56E-08	6.84E-07
GSTP1	mRNA	3.410985	9.58E-08	6.85E-07
EEF1A2	mRNA	5.109204	9.63E-08	6.88E-07
KRT86	mRNA	3.743546	9.69E-08	6.92E-07
PLIN5	mRNA	-2.44465	9.71E-08	6.93E-07
OR2I1P	mRNA	4.328969	9.74E-08	6.95E-07
KIAA1549	mRNA	2.327376	9.92E-08	7.07E-07
TMC6	mRNA	2.125146	9.96E-08	7.10E-07
TNFRSF19	mRNA	3.547975	1.00E-07	7.14E-07
GABRD	mRNA	3.63189	1.02E-07	7.26E-07
TPX2	mRNA	2.265485	1.02E-07	7.29E-07
ENTPD2	mRNA	2.829358	1.03E-07	7.32E-07
CD1D	mRNA	-2.39704	1.03E-07	7.34E-07
NRARP	mRNA	2.609992	1.04E-07	7.36E-07
ARHGEF39	mRNA	2.698287	1.05E-07	7.46E-07
CDC7	mRNA	2.181028	1.05E-07	7.46E-07
PYCARD	mRNA	2.219871	1.06E-07	7.51E-07
COL7A1	mRNA	3.484337	1.07E-07	7.60E-07
MC1R	mRNA	2.170423	1.08E-07	7.63E-07
SEMA6A	mRNA	2.615	1.11E-07	7.83E-07
TRIM46	mRNA	4.782236	1.12E-07	7.91E-07
CFHR2	mRNA	-5.10182	1.13E-07	7.94E-07

CREB5	mRNA	2.612737	1.13E-07	7.95E-07
RPL3L	mRNA	-2.06593	1.18E-07	8.27E-07
RNFT2	mRNA	3.939459	1.18E-07	8.27E-07
FMN1	mRNA	2.627317	1.20E-07	8.40E-07
BRSK2	mRNA	3.935228	1.21E-07	8.50E-07
EVX1	mRNA	7.820088	1.21E-07	8.50E-07
SLC6A14	mRNA	9.358574	1.22E-07	8.52E-07
FRMD5	mRNA	5.015065	1.27E-07	8.87E-07
GLOD5	mRNA	-2.39615	1.28E-07	8.92E-07
SNAP25	mRNA	3.485954	1.28E-07	8.92E-07
NPAS2	mRNA	2.665474	1.29E-07	9.00E-07
GAS2L3	mRNA	2.543652	1.31E-07	9.13E-07
FOXF2	mRNA	3.413774	1.32E-07	9.22E-07
PI3	mRNA	7.139605	1.33E-07	9.26E-07
TNNT2	mRNA	4.384141	1.34E-07	9.34E-07
RPL39L	mRNA	2.249952	1.35E-07	9.38E-07
TGFA	mRNA	2.02432	1.41E-07	9.77E-07
COL8A1	mRNA	2.969533	1.41E-07	9.79E-07
NPHP1	mRNA	2.158416	1.44E-07	1.00E-06
CST1	mRNA	8.948132	1.45E-07	1.01E-06
TUBB6	mRNA	2.045984	1.47E-07	1.02E-06
NTN3	mRNA	-2.39544	1.48E-07	1.02E-06
LBP	mRNA	-3.42866	1.49E-07	1.03E-06
MN1	mRNA	2.774378	1.50E-07	1.04E-06
APLP1	mRNA	5.40302	1.51E-07	1.05E-06
TMC2	mRNA	4.888463	1.52E-07	1.05E-06
TMEM54	mRNA	2.829303	1.54E-07	1.06E-06
ADGRB3	mRNA	-2.23342	1.59E-07	1.09E-06
GJB4	mRNA	6.698542	1.61E-07	1.11E-06
CKMT1B	mRNA	7.632288	1.62E-07	1.12E-06
LONRF3	mRNA	-2.0167	1.63E-07	1.12E-06
FHAD1	mRNA	2.731991	1.66E-07	1.14E-06
ZP3	mRNA	2.38999	1.69E-07	1.16E-06
CDH6	mRNA	4.292154	1.74E-07	1.19E-06
AL845331.2	mRNA	-2.61717	1.75E-07	1.20E-06
HIST1H2BG	mRNA	3.793523	1.76E-07	1.21E-06
RTL9	mRNA	-2.95377	1.79E-07	1.23E-06
RSPO3	mRNA	-2.86369	1.80E-07	1.23E-06
C20orf204	mRNA	2.909233	1.84E-07	1.26E-06
HTRA3	mRNA	2.716871	1.85E-07	1.26E-06
ARMCX2	mRNA	2.262051	1.87E-07	1.28E-06
KCNG1	mRNA	4.387152	1.91E-07	1.30E-06
PHEX	mRNA	3.329753	1.91E-07	1.30E-06
SPEF1	mRNA	4.471963	1.94E-07	1.32E-06
PGM2L1	mRNA	2.193765	1.95E-07	1.32E-06
CD177	mRNA	4.914347	1.98E-07	1.34E-06
CTSC	mRNA	2.373189	1.98E-07	1.34E-06

BARX2	mRNA	5.739644	1.98E-07	1.35E-06
MROH2B	mRNA	-2.99642	1.99E-07	1.35E-06
CAPN6	mRNA	4.478606	2.02E-07	1.37E-06
RNF224	mRNA	4.27967	2.03E-07	1.37E-06
POU5F1	mRNA	2.752519	2.08E-07	1.41E-06
CHST10	mRNA	2.273016	2.13E-07	1.43E-06
IL17RD	mRNA	2.693969	2.13E-07	1.44E-06
MUC1	mRNA	4.863219	2.15E-07	1.45E-06
PITX1	mRNA	5.150051	2.18E-07	1.47E-06
PLA2G4D	mRNA	7.255536	2.19E-07	1.47E-06
SERPINC1	mRNA	-4.86861	2.20E-07	1.48E-06
OSBPL7	mRNA	2.188764	2.23E-07	1.50E-06
STX1A	mRNA	3.178417	2.34E-07	1.57E-06
DZANK1	mRNA	2.032406	2.36E-07	1.58E-06
10-Mar	mRNA	5.174926	2.37E-07	1.59E-06
GIPR	mRNA	3.385476	2.37E-07	1.59E-06
CBSL	mRNA	-3.70868	2.38E-07	1.59E-06
OXTR	mRNA	4.529125	2.42E-07	1.62E-06
XPNPEP2	mRNA	-4.38689	2.42E-07	1.62E-06
GLIS3	mRNA	2.211817	2.46E-07	1.64E-06
CHST13	mRNA	-2.34109	2.50E-07	1.67E-06
SERTM2	mRNA	-4.0998	2.50E-07	1.67E-06
LPCAT1	mRNA	2.366234	2.53E-07	1.69E-06
INHA	mRNA	7.153806	2.55E-07	1.70E-06
SYNGR1	mRNA	2.123996	2.58E-07	1.71E-06
RSPH14	mRNA	3.556256	2.60E-07	1.73E-06
C15orf48	mRNA	3.542462	2.65E-07	1.76E-06
C5orf46	mRNA	5.897063	2.65E-07	1.76E-06
CDKN2A	mRNA	3.348333	2.66E-07	1.76E-06
EPHB2	mRNA	3.697266	2.67E-07	1.77E-06
SLC12A2	mRNA	3.341767	2.68E-07	1.77E-06
UNC13A	mRNA	5.033963	2.69E-07	1.78E-06
AP1G2	mRNA	2.303274	2.70E-07	1.79E-06
LOXL2	mRNA	2.263633	2.74E-07	1.81E-06
VWA7	mRNA	2.317083	2.77E-07	1.83E-06
ENAH	mRNA	2.256869	2.82E-07	1.86E-06
CYP2W1	mRNA	3.991178	2.83E-07	1.87E-06
ALOX12B	mRNA	5.043157	2.88E-07	1.90E-06
HPDL	mRNA	3.502939	2.90E-07	1.91E-06
PPP1R3C	mRNA	-2.43787	2.91E-07	1.91E-06
CFAP74	mRNA	-2.60026	2.94E-07	1.93E-06
IGF1R	mRNA	2.679571	2.95E-07	1.94E-06
C22orf23	mRNA	2.231249	2.95E-07	1.94E-06
KRT7	mRNA	3.032703	2.99E-07	1.96E-06
ABCC11	mRNA	-2.81213	2.99E-07	1.96E-06
TMEM190	mRNA	5.87971	2.99E-07	1.96E-06
VCAN	mRNA	3.589389	3.01E-07	1.97E-06

RUNDC3A	mRNA	4.763583	3.05E-07	2.00E-06
CNNM1	mRNA	4.028979	3.06E-07	2.01E-06
RAB40A	mRNA	2.595227	3.12E-07	2.04E-06
LRRC55	mRNA	-2.49179	3.17E-07	2.07E-06
UNC5A	mRNA	4.984066	3.17E-07	2.07E-06
NAT14	mRNA	2.089028	3.21E-07	2.10E-06
FP565260.6	mRNA	-2.10951	3.21E-07	2.10E-06
MKRN2OS	mRNA	2.860104	3.22E-07	2.10E-06
CCDC40	mRNA	2.126386	3.23E-07	2.11E-06
SMIM5	mRNA	3.20084	3.24E-07	2.12E-06
CHRNB2	mRNA	5.555956	3.29E-07	2.14E-06
F2RL3	mRNA	2.593173	3.30E-07	2.15E-06
CDC20B	mRNA	9.532682	3.37E-07	2.19E-06
LRRN2	mRNA	2.951701	3.46E-07	2.25E-06
IL33	mRNA	-2.29442	3.48E-07	2.26E-06
SLC5A12	mRNA	-3.96732	3.49E-07	2.26E-06
PIP5KL1	mRNA	3.290339	3.53E-07	2.29E-06
RAB11FIP1	mRNA	2.299744	3.56E-07	2.31E-06
MMP7	mRNA	4.871955	3.62E-07	2.34E-06
RSPH1	mRNA	3.013158	3.65E-07	2.37E-06
SLC19A3	mRNA	-2.3984	3.69E-07	2.39E-06
ATP2C2	mRNA	3.71488	3.69E-07	2.39E-06
RASAL1	mRNA	4.016857	3.70E-07	2.39E-06
ASIC1	mRNA	3.106213	3.71E-07	2.39E-06
FABP6	mRNA	8.608307	3.73E-07	2.41E-06
CMTM3	mRNA	2.169768	3.81E-07	2.46E-06
SLC22A9	mRNA	-3.75286	3.84E-07	2.48E-06
SPEF2	mRNA	2.000333	3.86E-07	2.49E-06
GJB2	mRNA	-2.33605	3.99E-07	2.57E-06
DPF3	mRNA	-2.4152	3.99E-07	2.57E-06
KCNQ3	mRNA	3.501953	4.01E-07	2.58E-06
GFRA2	mRNA	-2.36403	4.01E-07	2.58E-06
B3GNT5	mRNA	2.427507	4.15E-07	2.66E-06
FAM167A	mRNA	3.291368	4.22E-07	2.70E-06
ICA1L	mRNA	2.277276	4.23E-07	2.71E-06
LRP8	mRNA	3.668519	4.28E-07	2.74E-06
PCDHB10	mRNA	3.181952	4.32E-07	2.76E-06
FMNL2	mRNA	2.584256	4.40E-07	2.80E-06
GUCA2A	mRNA	6.004103	4.43E-07	2.82E-06
TUSC3	mRNA	2.310935	4.78E-07	3.04E-06
APCDD1	mRNA	3.22143	4.79E-07	3.04E-06
TMEM246	mRNA	2.115893	4.93E-07	3.12E-06
ANGPTL4	mRNA	-2.05524	4.95E-07	3.13E-06
NEIL3	mRNA	3.122683	5.01E-07	3.17E-06
LXN	mRNA	2.156841	5.05E-07	3.19E-06
MFAP2	mRNA	3.038593	5.05E-07	3.19E-06
RAB34	mRNA	2.633353	5.06E-07	3.19E-06

EFNA4	mRNA	2.178354	5.06E-07	3.19E-06
KRTAP5-6	mRNA	-2.96514	5.06E-07	3.19E-06
MYO3A	mRNA	4.246065	5.08E-07	3.20E-06
ABCA12	mRNA	5.48554	5.17E-07	3.25E-06
HIST1H4E	mRNA	4.149167	5.20E-07	3.26E-06
CCDC189	mRNA	2.470348	5.25E-07	3.30E-06
DRAM1	mRNA	2.205747	5.33E-07	3.34E-06
SMARCD3	mRNA	2.04419	5.35E-07	3.36E-06
ESYT3	mRNA	3.022885	5.39E-07	3.38E-06
PDE7A	mRNA	2.314719	5.50E-07	3.45E-06
MEGF10	mRNA	-2.50445	5.53E-07	3.46E-06
VN1R1	mRNA	2.72568	5.53E-07	3.46E-06
MFSD4A	mRNA	2.649479	5.58E-07	3.49E-06
ISM1	mRNA	-2.14573	5.60E-07	3.50E-06
PDE1C	mRNA	4.543019	5.66E-07	3.53E-06
PTPN13	mRNA	2.528199	5.69E-07	3.55E-06
CST2	mRNA	4.048534	5.78E-07	3.61E-06
LETM2	mRNA	2.889849	5.88E-07	3.66E-06
ARHGAP8	mRNA	3.518104	6.15E-07	3.81E-06
SPACA6	mRNA	2.07839	6.20E-07	3.84E-06
CYP4Z1	mRNA	-2.56483	6.30E-07	3.90E-06
RBBP8NL	mRNA	5.471524	6.35E-07	3.93E-06
CLVS1	mRNA	5.882732	6.35E-07	3.93E-06
TMTC2	mRNA	2.005331	6.39E-07	3.95E-06
MAPK12	mRNA	2.778453	6.56E-07	4.05E-06
FAM184A	mRNA	-2.57308	6.57E-07	4.06E-06
FAM155A	mRNA	3.995574	6.59E-07	4.06E-06
MAP3K21	mRNA	2.208027	6.59E-07	4.07E-06
KCNK12	mRNA	4.056848	6.62E-07	4.08E-06
HOPX	mRNA	2.774018	6.62E-07	4.08E-06
PALM2-AKAP2	mRNA	-2.34905	6.65E-07	4.09E-06
RAP1GAP	mRNA	2.431616	6.65E-07	4.09E-06
CCDC65	mRNA	2.082482	6.75E-07	4.15E-06
B3GALT5	mRNA	3.840441	6.77E-07	4.16E-06
COL9A2	mRNA	3.991723	6.81E-07	4.18E-06
PRRX1	mRNA	3.608837	6.84E-07	4.20E-06
SLC22A15	mRNA	2.88239	6.87E-07	4.22E-06
GPAT3	mRNA	-2.53743	7.00E-07	4.28E-06
AURKA	mRNA	2.099264	7.07E-07	4.33E-06
EVC2	mRNA	2.22745	7.09E-07	4.33E-06
ALOX5	mRNA	3.062672	7.13E-07	4.36E-06
CRHR1	mRNA	7.907038	7.15E-07	4.37E-06
AGTR1	mRNA	-2.718	7.27E-07	4.43E-06
ZNF888	mRNA	3.465852	7.33E-07	4.47E-06
GPR160	mRNA	2.526888	7.42E-07	4.51E-06
PADI3	mRNA	9.475169	7.47E-07	4.54E-06
TFAP2C	mRNA	4.548935	7.55E-07	4.59E-06

ATP10A	mRNA	2.168977	7.62E-07	4.63E-06
DNER	mRNA	6.568385	7.65E-07	4.64E-06
AKR7A3	mRNA	-2.99244	7.75E-07	4.70E-06
TMC7	mRNA	2.794665	7.82E-07	4.74E-06
AGXT	mRNA	-4.72076	7.87E-07	4.76E-06
TMCC2	mRNA	2.582935	7.87E-07	4.76E-06
SEC14L6	mRNA	3.572048	7.96E-07	4.81E-06
CNFN	mRNA	3.373539	8.07E-07	4.87E-06
RHOF	mRNA	2.595473	8.12E-07	4.89E-06
VTCN1	mRNA	4.791956	8.15E-07	4.91E-06
PTPN5	mRNA	4.153201	8.32E-07	5.01E-06
EGFL6	mRNA	4.28273	8.40E-07	5.06E-06
SLC24A3	mRNA	2.937414	8.40E-07	5.06E-06
MAP1A	mRNA	2.12262	8.42E-07	5.06E-06
FOS	mRNA	-2.21712	8.45E-07	5.08E-06
SPHK1	mRNA	3.342812	8.50E-07	5.11E-06
ANO4	mRNA	3.391649	8.51E-07	5.11E-06
STMN3	mRNA	2.500997	8.59E-07	5.15E-06
KIAA1024	mRNA	2.964266	8.75E-07	5.24E-06
TNNI3	mRNA	7.376371	8.76E-07	5.24E-06
SH3D21	mRNA	2.156059	8.80E-07	5.26E-06
TAS2R5	mRNA	3.065945	8.89E-07	5.31E-06
LDOC1	mRNA	2.644671	8.94E-07	5.34E-06
HNF1B	mRNA	2.719095	8.98E-07	5.35E-06
DNAJC6	mRNA	3.59474	9.02E-07	5.37E-06
ARL4C	mRNA	2.530318	9.20E-07	5.47E-06
BIK	mRNA	3.257153	9.31E-07	5.53E-06
C8G	mRNA	-2.51155	9.36E-07	5.56E-06
GPC4	mRNA	2.347814	9.45E-07	5.61E-06
PI15	mRNA	3.695807	9.46E-07	5.61E-06
CAPS	mRNA	2.46693	9.47E-07	5.61E-06
SEMA3C	mRNA	3.93332	9.50E-07	5.63E-06
TINAG	mRNA	8.556938	9.63E-07	5.70E-06
CLDN14	mRNA	-2.44242	9.68E-07	5.73E-06
LARP6	mRNA	2.17449	9.84E-07	5.82E-06
SLC7A9	mRNA	-2.67703	9.86E-07	5.83E-06
NT5DC4	mRNA	5.214536	9.87E-07	5.83E-06
GRP	mRNA	6.417772	9.94E-07	5.87E-06
PNCK	mRNA	4.856764	1.00E-06	5.90E-06
HPN	mRNA	-2.12622	1.04E-06	6.13E-06
IL10	mRNA	-2.45761	1.06E-06	6.20E-06
GNAO1	mRNA	-2.86405	1.07E-06	6.27E-06
FOLR1	mRNA	5.168111	1.07E-06	6.29E-06
ADGRL1	mRNA	2.20825	1.08E-06	6.31E-06
SALL1	mRNA	-2.34273	1.08E-06	6.31E-06
KLHL29	mRNA	2.035216	1.09E-06	6.37E-06
ENPP7	mRNA	-3.54783	1.09E-06	6.40E-06

BHLHE41	mRNA	2.639152	1.09E-06	6.40E-06
KCP	mRNA	3.556284	1.13E-06	6.59E-06
GAREM2	mRNA	2.572415	1.15E-06	6.69E-06
PGC	mRNA	11.49528	1.16E-06	6.76E-06
SLC9C1	mRNA	3.97149	1.20E-06	6.98E-06
ARRDC2	mRNA	2.166931	1.20E-06	7.00E-06
FUT2	mRNA	3.941994	1.21E-06	7.06E-06
CXCL2	mRNA	-2.01623	1.24E-06	7.18E-06
MORN3	mRNA	3.125426	1.25E-06	7.24E-06
ALPL	mRNA	-2.61106	1.26E-06	7.30E-06
HAPLN3	mRNA	2.638484	1.26E-06	7.32E-06
LRRC31	mRNA	-2.95519	1.27E-06	7.34E-06
LAMP3	mRNA	2.891166	1.28E-06	7.40E-06
SLC22A2	mRNA	-2.72474	1.29E-06	7.49E-06
KIAA1614	mRNA	2.76938	1.33E-06	7.67E-06
TNFRSF21	mRNA	2.250379	1.34E-06	7.71E-06
TRIM16	mRNA	2.08324	1.34E-06	7.74E-06
RSPO2	mRNA	-2.65472	1.34E-06	7.75E-06
PAX9	mRNA	4.448327	1.36E-06	7.81E-06
REG1A	mRNA	9.224364	1.40E-06	8.04E-06
FADS6	mRNA	-3.44876	1.41E-06	8.08E-06
YBX3	mRNA	2.005951	1.41E-06	8.10E-06
EXTL1	mRNA	3.270256	1.42E-06	8.16E-06
SPAG17	mRNA	5.154929	1.44E-06	8.24E-06
FIBCD1	mRNA	5.469186	1.44E-06	8.28E-06
NKPD1	mRNA	3.434614	1.45E-06	8.30E-06
LRRC75A	mRNA	2.217629	1.47E-06	8.42E-06
SOX11	mRNA	5.322107	1.48E-06	8.49E-06
PCDHB8	mRNA	4.47093	1.49E-06	8.51E-06
C15orf65	mRNA	2.038972	1.49E-06	8.51E-06
GPR84	mRNA	3.439142	1.49E-06	8.53E-06
ZNF365	mRNA	4.531268	1.50E-06	8.57E-06
C2CD4D	mRNA	2.853054	1.51E-06	8.60E-06
ATOH7	mRNA	-2.37264	1.51E-06	8.60E-06
GNRH2	mRNA	-2.12629	1.52E-06	8.66E-06
ARL14	mRNA	3.525434	1.56E-06	8.86E-06
PDP1	mRNA	2.116655	1.57E-06	8.94E-06
MINDY4	mRNA	2.183286	1.58E-06	9.00E-06
CDH3	mRNA	5.530509	1.59E-06	9.05E-06
PTGES	mRNA	3.363554	1.61E-06	9.16E-06
MAPK8IP2	mRNA	3.626346	1.63E-06	9.27E-06
AMN	mRNA	-2.68355	1.65E-06	9.37E-06
FDCSP	mRNA	10.57519	1.66E-06	9.44E-06
COL2A1	mRNA	-3.68588	1.70E-06	9.60E-06
TBC1D30	mRNA	2.25578	1.70E-06	9.61E-06
ABLIM2	mRNA	2.534421	1.72E-06	9.70E-06
SLC22A3	mRNA	-2.57215	1.73E-06	9.75E-06

MITF	mRNA	2.699453	1.73E-06	9.77E-06
EGLN3	mRNA	3.836463	1.74E-06	9.80E-06
SLC25A48	mRNA	-2.7819	1.75E-06	9.86E-06
CES4A	mRNA	-2.63537	1.76E-06	9.94E-06
CEACAM7	mRNA	7.480633	1.77E-06	9.96E-06
AQP1	mRNA	3.526392	1.78E-06	1.00E-05
KLF5	mRNA	3.173196	1.80E-06	1.01E-05
NEB	mRNA	4.774535	1.81E-06	1.02E-05
MFSD6L	mRNA	5.128728	1.81E-06	1.02E-05
TPM2	mRNA	2.274904	1.82E-06	1.02E-05
MRC1	mRNA	-2.59189	1.86E-06	1.04E-05
BCL11A	mRNA	3.534834	1.86E-06	1.05E-05
ISL2	mRNA	4.10317	1.87E-06	1.05E-05
KCND3	mRNA	-2.03488	1.88E-06	1.05E-05
HIST1H1E	mRNA	3.801845	1.90E-06	1.06E-05
KRT15	mRNA	4.623049	1.91E-06	1.07E-05
RNF223	mRNA	4.280296	1.92E-06	1.08E-05
C1orf100	mRNA	3.618497	1.94E-06	1.08E-05
SPOCD1	mRNA	4.570123	1.98E-06	1.10E-05
CYP27B1	mRNA	2.911174	2.00E-06	1.12E-05
ASAP2	mRNA	2.241078	2.02E-06	1.13E-05
APOBEC3C	mRNA	2.066952	2.02E-06	1.13E-05
FAM110A	mRNA	2.024184	2.04E-06	1.13E-05
NECTIN1	mRNA	2.240549	2.04E-06	1.13E-05
PRSS27	mRNA	2.257625	2.05E-06	1.14E-05
SLC6A9	mRNA	2.372207	2.05E-06	1.14E-05
CREG2	mRNA	4.383619	2.06E-06	1.15E-05
SLC7A6	mRNA	2.199863	2.07E-06	1.15E-05
VAV3	mRNA	3.173388	2.08E-06	1.15E-05
GINS3	mRNA	2.448434	2.09E-06	1.16E-05
CACNA1F	mRNA	3.408923	2.09E-06	1.16E-05
C10orf95	mRNA	2.637875	2.10E-06	1.16E-05
KRT81	mRNA	8.561223	2.16E-06	1.19E-05
KLHDC8A	mRNA	3.950791	2.16E-06	1.20E-05
FOXA3	mRNA	-2.23708	2.17E-06	1.20E-05
FNDC10	mRNA	2.786034	2.21E-06	1.22E-05
AKAIN1	mRNA	-3.28886	2.21E-06	1.22E-05
ARHGEF26	mRNA	-2.00114	2.22E-06	1.22E-05
LTBP2	mRNA	2.133228	2.23E-06	1.23E-05
ARHGAP22	mRNA	3.163521	2.23E-06	1.23E-05
ATP8A2	mRNA	5.073241	2.24E-06	1.23E-05
SAMD15	mRNA	2.595108	2.24E-06	1.23E-05
HIST1H3D	mRNA	3.274882	2.24E-06	1.23E-05
MCC	mRNA	-2.17477	2.25E-06	1.24E-05
DBNDD1	mRNA	2.018747	2.25E-06	1.24E-05
C6orf223	mRNA	4.319973	2.26E-06	1.24E-05
TMPRSS13	mRNA	4.191699	2.26E-06	1.24E-05

CATSPERB	mRNA	2.52886	2.26E-06	1.24E-05
TEX22	mRNA	2.476103	2.30E-06	1.26E-05
ARHGAP11A	mRNA	2.157441	2.30E-06	1.26E-05
LINGO1	mRNA	3.579781	2.33E-06	1.28E-05
CHRNA5	mRNA	3.121293	2.36E-06	1.29E-05
MMP12	mRNA	6.306459	2.37E-06	1.29E-05
PAQR8	mRNA	2.683113	2.40E-06	1.31E-05
CEACAM6	mRNA	6.123798	2.41E-06	1.31E-05
CBX2	mRNA	3.020301	2.41E-06	1.31E-05
FOXO6	mRNA	2.734765	2.41E-06	1.32E-05
KCNK9	mRNA	6.864089	2.46E-06	1.34E-05
FOXN1	mRNA	5.864448	2.49E-06	1.36E-05
TNFRSF10C	mRNA	2.615575	2.50E-06	1.36E-05
FERMT1	mRNA	3.410933	2.54E-06	1.38E-05
LRRN3	mRNA	-2.03152	2.57E-06	1.40E-05
TM4SF1	mRNA	2.204066	2.62E-06	1.42E-05
HBA1	mRNA	-3.21118	2.66E-06	1.44E-05
PPP1R14D	mRNA	5.716951	2.68E-06	1.45E-05
RHBG	mRNA	-2.78296	2.69E-06	1.46E-05
TRAM1L1	mRNA	3.352594	2.73E-06	1.48E-05
DMBT1	mRNA	7.796314	2.74E-06	1.48E-05
SLC26A9	mRNA	8.537247	2.76E-06	1.49E-05
BEST3	mRNA	6.276299	2.77E-06	1.49E-05
NUDT17	mRNA	2.18015	2.81E-06	1.52E-05
PPP2R3A	mRNA	2.33481	2.82E-06	1.52E-05
MCOLN3	mRNA	4.22169	2.88E-06	1.55E-05
FAM131C	mRNA	4.185039	2.88E-06	1.55E-05
PMAIP1	mRNA	3.167316	2.89E-06	1.55E-05
STRC	mRNA	3.555099	2.92E-06	1.57E-05
ALDH3B2	mRNA	7.362355	2.94E-06	1.58E-05
SLC2A1	mRNA	4.462775	2.94E-06	1.58E-05
RHBDL2	mRNA	2.992844	2.95E-06	1.58E-05
EPPK1	mRNA	3.224791	2.97E-06	1.59E-05
KCNH3	mRNA	4.423121	2.97E-06	1.59E-05
GRM5	mRNA	9.294907	2.97E-06	1.59E-05
ATP6V1B1	mRNA	3.811484	2.99E-06	1.60E-05
GHRHR	mRNA	4.977842	3.00E-06	1.60E-05
RASSF6	mRNA	2.641453	3.03E-06	1.62E-05
NEFL	mRNA	7.21471	3.07E-06	1.64E-05
CDK5R2	mRNA	5.744267	3.07E-06	1.64E-05
AMH	mRNA	4.187362	3.08E-06	1.65E-05
TMEM156	mRNA	3.148261	3.11E-06	1.66E-05
EFNA5	mRNA	3.767053	3.11E-06	1.66E-05
RNF182	mRNA	5.011763	3.16E-06	1.68E-05
B4GALNT1	mRNA	2.674054	3.24E-06	1.72E-05
PLG	mRNA	-4.85087	3.27E-06	1.74E-05
SRPX2	mRNA	2.747499	3.29E-06	1.75E-05

CAPN9	mRNA	4.68041	3.30E-06	1.75E-05
UGT1A10	mRNA	7.921222	3.33E-06	1.76E-05
SERHL2	mRNA	2.091961	3.34E-06	1.77E-05
CYBA	mRNA	2.309495	3.38E-06	1.79E-05
RIN1	mRNA	2.127388	3.39E-06	1.79E-05
PNMA6A	mRNA	-2.26862	3.40E-06	1.80E-05
ZCCHC12	mRNA	3.72668	3.42E-06	1.81E-05
S100A14	mRNA	3.173841	3.46E-06	1.83E-05
TF	mRNA	-4.25111	3.47E-06	1.84E-05
PRICKLE1	mRNA	2.417416	3.49E-06	1.84E-05
PROM2	mRNA	4.247721	3.50E-06	1.85E-05
AC068946.1	mRNA	2.636614	3.58E-06	1.89E-05
LDLRAD1	mRNA	4.72554	3.60E-06	1.90E-05
ANKRD2	mRNA	4.517871	3.63E-06	1.91E-05
MCF2L2	mRNA	2.869532	3.65E-06	1.92E-05
ENDOD1	mRNA	2.09976	3.66E-06	1.93E-05
C3orf80	mRNA	2.723261	3.70E-06	1.94E-05
APOBEC3B	mRNA	2.699901	3.73E-06	1.96E-05
VSTM5	mRNA	4.678573	3.76E-06	1.97E-05
MTHFD2	mRNA	2.329826	3.77E-06	1.97E-05
LMX1B	mRNA	4.900813	3.81E-06	1.99E-05
C2orf70	mRNA	4.164801	3.87E-06	2.02E-05
NXF3	mRNA	-2.39969	3.88E-06	2.03E-05
TCTEX1D2	mRNA	2.025747	3.91E-06	2.04E-05
VWA5B2	mRNA	3.762286	3.92E-06	2.04E-05
TMEM151A	mRNA	5.035699	3.93E-06	2.05E-05
FJX1	mRNA	2.279326	3.97E-06	2.07E-05
ZFHX4	mRNA	-2.14248	4.03E-06	2.09E-05
ANKRD1	mRNA	4.636173	4.04E-06	2.10E-05
GALNT6	mRNA	2.595723	4.07E-06	2.11E-05
HAVCR1	mRNA	6.683974	4.07E-06	2.11E-05
FOXS1	mRNA	2.539686	4.09E-06	2.12E-05
TNFSF9	mRNA	2.836235	4.11E-06	2.13E-05
OIP5	mRNA	2.105248	4.12E-06	2.13E-05
CCNO	mRNA	3.649263	4.16E-06	2.16E-05
FOXD4L1	mRNA	3.537177	4.16E-06	2.16E-05
MMP10	mRNA	4.915218	4.21E-06	2.18E-05
CA9	mRNA	5.792174	4.24E-06	2.19E-05
FGF12	mRNA	3.387791	4.24E-06	2.19E-05
DSC3	mRNA	5.779943	4.24E-06	2.19E-05
CDH17	mRNA	6.757542	4.36E-06	2.25E-05
NMNAT2	mRNA	3.473338	4.37E-06	2.25E-05
SFTPB	mRNA	-3.25672	4.45E-06	2.29E-05
PAX8	mRNA	3.066733	4.53E-06	2.33E-05
DOK7	mRNA	2.602451	4.56E-06	2.35E-05
SCX	mRNA	2.724498	4.62E-06	2.37E-05
LRRTM4	mRNA	-3.37439	4.64E-06	2.38E-05

GDPD4	mRNA	-2.51765	4.66E-06	2.39E-05
ERICH3	mRNA	-2.61725	4.67E-06	2.39E-05
TTYH1	mRNA	5.118762	4.73E-06	2.42E-05
SERPINA5	mRNA	-2.2706	4.75E-06	2.43E-05
CA1	mRNA	-3.44641	4.75E-06	2.43E-05
PLA2G2A	mRNA	-3.05737	4.80E-06	2.45E-05
TMOD1	mRNA	-2.24092	4.81E-06	2.46E-05
MUC20	mRNA	3.165519	4.83E-06	2.46E-05
SPDEF	mRNA	5.783935	4.83E-06	2.46E-05
APOA5	mRNA	-4.64714	4.84E-06	2.47E-05
CD300LG	mRNA	-3.13791	4.88E-06	2.49E-05
TM4SF5	mRNA	-2.39558	4.96E-06	2.53E-05
SLC6A17	mRNA	5.005013	5.15E-06	2.62E-05
DNAH10	mRNA	2.714607	5.18E-06	2.63E-05
GPRC5A	mRNA	5.1702	5.18E-06	2.63E-05
DNAH10OS	mRNA	2.472592	5.20E-06	2.64E-05
PRRX2	mRNA	3.794445	5.21E-06	2.64E-05
ANKS4B	mRNA	-2.3095	5.23E-06	2.65E-05
LRRC73	mRNA	3.049169	5.24E-06	2.66E-05
CCDC160	mRNA	3.369851	5.29E-06	2.68E-05
KIAA1324	mRNA	4.224399	5.38E-06	2.72E-05
SGO1	mRNA	2.316636	5.42E-06	2.74E-05
GRHL2	mRNA	3.735785	5.42E-06	2.74E-05
MYOM3	mRNA	6.703633	5.57E-06	2.81E-05
SCGB1D2	mRNA	8.631377	5.59E-06	2.82E-05
HSH2D	mRNA	2.557903	5.65E-06	2.85E-05
ZMYND10	mRNA	2.856841	5.68E-06	2.86E-05
SPOCK1	mRNA	3.962974	5.69E-06	2.87E-05
C18orf54	mRNA	2.215781	5.86E-06	2.94E-05
EVPL	mRNA	5.733992	5.94E-06	2.98E-05
CLDN18	mRNA	8.170066	5.95E-06	2.98E-05
PAEP	mRNA	8.669746	6.02E-06	3.01E-05
HHIP	mRNA	-2.77656	6.08E-06	3.04E-05
SLC9A2	mRNA	4.970542	6.13E-06	3.06E-05
CUZD1	mRNA	2.806886	6.16E-06	3.07E-05
TMEM72	mRNA	4.943938	6.17E-06	3.08E-05
LMX1A	mRNA	-3.64309	6.19E-06	3.09E-05
NUAK2	mRNA	2.515832	6.24E-06	3.11E-05
UPK3A	mRNA	4.582159	6.26E-06	3.12E-05
TECTB	mRNA	-2.89298	6.31E-06	3.14E-05
KRT17	mRNA	6.117551	6.34E-06	3.15E-05
TCP10L2	mRNA	-3.39585	6.40E-06	3.18E-05
DKK1	mRNA	7.126175	6.41E-06	3.19E-05
SYCE1L	mRNA	2.25702	6.41E-06	3.19E-05
KIAA0319	mRNA	4.259218	6.48E-06	3.22E-05
XKR5	mRNA	4.274885	6.53E-06	3.24E-05
AFP	mRNA	-3.16125	6.54E-06	3.24E-05

IRX2	mRNA	6.635744	6.57E-06	3.26E-05
PCOLCE2	mRNA	-2.42437	6.65E-06	3.30E-05
LMO1	mRNA	5.646029	6.66E-06	3.30E-05
EDA2R	mRNA	2.534679	6.75E-06	3.34E-05
CGB7	mRNA	4.954207	6.75E-06	3.34E-05
SCN3B	mRNA	3.050112	6.80E-06	3.36E-05
PDPN	mRNA	2.953952	6.88E-06	3.40E-05
GSDMC	mRNA	3.85465	6.88E-06	3.40E-05
SLC22A12	mRNA	-3.45186	6.91E-06	3.41E-05
ROR1	mRNA	2.79819	6.94E-06	3.42E-05
CDH11	mRNA	2.607274	7.02E-06	3.46E-05
CCL15-CCL14	mRNA	-2.73378	7.05E-06	3.47E-05
QSOX1	mRNA	2.379473	7.05E-06	3.47E-05
FGF21	mRNA	-3.81809	7.08E-06	3.48E-05
CACNG4	mRNA	5.790869	7.11E-06	3.50E-05
DNASE1	mRNA	2.394949	7.13E-06	3.51E-05
WTIP	mRNA	2.178956	7.16E-06	3.52E-05
CNIH2	mRNA	2.058929	7.27E-06	3.57E-05
ZIC5	mRNA	5.960169	7.29E-06	3.58E-05
TYRP1	mRNA	3.969583	7.38E-06	3.62E-05
CSPP1	mRNA	2.168762	7.40E-06	3.63E-05
HSPB8	mRNA	2.704394	7.49E-06	3.67E-05
LIPJ	mRNA	-2.17134	7.53E-06	3.69E-05
HIST1H2BF	mRNA	4.267042	7.57E-06	3.71E-05
ANKRD35	mRNA	-2.0622	7.60E-06	3.72E-05
PACRG	mRNA	-2.05027	7.61E-06	3.72E-05
LIPM	mRNA	4.405379	7.62E-06	3.73E-05
FAR2	mRNA	2.19533	7.67E-06	3.75E-05
SNAP91	mRNA	7.090589	7.70E-06	3.76E-05
ANKRD22	mRNA	3.365272	7.89E-06	3.85E-05
ADCY2	mRNA	4.989724	7.96E-06	3.88E-05
USP2	mRNA	-2.00806	7.97E-06	3.89E-05
GOLGA8M	mRNA	-2.08879	8.19E-06	3.99E-05
COL1A1	mRNA	2.014095	8.28E-06	4.03E-05
HOXC9	mRNA	6.509516	8.29E-06	4.04E-05
NANOS1	mRNA	3.27779	8.33E-06	4.05E-05
PLA2G4E	mRNA	4.398001	8.50E-06	4.13E-05
PCDHGA1	mRNA	3.440687	8.51E-06	4.14E-05
ENO2	mRNA	2.79542	8.53E-06	4.14E-05
MEX3B	mRNA	2.12842	8.62E-06	4.18E-05
GPR63	mRNA	2.987479	8.65E-06	4.19E-05
ZAN	mRNA	-2.68586	8.73E-06	4.23E-05
INSL3	mRNA	2.918879	8.75E-06	4.24E-05
HOXC6	mRNA	5.233286	8.79E-06	4.26E-05
CAPN8	mRNA	4.72773	8.79E-06	4.26E-05
4-Mar	mRNA	4.315593	8.90E-06	4.31E-05
HOXB13	mRNA	10.17083	8.93E-06	4.32E-05

SIX2	mRNA	4.671195	8.94E-06	4.32E-05
GPR19	mRNA	3.114927	9.07E-06	4.38E-05
GPC2	mRNA	2.779746	9.11E-06	4.40E-05
SCAMP5	mRNA	2.607607	9.12E-06	4.40E-05
LRRC36	mRNA	3.167763	9.13E-06	4.40E-05
HRH1	mRNA	2.523669	9.35E-06	4.51E-05
CHST3	mRNA	2.525027	9.37E-06	4.52E-05
HSD3B2	mRNA	-2.97328	9.46E-06	4.55E-05
LOXL1	mRNA	2.017363	9.48E-06	4.56E-05
LIF	mRNA	3.006637	9.48E-06	4.56E-05
ADAMDEC1	mRNA	3.615088	9.56E-06	4.59E-05
PCDHB13	mRNA	2.799493	9.60E-06	4.61E-05
TMPRSS4	mRNA	7.144247	9.62E-06	4.62E-05
UCHL1	mRNA	3.205769	9.70E-06	4.65E-05
TERT	mRNA	7.051414	9.76E-06	4.68E-05
SLC5A8	mRNA	7.805997	9.77E-06	4.68E-05
IL20RA	mRNA	3.404076	9.89E-06	4.73E-05
AANAT	mRNA	3.823502	1.01E-05	4.83E-05
EPHA6	mRNA	3.679111	1.01E-05	4.84E-05
CEACAM5	mRNA	9.690035	1.02E-05	4.86E-05
JHY	mRNA	2.151182	1.02E-05	4.89E-05
NLGN1	mRNA	5.086854	1.03E-05	4.90E-05
KSR2	mRNA	3.3381	1.03E-05	4.93E-05
KIAA1211L	mRNA	2.128096	1.04E-05	4.93E-05
ZNF233	mRNA	2.331596	1.10E-05	5.20E-05
SEMA3E	mRNA	4.164922	1.11E-05	5.25E-05
MYRFL	mRNA	3.291982	1.11E-05	5.29E-05
B3GALT1	mRNA	5.492318	1.12E-05	5.29E-05
CASC1	mRNA	2.841038	1.13E-05	5.33E-05
CEP126	mRNA	2.206064	1.14E-05	5.38E-05
FAM84A	mRNA	2.452756	1.14E-05	5.41E-05
CKLF-CMTM1	mRNA	2.33538	1.14E-05	5.41E-05
SCIN	mRNA	3.581353	1.15E-05	5.42E-05
SLC22A4	mRNA	2.051982	1.16E-05	5.46E-05
XYLT1	mRNA	2.407087	1.16E-05	5.48E-05
REN	mRNA	-2.27677	1.17E-05	5.52E-05
HMGA2	mRNA	5.601991	1.17E-05	5.52E-05
ERFE	mRNA	2.649752	1.18E-05	5.55E-05
FZD10	mRNA	5.298497	1.18E-05	5.55E-05
REEP2	mRNA	2.719572	1.18E-05	5.56E-05
MMP1	mRNA	4.21318	1.18E-05	5.58E-05
RET	mRNA	-2.16807	1.19E-05	5.58E-05
TREX2	mRNA	2.300845	1.19E-05	5.60E-05
KCNG3	mRNA	5.98971	1.19E-05	5.60E-05
LAT2	mRNA	2.107942	1.21E-05	5.68E-05
RUFY4	mRNA	3.012152	1.22E-05	5.74E-05
TP73	mRNA	2.750723	1.24E-05	5.80E-05

UBXN10	mRNA	-2.2734	1.24E-05	5.81E-05
UGT8	mRNA	5.08338	1.25E-05	5.85E-05
SPINK1	mRNA	5.526167	1.28E-05	6.00E-05
TMPRSS7	mRNA	4.006696	1.29E-05	6.03E-05
ART5	mRNA	-2.17053	1.29E-05	6.03E-05
PIF1	mRNA	2.387754	1.29E-05	6.05E-05
CKB	mRNA	2.533451	1.29E-05	6.05E-05
ERP27	mRNA	3.266412	1.29E-05	6.05E-05
TMEM253	mRNA	3.298345	1.30E-05	6.06E-05
MYCBPAP	mRNA	3.10713	1.30E-05	6.08E-05
IL18	mRNA	3.225849	1.30E-05	6.09E-05
PROM1	mRNA	3.428796	1.31E-05	6.10E-05
NBEA	mRNA	2.109309	1.31E-05	6.10E-05
FAM19A5	mRNA	2.872588	1.32E-05	6.14E-05
KHDC1	mRNA	3.374474	1.33E-05	6.19E-05
NMU	mRNA	7.613363	1.33E-05	6.21E-05
CCNJL	mRNA	3.077186	1.34E-05	6.24E-05
ACOT11	mRNA	2.548343	1.38E-05	6.40E-05
NETO2	mRNA	2.856124	1.39E-05	6.47E-05
SLCO1A2	mRNA	-3.25	1.39E-05	6.48E-05
ADAM22	mRNA	2.470756	1.40E-05	6.50E-05
PKP1	mRNA	4.786291	1.42E-05	6.57E-05
PCDHB14	mRNA	2.204081	1.42E-05	6.57E-05
ISYNA1	mRNA	2.323486	1.44E-05	6.65E-05
ROPN1B	mRNA	-2.29518	1.45E-05	6.70E-05
KLK1	mRNA	6.751986	1.46E-05	6.73E-05
CRABP2	mRNA	3.471421	1.47E-05	6.79E-05
GCM1	mRNA	5.313155	1.47E-05	6.79E-05
TRAPPC3L	mRNA	-2.17237	1.48E-05	6.83E-05
GLYATL3	mRNA	-4.01898	1.51E-05	6.95E-05
SP6	mRNA	5.153977	1.51E-05	6.98E-05
AATK	mRNA	2.340703	1.52E-05	7.01E-05
CSMD2	mRNA	4.401498	1.54E-05	7.12E-05
LHFPL3	mRNA	5.016879	1.59E-05	7.30E-05
PTGFR	mRNA	3.334866	1.59E-05	7.32E-05
LY6H	mRNA	3.073514	1.59E-05	7.33E-05
GABRE	mRNA	2.139567	1.60E-05	7.35E-05
KLHL35	mRNA	3.314593	1.60E-05	7.37E-05
PKHD1	mRNA	2.737785	1.61E-05	7.39E-05
CBLN1	mRNA	-2.32551	1.61E-05	7.41E-05
NANOS3	mRNA	2.661308	1.62E-05	7.44E-05
VSIG10L	mRNA	2.594048	1.63E-05	7.48E-05
FAM109B	mRNA	2.078275	1.64E-05	7.53E-05
RANBP17	mRNA	2.696478	1.64E-05	7.53E-05
ELFN2	mRNA	3.184946	1.65E-05	7.57E-05
LHFPL5	mRNA	5.443291	1.66E-05	7.58E-05
HMGCLL1	mRNA	-2.44361	1.66E-05	7.59E-05

SLC13A1	mRNA	6.452143	1.66E-05	7.60E-05
CEMIP	mRNA	3.171322	1.67E-05	7.64E-05
ATP2B2	mRNA	-2.719	1.67E-05	7.65E-05
KLK11	mRNA	5.770758	1.68E-05	7.68E-05
SPNS2	mRNA	2.320813	1.73E-05	7.90E-05
SMCO2	mRNA	2.864277	1.78E-05	8.11E-05
ACRV1	mRNA	2.533205	1.78E-05	8.12E-05
HIST1H3E	mRNA	2.249341	1.79E-05	8.16E-05
PRRT2	mRNA	2.442658	1.80E-05	8.19E-05
DSCAML1	mRNA	3.141311	1.81E-05	8.22E-05
BPIFB4	mRNA	-4.27208	1.82E-05	8.28E-05
DIRAS1	mRNA	4.19547	1.83E-05	8.29E-05
ACMSD	mRNA	-2.26522	1.84E-05	8.34E-05
SNURF	mRNA	2.455266	1.88E-05	8.52E-05
MAP2	mRNA	2.385705	1.90E-05	8.62E-05
WFDC2	mRNA	3.855744	1.90E-05	8.62E-05
GCNT4	mRNA	2.227426	1.99E-05	9.00E-05
WNT2B	mRNA	3.342897	2.00E-05	9.01E-05
HIST1H3J	mRNA	5.429395	2.04E-05	9.17E-05
HAS3	mRNA	2.975363	2.05E-05	9.23E-05
FXYD2	mRNA	3.868858	2.08E-05	9.33E-05
NDRG4	mRNA	4.627168	2.08E-05	9.34E-05
ANXA13	mRNA	3.448703	2.08E-05	9.35E-05
S100A1	mRNA	4.201015	2.09E-05	9.39E-05
ACTBL2	mRNA	8.886489	2.11E-05	9.46E-05
CST6	mRNA	5.173099	2.11E-05	9.46E-05
FER1L5	mRNA	3.949888	2.12E-05	9.48E-05
C7	mRNA	-2.26898	2.12E-05	9.51E-05
WNT10A	mRNA	3.841371	2.13E-05	9.53E-05
LPA	mRNA	-4.35381	2.13E-05	9.53E-05
PCDHB16	mRNA	2.637471	2.13E-05	9.53E-05
RAB38	mRNA	2.226325	2.16E-05	9.65E-05
A4GALT	mRNA	2.085628	2.16E-05	9.65E-05
TPPP3	mRNA	3.132972	2.18E-05	9.74E-05
TDRD5	mRNA	5.650243	2.19E-05	9.74E-05
KYNU	mRNA	-2.01789	2.20E-05	9.80E-05
ARL9	mRNA	3.565204	2.23E-05	9.94E-05
C5orf38	mRNA	6.768103	2.27E-05	0.000101
RASD2	mRNA	2.565467	2.29E-05	0.000102
TMEM132B	mRNA	3.488465	2.29E-05	0.000102
PLAU	mRNA	2.72942	2.30E-05	0.000102
SPIB	mRNA	4.662152	2.30E-05	0.000102
ZDHHC19	mRNA	-2.68484	2.32E-05	0.000103
MEX3A	mRNA	2.794876	2.33E-05	0.000103
PCDHB9	mRNA	2.951562	2.35E-05	0.000104
ATG9B	mRNA	2.27644	2.39E-05	0.000106
PLEKHS1	mRNA	6.241169	2.40E-05	0.000106

CCDC114	mRNA	3.461451	2.40E-05	0.000106
ERVMER34-1	mRNA	3.421516	2.42E-05	0.000107
SMIM32	mRNA	5.109839	2.43E-05	0.000107
COX6B2	mRNA	5.134674	2.44E-05	0.000108
KCNH6	mRNA	9.540578	2.45E-05	0.000108
AC244517.6	mRNA	5.54608	2.46E-05	0.000108
PRAMEF33	mRNA	-3.95317	2.46E-05	0.000109
SOAT2	mRNA	-2.5447	2.47E-05	0.000109
GSTA2	mRNA	-4.48028	2.49E-05	0.00011
FOSB	mRNA	-2.53741	2.49E-05	0.00011
STK33	mRNA	3.275396	2.50E-05	0.00011
SCD5	mRNA	2.884829	2.53E-05	0.000111
SLC4A9	mRNA	3.220397	2.53E-05	0.000111
TMPRSS6	mRNA	-2.37802	2.55E-05	0.000112
CCND2	mRNA	2.695078	2.58E-05	0.000113
FZD2	mRNA	2.740351	2.58E-05	0.000113
EPHX3	mRNA	3.257425	2.59E-05	0.000114
BHLHA9	mRNA	-2.94479	2.61E-05	0.000115
PLCB4	mRNA	2.605311	2.64E-05	0.000116
FAM90A1	mRNA	2.965845	2.65E-05	0.000116
IGSF9B	mRNA	3.925993	2.71E-05	0.000119
TMSB15A	mRNA	2.710106	2.72E-05	0.000119
DPEP1	mRNA	5.180994	2.74E-05	0.00012
ZNF385C	mRNA	2.232442	2.74E-05	0.00012
ZNF286B	mRNA	2.215141	2.78E-05	0.000121
MRAP2	mRNA	3.140171	2.79E-05	0.000122
PSCA	mRNA	7.471973	2.79E-05	0.000122
MCUB	mRNA	2.031521	2.81E-05	0.000123
DKKL1	mRNA	2.859956	2.82E-05	0.000123
PCSK1N	mRNA	5.397626	2.82E-05	0.000123
AC092042.3	mRNA	-2.53513	2.84E-05	0.000123
ADAM12	mRNA	2.747843	2.84E-05	0.000124
ERVFRD-1	mRNA	-2.45095	2.87E-05	0.000125
SLFN12	mRNA	2.000145	2.93E-05	0.000127
IGF2BP3	mRNA	4.15114	2.93E-05	0.000127
SYT16	mRNA	5.311112	2.95E-05	0.000128
HOXC4	mRNA	3.886588	2.97E-05	0.000129
ADM5	mRNA	2.757842	3.07E-05	0.000133
DUOXA1	mRNA	4.142176	3.09E-05	0.000133
HAPLN1	mRNA	7.745897	3.10E-05	0.000134
SLC16A11	mRNA	-2.01421	3.15E-05	0.000136
NHS	mRNA	2.320709	3.19E-05	0.000137
PDGFRL	mRNA	2.177328	3.21E-05	0.000138
RGPD1	mRNA	-2.28971	3.24E-05	0.00014
SSTR5	mRNA	4.634965	3.25E-05	0.00014
RNF39	mRNA	2.525876	3.25E-05	0.00014
NUTM2G	mRNA	2.865204	3.25E-05	0.00014

FUT3	mRNA	3.680183	3.25E-05	0.00014
CHGB	mRNA	5.649004	3.25E-05	0.00014
FBXL16	mRNA	3.040134	3.26E-05	0.00014
MCAM	mRNA	2.198479	3.26E-05	0.00014
DAW1	mRNA	5.726922	3.27E-05	0.00014
EFNA3	mRNA	2.732565	3.28E-05	0.000141
HOXB6	mRNA	3.139583	3.29E-05	0.000141
TAS2R4	mRNA	2.256	3.32E-05	0.000142
CAPN13	mRNA	3.817426	3.33E-05	0.000143
ACSBG2	mRNA	2.555435	3.34E-05	0.000143
DZIP1L	mRNA	2.017512	3.37E-05	0.000144
ACE	mRNA	3.157595	3.37E-05	0.000144
KISS1R	mRNA	5.475985	3.52E-05	0.00015
SHISA9	mRNA	4.401636	3.53E-05	0.00015
TMEM270	mRNA	3.326168	3.53E-05	0.00015
NME9	mRNA	2.261864	3.54E-05	0.000151
MBOAT1	mRNA	2.203161	3.55E-05	0.000151
MDH1B	mRNA	2.410808	3.56E-05	0.000151
VSIG1	mRNA	6.848059	3.57E-05	0.000152
ESM1	mRNA	3.500396	3.58E-05	0.000152
GABRG1	mRNA	-2.54874	3.58E-05	0.000152
TEX19	mRNA	6.061521	3.59E-05	0.000152
3-Mar	mRNA	2.061686	3.60E-05	0.000153
YJEFN3	mRNA	2.048088	3.61E-05	0.000153
RDM1	mRNA	3.122988	3.61E-05	0.000153
HIGD1B	mRNA	2.01009	3.61E-05	0.000153
ENTPD3	mRNA	4.848548	3.65E-05	0.000155
ADAM32	mRNA	2.883177	3.67E-05	0.000155
ZNF280B	mRNA	2.984385	3.69E-05	0.000156
FBXL13	mRNA	2.555613	3.69E-05	0.000156
LAYN	mRNA	2.829574	3.72E-05	0.000157
ZNF488	mRNA	3.408396	3.73E-05	0.000157
PAPLN	mRNA	2.267821	3.74E-05	0.000158
PDLIM4	mRNA	3.163081	3.76E-05	0.000159
PRAMEF10	mRNA	-4.2172	3.78E-05	0.000159
FAM171B	mRNA	2.024108	3.79E-05	0.00016
RASGRF1	mRNA	3.071597	3.79E-05	0.00016
WIF1	mRNA	7.775381	3.81E-05	0.00016
FOXD4	mRNA	2.683164	3.82E-05	0.000161
WNT3A	mRNA	4.004225	3.90E-05	0.000164
OR2B6	mRNA	4.505575	3.92E-05	0.000165
CRYAB	mRNA	3.902959	3.93E-05	0.000165
VAX2	mRNA	3.488232	3.99E-05	0.000167
TBX4	mRNA	6.920898	4.00E-05	0.000167
HK2	mRNA	2.673872	4.02E-05	0.000168
ANGPTL8	mRNA	-3.25755	4.04E-05	0.000169
GPX8	mRNA	2.110573	4.05E-05	0.000169

C19orf81	mRNA	4.852808	4.08E-05	0.00017
CNGB1	mRNA	5.740765	4.08E-05	0.00017
CNKSR1	mRNA	2.138221	4.08E-05	0.00017
CXCL1	mRNA	2.834412	4.11E-05	0.000171
SLC9A5	mRNA	2.215263	4.11E-05	0.000171
HCN2	mRNA	3.459054	4.13E-05	0.000172
TMEM200A	mRNA	2.37422	4.14E-05	0.000172
GAS7	mRNA	3.426536	4.18E-05	0.000174
HIST1H1D	mRNA	5.826509	4.19E-05	0.000174
SHOX2	mRNA	3.049165	4.22E-05	0.000175
NUDT11	mRNA	3.356396	4.25E-05	0.000176
ZP1	mRNA	4.811471	4.28E-05	0.000178
TMEM130	mRNA	3.872144	4.30E-05	0.000178
NKAIN1	mRNA	5.013422	4.32E-05	0.000179
TRIM72	mRNA	5.163545	4.39E-05	0.000182
MNS1	mRNA	2.09023	4.41E-05	0.000183
PDIA2	mRNA	5.305519	4.42E-05	0.000183
GPR161	mRNA	2.030218	4.45E-05	0.000184
CLLU1OS	mRNA	6.601824	4.47E-05	0.000185
C2CD4A	mRNA	4.140849	4.53E-05	0.000187
FAM24B	mRNA	2.312059	4.54E-05	0.000187
AGBL4	mRNA	-2.26458	4.59E-05	0.000189
B3GNT7	mRNA	2.852951	4.59E-05	0.000189
JPH1	mRNA	3.080674	4.63E-05	0.00019
HSF2BP	mRNA	2.103664	4.65E-05	0.000191
CARNS1	mRNA	3.512837	4.66E-05	0.000192
C6orf141	mRNA	3.296896	4.69E-05	0.000193
HRK	mRNA	4.676	4.70E-05	0.000193
MMP28	mRNA	3.460788	4.71E-05	0.000193
C9orf116	mRNA	2.273883	4.71E-05	0.000193
DIRAS3	mRNA	-2.2515	4.74E-05	0.000194
LRRIQ1	mRNA	4.238807	4.75E-05	0.000195
CHST1	mRNA	2.372665	4.77E-05	0.000196
FLNC	mRNA	2.738391	4.84E-05	0.000198
B3GNT4	mRNA	2.153589	4.88E-05	0.0002
CACNA1E	mRNA	4.060504	4.96E-05	0.000203
VIM	mRNA	2.019675	5.09E-05	0.000208
C6orf222	mRNA	5.674792	5.15E-05	0.00021
ZNF43	mRNA	2.365034	5.18E-05	0.000211
SMPDL3B	mRNA	2.540798	5.22E-05	0.000213
RCOR2	mRNA	2.880573	5.24E-05	0.000213
SIX4	mRNA	3.145204	5.26E-05	0.000214
MGAM2	mRNA	4.366446	5.31E-05	0.000216
AC011479.1	mRNA	3.353655	5.37E-05	0.000218
DZIP1	mRNA	2.59876	5.45E-05	0.000221
APOC2	mRNA	-3.12717	5.49E-05	0.000222
RBMXL2	mRNA	-2.50202	5.50E-05	0.000223

FAM171A1	mRNA	2.031723	5.55E-05	0.000225
MTUS2	mRNA	-2.58602	5.56E-05	0.000225
NPHS1	mRNA	5.829776	5.62E-05	0.000227
MYLK2	mRNA	2.316926	5.62E-05	0.000227
CLCNKB	mRNA	3.053717	5.63E-05	0.000227
KCNN1	mRNA	2.973107	5.65E-05	0.000228
SLC4A10	mRNA	-2.29986	5.71E-05	0.00023
NLRP9	mRNA	3.981943	5.71E-05	0.00023
PAK6	mRNA	3.231806	5.75E-05	0.000231
KIF12	mRNA	2.262984	5.75E-05	0.000231
ARHGAP40	mRNA	5.835314	5.75E-05	0.000231
VASH2	mRNA	2.054286	5.77E-05	0.000232
POSTN	mRNA	2.418837	5.80E-05	0.000233
FHOD3	mRNA	2.356387	5.82E-05	0.000234
PCSK9	mRNA	-2.02438	5.88E-05	0.000236
FCGBP	mRNA	2.923521	5.94E-05	0.000238
DNAAF3	mRNA	2.465216	6.07E-05	0.000243
HIST1H2AD	mRNA	2.789244	6.11E-05	0.000245
PAQR6	mRNA	2.100991	6.13E-05	0.000245
RASGRF2	mRNA	2.051383	6.14E-05	0.000246
AC010616.1	mRNA	4.519817	6.15E-05	0.000246
KRT6B	mRNA	7.62948	6.19E-05	0.000248
MMP9	mRNA	3.107386	6.21E-05	0.000248
FNDC1	mRNA	2.236	6.24E-05	0.000249
LFNG	mRNA	2.848579	6.24E-05	0.000249
COMP	mRNA	3.445105	6.26E-05	0.00025
PCDHGC5	mRNA	3.425294	6.26E-05	0.00025
TRIM6	mRNA	2.165255	6.28E-05	0.000251
GALNT7	mRNA	2.4337	6.35E-05	0.000254
PSG4	mRNA	6.736424	6.36E-05	0.000254
SYT8	mRNA	5.413716	6.39E-05	0.000255
HOXB7	mRNA	4.00113	6.44E-05	0.000257
COL17A1	mRNA	7.58615	6.50E-05	0.000259
KCTD19	mRNA	3.354595	6.50E-05	0.000259
SFRP4	mRNA	2.90195	6.52E-05	0.000259
LAMP5	mRNA	4.849649	6.53E-05	0.00026
HOXA10	mRNA	5.771387	6.64E-05	0.000264
IGFL2	mRNA	5.482364	6.68E-05	0.000265
CRLF2	mRNA	4.595058	6.70E-05	0.000266
ARTN	mRNA	2.604764	6.71E-05	0.000267
CEND1	mRNA	3.300399	6.79E-05	0.000269
MSLN	mRNA	6.830674	6.85E-05	0.000271
DPP4	mRNA	-2.15081	6.85E-05	0.000271
SERPINE2	mRNA	2.41335	6.93E-05	0.000275
ISLR2	mRNA	2.21806	6.99E-05	0.000277
CHST6	mRNA	3.378953	7.09E-05	0.00028
C16orf74	mRNA	3.053758	7.26E-05	0.000286

PRSS21	mRNA	4.994936	7.48E-05	0.000294
TGM3	mRNA	5.597973	7.51E-05	0.000295
SH2D4B	mRNA	3.13956	7.55E-05	0.000297
OLR1	mRNA	2.7018	7.69E-05	0.000301
KIAA1211	mRNA	2.381303	7.70E-05	0.000301
CCDC33	mRNA	5.348277	7.74E-05	0.000303
SSTR1	mRNA	-2.03487	7.75E-05	0.000303
NKX2-8	mRNA	4.098532	7.76E-05	0.000303
CEL	mRNA	3.827433	7.77E-05	0.000304
SSTR3	mRNA	5.085757	7.79E-05	0.000304
MAG	mRNA	-2.686	7.86E-05	0.000307
CADPS	mRNA	3.451793	7.86E-05	0.000307
SLC30A2	mRNA	4.147619	7.90E-05	0.000308
TNFRSF4	mRNA	2.143924	7.92E-05	0.000309
ITGB6	mRNA	4.678738	7.97E-05	0.000311
SPRR3	mRNA	7.014881	8.04E-05	0.000313
ALDH3B1	mRNA	2.061757	8.15E-05	0.000317
GGN	mRNA	2.177177	8.16E-05	0.000317
PNMA8A	mRNA	3.444033	8.23E-05	0.000319
PKDCC	mRNA	2.625634	8.31E-05	0.000322
PLEKHH2	mRNA	3.025711	8.33E-05	0.000323
PPFIA4	mRNA	2.849935	8.34E-05	0.000323
FOXC2	mRNA	2.411576	8.43E-05	0.000327
HSD11B2	mRNA	2.037697	8.49E-05	0.000328
CABP1	mRNA	4.211571	8.49E-05	0.000329
DQX1	mRNA	5.550723	8.58E-05	0.000332
TMEM145	mRNA	3.741846	8.61E-05	0.000333
SAPCD1	mRNA	2.194533	8.69E-05	0.000335
CLUL1	mRNA	2.473074	8.69E-05	0.000335
NME5	mRNA	2.678039	8.79E-05	0.000339
GRAMD2A	mRNA	3.198665	8.80E-05	0.000339
PROCA1	mRNA	2.034088	8.80E-05	0.000339
RAB3B	mRNA	3.538759	8.87E-05	0.000342
SPX	mRNA	-2.70588	8.91E-05	0.000343
ZSCAN23	mRNA	3.085666	9.04E-05	0.000348
PLCH1	mRNA	2.845741	9.23E-05	0.000354
MUC16	mRNA	7.074747	9.23E-05	0.000354
CFAP43	mRNA	2.00896	9.24E-05	0.000355
ANGPTL7	mRNA	-2.78716	9.32E-05	0.000358
APLN	mRNA	2.786582	9.35E-05	0.000359
PCDHB2	mRNA	3.63118	9.36E-05	0.000359
SRPK3	mRNA	2.15793	9.40E-05	0.00036
CYS1	mRNA	3.44215	9.43E-05	0.000361
ZPLD1	mRNA	3.795592	9.44E-05	0.000361
CDKL4	mRNA	2.735333	9.52E-05	0.000364
ADORA1	mRNA	3.733842	9.55E-05	0.000365
FNDC11	mRNA	2.692573	9.57E-05	0.000366

DPT	mRNA	-2.24353	9.58E-05	0.000366
RERG	mRNA	2.367642	9.72E-05	0.000371
PODNL1	mRNA	2.30265	9.73E-05	0.000371
GAP43	mRNA	2.941832	9.87E-05	0.000376
HOXB5	mRNA	2.371308	9.94E-05	0.000378
CDX1	mRNA	3.696114	9.95E-05	0.000379
CPNE9	mRNA	2.508249	1.00E-04	0.00038
LCN2	mRNA	3.628477	0.0001	0.000381
ARSI	mRNA	3.874847	0.000101	0.000385
ATP1A3	mRNA	3.76893	0.000103	0.00039
KIF5A	mRNA	3.662644	0.000103	0.00039
HOXB8	mRNA	5.770233	0.000103	0.00039
ZNF827	mRNA	2.082915	0.000103	0.000391
ADAMTS14	mRNA	3.130483	0.000103	0.000391
CXCL5	mRNA	4.936367	0.000104	0.000393
TRIM36	mRNA	2.117901	0.000104	0.000394
FOXC1	mRNA	2.527324	0.000105	0.000399
SEMA7A	mRNA	2.57422	0.000107	0.000404
DISP3	mRNA	3.153524	0.000107	0.000404
SLCO5A1	mRNA	2.789345	0.000109	0.000409
CCSER1	mRNA	3.24699	0.000109	0.00041
LY6G6C	mRNA	4.895625	0.000109	0.000412
ZNF391	mRNA	2.624395	0.00011	0.000413
PEBP4	mRNA	-2.31541	0.000112	0.000421
CLCNKA	mRNA	3.353601	0.000113	0.000425
SLC28A3	mRNA	3.394092	0.000114	0.000428
PBX4	mRNA	2.370449	0.000115	0.000429
ANKDD1B	mRNA	2.202277	0.000115	0.000431
NLRP2	mRNA	4.398332	0.000116	0.000433
ANKFN1	mRNA	6.522276	0.000116	0.000434
GNAT1	mRNA	-2.82652	0.000116	0.000434
GALR3	mRNA	-2.7126	0.000116	0.000434
ACTA1	mRNA	3.447309	0.000116	0.000434
MGAT3	mRNA	3.734276	0.000116	0.000434
ZNF610	mRNA	2.093581	0.000116	0.000435
CTTNBP2	mRNA	2.496369	0.000118	0.000441
PRSS1	mRNA	8.630182	0.000118	0.000442
PTH1R	mRNA	-2.02438	0.000119	0.000444
FCAMR	mRNA	-2.93138	0.00012	0.000446
ZBED2	mRNA	4.470551	0.000121	0.000452
TPSG1	mRNA	3.010202	0.000122	0.000453
TRPM1	mRNA	-2.14817	0.000123	0.000458
CR2	mRNA	7.470883	0.000125	0.000462
FAM81B	mRNA	5.363239	0.000125	0.000463
ONECUT3	mRNA	7.50981	0.000126	0.000468
VWDE	mRNA	3.014485	0.000126	0.000468
FAM72B	mRNA	2.230963	0.000129	0.000476

SGSM1	mRNA	2.100723	0.00013	0.00048
PCDHGA6	mRNA	2.186111	0.00013	0.000481
HOXA13	mRNA	5.920522	0.000131	0.000484
SYNDIG1	mRNA	3.673598	0.000131	0.000485
HPCA	mRNA	2.959882	0.000132	0.000488
EPHB3	mRNA	3.489103	0.000133	0.00049
CLEC5A	mRNA	2.42616	0.000134	0.000493
SLC6A2	mRNA	-3.62956	0.000134	0.000493
FBXO43	mRNA	2.266433	0.000134	0.000494
MYB	mRNA	3.438688	0.000135	0.000495
TNFRSF18	mRNA	2.549175	0.000135	0.000497
ENOX1	mRNA	2.250038	0.000135	0.000497
ZNF300	mRNA	2.636999	0.000135	0.000497
CDSN	mRNA	3.866228	0.000136	0.0005
BIRC3	mRNA	2.006321	0.000137	0.000503
AQP8	mRNA	-2.7515	0.000138	0.000507
CATSPER1	mRNA	2.821793	0.000138	0.000507
PRKCG	mRNA	5.19493	0.000139	0.000509
KCNH4	mRNA	3.023292	0.000139	0.000509
STEAP1B	mRNA	4.534198	0.000139	0.000509
FOXL1	mRNA	3.168905	0.000139	0.000509
ZIC2	mRNA	4.390297	0.00014	0.000512
CPA4	mRNA	4.241984	0.000141	0.000517
IL1A	mRNA	4.61482	0.000142	0.000518
PAQR9	mRNA	-2.31482	0.000142	0.000519
OLFM4	mRNA	7.63461	0.000143	0.000522
DNAJA4	mRNA	2.138882	0.000147	0.000536
KRT79	mRNA	5.659774	0.000148	0.00054
CTSE	mRNA	5.86563	0.000149	0.000541
B3GNT8	mRNA	2.123841	0.000149	0.000543
IL17REL	mRNA	4.158432	0.00015	0.000544
KLK13	mRNA	4.662157	0.000151	0.000546
CARD14	mRNA	2.632332	0.000151	0.000546
CADM2	mRNA	-2.49723	0.000151	0.000548
SRMS	mRNA	2.199528	0.000152	0.000551
HDC	mRNA	-2.22868	0.000154	0.000556
IGSF11	mRNA	4.721944	0.000154	0.000557
RGS9BP	mRNA	3.757507	0.000154	0.000557
CHRNA6	mRNA	3.430988	0.000155	0.000561
SAMD13	mRNA	2.727043	0.000155	0.000562
FRMPD2	mRNA	3.528003	0.000157	0.000567
TRIM40	mRNA	4.243542	0.000158	0.00057
RGS20	mRNA	2.911805	0.000158	0.000571
FAM72C	mRNA	3.53462	0.00016	0.000577
DLX6	mRNA	5.127251	0.000161	0.000581
NRG3	mRNA	2.877565	0.000162	0.000584
SLC34A3	mRNA	3.256418	0.000162	0.000585

CFTR	mRNA	3.556137	0.000164	0.00059
PRAME	mRNA	6.18499	0.000166	0.000596
RNF157	mRNA	2.462835	0.000167	0.000601
PCDHGC4	mRNA	3.402479	0.000168	0.000602
ANKRD18B	mRNA	3.504933	0.000169	0.000605
HTR3A	mRNA	4.779505	0.00017	0.000608
AARD	mRNA	7.359863	0.00017	0.000608
MYEOV	mRNA	3.921928	0.00017	0.000608
IL22RA2	mRNA	6.795519	0.000171	0.000611
HIST1H2AI	mRNA	6.355161	0.000171	0.000613
TMEM119	mRNA	2.481372	0.000173	0.000618
GRIN2B	mRNA	-3.07152	0.000173	0.000619
UGT1A9	mRNA	-3.06233	0.000173	0.00062
KCNH2	mRNA	3.443339	0.000174	0.000622
MYADML2	mRNA	3.877586	0.000175	0.000625
TFF1	mRNA	6.65362	0.000175	0.000625
EMX1	mRNA	3.809248	0.000175	0.000625
GPR37L1	mRNA	2.074607	0.000176	0.000628
JAKMIP2	mRNA	-2.02512	0.000176	0.000629
ZNF486	mRNA	2.550097	0.000177	0.000631
PRKG2	mRNA	4.784631	0.000177	0.000631
FAM189A1	mRNA	3.177083	0.000177	0.000632
BEGAIN	mRNA	2.792822	0.000178	0.000634
TLR5	mRNA	2.187811	0.000179	0.000636
NPIPB13	mRNA	2.965486	0.000182	0.000646
SPDYE2	mRNA	2.741514	0.000183	0.000649
SOX8	mRNA	3.646111	0.000184	0.000652
TFAP2E	mRNA	2.590855	0.000184	0.000654
RNF183	mRNA	3.597373	0.000185	0.000657
STK32A	mRNA	3.041088	0.000187	0.000661
GFPT2	mRNA	2.562897	0.000187	0.000662
STC1	mRNA	2.156811	0.000187	0.000663
CDX2	mRNA	6.667739	0.000188	0.000664
TLL2	mRNA	2.260843	0.000188	0.000665
TNFRSF13C	mRNA	4.333195	0.000188	0.000666
STRIP2	mRNA	2.632582	0.000189	0.000668
CERS1	mRNA	3.665931	0.000189	0.000669
CUBN	mRNA	3.178963	0.00019	0.000672
UPK3B	mRNA	3.680142	0.000192	0.000677
NPFFR1	mRNA	4.209113	0.000192	0.000677
C5orf67	mRNA	4.470645	0.000193	0.000679
GNG4	mRNA	4.866754	0.000193	0.000681
LCA5	mRNA	2.136918	0.000193	0.000681
KCNN4	mRNA	3.711193	0.000194	0.000681
KRT20	mRNA	12.29278	0.000195	0.000687
AICDA	mRNA	5.531416	0.000197	0.000693
ELAVL3	mRNA	4.530089	0.0002	0.000701

LTB	mRNA	2.982555	0.000201	0.000703
NETO1	mRNA	5.609256	0.000202	0.000708
DNAH3	mRNA	3.177743	0.000203	0.000711
FAM3B	mRNA	3.20581	0.000203	0.000711
AQP5	mRNA	6.792337	0.000203	0.000711
CACNG8	mRNA	3.006732	0.000204	0.000713
PKIA	mRNA	3.567842	0.000204	0.000715
CHRNB4	mRNA	3.511428	0.000205	0.000717
ANXA8L1	mRNA	2.892619	0.000206	0.00072
OR51E1	mRNA	2.249273	0.000211	0.000736
STOX1	mRNA	2.230161	0.000212	0.000737
ATP2A1	mRNA	2.142708	0.000216	0.000751
SHISAL2A	mRNA	3.459927	0.000217	0.000754
JCAD	mRNA	2.380615	0.000217	0.000755
LDHB	mRNA	2.089456	0.000218	0.000757
CREB3L1	mRNA	2.518996	0.000218	0.000758
CD44	mRNA	2.40335	0.000218	0.000759
VGLL3	mRNA	2.638553	0.000219	0.00076
TNNI2	mRNA	4.096274	0.000219	0.000761
CABYR	mRNA	2.632982	0.000219	0.000762
NKX2-5	mRNA	7.314059	0.000221	0.000766
ORAOV1	mRNA	2.577499	0.000226	0.000781
SH3TC2	mRNA	2.666248	0.000226	0.000782
CFAP61	mRNA	2.85266	0.000228	0.000788
CAMK2N2	mRNA	2.596554	0.000228	0.000789
RTN4RL1	mRNA	2.084804	0.00023	0.000794
GSTM1	mRNA	-3.16488	0.00023	0.000795
APBA2	mRNA	2.250312	0.000232	0.000799
DEFB132	mRNA	-2.92853	0.000234	0.000807
DPYSL5	mRNA	6.889587	0.000234	0.000807
TTC23L	mRNA	2.002822	0.000234	0.000808
SHISA8	mRNA	7.245842	0.000235	0.000808
STYK1	mRNA	3.607854	0.000235	0.000808
AKNAD1	mRNA	3.563191	0.000235	0.00081
C1QL1	mRNA	3.989444	0.000235	0.000811
LDHC	mRNA	-2.51898	0.000236	0.000814
SMIM23	mRNA	5.590016	0.000237	0.000815
ADAMTS16	mRNA	3.807108	0.000241	0.000829
DRC7	mRNA	2.768435	0.000245	0.00084
C3orf36	mRNA	2.566351	0.000245	0.000841
SLITRK5	mRNA	3.720057	0.000247	0.000846
GJA3	mRNA	3.016478	0.000247	0.000846
QRFP	mRNA	2.23674	0.00025	0.000855
BCL2L15	mRNA	3.622701	0.000253	0.000864
OR2H2	mRNA	3.396349	0.000256	0.000875
AC106782.1	mRNA	2.778343	0.000258	0.000881
SCNN1A	mRNA	2.905677	0.00026	0.000889

WDR63	mRNA	2.615396	0.000261	0.000891
ADORA2B	mRNA	2.079207	0.000262	0.000892
AC004233.2	mRNA	4.663068	0.000262	0.000894
SPIC	mRNA	-2.52314	0.000263	0.000897
CLDN9	mRNA	2.699271	0.000263	0.000897
INPP4B	mRNA	2.243411	0.000267	0.000907
C3orf14	mRNA	2.097514	0.000267	0.000907
FEZF1	mRNA	7.267869	0.000267	0.000908
SLC9A3	mRNA	4.466768	0.000268	0.00091
C17orf99	mRNA	4.609681	0.000268	0.000911
WDR38	mRNA	3.883471	0.000269	0.000913
MYO1A	mRNA	5.01048	0.000273	0.000925
CDKL2	mRNA	3.257942	0.000275	0.000932
PTGES3L	mRNA	2.158116	0.000276	0.000934
CXCL8	mRNA	2.593472	0.000284	0.000959
CELA3A	mRNA	9.581044	0.000286	0.000964
CELF4	mRNA	2.986169	0.000286	0.000964
FAM218A	mRNA	2.716861	0.000286	0.000964
IGDCC4	mRNA	2.901524	0.000289	0.000971
ST6GALNAC5	mRNA	3.975693	0.00029	0.000974
TMSB15B	mRNA	2.477899	0.00029	0.000975
DRD1	mRNA	-2.43709	0.000292	0.000979
EPHB6	mRNA	2.027299	0.000296	0.000992
HTR1F	mRNA	7.327644	0.000296	0.000993
CKMT1A	mRNA	4.382158	0.000298	0.001
CRYBA2	mRNA	7.008892	0.000302	0.001011
TAF4B	mRNA	2.062595	0.000302	0.001011
LINC00483	mRNA	4.859751	0.000303	0.001013
CBLN4	mRNA	-2.26211	0.000303	0.001015
DDN	mRNA	2.178541	0.000306	0.001024
TRPA1	mRNA	4.93377	0.000307	0.001026
FATE1	mRNA	2.612993	0.000309	0.001032
DNAH5	mRNA	2.61009	0.000311	0.001039
RASSF10	mRNA	4.550686	0.000313	0.001043
STK32B	mRNA	2.434673	0.000318	0.001059
CD70	mRNA	3.979054	0.000319	0.001061
RFLNA	mRNA	3.568717	0.000321	0.001067
EGR4	mRNA	3.364628	0.000321	0.001068
TPH1	mRNA	2.084176	0.000322	0.001069
OR13A1	mRNA	6.363313	0.000323	0.001073
IGF2BP1	mRNA	4.585262	0.000323	0.001074
TNS4	mRNA	5.938099	0.000326	0.001083
TTLL6	mRNA	4.028062	0.000334	0.001106
MAP7D2	mRNA	2.896355	0.000334	0.001107
MEOX1	mRNA	2.577193	0.000336	0.00111
SPINK13	mRNA	3.92403	0.000336	0.001112
ANOS1	mRNA	2.12117	0.000337	0.001116

GJB5	mRNA	4.347276	0.000338	0.001117
PRSS2	mRNA	6.942875	0.000338	0.001118
RAB27B	mRNA	2.338788	0.000346	0.001143
HR	mRNA	2.529174	0.000348	0.001146
C2CD4C	mRNA	2.859288	0.000352	0.001159
HIST1H4D	mRNA	3.224657	0.000353	0.001162
TRIM50	mRNA	5.264914	0.000358	0.001177
USH1G	mRNA	4.596739	0.000359	0.00118
KLHL30	mRNA	2.820247	0.000364	0.001195
ACAN	mRNA	2.822214	0.000365	0.001198
EPHX4	mRNA	2.798314	0.000365	0.001199
CCDC192	mRNA	2.114287	0.000367	0.001204
OR2H1	mRNA	7.350146	0.000369	0.00121
ISL1	mRNA	7.289986	0.000373	0.001224
UCN2	mRNA	3.436046	0.000374	0.001224
CACNA2D2	mRNA	5.210411	0.000374	0.001225
LYPD3	mRNA	2.733508	0.000375	0.001226
MAPT	mRNA	2.080117	0.000375	0.001227
HTR1D	mRNA	3.880317	0.000378	0.001236
FAM57B	mRNA	2.470812	0.000381	0.001246
DOCK3	mRNA	2.17948	0.000381	0.001247
OXCT1	mRNA	2.115561	0.000383	0.001252
C1orf167	mRNA	-2.23876	0.000385	0.001256
SMIM6	mRNA	2.091876	0.000385	0.001257
ZBTB16	mRNA	-2.09891	0.000386	0.00126
IL4I1	mRNA	2.109302	0.000387	0.001261
VILL	mRNA	2.73997	0.000389	0.001267
MYO16	mRNA	-2.01212	0.000389	0.001268
ITLN2	mRNA	-2.36421	0.000393	0.001279
KCNB1	mRNA	-2.13295	0.000393	0.001279
BMP5	mRNA	-2.0513	0.000395	0.001286
CHRNA3	mRNA	3.942879	0.000399	0.001298
PIANP	mRNA	2.314307	0.000399	0.001298
HOXC8	mRNA	5.055827	0.0004	0.001301
BCAT1	mRNA	2.209696	0.000402	0.001305
ZSWIM5	mRNA	2.164816	0.000402	0.001307
MRAP	mRNA	-2.48367	0.000403	0.001308
FSTL4	mRNA	4.665534	0.000405	0.001314
OPRPN	mRNA	-4.0674	0.000409	0.001327
FOXI3	mRNA	5.381807	0.00041	0.001329
GP2	mRNA	5.800039	0.000412	0.001335
PLEKHD1	mRNA	3.276866	0.000415	0.001344
CELSR3	mRNA	2.309679	0.000415	0.001345
BARX1	mRNA	-2.10999	0.000416	0.001345
CALHM1	mRNA	4.633216	0.000416	0.001347
PKHD1L1	mRNA	-2.14376	0.000419	0.001355
SOHLH1	mRNA	-2.59028	0.000419	0.001355

SNCB	mRNA	7.869937	0.000421	0.00136
TRIM67	mRNA	3.600566	0.000429	0.001383
REG1B	mRNA	10.80887	0.000431	0.001387
TACR2	mRNA	2.506899	0.000431	0.001389
IQUB	mRNA	2.405978	0.000435	0.001399
DUOX2	mRNA	4.742951	0.000438	0.001407
CNTNAP5	mRNA	7.806785	0.000443	0.00142
S100P	mRNA	5.948573	0.000443	0.001421
TACSTD2	mRNA	3.189705	0.000445	0.001427
LKAAEAR1	mRNA	4.599385	0.000446	0.00143
MYT1	mRNA	2.436835	0.000447	0.001431
CACNB4	mRNA	2.256341	0.000451	0.001444
RTN1	mRNA	3.203196	0.000451	0.001445
ZNF439	mRNA	2.085367	0.000454	0.001451
CTXND1	mRNA	-2.26509	0.000457	0.001462
C2CD6	mRNA	2.481902	0.000465	0.001484
S100A5	mRNA	3.265471	0.000465	0.001485
CALB1	mRNA	5.346347	0.000468	0.001494
DPP10	mRNA	2.3444	0.000469	0.001495
SIK1	mRNA	-2.30486	0.00047	0.0015
ACTL8	mRNA	7.036641	0.000471	0.001502
S100A4	mRNA	2.077911	0.000473	0.001509
HIST1H2BE	mRNA	2.708351	0.000474	0.001511
CTRC	mRNA	5.895348	0.000477	0.00152
INHBE	mRNA	-2.27415	0.000477	0.00152
CA4	mRNA	4.734761	0.000484	0.00154
FLRT2	mRNA	2.633658	0.000485	0.001542
SCN5A	mRNA	3.212667	0.000485	0.001543
MCHR1	mRNA	-2.30285	0.000486	0.001545
TSPAN5	mRNA	2.163226	0.000488	0.001549
DMP1	mRNA	5.876112	0.000488	0.001549
LRIG3	mRNA	2.158046	0.000488	0.00155
PRR15	mRNA	3.717249	0.000494	0.001568
EN1	mRNA	5.898473	0.000504	0.001595
CD22	mRNA	3.206679	0.000505	0.001597
C19orf67	mRNA	2.650098	0.000507	0.001605
CCNI2	mRNA	2.874587	0.000508	0.001606
TENM4	mRNA	2.620938	0.000516	0.00163
KLK10	mRNA	4.564491	0.00052	0.001641
EBF2	mRNA	2.529996	0.00052	0.001642
NAT8L	mRNA	3.424482	0.000527	0.00166
RIPK3	mRNA	2.042106	0.000527	0.001662
MS4A8	mRNA	4.817518	0.000528	0.001662
GRID1	mRNA	2.39479	0.00053	0.001667
BANK1	mRNA	3.900273	0.000536	0.001685
IL2RA	mRNA	2.748811	0.000539	0.001695
SLC30A3	mRNA	4.050479	0.00054	0.001697

CTRB2	mRNA	7.593984	0.000541	0.0017
LTF	mRNA	3.870559	0.000542	0.001703
SYT6	mRNA	2.48092	0.000546	0.001714
PLSCR2	mRNA	2.379025	0.000551	0.001729
RAD51AP2	mRNA	-2.30363	0.000553	0.001732
JSRP1	mRNA	3.249591	0.000582	0.001816
DCX	mRNA	3.298823	0.000583	0.00182
MYH15	mRNA	2.665228	0.000585	0.001826
C11orf91	mRNA	2.43186	0.000589	0.001835
ABCB5	mRNA	3.469989	0.000589	0.001836
RGS4	mRNA	3.601355	0.00059	0.001838
COL4A3	mRNA	2.205504	0.000601	0.00187
DOC2A	mRNA	2.5855	0.000602	0.001873
PRDM16	mRNA	2.34062	0.000605	0.00188
AC119396.1	mRNA	2.068473	0.000606	0.001884
LHB	mRNA	3.145841	0.000606	0.001884
PLAT	mRNA	2.339964	0.000609	0.00189
FAM196A	mRNA	2.296673	0.00061	0.001893
SPRR1A	mRNA	6.771613	0.000615	0.001905
SERPINB5	mRNA	5.972009	0.000618	0.001914
PAPPA2	mRNA	-2.10115	0.000623	0.001929
B3GALT2	mRNA	3.457199	0.000624	0.001932
C2orf91	mRNA	5.939392	0.000627	0.001938
GPR87	mRNA	6.499087	0.000637	0.001966
COLGALT2	mRNA	4.795546	0.000638	0.001969
RBFOX1	mRNA	7.15793	0.000638	0.00197
PSG9	mRNA	4.153557	0.000647	0.001993
PAK5	mRNA	3.213569	0.000648	0.001997
MUC4	mRNA	3.103429	0.00065	0.002002
SOX21	mRNA	5.133133	0.00065	0.002002
IQCA1	mRNA	2.708308	0.000655	0.002014
AC008687.4	mRNA	3.945581	0.000664	0.002041
HIST1H2BJ	mRNA	2.625436	0.000668	0.00205
PLPPR3	mRNA	3.263371	0.000669	0.002053
CLDN6	mRNA	4.998822	0.000672	0.002061
APCDD1L	mRNA	6.735731	0.000673	0.002063
PCDHB11	mRNA	2.677076	0.000678	0.002078
PAX2	mRNA	5.325983	0.000686	0.0021
ALPK3	mRNA	2.244087	0.000688	0.002106
PDE6B	mRNA	2.431773	0.00069	0.00211
AL031708.1	mRNA	2.078658	0.00069	0.002112
SEZ6	mRNA	5.333299	0.000691	0.002112
SYT3	mRNA	3.297814	0.000693	0.002121
OVOL1	mRNA	3.514318	0.000696	0.002128
KRTAP5-5	mRNA	5.783611	0.000703	0.002147
CD1A	mRNA	4.047217	0.000704	0.00215
FAM3D	mRNA	3.985568	0.000707	0.002157

TTBK1	mRNA	-2.00958	0.000714	0.002177
NRG2	mRNA	3.42281	0.000715	0.002179
ADGRF2	mRNA	4.974955	0.00072	0.002192
CIDEC	mRNA	-2.1307	0.000725	0.002205
PVRIG	mRNA	2.005256	0.000729	0.002215
VPREB3	mRNA	4.299799	0.00073	0.002218
TRPC6	mRNA	2.322896	0.000733	0.002228
SHISA6	mRNA	3.869919	0.000742	0.002251
NDUFA4L2	mRNA	2.197241	0.000743	0.002254
CAMKV	mRNA	6.971256	0.000748	0.002265
MALL	mRNA	3.142318	0.000751	0.002274
PCDHB6	mRNA	2.471919	0.000752	0.002277
POU3F3	mRNA	8.811274	0.000755	0.002283
KCNJ16	mRNA	2.749596	0.000765	0.002312
UPK2	mRNA	2.596307	0.000767	0.002317
AC112229.3	mRNA	3.697735	0.000767	0.002317
GJA1	mRNA	2.225876	0.000773	0.002334
AVPR1B	mRNA	5.107301	0.000774	0.002337
PTPRR	mRNA	3.798993	0.000783	0.002361
TNC	mRNA	2.591511	0.000783	0.002361
GGT6	mRNA	3.518014	0.000786	0.002369
DMRT2	mRNA	4.351221	0.000799	0.002404
DNAJB13	mRNA	2.476306	0.000802	0.002412
HIST3H3	mRNA	4.327142	0.000805	0.00242
HS6ST2	mRNA	4.409817	0.000808	0.002428
HOXD9	mRNA	3.072444	0.000813	0.002439
ASXL3	mRNA	-2.06649	0.000817	0.00245
LGALS9B	mRNA	3.189291	0.000818	0.00245
DMC1	mRNA	2.006643	0.000823	0.002464
ANXA10	mRNA	-2.68448	0.000827	0.002475
PRR5-ARHGAP8	mRNA	2.62955	0.00083	0.002483
DLGAP1	mRNA	2.408137	0.000845	0.002527
IYD	mRNA	-2.18486	0.000861	0.002565
CBLN2	mRNA	4.469473	0.000862	0.002568
ODAM	mRNA	6.177559	0.000868	0.002583
SLC6A4	mRNA	2.781446	0.000871	0.00259
IGFL1	mRNA	9.966364	0.000875	0.002602
KCNJ6	mRNA	8.163128	0.00088	0.002614
DUOXA2	mRNA	4.86092	0.00089	0.002639
HOXC13	mRNA	6.283901	0.000893	0.002649
AFF2	mRNA	3.629212	0.000894	0.002652
SGCZ	mRNA	-2.23352	0.000912	0.002698
CACNA2D3	mRNA	2.734595	0.000921	0.002718
SCG3	mRNA	5.500512	0.00093	0.002744
IGSF5	mRNA	2.821008	0.000936	0.00276
TENM2	mRNA	-2.27928	0.000944	0.002779
ADAMTS18	mRNA	3.359237	0.00096	0.002822

CACNG6	mRNA	6.308353	0.000969	0.002845
ERC2	mRNA	2.925518	0.000975	0.002862
MBOAT4	mRNA	2.99558	0.000981	0.002878
DMRT1	mRNA	6.245751	0.000984	0.002886
AC013470.2	mRNA	5.35627	0.000985	0.002889
EN2	mRNA	3.959324	0.00099	0.002904
TRIM31	mRNA	2.951286	0.000991	0.002906
REC8	mRNA	2.344299	0.000999	0.002927
AK5	mRNA	2.542293	0.001002	0.002934
NBL1	mRNA	2.023916	0.001003	0.002936
KRT16	mRNA	5.820859	0.001005	0.002942
TRPC4	mRNA	2.209988	0.001007	0.002947
SEMA3A	mRNA	2.817061	0.001008	0.002948
CDH20	mRNA	5.822648	0.001009	0.00295
FAM83E	mRNA	3.830435	0.001009	0.00295
ADRA2A	mRNA	2.800803	0.001011	0.002954
IRX3	mRNA	2.794191	0.001014	0.002962
GNG13	mRNA	4.480365	0.001015	0.002963
KRT83	mRNA	4.233138	0.001017	0.002968
OR51E2	mRNA	2.697358	0.001019	0.002973
HOXA3	mRNA	2.619996	0.00103	0.003003
FGF9	mRNA	5.571025	0.00103	0.003003
ALKAL2	mRNA	2.36397	0.001037	0.003022
ANK1	mRNA	2.549408	0.001041	0.003029
MGAT5B	mRNA	3.441991	0.001042	0.003033
PSG8	mRNA	5.945046	0.001067	0.003096
REG3A	mRNA	8.031412	0.001068	0.003097
HOXA6	mRNA	4.475026	0.001069	0.003101
KLK14	mRNA	2.234955	0.001075	0.003115
A4GNT	mRNA	2.374995	0.001078	0.003122
NCCRP1	mRNA	3.354649	0.001078	0.003122
LENEP	mRNA	4.601511	0.001079	0.003124
ZNF66	mRNA	2.193493	0.001085	0.003139
NCR3LG1	mRNA	2.335977	0.001086	0.003141
CST4	mRNA	6.059523	0.001093	0.003159
KLK6	mRNA	6.661342	0.001109	0.003199
MUC5AC	mRNA	8.600673	0.00111	0.003199
MORC1	mRNA	-2.22784	0.00111	0.003201
CITED1	mRNA	3.107399	0.001111	0.003203
SULT1C4	mRNA	3.683375	0.001112	0.003205
GPR1	mRNA	3.34938	0.001125	0.003241
GABBR2	mRNA	3.14692	0.001127	0.003244
SYTL1	mRNA	2.112165	0.001129	0.003249
GLP1R	mRNA	5.208746	0.001148	0.003297
XIRP1	mRNA	3.317884	0.001153	0.003309
SLN	mRNA	5.143194	0.001153	0.003309
FOXQ1	mRNA	2.751893	0.001164	0.003338

ASGR2	mRNA	-2.04652	0.001172	0.003361
SFTPA1	mRNA	-2.71649	0.001175	0.003367
CPLX2	mRNA	9.865484	0.001176	0.003369
SYT5	mRNA	3.867939	0.001176	0.00337
BCAS1	mRNA	2.772269	0.001184	0.00339
CTRB1	mRNA	8.091592	0.001184	0.00339
BIRC7	mRNA	3.507836	0.0012	0.003429
SYP	mRNA	2.342841	0.001203	0.003439
MYO18B	mRNA	3.103853	0.001204	0.00344
SCGB2A1	mRNA	3.753641	0.001205	0.003443
OTOP3	mRNA	6.991756	0.001215	0.003466
FAM72D	mRNA	2.1549	0.001219	0.003475
RAET1L	mRNA	3.824923	0.001219	0.003475
SRRM4	mRNA	7.801614	0.00122	0.003476
NDP	mRNA	5.468843	0.001234	0.003513
C11orf70	mRNA	2.316677	0.001259	0.003576
SLC2A5	mRNA	2.296442	0.001262	0.003584
HES7	mRNA	3.188618	0.001268	0.003598
CFAP73	mRNA	2.259114	0.001274	0.003614
PRIMA1	mRNA	-2.05498	0.001276	0.003616
ZNF750	mRNA	3.685612	0.001282	0.003633
ZBTB32	mRNA	2.530082	0.001295	0.003666
TMEM158	mRNA	2.505286	0.001304	0.003687
SFTA2	mRNA	3.133248	0.001315	0.003713
LPAR3	mRNA	4.693375	0.001326	0.00374
DLX3	mRNA	3.697079	0.001328	0.003747
SPACA4	mRNA	4.321201	0.00135	0.003805
PHYHIPL	mRNA	2.651726	0.001351	0.003807
C1orf94	mRNA	6.865789	0.001366	0.003845
MUC19	mRNA	3.310275	0.001376	0.003871
CDH16	mRNA	4.84329	0.001377	0.003874
FXYD4	mRNA	5.334971	0.001386	0.003897
KCND2	mRNA	2.282128	0.001403	0.003939
ALX4	mRNA	6.088736	0.001407	0.003949
EFHC2	mRNA	3.112391	0.001408	0.003951
LRRN4	mRNA	2.581281	0.001414	0.003963
HIST1H2BH	mRNA	4.328405	0.001426	0.003992
TMPRSS11E	mRNA	5.310451	0.001433	0.004013
P2RX5	mRNA	3.340509	0.001436	0.00402
BATF	mRNA	2.299509	0.001445	0.004041
POPDC3	mRNA	7.464884	0.001466	0.004097
KIRREL2	mRNA	4.049699	0.001469	0.004104
TTLL10	mRNA	2.580728	0.001471	0.004107
PSAPL1	mRNA	5.664998	0.001474	0.004114
SPAG6	mRNA	4.869415	0.001477	0.004121
KCNH8	mRNA	2.950461	0.001477	0.004121
CYP26B1	mRNA	2.774139	0.001481	0.00413

ERBB4	mRNA	2.809203	0.001486	0.00414
MYBPC2	mRNA	2.946333	0.001489	0.004148
PTH2R	mRNA	3.729563	0.001508	0.004194
CPEB1	mRNA	3.483882	0.001515	0.004211
PNPLA1	mRNA	3.969241	0.001519	0.004221
PCP4	mRNA	4.80385	0.001526	0.00424
TMPRSS3	mRNA	2.756622	0.001535	0.004264
CALCB	mRNA	4.507939	0.001537	0.004268
CA8	mRNA	4.349067	0.001545	0.004286
FAM153A	mRNA	3.105039	0.001562	0.00433
IGFL4	mRNA	2.991006	0.001569	0.004348
TFPI2	mRNA	3.776102	0.001576	0.004362
COCH	mRNA	2.623938	0.001593	0.004403
ALOXE3	mRNA	2.64999	0.001594	0.004404
TRIM7	mRNA	2.490801	0.001619	0.004463
FAM71F1	mRNA	3.071875	0.001629	0.00449
GALNT14	mRNA	2.830795	0.001632	0.004497
C7orf61	mRNA	2.025216	0.001654	0.004552
HIST1H2BO	mRNA	3.361602	0.001662	0.004571
LRRC26	mRNA	2.915276	0.001664	0.004574
LAMA1	mRNA	4.301283	0.001669	0.004588
C2orf73	mRNA	3.08882	0.001675	0.004601
CCND3	mRNA	2.121951	0.001676	0.004605
SCUBE3	mRNA	2.582629	0.001682	0.004619
IAPP	mRNA	-2.66533	0.001687	0.00463
TGM5	mRNA	4.791967	0.001688	0.00463
CHRNG	mRNA	2.534589	0.001721	0.004713
OR11A1	mRNA	6.767965	0.001722	0.004713
SLC10A2	mRNA	6.877598	0.001732	0.004739
SIGLEC15	mRNA	3.289798	0.00174	0.004759
KRT40	mRNA	3.67755	0.001761	0.00481
NRIP3	mRNA	2.049786	0.001774	0.00484
SLCO4A1	mRNA	2.483763	0.001776	0.004846
TIMD4	mRNA	-2.01304	0.001778	0.004849
OTP	mRNA	4.291711	0.001779	0.00485
DNAH9	mRNA	2.286914	0.001779	0.00485
SLITRK4	mRNA	2.342372	0.001805	0.00491
FER1L6	mRNA	4.637826	0.001807	0.004915
CSF2	mRNA	3.600846	0.001816	0.004938
OR12D2	mRNA	6.910356	0.001818	0.004942
RYR2	mRNA	2.975223	0.00182	0.004945
IL17B	mRNA	3.007689	0.001824	0.004955
ANKRD33B	mRNA	2.294833	0.001833	0.004978
CROCC2	mRNA	-2.32888	0.001834	0.004979
PITX2	mRNA	3.641557	0.001842	0.004998
KCNJ13	mRNA	5.758654	0.001857	0.005035
CALCA	mRNA	4.610827	0.001858	0.005035

FOXE1	mRNA	4.612789	0.001858	0.005037
IL31RA	mRNA	3.684325	0.001863	0.005044
CPA6	mRNA	3.715776	0.001865	0.005048
LHFPL4	mRNA	5.263205	0.001867	0.005054
DACH2	mRNA	3.751782	0.001881	0.005086
NOX5	mRNA	3.762965	0.001885	0.005094
AQP2	mRNA	6.054971	0.001885	0.005096
MSH4	mRNA	2.4081	0.001891	0.005108
GALNT5	mRNA	3.643101	0.001897	0.005123
1-Dec	mRNA	2.661927	0.001903	0.005136
C21orf33	mRNA	-2.00051	0.001909	0.005148
LGALS7B	mRNA	4.72858	0.001909	0.005148
TUBB2B	mRNA	2.07429	0.001915	0.005163
ZNF560	mRNA	6.237794	0.001938	0.005221
AGR2	mRNA	5.093952	0.001943	0.005233
CHST8	mRNA	3.538683	0.001955	0.005261
CD19	mRNA	5.160745	0.001956	0.005262
CPA2	mRNA	5.167431	0.00196	0.005271
LRRC66	mRNA	2.246432	0.001966	0.005287
SLC6A3	mRNA	3.432578	0.001966	0.005287
SMC1B	mRNA	2.101981	0.001971	0.005297
PGLYRP4	mRNA	3.281515	0.001989	0.005346
HOXD10	mRNA	5.335424	0.00199	0.005348
KRT14	mRNA	4.429158	0.001994	0.005356
SLC22A31	mRNA	3.935603	0.001995	0.005359
TCN1	mRNA	3.908384	0.002001	0.005372
MSLNL	mRNA	8.055265	0.002034	0.005451
VWC2L	mRNA	3.955438	0.002043	0.005472
TFF3	mRNA	3.799413	0.002043	0.005473
PCDHA1	mRNA	3.926548	0.002068	0.005524
SLC7A5	mRNA	2.017068	0.002071	0.005532
DHDH	mRNA	2.032561	0.002079	0.00555
TINCR	mRNA	2.494829	0.002091	0.005577
PRR25	mRNA	2.952826	0.002091	0.005577
FAM135B	mRNA	3.39268	0.002104	0.005608
IRX6	mRNA	5.132698	0.002111	0.005625
KLK12	mRNA	5.437217	0.002128	0.005664
PYDC1	mRNA	5.026707	0.002128	0.005664
OCA2	mRNA	3.237747	0.00213	0.005667
RAET1E	mRNA	2.796975	0.002134	0.005675
PRAC2	mRNA	5.859489	0.002139	0.005687
LIPI	mRNA	3.984371	0.002143	0.005696
PPP1R36	mRNA	2.319669	0.002152	0.005719
GPA33	mRNA	3.298842	0.002154	0.005722
C8orf74	mRNA	4.332878	0.002159	0.005733
TRPV6	mRNA	2.412088	0.002183	0.005792
SCG2	mRNA	2.700952	0.002201	0.005834

DLX1	mRNA	2.88345	0.002201	0.005834
GDF5	mRNA	4.934407	0.002202	0.005834
CXCL3	mRNA	2.240356	0.002227	0.005892
KRTAP2-3	mRNA	6.284327	0.002267	0.005984
MSX2	mRNA	2.88772	0.002272	0.005996
BPIFB2	mRNA	-2.41568	0.002275	0.006002
KLK5	mRNA	8.014858	0.002276	0.006002
CRB2	mRNA	3.4197	0.00229	0.006036
FAM19A3	mRNA	3.112865	0.002296	0.006048
SLC38A5	mRNA	2.26566	0.002296	0.006048
SNCG	mRNA	2.501625	0.002307	0.006071
GREM1	mRNA	2.267788	0.002318	0.006095
AIRE	mRNA	3.505262	0.002321	0.006104
HLA-DOB	mRNA	2.35976	0.002346	0.006163
UNC80	mRNA	3.591609	0.002366	0.006208
MESP2	mRNA	2.217917	0.002369	0.006214
PCDHGA7	mRNA	2.0276	0.002384	0.006248
C7orf57	mRNA	3.04179	0.002395	0.006276
ATP10B	mRNA	3.358499	0.002395	0.006276
VAT1L	mRNA	2.819377	0.002412	0.006316
GDPD2	mRNA	2.704831	0.002437	0.006371
VAX1	mRNA	5.791175	0.002445	0.006391
CLIC3	mRNA	2.555881	0.002461	0.006427
KRTDAP	mRNA	2.288322	0.002469	0.006444
VGLL1	mRNA	4.138543	0.002478	0.006465
SLC39A12	mRNA	-2.51237	0.002495	0.006503
PDE10A	mRNA	2.024556	0.002502	0.006519
PCDHB7	mRNA	2.080767	0.002504	0.006523
SLC30A8	mRNA	7.958254	0.00251	0.006536
PLCZ1	mRNA	-2.4026	0.002532	0.006585
FCRL4	mRNA	5.729808	0.002537	0.006596
TSPAN19	mRNA	4.40949	0.002548	0.006622
TEX15	mRNA	3.354713	0.002598	0.00674
KIAA1257	mRNA	2.065673	0.002599	0.006741
ECEL1	mRNA	5.586713	0.002616	0.006783
VSX1	mRNA	3.509949	0.002616	0.006783
RP1L1	mRNA	2.878354	0.002624	0.006802
TRIM29	mRNA	3.548302	0.002647	0.006855
PODXL2	mRNA	2.605378	0.002658	0.006879
GRIN2D	mRNA	2.027684	0.002689	0.006953
HOXD4	mRNA	3.685283	0.002696	0.006971
MAB21L2	mRNA	3.077981	0.002698	0.006975
COL11A1	mRNA	3.145609	0.002719	0.007023
ZFP57	mRNA	3.753233	0.002719	0.007023
NRCAM	mRNA	2.057746	0.002748	0.007085
QPCT	mRNA	2.630306	0.002753	0.007099
SLC15A5	mRNA	-2.33329	0.002783	0.007166

TOX	mRNA	2.558026	0.002798	0.007202
LIX1	mRNA	6.023903	0.002803	0.007213
TRPV5	mRNA	2.570498	0.002834	0.007281
DLL3	mRNA	3.584267	0.002859	0.007332
ASIC4	mRNA	2.553961	0.002873	0.007359
C6orf15	mRNA	6.910707	0.002893	0.007406
HS6ST3	mRNA	4.149569	0.002941	0.007517
ZCCHC18	mRNA	2.104828	0.002959	0.007557
TMEM40	mRNA	4.337565	0.002982	0.007613
RHEX	mRNA	3.391649	0.003034	0.007723
HOXC11	mRNA	6.666256	0.00304	0.007736
BTN1A1	mRNA	5.388087	0.003061	0.00778
SCEL	mRNA	4.306926	0.003096	0.007856
OR10Q1	mRNA	3.741442	0.003123	0.007918
HIST1H1B	mRNA	4.570636	0.003131	0.007936
TMEM171	mRNA	2.209525	0.003141	0.007961
LPO	mRNA	5.352915	0.003148	0.007973
STXBP5L	mRNA	7.293033	0.00319	0.008064
TMSB15B	mRNA	2.591697	0.003202	0.008091
UGT1A8	mRNA	5.514106	0.003205	0.008096
RTBDN	mRNA	4.444194	0.003206	0.008097
UBE2U	mRNA	4.538759	0.003216	0.00812
IL37	mRNA	4.917903	0.003238	0.008167
SAP25	mRNA	2.205216	0.003249	0.008193
AL355102.2	mRNA	4.016619	0.003254	0.008204
HOXD1	mRNA	4.581722	0.003281	0.008268
GABRP	mRNA	3.532471	0.003288	0.008282
CXCL6	mRNA	2.277736	0.003308	0.008325
VSIG8	mRNA	2.101247	0.003308	0.008325
CPA5	mRNA	2.641588	0.003311	0.008329
HS3ST5	mRNA	4.707185	0.003317	0.008342
AJAP1	mRNA	2.79377	0.003318	0.008343
GRIP2	mRNA	2.281122	0.003329	0.008365
ANKRD7	mRNA	2.768658	0.00334	0.008389
EPHA7	mRNA	4.772008	0.003348	0.008408
HOXC10	mRNA	5.765208	0.00339	0.0085
LRRD1	mRNA	2.835372	0.003404	0.008533
CEP295NL	mRNA	2.039117	0.003414	0.008554
RNF150	mRNA	2.015219	0.003452	0.008634
NPPC	mRNA	4.456265	0.003478	0.008693
GIP	mRNA	5.3717	0.003491	0.008722
KRTAP4-1	mRNA	5.690271	0.003496	0.00873
KRT6C	mRNA	7.745709	0.003519	0.008781
CYP2S1	mRNA	2.727491	0.003521	0.008785
TMEM179	mRNA	2.77869	0.003537	0.008822
C8orf88	mRNA	2.299864	0.003551	0.008851
SH3GL3	mRNA	3.9823	0.003554	0.008858

CFAP77	mRNA	2.739862	0.003563	0.008879
PCDH7	mRNA	2.337832	0.003575	0.008906
CHI3L2	mRNA	2.604364	0.003594	0.008949
SLC5A1	mRNA	2.52901	0.003608	0.008979
PEX5L	mRNA	2.790733	0.003633	0.00904
PHF21B	mRNA	4.361248	0.003656	0.009089
MUC2	mRNA	3.722016	0.003662	0.009102
VWA2	mRNA	3.256908	0.003663	0.009103
KIF5C	mRNA	2.512836	0.003668	0.009115
TEX11	mRNA	2.575877	0.003709	0.009205
RFX8	mRNA	2.298957	0.003743	0.009281
PLP1	mRNA	-2.10952	0.003767	0.00933
APOBEC2	mRNA	2.443955	0.003773	0.009342
CPNE5	mRNA	2.061715	0.003793	0.009387
IL9R	mRNA	2.049223	0.003813	0.009427
CDH15	mRNA	-2.14185	0.003829	0.009464
GPR156	mRNA	2.463684	0.003856	0.009529
PIP	mRNA	5.940011	0.003891	0.009603
ARSH	mRNA	5.059999	0.003913	0.009651
KCNH5	mRNA	5.238162	0.003926	0.009682
TSPYL5	mRNA	2.04316	0.003932	0.009691
TAC3	mRNA	3.985794	0.003935	0.009695
ZNF280A	mRNA	5.331698	0.003953	0.00973
BDNF	mRNA	2.378925	0.003962	0.009749
LANCL3	mRNA	2.295131	0.003987	0.009801
OTOGL	mRNA	2.507477	0.003995	0.009821
RNASE7	mRNA	2.633461	0.004026	0.009879
TM4SF19-TCTEX1D2	mRNA	3.678553	0.004052	0.009934

Table S3. One hundred and seventeen DElncRNAs interact with fourteen DEmiRNAs retrieved from the miRcode database.

lncRNA	miRNA
AL591845.1	hsa-mir-96
AL591845.1	hsa-mir-182
AL591845.1	hsa-mir-122
AL591845.1	hsa-mir-383
KIAA0087	hsa-mir-96
KIAA0087	hsa-mir-141
KIAA0087	hsa-mir-200a
KIAA0087	hsa-mir-182
KIAA0087	hsa-mir-183
KIAA0087	hsa-mir-429
KIAA0087	hsa-mir-211
KIAA0087	hsa-mir-383
H19	hsa-mir-141
H19	hsa-mir-200a

C2orf48	hsa-mir-183
C2orf48	hsa-mir-211
C2orf48	hsa-mir-122
AC005280.1	hsa-mir-96
AC005280.1	hsa-mir-182
AC127496.1	hsa-mir-96
AC127496.1	hsa-mir-182
AC127496.1	hsa-mir-183
AC127496.1	hsa-mir-211
AC127496.1	hsa-mir-122
C15orf56	hsa-mir-144
C15orf56	hsa-mir-182
C15orf56	hsa-mir-183
C15orf56	hsa-mir-122
AC012074.1	hsa-mir-122
C10orf91	hsa-mir-429
C10orf91	hsa-mir-211
C10orf91	hsa-mir-122
LINC00304	hsa-mir-183
LINC00304	hsa-mir-187
LINC00304	hsa-mir-222
FAM87A	hsa-mir-96
FAM87A	hsa-mir-141
FAM87A	hsa-mir-200a
FAM87A	hsa-mir-222
FAM87A	hsa-mir-31
COL18A1-AS1	hsa-mir-187
LINC00315	hsa-mir-183
LINC00313	hsa-mir-187
LINC00313	hsa-mir-211
LINC00313	hsa-mir-122
LINC00313	hsa-mir-31
C9orf163	hsa-mir-21
AC087392.1	hsa-mir-144
AC087392.1	hsa-mir-429
AC087392.1	hsa-mir-222
LINC00336	hsa-mir-96
LINC00336	hsa-mir-21
AC116351.1	hsa-mir-31
PSORS1C3	hsa-mir-211
FAM201A	hsa-mir-96
FAM201A	hsa-mir-144
FAM201A	hsa-mir-182
FAM201A	hsa-mir-21
FAM201A	hsa-mir-222
CLLU1	hsa-mir-141
CLLU1	hsa-mir-200a

CLLU1	hsa-mir-144
CLLU1	hsa-mir-429
CLLU1	hsa-mir-211
CLLU1	hsa-mir-21
CLLU1	hsa-mir-222
CLLU1	hsa-mir-31
TTLL10-AS1	hsa-mir-122
AL359878.1	hsa-mir-141
AL359878.1	hsa-mir-200a
FAM99A	hsa-mir-122
AC108134.1	hsa-mir-182
AC108134.1	hsa-mir-183
AC092117.1	hsa-mir-141
AC092117.1	hsa-mir-200a
AC092117.1	hsa-mir-211
AP000525.1	hsa-mir-31
AL137145.1	hsa-mir-211
AL137145.1	hsa-mir-222
AL137145.1	hsa-mir-31
AL021068.1	hsa-mir-429
AL021068.1	hsa-mir-211
AL021068.1	hsa-mir-122
UCA1	hsa-mir-96
UCA1	hsa-mir-182
UCA1	hsa-mir-122
UCA1	hsa-mir-383
AC010336.2	hsa-mir-141
AC010336.2	hsa-mir-200a
AC010336.2	hsa-mir-144
AC010336.2	hsa-mir-182
AC010336.2	hsa-mir-429
AC010336.2	hsa-mir-211
AC010336.2	hsa-mir-122
AC010336.2	hsa-mir-31
AC011481.1	hsa-mir-211
AL137145.2	hsa-mir-144
AL137145.2	hsa-mir-182
AL137145.2	hsa-mir-429
AL137145.2	hsa-mir-31
AC016773.1	hsa-mir-122
AC016773.1	hsa-mir-31
AL354984.1	hsa-mir-429
AC090150.1	hsa-mir-182
AC080037.1	hsa-mir-429
AC096642.1	hsa-mir-96
CLDN10-AS1	hsa-mir-222
POU6F2-AS1	hsa-mir-144

LINC00466	hsa-mir-96
LINC00466	hsa-mir-141
LINC00466	hsa-mir-200a
LINC00466	hsa-mir-144
LINC00466	hsa-mir-183
LINC00466	hsa-mir-429
LINC00466	hsa-mir-211
LINC00466	hsa-mir-21
WARS2-IT1	hsa-mir-144
THRB-IT1	hsa-mir-429
THRB-IT1	hsa-mir-211
LINC00184	hsa-mir-31
LINC00337	hsa-mir-182
LINC00337	hsa-mir-383
SFTA1P	hsa-mir-182
SFTA1P	hsa-mir-211
SFTA1P	hsa-mir-222
SFTA1P	hsa-mir-122
BOLA3-AS1	hsa-mir-182
BOLA3-AS1	hsa-mir-429
BOLA3-AS1	hsa-mir-211
BOLA3-AS1	hsa-mir-122
BOLA3-AS1	hsa-mir-383
DBH-AS1	hsa-mir-211
AL356356.1	hsa-mir-141
AL356356.1	hsa-mir-200a
AL356356.1	hsa-mir-211
LINC00323	hsa-mir-96
LINC00323	hsa-mir-182
LINC00323	hsa-mir-211
FAM66C	hsa-mir-183
FAM66C	hsa-mir-187
FAM66C	hsa-mir-429
FAM66C	hsa-mir-211
FAM66C	hsa-mir-21
FAM66C	hsa-mir-222
FAM66C	hsa-mir-122
LINC00348	hsa-mir-141
LINC00348	hsa-mir-200a
LINC00348	hsa-mir-144
HOTAIR	hsa-mir-211
HOTAIR	hsa-mir-21
HOTAIR	hsa-mir-222
LINC00242	hsa-mir-96
LINC00242	hsa-mir-141
LINC00242	hsa-mir-200a
LINC00242	hsa-mir-211

LINC00242	hsa-mir-222
LINC00242	hsa-mir-31
LRRC3-AS1	hsa-mir-96
LRRC3-AS1	hsa-mir-182
LRRC3-AS1	hsa-mir-211
SZT2-AS1	hsa-mir-183
UBE2Q1-AS1	hsa-mir-96
UBE2Q1-AS1	hsa-mir-182
UBE2Q1-AS1	hsa-mir-187
UBE2Q1-AS1	hsa-mir-211
LINC00443	hsa-mir-141
LINC00443	hsa-mir-200a
LINC00443	hsa-mir-144
LINC00443	hsa-mir-183
LINC00443	hsa-mir-211
HM13-AS1	hsa-mir-187
HM13-AS1	hsa-mir-31
AL161645.1	hsa-mir-96
AL161645.1	hsa-mir-144
AL161645.1	hsa-mir-211
AL161645.1	hsa-mir-122
DLG3-AS1	hsa-mir-222
DLX6-AS1	hsa-mir-141
DLX6-AS1	hsa-mir-200a
DLX6-AS1	hsa-mir-144
DLX6-AS1	hsa-mir-429
DLX6-AS1	hsa-mir-211
DLX6-AS1	hsa-mir-122
DLX6-AS1	hsa-mir-31
DLX6-AS1	hsa-mir-383
F10-AS1	hsa-mir-182
HS1BP3-IT1	hsa-mir-21
SLC6A1-AS1	hsa-mir-383
FAM215B	hsa-mir-31
AC037487.1	hsa-mir-222
AC110491.1	hsa-mir-141
AC110491.1	hsa-mir-200a
AC110491.1	hsa-mir-182
AC110491.1	hsa-mir-429
AC110491.1	hsa-mir-211
AC110491.1	hsa-mir-222
LINC00460	hsa-mir-429
LINC00460	hsa-mir-222
TPRG1-AS1	hsa-mir-182
AC092171.1	hsa-mir-96
AC092171.1	hsa-mir-182
AC092171.1	hsa-mir-187

AC092171.1	hsa-mir-383
MAGI2-AS3	hsa-mir-141
MAGI2-AS3	hsa-mir-200a
MAGI2-AS3	hsa-mir-144
MAGI2-AS3	hsa-mir-429
MAGI2-AS3	hsa-mir-211
MAGI2-AS3	hsa-mir-122
MAGI2-AS3	hsa-mir-31
GAS5	hsa-mir-96
GAS5	hsa-mir-144
GAS5	hsa-mir-182
GAS5	hsa-mir-429
GAS5	hsa-mir-21
GAS5	hsa-mir-222
GAS5	hsa-mir-31
MLIP-AS1	hsa-mir-144
MLIP-AS1	hsa-mir-222
MLIP-AS1	hsa-mir-383
LINC00402	hsa-mir-141
LINC00402	hsa-mir-200a
LINC00402	hsa-mir-182
LINC00402	hsa-mir-429
LINC00402	hsa-mir-211
LINC00402	hsa-mir-383
LINC00494	hsa-mir-182
LINC00494	hsa-mir-222
LINC00494	hsa-mir-31
LINC00494	hsa-mir-383
SAPCD1-AS1	hsa-mir-187
SAPCD1-AS1	hsa-mir-211
NEXN-AS1	hsa-mir-211
AC015987.1	hsa-mir-429
AC015987.1	hsa-mir-211
AC015987.1	hsa-mir-383
LINC00316	hsa-mir-187
LINC00316	hsa-mir-122
AL137798.1	hsa-mir-211
AL137798.1	hsa-mir-122
AC009121.1	hsa-mir-141
AC009121.1	hsa-mir-200a
CLRN1-AS1	hsa-mir-429
CLRN1-AS1	hsa-mir-211
CLRN1-AS1	hsa-mir-222
AL391832.1	hsa-mir-383
AP004609.1	hsa-mir-383
ITIH4-AS1	hsa-mir-211
ITIH4-AS1	hsa-mir-122

AC080129.1	hsa-mir-122
AC080129.1	hsa-mir-383
AL118511.1	hsa-mir-383
TM4SF1-AS1	hsa-mir-141
TM4SF1-AS1	hsa-mir-200a
ARHGAP31-AS1	hsa-mir-122
AL359541.1	hsa-mir-122
SYNPR-AS1	hsa-mir-96
SYNPR-AS1	hsa-mir-182
SYNPR-AS1	hsa-mir-383
KLHL6-AS1	hsa-mir-96
KLHL6-AS1	hsa-mir-144
GK-AS1	hsa-mir-429
AL133367.1	hsa-mir-383
AC073352.1	hsa-mir-96
AC073352.1	hsa-mir-182
HOTTIP	hsa-mir-141
HOTTIP	hsa-mir-200a
HOTTIP	hsa-mir-187
HOTTIP	hsa-mir-211
HOTTIP	hsa-mir-31
AL445228.2	hsa-mir-383
CRNDE	hsa-mir-144
CRNDE	hsa-mir-183
CRNDE	hsa-mir-222
CRNDE	hsa-mir-31
AL139147.1	hsa-mir-182
AL139147.1	hsa-mir-383
PVT1	hsa-mir-183
PVT1	hsa-mir-187
PVT1	hsa-mir-21
PVT1	hsa-mir-222
PVT1	hsa-mir-31
PVT1	hsa-mir-383
AP001781.1	hsa-mir-211
AC147651.1	hsa-mir-222
NRG1-IT1	hsa-mir-141
NRG1-IT1	hsa-mir-200a
NRG1-IT1	hsa-mir-144
NRG1-IT1	hsa-mir-383
FAM66D	hsa-mir-141
FAM66D	hsa-mir-200a
FAM66D	hsa-mir-187
FAM66D	hsa-mir-222
GRM5-AS1	hsa-mir-96
GRM5-AS1	hsa-mir-144
GRM5-AS1	hsa-mir-182

GRM5-AS1	hsa-mir-429
GRM5-AS1	hsa-mir-21
GRM5-AS1	hsa-mir-31
GRM5-AS1	hsa-mir-383
SNHG1	hsa-mir-141
SNHG1	hsa-mir-200a
SNHG1	hsa-mir-144
SNHG1	hsa-mir-182
SNHG1	hsa-mir-211
SNHG1	hsa-mir-21
SNHG1	hsa-mir-122
SNHG1	hsa-mir-383
AC110619.1	hsa-mir-122
AL139385.1	hsa-mir-183
AC093734.1	hsa-mir-187
AC093734.1	hsa-mir-122
AP006285.1	hsa-mir-222
AP006285.1	hsa-mir-122
RERG-IT1	hsa-mir-182
RERG-IT1	hsa-mir-21
AP001029.2	hsa-mir-31
AL021707.2	hsa-mir-383
LINC00261	hsa-mir-144
LINC00261	hsa-mir-182
LINC00261	hsa-mir-183
LINC00261	hsa-mir-429
LINC00261	hsa-mir-211
LINC00261	hsa-mir-31
FBXL19-AS1	hsa-mir-141
FBXL19-AS1	hsa-mir-200a
FBXL19-AS1	hsa-mir-122
DNM1P35	hsa-mir-122

Table S4. Thirteen DEmiRNAs interact with the sixty DEmRNAs retrieved from the miRDB, miRTarBase and TargetScan databases.

DEmiRNA	DEmRNA
hsa-mir-211	FOXC1
hsa-mir-383	DIO1
hsa-mir-200a	KIAA1549
hsa-mir-182	MTSS1
hsa-mir-200a	SCD5
hsa-mir-31	FOXD4L1
hsa-mir-211	SLC43A1
hsa-mir-141	SCD5
hsa-mir-182	NPTX1
hsa-mir-21	EDIL3
hsa-mir-122	SLC52A2

hsa-mir-211	SAMD5
hsa-mir-182	RARG
hsa-mir-222	ESR1
hsa-mir-21	FAM46A
hsa-mir-21	ELOVL7
hsa-mir-144	ELL2
hsa-mir-211	ELOVL6
hsa-mir-211	NPTX1
hsa-mir-200a	SLC35D1
hsa-mir-182	MITF
hsa-mir-211	HOXC8
hsa-mir-144	FZD6
hsa-mir-182	BDNF
hsa-mir-21	SCRN1
hsa-mir-211	TMTC2
hsa-mir-183	KIF5C
hsa-mir-429	SHCBP1
hsa-mir-21	NTF3
hsa-mir-122	SLC7A1
hsa-mir-211	ZCCHC24
hsa-mir-31	HOXC13
hsa-mir-141	KIAA1549
hsa-mir-211	IL11
hsa-mir-96	TMEM170B
hsa-mir-144	MFSD6
hsa-mir-96	TSKU
hsa-mir-122	PKM
hsa-mir-122	GALNT3
hsa-mir-141	HOXB5
hsa-mir-21	PIK3R1
hsa-mir-21	CPEB3
hsa-mir-200a	HOXB5
hsa-mir-122	ALDOA
hsa-mir-21	PTPN14
hsa-mir-200a	MACC1
hsa-mir-182	FGF9
hsa-mir-211	CREB5
hsa-mir-222	FOS
hsa-mir-200a	EPHA7
hsa-mir-96	PRDM16
hsa-mir-200a	UBASH3B
hsa-mir-141	TGFB2
hsa-mir-21	JAG1
hsa-mir-183	CCNB1
hsa-mir-31	FOXD4
hsa-mir-141	EPHA7
hsa-mir-429	PMAIP1

hsa-mir-222	GALNT3
hsa-mir-141	MACC1
hsa-mir-96	SCARB1
hsa-mir-141	SLC35D1
hsa-mir-182	FOXF2
hsa-mir-144	HOXA10
hsa-mir-96	SLC25A25
hsa-mir-144	PANK1
hsa-mir-21	EPM2A
hsa-mir-21	JPH1

Table S5. The interactions of the ceRNA network in cholangiocarcinoma.

lncRNA	miRNA	mRNA
AL591845.1	hsa-mir-96	TMEM170B
AL591845.1	hsa-mir-182	MTSS1
AL591845.1	hsa-mir-122	SLC52A2
AL591845.1	hsa-mir-383	DIO1
KIAA0087	hsa-mir-96	TMEM170B
KIAA0087	hsa-mir-141	SCD5
KIAA0087	hsa-mir-200a	KIAA1549
KIAA0087	hsa-mir-182	MTSS1
KIAA0087	hsa-mir-183	KIF5C
KIAA0087	hsa-mir-429	SHCBP1
KIAA0087	hsa-mir-211	FOXC1
KIAA0087	hsa-mir-383	DIO1
H19	hsa-mir-141	SCD5
H19	hsa-mir-200a	KIAA1549
C2orf48	hsa-mir-183	KIF5C
C2orf48	hsa-mir-211	FOXC1
C2orf48	hsa-mir-122	SLC52A2
AC005280.1	hsa-mir-96	TMEM170B
AC005280.1	hsa-mir-182	MTSS1
AC127496.1	hsa-mir-96	TMEM170B
AC127496.1	hsa-mir-182	MTSS1
AC127496.1	hsa-mir-183	KIF5C
AC127496.1	hsa-mir-211	FOXC1
AC127496.1	hsa-mir-122	SLC52A2
C15orf56	hsa-mir-144	ELL2
C15orf56	hsa-mir-182	MTSS1
C15orf56	hsa-mir-183	KIF5C
C15orf56	hsa-mir-122	SLC52A2
AC012074.1	hsa-mir-122	SLC52A2
C10orf91	hsa-mir-429	SHCBP1
C10orf91	hsa-mir-211	FOXC1
C10orf91	hsa-mir-122	SLC52A2
LINC00304	hsa-mir-183	KIF5C
LINC00304	hsa-mir-222	ESR1

FAM87A	hsa-mir-96	TMEM170B
FAM87A	hsa-mir-141	SCD5
FAM87A	hsa-mir-200a	KIAA1549
FAM87A	hsa-mir-222	ESR1
FAM87A	hsa-mir-31	FOXD4L1
LINC00315	hsa-mir-183	KIF5C
LINC00313	hsa-mir-211	FOXC1
LINC00313	hsa-mir-122	SLC52A2
LINC00313	hsa-mir-31	FOXD4L1
C9orf163	hsa-mir-21	EDIL3
AC087392.1	hsa-mir-144	ELL2
AC087392.1	hsa-mir-429	SHCBP1
AC087392.1	hsa-mir-222	ESR1
LINC00336	hsa-mir-96	TMEM170B
LINC00336	hsa-mir-21	EDIL3
AC116351.1	hsa-mir-31	FOXD4L1
PSORS1C3	hsa-mir-211	FOXC1
FAM201A	hsa-mir-96	TMEM170B
FAM201A	hsa-mir-144	ELL2
FAM201A	hsa-mir-182	MTSS1
FAM201A	hsa-mir-21	EDIL3
FAM201A	hsa-mir-222	ESR1
CLLU1	hsa-mir-141	SCD5
CLLU1	hsa-mir-200a	KIAA1549
CLLU1	hsa-mir-144	ELL2
CLLU1	hsa-mir-429	SHCBP1
CLLU1	hsa-mir-211	FOXC1
CLLU1	hsa-mir-21	EDIL3
CLLU1	hsa-mir-222	ESR1
CLLU1	hsa-mir-31	FOXD4L1
TTLL10-AS1	hsa-mir-122	SLC52A2
AL359878.1	hsa-mir-141	SCD5
AL359878.1	hsa-mir-200a	KIAA1549
FAM99A	hsa-mir-122	SLC52A2
AC108134.1	hsa-mir-182	MTSS1
AC108134.1	hsa-mir-183	KIF5C
AC092117.1	hsa-mir-141	SCD5
AC092117.1	hsa-mir-200a	KIAA1549
AC092117.1	hsa-mir-211	FOXC1
AP000525.1	hsa-mir-31	FOXD4L1
AL137145.1	hsa-mir-211	FOXC1
AL137145.1	hsa-mir-222	ESR1
AL137145.1	hsa-mir-31	FOXD4L1
AL021068.1	hsa-mir-429	SHCBP1
AL021068.1	hsa-mir-211	FOXC1
AL021068.1	hsa-mir-122	SLC52A2
UCA1	hsa-mir-96	TMEM170B

UCA1	hsa-mir-182	MTSS1
UCA1	hsa-mir-122	SLC52A2
UCA1	hsa-mir-383	DIO1
AC010336.2	hsa-mir-141	SCD5
AC010336.2	hsa-mir-200a	KIAA1549
AC010336.2	hsa-mir-144	ELL2
AC010336.2	hsa-mir-182	MTSS1
AC010336.2	hsa-mir-429	SHCBP1
AC010336.2	hsa-mir-211	FOXC1
AC010336.2	hsa-mir-122	SLC52A2
AC010336.2	hsa-mir-31	FOXD4L1
AC011481.1	hsa-mir-211	FOXC1
AL137145.2	hsa-mir-144	ELL2
AL137145.2	hsa-mir-182	MTSS1
AL137145.2	hsa-mir-429	SHCBP1
AL137145.2	hsa-mir-31	FOXD4L1
AC016773.1	hsa-mir-122	SLC52A2
AC016773.1	hsa-mir-31	FOXD4L1
AL354984.1	hsa-mir-429	SHCBP1
AC090150.1	hsa-mir-182	MTSS1
AC080037.1	hsa-mir-429	SHCBP1
AC096642.1	hsa-mir-96	TMEM170B
CLDN10-AS1	hsa-mir-222	ESR1
POU6F2-AS1	hsa-mir-144	ELL2
LINC00466	hsa-mir-96	TMEM170B
LINC00466	hsa-mir-141	SCD5
LINC00466	hsa-mir-200a	KIAA1549
LINC00466	hsa-mir-144	ELL2
LINC00466	hsa-mir-183	KIF5C
LINC00466	hsa-mir-429	SHCBP1
LINC00466	hsa-mir-211	FOXC1
LINC00466	hsa-mir-21	EDIL3
WARS2-IT1	hsa-mir-144	ELL2
THRB-IT1	hsa-mir-429	SHCBP1
THRB-IT1	hsa-mir-211	FOXC1
LINC00184	hsa-mir-31	FOXD4L1
LINC00337	hsa-mir-182	MTSS1
LINC00337	hsa-mir-383	DIO1
SFTA1P	hsa-mir-182	MTSS1
SFTA1P	hsa-mir-211	FOXC1
SFTA1P	hsa-mir-222	ESR1
SFTA1P	hsa-mir-122	SLC52A2
BOLA3-AS1	hsa-mir-182	MTSS1
BOLA3-AS1	hsa-mir-429	SHCBP1
BOLA3-AS1	hsa-mir-211	FOXC1
BOLA3-AS1	hsa-mir-122	SLC52A2
BOLA3-AS1	hsa-mir-383	DIO1

DBH-AS1	hsa-mir-211	FOXC1
AL356356.1	hsa-mir-141	SCD5
AL356356.1	hsa-mir-200a	KIAA1549
AL356356.1	hsa-mir-211	FOXC1
LINC00323	hsa-mir-96	TMEM170B
LINC00323	hsa-mir-182	MTSS1
LINC00323	hsa-mir-211	FOXC1
FAM66C	hsa-mir-183	KIF5C
FAM66C	hsa-mir-429	SHCBP1
FAM66C	hsa-mir-211	FOXC1
FAM66C	hsa-mir-21	EDIL3
FAM66C	hsa-mir-222	ESR1
FAM66C	hsa-mir-122	SLC52A2
LINC00348	hsa-mir-141	SCD5
LINC00348	hsa-mir-200a	KIAA1549
LINC00348	hsa-mir-144	ELL2
HOTAIR	hsa-mir-211	FOXC1
HOTAIR	hsa-mir-21	EDIL3
HOTAIR	hsa-mir-222	ESR1
LINC00242	hsa-mir-96	TMEM170B
LINC00242	hsa-mir-141	SCD5
LINC00242	hsa-mir-200a	KIAA1549
LINC00242	hsa-mir-211	FOXC1
LINC00242	hsa-mir-222	ESR1
LINC00242	hsa-mir-31	FOXD4L1
LRRC3-AS1	hsa-mir-96	TMEM170B
LRRC3-AS1	hsa-mir-182	MTSS1
LRRC3-AS1	hsa-mir-211	FOXC1
SZT2-AS1	hsa-mir-183	KIF5C
UBE2Q1-AS1	hsa-mir-96	TMEM170B
UBE2Q1-AS1	hsa-mir-182	MTSS1
UBE2Q1-AS1	hsa-mir-211	FOXC1
LINC00443	hsa-mir-141	SCD5
LINC00443	hsa-mir-200a	KIAA1549
LINC00443	hsa-mir-144	ELL2
LINC00443	hsa-mir-183	KIF5C
LINC00443	hsa-mir-211	FOXC1
HM13-AS1	hsa-mir-31	FOXD4L1
AL161645.1	hsa-mir-96	TMEM170B
AL161645.1	hsa-mir-144	ELL2
AL161645.1	hsa-mir-211	FOXC1
AL161645.1	hsa-mir-122	SLC52A2
DLG3-AS1	hsa-mir-222	ESR1
DLX6-AS1	hsa-mir-141	SCD5
DLX6-AS1	hsa-mir-200a	KIAA1549
DLX6-AS1	hsa-mir-144	ELL2
DLX6-AS1	hsa-mir-429	SHCBP1

DLX6-AS1	hsa-mir-211	FOXC1
DLX6-AS1	hsa-mir-122	SLC52A2
DLX6-AS1	hsa-mir-31	FOXD4L1
DLX6-AS1	hsa-mir-383	DIO1
F10-AS1	hsa-mir-182	MTSS1
HS1BP3-IT1	hsa-mir-21	EDIL3
SLC6A1-AS1	hsa-mir-383	DIO1
FAM215B	hsa-mir-31	FOXD4L1
AC037487.1	hsa-mir-222	ESR1
AC110491.1	hsa-mir-141	SCD5
AC110491.1	hsa-mir-200a	KIAA1549
AC110491.1	hsa-mir-182	MTSS1
AC110491.1	hsa-mir-429	SHCBP1
AC110491.1	hsa-mir-211	FOXC1
AC110491.1	hsa-mir-222	ESR1
LINC00460	hsa-mir-429	SHCBP1
LINC00460	hsa-mir-222	ESR1
TPRG1-AS1	hsa-mir-182	MTSS1
AC092171.1	hsa-mir-96	TMEM170B
AC092171.1	hsa-mir-182	MTSS1
AC092171.1	hsa-mir-383	DIO1
MAGI2-AS3	hsa-mir-141	SCD5
MAGI2-AS3	hsa-mir-200a	KIAA1549
MAGI2-AS3	hsa-mir-144	ELL2
MAGI2-AS3	hsa-mir-429	SHCBP1
MAGI2-AS3	hsa-mir-211	FOXC1
MAGI2-AS3	hsa-mir-122	SLC52A2
MAGI2-AS3	hsa-mir-31	FOXD4L1
GAS5	hsa-mir-96	TMEM170B
GAS5	hsa-mir-144	ELL2
GAS5	hsa-mir-182	MTSS1
GAS5	hsa-mir-429	SHCBP1
GAS5	hsa-mir-21	EDIL3
GAS5	hsa-mir-222	ESR1
GAS5	hsa-mir-31	FOXD4L1
MLIP-AS1	hsa-mir-144	ELL2
MLIP-AS1	hsa-mir-222	ESR1
MLIP-AS1	hsa-mir-383	DIO1
LINC00402	hsa-mir-141	SCD5
LINC00402	hsa-mir-200a	KIAA1549
LINC00402	hsa-mir-182	MTSS1
LINC00402	hsa-mir-429	SHCBP1
LINC00402	hsa-mir-211	FOXC1
LINC00402	hsa-mir-383	DIO1
LINC00494	hsa-mir-182	MTSS1
LINC00494	hsa-mir-222	ESR1
LINC00494	hsa-mir-31	FOXD4L1

LINC00494	hsa-mir-383	DIO1
SAPCD1-AS1	hsa-mir-211	FOXC1
NEXN-AS1	hsa-mir-211	FOXC1
AC015987.1	hsa-mir-429	SHCBP1
AC015987.1	hsa-mir-211	FOXC1
AC015987.1	hsa-mir-383	DIO1
LINC00316	hsa-mir-122	SLC52A2
AL137798.1	hsa-mir-211	FOXC1
AL137798.1	hsa-mir-122	SLC52A2
AC009121.1	hsa-mir-141	SCD5
AC009121.1	hsa-mir-200a	KIAA1549
CLRN1-AS1	hsa-mir-429	SHCBP1
CLRN1-AS1	hsa-mir-211	FOXC1
CLRN1-AS1	hsa-mir-222	ESR1
AL391832.1	hsa-mir-383	DIO1
AP004609.1	hsa-mir-383	DIO1
ITIH4-AS1	hsa-mir-211	FOXC1
ITIH4-AS1	hsa-mir-122	SLC52A2
AC080129.1	hsa-mir-122	SLC52A2
AC080129.1	hsa-mir-383	DIO1
AL118511.1	hsa-mir-383	DIO1
TM4SF1-AS1	hsa-mir-141	SCD5
TM4SF1-AS1	hsa-mir-200a	KIAA1549
ARHGAP31-AS1	hsa-mir-122	SLC52A2
AL359541.1	hsa-mir-122	SLC52A2
SYNPR-AS1	hsa-mir-96	TMEM170B
SYNPR-AS1	hsa-mir-182	MTSS1
SYNPR-AS1	hsa-mir-383	DIO1
KLHL6-AS1	hsa-mir-96	TMEM170B
KLHL6-AS1	hsa-mir-144	ELL2
GK-AS1	hsa-mir-429	SHCBP1
AL133367.1	hsa-mir-383	DIO1
AC073352.1	hsa-mir-96	TMEM170B
AC073352.1	hsa-mir-182	MTSS1
HOTTIP	hsa-mir-141	SCD5
HOTTIP	hsa-mir-200a	KIAA1549
HOTTIP	hsa-mir-211	FOXC1
HOTTIP	hsa-mir-31	FOXD4L1
AL445228.2	hsa-mir-383	DIO1
CRNDE	hsa-mir-144	ELL2
CRNDE	hsa-mir-183	KIF5C
CRNDE	hsa-mir-222	ESR1
CRNDE	hsa-mir-31	FOXD4L1
AL139147.1	hsa-mir-182	MTSS1
AL139147.1	hsa-mir-383	DIO1
PVT1	hsa-mir-183	KIF5C
PVT1	hsa-mir-21	EDIL3

PVT1	hsa-mir-222	ESR1
PVT1	hsa-mir-31	FOXD4L1
PVT1	hsa-mir-383	DIO1
AP001781.1	hsa-mir-211	FOXC1
AC147651.1	hsa-mir-222	ESR1
NRG1-IT1	hsa-mir-141	SCD5
NRG1-IT1	hsa-mir-200a	KIAA1549
NRG1-IT1	hsa-mir-144	ELL2
NRG1-IT1	hsa-mir-383	DIO1
FAM66D	hsa-mir-141	SCD5
FAM66D	hsa-mir-200a	KIAA1549
FAM66D	hsa-mir-222	ESR1
GRM5-AS1	hsa-mir-96	TMEM170B
GRM5-AS1	hsa-mir-144	ELL2
GRM5-AS1	hsa-mir-182	MTSS1
GRM5-AS1	hsa-mir-429	SHCBP1
GRM5-AS1	hsa-mir-21	EDIL3
GRM5-AS1	hsa-mir-31	FOXD4L1
GRM5-AS1	hsa-mir-383	DIO1
SNHG1	hsa-mir-141	SCD5
SNHG1	hsa-mir-200a	KIAA1549
SNHG1	hsa-mir-144	ELL2
SNHG1	hsa-mir-182	MTSS1
SNHG1	hsa-mir-211	FOXC1
SNHG1	hsa-mir-21	EDIL3
SNHG1	hsa-mir-122	SLC52A2
SNHG1	hsa-mir-383	DIO1
AC110619.1	hsa-mir-122	SLC52A2
AL139385.1	hsa-mir-183	KIF5C
AC093734.1	hsa-mir-122	SLC52A2
AP006285.1	hsa-mir-222	ESR1
AP006285.1	hsa-mir-122	SLC52A2
RERG-IT1	hsa-mir-182	MTSS1
RERG-IT1	hsa-mir-21	EDIL3
AP001029.2	hsa-mir-31	FOXD4L1
AL021707.2	hsa-mir-383	DIO1
LINC00261	hsa-mir-144	ELL2
LINC00261	hsa-mir-182	MTSS1
LINC00261	hsa-mir-183	KIF5C
LINC00261	hsa-mir-429	SHCBP1
LINC00261	hsa-mir-211	FOXC1
LINC00261	hsa-mir-31	FOXD4L1
FBXL19-AS1	hsa-mir-141	SCD5
FBXL19-AS1	hsa-mir-200a	KIAA1549
FBXL19-AS1	hsa-mir-122	SLC52A2
DNM1P35	hsa-mir-122	SLC52A2
AL591845.1	hsa-mir-122	SLC7A1

C2orf48	hsa-mir-122	SLC7A1
AC127496.1	hsa-mir-122	SLC7A1
C15orf56	hsa-mir-122	SLC7A1
AC012074.1	hsa-mir-122	SLC7A1
C10orf91	hsa-mir-122	SLC7A1
LINC00313	hsa-mir-122	SLC7A1
TTLL10-AS1	hsa-mir-122	SLC7A1
FAM99A	hsa-mir-122	SLC7A1
AL021068.1	hsa-mir-122	SLC7A1
UCA1	hsa-mir-122	SLC7A1
AC010336.2	hsa-mir-122	SLC7A1
AC016773.1	hsa-mir-122	SLC7A1
SFTA1P	hsa-mir-122	SLC7A1
BOLA3-AS1	hsa-mir-122	SLC7A1
FAM66C	hsa-mir-122	SLC7A1
AL161645.1	hsa-mir-122	SLC7A1
DLX6-AS1	hsa-mir-122	SLC7A1
MAGI2-AS3	hsa-mir-122	SLC7A1
LINC00316	hsa-mir-122	SLC7A1
AL137798.1	hsa-mir-122	SLC7A1
ITIH4-AS1	hsa-mir-122	SLC7A1
AC080129.1	hsa-mir-122	SLC7A1
ARHGAP31-AS1	hsa-mir-122	SLC7A1
AL359541.1	hsa-mir-122	SLC7A1
SNHG1	hsa-mir-122	SLC7A1
AC110619.1	hsa-mir-122	SLC7A1
AC093734.1	hsa-mir-122	SLC7A1
AP006285.1	hsa-mir-122	SLC7A1
FBXL19-AS1	hsa-mir-122	SLC7A1
DNM1P35	hsa-mir-122	SLC7A1
AL591845.1	hsa-mir-122	PKM
C2orf48	hsa-mir-122	PKM
AC127496.1	hsa-mir-122	PKM
C15orf56	hsa-mir-122	PKM
AC012074.1	hsa-mir-122	PKM
C10orf91	hsa-mir-122	PKM
LINC00313	hsa-mir-122	PKM
TTLL10-AS1	hsa-mir-122	PKM
FAM99A	hsa-mir-122	PKM
AL021068.1	hsa-mir-122	PKM
UCA1	hsa-mir-122	PKM
AC010336.2	hsa-mir-122	PKM
AC016773.1	hsa-mir-122	PKM
SFTA1P	hsa-mir-122	PKM
BOLA3-AS1	hsa-mir-122	PKM
FAM66C	hsa-mir-122	PKM
AL161645.1	hsa-mir-122	PKM

DLX6-AS1	hsa-mir-122	PKM
MAGI2-AS3	hsa-mir-122	PKM
LINC00316	hsa-mir-122	PKM
AL137798.1	hsa-mir-122	PKM
ITIH4-AS1	hsa-mir-122	PKM
AC080129.1	hsa-mir-122	PKM
ARHGAP31-AS1	hsa-mir-122	PKM
AL359541.1	hsa-mir-122	PKM
SNHG1	hsa-mir-122	PKM
AC110619.1	hsa-mir-122	PKM
AC093734.1	hsa-mir-122	PKM
AP006285.1	hsa-mir-122	PKM
FBXL19-AS1	hsa-mir-122	PKM
DNM1P35	hsa-mir-122	PKM
AL591845.1	hsa-mir-122	GALNT3
C2orf48	hsa-mir-122	GALNT3
AC127496.1	hsa-mir-122	GALNT3
C15orf56	hsa-mir-122	GALNT3
AC012074.1	hsa-mir-122	GALNT3
C10orf91	hsa-mir-122	GALNT3
LINC00313	hsa-mir-122	GALNT3
TTLL10-AS1	hsa-mir-122	GALNT3
FAM99A	hsa-mir-122	GALNT3
AL021068.1	hsa-mir-122	GALNT3
UCA1	hsa-mir-122	GALNT3
AC010336.2	hsa-mir-122	GALNT3
AC016773.1	hsa-mir-122	GALNT3
SFTA1P	hsa-mir-122	GALNT3
BOLA3-AS1	hsa-mir-122	GALNT3
FAM66C	hsa-mir-122	GALNT3
AL161645.1	hsa-mir-122	GALNT3
DLX6-AS1	hsa-mir-122	GALNT3
MAGI2-AS3	hsa-mir-122	GALNT3
LINC00316	hsa-mir-122	GALNT3
AL137798.1	hsa-mir-122	GALNT3
ITIH4-AS1	hsa-mir-122	GALNT3
AC080129.1	hsa-mir-122	GALNT3
ARHGAP31-AS1	hsa-mir-122	GALNT3
AL359541.1	hsa-mir-122	GALNT3
SNHG1	hsa-mir-122	GALNT3
AC110619.1	hsa-mir-122	GALNT3
AC093734.1	hsa-mir-122	GALNT3
AP006285.1	hsa-mir-122	GALNT3
FBXL19-AS1	hsa-mir-122	GALNT3
DNM1P35	hsa-mir-122	GALNT3
AL591845.1	hsa-mir-122	ALDOA
C2orf48	hsa-mir-122	ALDOA

AC127496.1	hsa-mir-122	ALDOA
C15orf56	hsa-mir-122	ALDOA
AC012074.1	hsa-mir-122	ALDOA
C10orf91	hsa-mir-122	ALDOA
LINC00313	hsa-mir-122	ALDOA
TTLL10-AS1	hsa-mir-122	ALDOA
FAM99A	hsa-mir-122	ALDOA
AL021068.1	hsa-mir-122	ALDOA
UCA1	hsa-mir-122	ALDOA
AC010336.2	hsa-mir-122	ALDOA
AC016773.1	hsa-mir-122	ALDOA
SFTA1P	hsa-mir-122	ALDOA
BOLA3-AS1	hsa-mir-122	ALDOA
FAM66C	hsa-mir-122	ALDOA
AL161645.1	hsa-mir-122	ALDOA
DLX6-AS1	hsa-mir-122	ALDOA
MAGI2-AS3	hsa-mir-122	ALDOA
LINC00316	hsa-mir-122	ALDOA
AL137798.1	hsa-mir-122	ALDOA
ITIH4-AS1	hsa-mir-122	ALDOA
AC080129.1	hsa-mir-122	ALDOA
ARHGAP31-AS1	hsa-mir-122	ALDOA
AL359541.1	hsa-mir-122	ALDOA
SNHG1	hsa-mir-122	ALDOA
AC110619.1	hsa-mir-122	ALDOA
AC093734.1	hsa-mir-122	ALDOA
AP006285.1	hsa-mir-122	ALDOA
FBXL19-AS1	hsa-mir-122	ALDOA
DNM1P35	hsa-mir-122	ALDOA
KIAA0087	hsa-mir-141	KIAA1549
H19	hsa-mir-141	KIAA1549
FAM87A	hsa-mir-141	KIAA1549
CLLU1	hsa-mir-141	KIAA1549
AL359878.1	hsa-mir-141	KIAA1549
AC092117.1	hsa-mir-141	KIAA1549
AC010336.2	hsa-mir-141	KIAA1549
LINC00466	hsa-mir-141	KIAA1549
AL356356.1	hsa-mir-141	KIAA1549
LINC00348	hsa-mir-141	KIAA1549
LINC00242	hsa-mir-141	KIAA1549
LINC00443	hsa-mir-141	KIAA1549
DLX6-AS1	hsa-mir-141	KIAA1549
AC110491.1	hsa-mir-141	KIAA1549
MAGI2-AS3	hsa-mir-141	KIAA1549
LINC00402	hsa-mir-141	KIAA1549
AC009121.1	hsa-mir-141	KIAA1549
TM4SF1-AS1	hsa-mir-141	KIAA1549

HOTTIP	hsa-mir-141	KIAA1549
NRG1-IT1	hsa-mir-141	KIAA1549
FAM66D	hsa-mir-141	KIAA1549
SNHG1	hsa-mir-141	KIAA1549
FBXL19-AS1	hsa-mir-141	KIAA1549
KIAA0087	hsa-mir-141	HOXB5
H19	hsa-mir-141	HOXB5
FAM87A	hsa-mir-141	HOXB5
CLLU1	hsa-mir-141	HOXB5
AL359878.1	hsa-mir-141	HOXB5
AC092117.1	hsa-mir-141	HOXB5
AC010336.2	hsa-mir-141	HOXB5
LINC00466	hsa-mir-141	HOXB5
AL356356.1	hsa-mir-141	HOXB5
LINC00348	hsa-mir-141	HOXB5
LINC00242	hsa-mir-141	HOXB5
LINC00443	hsa-mir-141	HOXB5
DLX6-AS1	hsa-mir-141	HOXB5
AC110491.1	hsa-mir-141	HOXB5
MAGI2-AS3	hsa-mir-141	HOXB5
LINC00402	hsa-mir-141	HOXB5
AC009121.1	hsa-mir-141	HOXB5
TM4SF1-AS1	hsa-mir-141	HOXB5
HOTTIP	hsa-mir-141	HOXB5
NRG1-IT1	hsa-mir-141	HOXB5
FAM66D	hsa-mir-141	HOXB5
SNHG1	hsa-mir-141	HOXB5
FBXL19-AS1	hsa-mir-141	HOXB5
KIAA0087	hsa-mir-141	TGFB2
H19	hsa-mir-141	TGFB2
FAM87A	hsa-mir-141	TGFB2
CLLU1	hsa-mir-141	TGFB2
AL359878.1	hsa-mir-141	TGFB2
AC092117.1	hsa-mir-141	TGFB2
AC010336.2	hsa-mir-141	TGFB2
LINC00466	hsa-mir-141	TGFB2
AL356356.1	hsa-mir-141	TGFB2
LINC00348	hsa-mir-141	TGFB2
LINC00242	hsa-mir-141	TGFB2
LINC00443	hsa-mir-141	TGFB2
DLX6-AS1	hsa-mir-141	TGFB2
AC110491.1	hsa-mir-141	TGFB2
MAGI2-AS3	hsa-mir-141	TGFB2
LINC00402	hsa-mir-141	TGFB2
AC009121.1	hsa-mir-141	TGFB2
TM4SF1-AS1	hsa-mir-141	TGFB2
HOTTIP	hsa-mir-141	TGFB2

NRG1-IT1	hsa-mir-141	TGFB2
FAM66D	hsa-mir-141	TGFB2
SNHG1	hsa-mir-141	TGFB2
FBXL19-AS1	hsa-mir-141	TGFB2
KIAA0087	hsa-mir-141	EPHA7
H19	hsa-mir-141	EPHA7
FAM87A	hsa-mir-141	EPHA7
CLLU1	hsa-mir-141	EPHA7
AL359878.1	hsa-mir-141	EPHA7
AC092117.1	hsa-mir-141	EPHA7
AC010336.2	hsa-mir-141	EPHA7
LINC00466	hsa-mir-141	EPHA7
AL356356.1	hsa-mir-141	EPHA7
LINC00348	hsa-mir-141	EPHA7
LINC00242	hsa-mir-141	EPHA7
LINC00443	hsa-mir-141	EPHA7
DLX6-AS1	hsa-mir-141	EPHA7
AC110491.1	hsa-mir-141	EPHA7
MAGI2-AS3	hsa-mir-141	EPHA7
LINC00402	hsa-mir-141	EPHA7
AC009121.1	hsa-mir-141	EPHA7
TM4SF1-AS1	hsa-mir-141	EPHA7
HOTTIP	hsa-mir-141	EPHA7
NRG1-IT1	hsa-mir-141	EPHA7
FAM66D	hsa-mir-141	EPHA7
SNHG1	hsa-mir-141	EPHA7
FBXL19-AS1	hsa-mir-141	EPHA7
KIAA0087	hsa-mir-141	MACC1
H19	hsa-mir-141	MACC1
FAM87A	hsa-mir-141	MACC1
CLLU1	hsa-mir-141	MACC1
AL359878.1	hsa-mir-141	MACC1
AC092117.1	hsa-mir-141	MACC1
AC010336.2	hsa-mir-141	MACC1
LINC00466	hsa-mir-141	MACC1
AL356356.1	hsa-mir-141	MACC1
LINC00348	hsa-mir-141	MACC1
LINC00242	hsa-mir-141	MACC1
LINC00443	hsa-mir-141	MACC1
DLX6-AS1	hsa-mir-141	MACC1
AC110491.1	hsa-mir-141	MACC1
MAGI2-AS3	hsa-mir-141	MACC1
LINC00402	hsa-mir-141	MACC1
AC009121.1	hsa-mir-141	MACC1
TM4SF1-AS1	hsa-mir-141	MACC1
HOTTIP	hsa-mir-141	MACC1
NRG1-IT1	hsa-mir-141	MACC1

FAM66D	hsa-mir-141	MACC1
SNHG1	hsa-mir-141	MACC1
FBXL19-AS1	hsa-mir-141	MACC1
KIAA0087	hsa-mir-141	SLC35D1
H19	hsa-mir-141	SLC35D1
FAM87A	hsa-mir-141	SLC35D1
CLLU1	hsa-mir-141	SLC35D1
AL359878.1	hsa-mir-141	SLC35D1
AC092117.1	hsa-mir-141	SLC35D1
AC010336.2	hsa-mir-141	SLC35D1
LINC00466	hsa-mir-141	SLC35D1
AL356356.1	hsa-mir-141	SLC35D1
LINC00348	hsa-mir-141	SLC35D1
LINC00242	hsa-mir-141	SLC35D1
LINC00443	hsa-mir-141	SLC35D1
DLX6-AS1	hsa-mir-141	SLC35D1
AC110491.1	hsa-mir-141	SLC35D1
MAGI2-AS3	hsa-mir-141	SLC35D1
LINC00402	hsa-mir-141	SLC35D1
AC009121.1	hsa-mir-141	SLC35D1
TM4SF1-AS1	hsa-mir-141	SLC35D1
HOTTIP	hsa-mir-141	SLC35D1
NRG1-IT1	hsa-mir-141	SLC35D1
FAM66D	hsa-mir-141	SLC35D1
SNHG1	hsa-mir-141	SLC35D1
FBXL19-AS1	hsa-mir-141	SLC35D1
C15orf56	hsa-mir-144	FZD6
AC087392.1	hsa-mir-144	FZD6
FAM201A	hsa-mir-144	FZD6
CLLU1	hsa-mir-144	FZD6
AC010336.2	hsa-mir-144	FZD6
AL137145.2	hsa-mir-144	FZD6
POU6F2-AS1	hsa-mir-144	FZD6
LINC00466	hsa-mir-144	FZD6
WARS2-IT1	hsa-mir-144	FZD6
LINC00348	hsa-mir-144	FZD6
LINC00443	hsa-mir-144	FZD6
AL161645.1	hsa-mir-144	FZD6
DLX6-AS1	hsa-mir-144	FZD6
MAGI2-AS3	hsa-mir-144	FZD6
GAS5	hsa-mir-144	FZD6
MLIP-AS1	hsa-mir-144	FZD6
KLHL6-AS1	hsa-mir-144	FZD6
CRNDE	hsa-mir-144	FZD6
NRG1-IT1	hsa-mir-144	FZD6
GRM5-AS1	hsa-mir-144	FZD6
SNHG1	hsa-mir-144	FZD6

LINC00261	hsa-mir-144	FZD6
C15orf56	hsa-mir-144	MFSD6
AC087392.1	hsa-mir-144	MFSD6
FAM201A	hsa-mir-144	MFSD6
CLLU1	hsa-mir-144	MFSD6
AC010336.2	hsa-mir-144	MFSD6
AL137145.2	hsa-mir-144	MFSD6
POU6F2-AS1	hsa-mir-144	MFSD6
LINC00466	hsa-mir-144	MFSD6
WARS2-IT1	hsa-mir-144	MFSD6
LINC00348	hsa-mir-144	MFSD6
LINC00443	hsa-mir-144	MFSD6
AL161645.1	hsa-mir-144	MFSD6
DLX6-AS1	hsa-mir-144	MFSD6
MAGI2-AS3	hsa-mir-144	MFSD6
GAS5	hsa-mir-144	MFSD6
MLIP-AS1	hsa-mir-144	MFSD6
KLHL6-AS1	hsa-mir-144	MFSD6
CRNDE	hsa-mir-144	MFSD6
NRG1-IT1	hsa-mir-144	MFSD6
GRM5-AS1	hsa-mir-144	MFSD6
SNHG1	hsa-mir-144	MFSD6
LINC00261	hsa-mir-144	MFSD6
C15orf56	hsa-mir-144	HOXA10
AC087392.1	hsa-mir-144	HOXA10
FAM201A	hsa-mir-144	HOXA10
CLLU1	hsa-mir-144	HOXA10
AC010336.2	hsa-mir-144	HOXA10
AL137145.2	hsa-mir-144	HOXA10
POU6F2-AS1	hsa-mir-144	HOXA10
LINC00466	hsa-mir-144	HOXA10
WARS2-IT1	hsa-mir-144	HOXA10
LINC00348	hsa-mir-144	HOXA10
LINC00443	hsa-mir-144	HOXA10
AL161645.1	hsa-mir-144	HOXA10
DLX6-AS1	hsa-mir-144	HOXA10
MAGI2-AS3	hsa-mir-144	HOXA10
GAS5	hsa-mir-144	HOXA10
MLIP-AS1	hsa-mir-144	HOXA10
KLHL6-AS1	hsa-mir-144	HOXA10
CRNDE	hsa-mir-144	HOXA10
NRG1-IT1	hsa-mir-144	HOXA10
GRM5-AS1	hsa-mir-144	HOXA10
SNHG1	hsa-mir-144	HOXA10
LINC00261	hsa-mir-144	HOXA10
C15orf56	hsa-mir-144	PANK1
AC087392.1	hsa-mir-144	PANK1

FAM201A	hsa-mir-144	PANK1
CLLU1	hsa-mir-144	PANK1
AC010336.2	hsa-mir-144	PANK1
AL137145.2	hsa-mir-144	PANK1
POU6F2-AS1	hsa-mir-144	PANK1
LINC00466	hsa-mir-144	PANK1
WARS2-IT1	hsa-mir-144	PANK1
LINC00348	hsa-mir-144	PANK1
LINC00443	hsa-mir-144	PANK1
AL161645.1	hsa-mir-144	PANK1
DLX6-AS1	hsa-mir-144	PANK1
MAGI2-AS3	hsa-mir-144	PANK1
GAS5	hsa-mir-144	PANK1
MLIP-AS1	hsa-mir-144	PANK1
KLHL6-AS1	hsa-mir-144	PANK1
CRNDE	hsa-mir-144	PANK1
NRG1-IT1	hsa-mir-144	PANK1
GRM5-AS1	hsa-mir-144	PANK1
SNHG1	hsa-mir-144	PANK1
LINC00261	hsa-mir-144	PANK1
AL591845.1	hsa-mir-182	NPTX1
KIAA0087	hsa-mir-182	NPTX1
AC005280.1	hsa-mir-182	NPTX1
AC127496.1	hsa-mir-182	NPTX1
C15orf56	hsa-mir-182	NPTX1
FAM201A	hsa-mir-182	NPTX1
AC108134.1	hsa-mir-182	NPTX1
UCA1	hsa-mir-182	NPTX1
AC010336.2	hsa-mir-182	NPTX1
AL137145.2	hsa-mir-182	NPTX1
AC090150.1	hsa-mir-182	NPTX1
LINC00337	hsa-mir-182	NPTX1
SFTA1P	hsa-mir-182	NPTX1
BOLA3-AS1	hsa-mir-182	NPTX1
LINC00323	hsa-mir-182	NPTX1
LRRC3-AS1	hsa-mir-182	NPTX1
UBE2Q1-AS1	hsa-mir-182	NPTX1
F10-AS1	hsa-mir-182	NPTX1
AC110491.1	hsa-mir-182	NPTX1
TPRG1-AS1	hsa-mir-182	NPTX1
AC092171.1	hsa-mir-182	NPTX1
GAS5	hsa-mir-182	NPTX1
LINC00402	hsa-mir-182	NPTX1
LINC00494	hsa-mir-182	NPTX1
SYNPR-AS1	hsa-mir-182	NPTX1
AC073352.1	hsa-mir-182	NPTX1
AL139147.1	hsa-mir-182	NPTX1

GRM5-AS1	hsa-mir-182	NPTX1
SNHG1	hsa-mir-182	NPTX1
RERG-IT1	hsa-mir-182	NPTX1
LINC00261	hsa-mir-182	NPTX1
AL591845.1	hsa-mir-182	RARG
KIAA0087	hsa-mir-182	RARG
AC005280.1	hsa-mir-182	RARG
AC127496.1	hsa-mir-182	RARG
C15orf56	hsa-mir-182	RARG
FAM201A	hsa-mir-182	RARG
AC108134.1	hsa-mir-182	RARG
UCA1	hsa-mir-182	RARG
AC010336.2	hsa-mir-182	RARG
AL137145.2	hsa-mir-182	RARG
AC090150.1	hsa-mir-182	RARG
LINC00337	hsa-mir-182	RARG
SFTA1P	hsa-mir-182	RARG
BOLA3-AS1	hsa-mir-182	RARG
LINC00323	hsa-mir-182	RARG
LRRC3-AS1	hsa-mir-182	RARG
UBE2Q1-AS1	hsa-mir-182	RARG
F10-AS1	hsa-mir-182	RARG
AC110491.1	hsa-mir-182	RARG
TPRG1-AS1	hsa-mir-182	RARG
AC092171.1	hsa-mir-182	RARG
GAS5	hsa-mir-182	RARG
LINC00402	hsa-mir-182	RARG
LINC00494	hsa-mir-182	RARG
SYNPR-AS1	hsa-mir-182	RARG
AC073352.1	hsa-mir-182	RARG
AL139147.1	hsa-mir-182	RARG
GRM5-AS1	hsa-mir-182	RARG
SNHG1	hsa-mir-182	RARG
RERG-IT1	hsa-mir-182	RARG
LINC00261	hsa-mir-182	RARG
AL591845.1	hsa-mir-182	MITF
KIAA0087	hsa-mir-182	MITF
AC005280.1	hsa-mir-182	MITF
AC127496.1	hsa-mir-182	MITF
C15orf56	hsa-mir-182	MITF
FAM201A	hsa-mir-182	MITF
AC108134.1	hsa-mir-182	MITF
UCA1	hsa-mir-182	MITF
AC010336.2	hsa-mir-182	MITF
AL137145.2	hsa-mir-182	MITF
AC090150.1	hsa-mir-182	MITF
LINC00337	hsa-mir-182	MITF

SFTA1P	hsa-mir-182	MITF
BOLA3-AS1	hsa-mir-182	MITF
LINC00323	hsa-mir-182	MITF
LRRC3-AS1	hsa-mir-182	MITF
UBE2Q1-AS1	hsa-mir-182	MITF
F10-AS1	hsa-mir-182	MITF
AC110491.1	hsa-mir-182	MITF
TPRG1-AS1	hsa-mir-182	MITF
AC092171.1	hsa-mir-182	MITF
GAS5	hsa-mir-182	MITF
LINC00402	hsa-mir-182	MITF
LINC00494	hsa-mir-182	MITF
SYNPR-AS1	hsa-mir-182	MITF
AC073352.1	hsa-mir-182	MITF
AL139147.1	hsa-mir-182	MITF
GRM5-AS1	hsa-mir-182	MITF
SNHG1	hsa-mir-182	MITF
RERG-IT1	hsa-mir-182	MITF
LINC00261	hsa-mir-182	MITF
AL591845.1	hsa-mir-182	BDNF
KIAA0087	hsa-mir-182	BDNF
AC005280.1	hsa-mir-182	BDNF
AC127496.1	hsa-mir-182	BDNF
C15orf56	hsa-mir-182	BDNF
FAM201A	hsa-mir-182	BDNF
AC108134.1	hsa-mir-182	BDNF
UCA1	hsa-mir-182	BDNF
AC010336.2	hsa-mir-182	BDNF
AL137145.2	hsa-mir-182	BDNF
AC090150.1	hsa-mir-182	BDNF
LINC00337	hsa-mir-182	BDNF
SFTA1P	hsa-mir-182	BDNF
BOLA3-AS1	hsa-mir-182	BDNF
LINC00323	hsa-mir-182	BDNF
LRRC3-AS1	hsa-mir-182	BDNF
UBE2Q1-AS1	hsa-mir-182	BDNF
F10-AS1	hsa-mir-182	BDNF
AC110491.1	hsa-mir-182	BDNF
TPRG1-AS1	hsa-mir-182	BDNF
AC092171.1	hsa-mir-182	BDNF
GAS5	hsa-mir-182	BDNF
LINC00402	hsa-mir-182	BDNF
LINC00494	hsa-mir-182	BDNF
SYNPR-AS1	hsa-mir-182	BDNF
AC073352.1	hsa-mir-182	BDNF
AL139147.1	hsa-mir-182	BDNF
GRM5-AS1	hsa-mir-182	BDNF

SNHG1	hsa-mir-182	BDNF
RERG-IT1	hsa-mir-182	BDNF
LINC00261	hsa-mir-182	BDNF
AL591845.1	hsa-mir-182	FGF9
KIAA0087	hsa-mir-182	FGF9
AC005280.1	hsa-mir-182	FGF9
AC127496.1	hsa-mir-182	FGF9
C15orf56	hsa-mir-182	FGF9
FAM201A	hsa-mir-182	FGF9
AC108134.1	hsa-mir-182	FGF9
UCA1	hsa-mir-182	FGF9
AC010336.2	hsa-mir-182	FGF9
AL137145.2	hsa-mir-182	FGF9
AC090150.1	hsa-mir-182	FGF9
LINC00337	hsa-mir-182	FGF9
SFTA1P	hsa-mir-182	FGF9
BOLA3-AS1	hsa-mir-182	FGF9
LINC00323	hsa-mir-182	FGF9
LRRC3-AS1	hsa-mir-182	FGF9
UBE2Q1-AS1	hsa-mir-182	FGF9
F10-AS1	hsa-mir-182	FGF9
AC110491.1	hsa-mir-182	FGF9
TPRG1-AS1	hsa-mir-182	FGF9
AC092171.1	hsa-mir-182	FGF9
GAS5	hsa-mir-182	FGF9
LINC00402	hsa-mir-182	FGF9
LINC00494	hsa-mir-182	FGF9
SYNPR-AS1	hsa-mir-182	FGF9
AC073352.1	hsa-mir-182	FGF9
AL139147.1	hsa-mir-182	FGF9
GRM5-AS1	hsa-mir-182	FGF9
SNHG1	hsa-mir-182	FGF9
RERG-IT1	hsa-mir-182	FGF9
LINC00261	hsa-mir-182	FGF9
AL591845.1	hsa-mir-182	FOXF2
KIAA0087	hsa-mir-182	FOXF2
AC005280.1	hsa-mir-182	FOXF2
AC127496.1	hsa-mir-182	FOXF2
C15orf56	hsa-mir-182	FOXF2
FAM201A	hsa-mir-182	FOXF2
AC108134.1	hsa-mir-182	FOXF2
UCA1	hsa-mir-182	FOXF2
AC010336.2	hsa-mir-182	FOXF2
AL137145.2	hsa-mir-182	FOXF2
AC090150.1	hsa-mir-182	FOXF2
LINC00337	hsa-mir-182	FOXF2
SFTA1P	hsa-mir-182	FOXF2

BOLA3-AS1	hsa-mir-182	FOXF2
LINC00323	hsa-mir-182	FOXF2
LRRC3-AS1	hsa-mir-182	FOXF2
UBE2Q1-AS1	hsa-mir-182	FOXF2
F10-AS1	hsa-mir-182	FOXF2
AC110491.1	hsa-mir-182	FOXF2
TPRG1-AS1	hsa-mir-182	FOXF2
AC092171.1	hsa-mir-182	FOXF2
GAS5	hsa-mir-182	FOXF2
LINC00402	hsa-mir-182	FOXF2
LINC00494	hsa-mir-182	FOXF2
SYNPR-AS1	hsa-mir-182	FOXF2
AC073352.1	hsa-mir-182	FOXF2
AL139147.1	hsa-mir-182	FOXF2
GRM5-AS1	hsa-mir-182	FOXF2
SNHG1	hsa-mir-182	FOXF2
RERG-IT1	hsa-mir-182	FOXF2
LINC00261	hsa-mir-182	FOXF2
KIAA0087	hsa-mir-183	CCNB1
C2orf48	hsa-mir-183	CCNB1
AC127496.1	hsa-mir-183	CCNB1
C15orf56	hsa-mir-183	CCNB1
LINC00304	hsa-mir-183	CCNB1
LINC00315	hsa-mir-183	CCNB1
AC108134.1	hsa-mir-183	CCNB1
LINC00466	hsa-mir-183	CCNB1
FAM66C	hsa-mir-183	CCNB1
SZT2-AS1	hsa-mir-183	CCNB1
LINC00443	hsa-mir-183	CCNB1
CRNDE	hsa-mir-183	CCNB1
PVT1	hsa-mir-183	CCNB1
AL139385.1	hsa-mir-183	CCNB1
LINC00261	hsa-mir-183	CCNB1
KIAA0087	hsa-mir-200a	SCD5
H19	hsa-mir-200a	SCD5
FAM87A	hsa-mir-200a	SCD5
CLLU1	hsa-mir-200a	SCD5
AL359878.1	hsa-mir-200a	SCD5
AC092117.1	hsa-mir-200a	SCD5
AC010336.2	hsa-mir-200a	SCD5
LINC00466	hsa-mir-200a	SCD5
AL356356.1	hsa-mir-200a	SCD5
LINC00348	hsa-mir-200a	SCD5
LINC00242	hsa-mir-200a	SCD5
LINC00443	hsa-mir-200a	SCD5
DLX6-AS1	hsa-mir-200a	SCD5
AC110491.1	hsa-mir-200a	SCD5

MAGI2-AS3	hsa-mir-200a	SCD5
LINC00402	hsa-mir-200a	SCD5
AC009121.1	hsa-mir-200a	SCD5
TM4SF1-AS1	hsa-mir-200a	SCD5
HOTTIP	hsa-mir-200a	SCD5
NRG1-IT1	hsa-mir-200a	SCD5
FAM66D	hsa-mir-200a	SCD5
SNHG1	hsa-mir-200a	SCD5
FBXL19-AS1	hsa-mir-200a	SCD5
KIAA0087	hsa-mir-200a	SLC35D1
H19	hsa-mir-200a	SLC35D1
FAM87A	hsa-mir-200a	SLC35D1
CLLU1	hsa-mir-200a	SLC35D1
AL359878.1	hsa-mir-200a	SLC35D1
AC092117.1	hsa-mir-200a	SLC35D1
AC010336.2	hsa-mir-200a	SLC35D1
LINC00466	hsa-mir-200a	SLC35D1
AL356356.1	hsa-mir-200a	SLC35D1
LINC00348	hsa-mir-200a	SLC35D1
LINC00242	hsa-mir-200a	SLC35D1
LINC00443	hsa-mir-200a	SLC35D1
DLX6-AS1	hsa-mir-200a	SLC35D1
AC110491.1	hsa-mir-200a	SLC35D1
MAGI2-AS3	hsa-mir-200a	SLC35D1
LINC00402	hsa-mir-200a	SLC35D1
AC009121.1	hsa-mir-200a	SLC35D1
TM4SF1-AS1	hsa-mir-200a	SLC35D1
HOTTIP	hsa-mir-200a	SLC35D1
NRG1-IT1	hsa-mir-200a	SLC35D1
FAM66D	hsa-mir-200a	SLC35D1
SNHG1	hsa-mir-200a	SLC35D1
FBXL19-AS1	hsa-mir-200a	SLC35D1
KIAA0087	hsa-mir-200a	HOXB5
H19	hsa-mir-200a	HOXB5
FAM87A	hsa-mir-200a	HOXB5
CLLU1	hsa-mir-200a	HOXB5
AL359878.1	hsa-mir-200a	HOXB5
AC092117.1	hsa-mir-200a	HOXB5
AC010336.2	hsa-mir-200a	HOXB5
LINC00466	hsa-mir-200a	HOXB5
AL356356.1	hsa-mir-200a	HOXB5
LINC00348	hsa-mir-200a	HOXB5
LINC00242	hsa-mir-200a	HOXB5
LINC00443	hsa-mir-200a	HOXB5
DLX6-AS1	hsa-mir-200a	HOXB5
AC110491.1	hsa-mir-200a	HOXB5
MAGI2-AS3	hsa-mir-200a	HOXB5

LINC00402	hsa-mir-200a	HOXB5
AC009121.1	hsa-mir-200a	HOXB5
TM4SF1-AS1	hsa-mir-200a	HOXB5
HOTTIP	hsa-mir-200a	HOXB5
NRG1-IT1	hsa-mir-200a	HOXB5
FAM66D	hsa-mir-200a	HOXB5
SNHG1	hsa-mir-200a	HOXB5
FBXL19-AS1	hsa-mir-200a	HOXB5
KIAA0087	hsa-mir-200a	MACC1
H19	hsa-mir-200a	MACC1
FAM87A	hsa-mir-200a	MACC1
CLLU1	hsa-mir-200a	MACC1
AL359878.1	hsa-mir-200a	MACC1
AC092117.1	hsa-mir-200a	MACC1
AC010336.2	hsa-mir-200a	MACC1
LINC00466	hsa-mir-200a	MACC1
AL356356.1	hsa-mir-200a	MACC1
LINC00348	hsa-mir-200a	MACC1
LINC00242	hsa-mir-200a	MACC1
LINC00443	hsa-mir-200a	MACC1
DLX6-AS1	hsa-mir-200a	MACC1
AC110491.1	hsa-mir-200a	MACC1
MAGI2-AS3	hsa-mir-200a	MACC1
LINC00402	hsa-mir-200a	MACC1
AC009121.1	hsa-mir-200a	MACC1
TM4SF1-AS1	hsa-mir-200a	MACC1
HOTTIP	hsa-mir-200a	MACC1
NRG1-IT1	hsa-mir-200a	MACC1
FAM66D	hsa-mir-200a	MACC1
SNHG1	hsa-mir-200a	MACC1
FBXL19-AS1	hsa-mir-200a	MACC1
KIAA0087	hsa-mir-200a	EPHA7
H19	hsa-mir-200a	EPHA7
FAM87A	hsa-mir-200a	EPHA7
CLLU1	hsa-mir-200a	EPHA7
AL359878.1	hsa-mir-200a	EPHA7
AC092117.1	hsa-mir-200a	EPHA7
AC010336.2	hsa-mir-200a	EPHA7
LINC00466	hsa-mir-200a	EPHA7
AL356356.1	hsa-mir-200a	EPHA7
LINC00348	hsa-mir-200a	EPHA7
LINC00242	hsa-mir-200a	EPHA7
LINC00443	hsa-mir-200a	EPHA7
DLX6-AS1	hsa-mir-200a	EPHA7
AC110491.1	hsa-mir-200a	EPHA7
MAGI2-AS3	hsa-mir-200a	EPHA7
LINC00402	hsa-mir-200a	EPHA7

AC009121.1	hsa-mir-200a	EPHA7
TM4SF1-AS1	hsa-mir-200a	EPHA7
HOTTIP	hsa-mir-200a	EPHA7
NRG1-IT1	hsa-mir-200a	EPHA7
FAM66D	hsa-mir-200a	EPHA7
SNHG1	hsa-mir-200a	EPHA7
FBXL19-AS1	hsa-mir-200a	EPHA7
KIAA0087	hsa-mir-200a	UBASH3B
H19	hsa-mir-200a	UBASH3B
FAM87A	hsa-mir-200a	UBASH3B
CLLU1	hsa-mir-200a	UBASH3B
AL359878.1	hsa-mir-200a	UBASH3B
AC092117.1	hsa-mir-200a	UBASH3B
AC010336.2	hsa-mir-200a	UBASH3B
LINC00466	hsa-mir-200a	UBASH3B
AL356356.1	hsa-mir-200a	UBASH3B
LINC00348	hsa-mir-200a	UBASH3B
LINC00242	hsa-mir-200a	UBASH3B
LINC00443	hsa-mir-200a	UBASH3B
DLX6-AS1	hsa-mir-200a	UBASH3B
AC110491.1	hsa-mir-200a	UBASH3B
MAGI2-AS3	hsa-mir-200a	UBASH3B
LINC00402	hsa-mir-200a	UBASH3B
AC009121.1	hsa-mir-200a	UBASH3B
TM4SF1-AS1	hsa-mir-200a	UBASH3B
HOTTIP	hsa-mir-200a	UBASH3B
NRG1-IT1	hsa-mir-200a	UBASH3B
FAM66D	hsa-mir-200a	UBASH3B
SNHG1	hsa-mir-200a	UBASH3B
FBXL19-AS1	hsa-mir-200a	UBASH3B
C9orf163	hsa-mir-21	FAM46A
LINC00336	hsa-mir-21	FAM46A
FAM201A	hsa-mir-21	FAM46A
CLLU1	hsa-mir-21	FAM46A
LINC00466	hsa-mir-21	FAM46A
FAM66C	hsa-mir-21	FAM46A
HOTAIR	hsa-mir-21	FAM46A
HS1BP3-IT1	hsa-mir-21	FAM46A
GAS5	hsa-mir-21	FAM46A
PVT1	hsa-mir-21	FAM46A
GRM5-AS1	hsa-mir-21	FAM46A
SNHG1	hsa-mir-21	FAM46A
RERG-IT1	hsa-mir-21	FAM46A
C9orf163	hsa-mir-21	JPH1
LINC00336	hsa-mir-21	JPH1
FAM201A	hsa-mir-21	JPH1
CLLU1	hsa-mir-21	JPH1

LINC00466	hsa-mir-21	JPH1
FAM66C	hsa-mir-21	JPH1
HOTAIR	hsa-mir-21	JPH1
HS1BP3-IT1	hsa-mir-21	JPH1
GAS5	hsa-mir-21	JPH1
PVT1	hsa-mir-21	JPH1
GRM5-AS1	hsa-mir-21	JPH1
SNHG1	hsa-mir-21	JPH1
RERG-IT1	hsa-mir-21	JPH1
C9orf163	hsa-mir-21	ELOVL7
LINC00336	hsa-mir-21	ELOVL7
FAM201A	hsa-mir-21	ELOVL7
CLLU1	hsa-mir-21	ELOVL7
LINC00466	hsa-mir-21	ELOVL7
FAM66C	hsa-mir-21	ELOVL7
HOTAIR	hsa-mir-21	ELOVL7
HS1BP3-IT1	hsa-mir-21	ELOVL7
GAS5	hsa-mir-21	ELOVL7
PVT1	hsa-mir-21	ELOVL7
GRM5-AS1	hsa-mir-21	ELOVL7
SNHG1	hsa-mir-21	ELOVL7
RERG-IT1	hsa-mir-21	ELOVL7
C9orf163	hsa-mir-21	CPEB3
LINC00336	hsa-mir-21	CPEB3
FAM201A	hsa-mir-21	CPEB3
CLLU1	hsa-mir-21	CPEB3
LINC00466	hsa-mir-21	CPEB3
FAM66C	hsa-mir-21	CPEB3
HOTAIR	hsa-mir-21	CPEB3
HS1BP3-IT1	hsa-mir-21	CPEB3
GAS5	hsa-mir-21	CPEB3
PVT1	hsa-mir-21	CPEB3
GRM5-AS1	hsa-mir-21	CPEB3
SNHG1	hsa-mir-21	CPEB3
RERG-IT1	hsa-mir-21	CPEB3
C9orf163	hsa-mir-21	SCRN1
LINC00336	hsa-mir-21	SCRN1
FAM201A	hsa-mir-21	SCRN1
CLLU1	hsa-mir-21	SCRN1
LINC00466	hsa-mir-21	SCRN1
FAM66C	hsa-mir-21	SCRN1
HOTAIR	hsa-mir-21	SCRN1
HS1BP3-IT1	hsa-mir-21	SCRN1
GAS5	hsa-mir-21	SCRN1
PVT1	hsa-mir-21	SCRN1
GRM5-AS1	hsa-mir-21	SCRN1
SNHG1	hsa-mir-21	SCRN1

RERG-IT1	hsa-mir-21	SCRN1
C9orf163	hsa-mir-21	PTPN14
LINC00336	hsa-mir-21	PTPN14
FAM201A	hsa-mir-21	PTPN14
CLLU1	hsa-mir-21	PTPN14
LINC00466	hsa-mir-21	PTPN14
FAM66C	hsa-mir-21	PTPN14
HOTAIR	hsa-mir-21	PTPN14
HS1BP3-IT1	hsa-mir-21	PTPN14
GAS5	hsa-mir-21	PTPN14
PVT1	hsa-mir-21	PTPN14
GRM5-AS1	hsa-mir-21	PTPN14
SNHG1	hsa-mir-21	PTPN14
RERG-IT1	hsa-mir-21	PTPN14
C9orf163	hsa-mir-21	NTF3
LINC00336	hsa-mir-21	NTF3
FAM201A	hsa-mir-21	NTF3
CLLU1	hsa-mir-21	NTF3
LINC00466	hsa-mir-21	NTF3
FAM66C	hsa-mir-21	NTF3
HOTAIR	hsa-mir-21	NTF3
HS1BP3-IT1	hsa-mir-21	NTF3
GAS5	hsa-mir-21	NTF3
PVT1	hsa-mir-21	NTF3
GRM5-AS1	hsa-mir-21	NTF3
SNHG1	hsa-mir-21	NTF3
RERG-IT1	hsa-mir-21	NTF3
C9orf163	hsa-mir-21	JAG1
LINC00336	hsa-mir-21	JAG1
FAM201A	hsa-mir-21	JAG1
CLLU1	hsa-mir-21	JAG1
LINC00466	hsa-mir-21	JAG1
FAM66C	hsa-mir-21	JAG1
HOTAIR	hsa-mir-21	JAG1
HS1BP3-IT1	hsa-mir-21	JAG1
GAS5	hsa-mir-21	JAG1
PVT1	hsa-mir-21	JAG1
GRM5-AS1	hsa-mir-21	JAG1
SNHG1	hsa-mir-21	JAG1
RERG-IT1	hsa-mir-21	JAG1
C9orf163	hsa-mir-21	PIK3R1
LINC00336	hsa-mir-21	PIK3R1
FAM201A	hsa-mir-21	PIK3R1
CLLU1	hsa-mir-21	PIK3R1
LINC00466	hsa-mir-21	PIK3R1
FAM66C	hsa-mir-21	PIK3R1
HOTAIR	hsa-mir-21	PIK3R1

HS1BP3-IT1	hsa-mir-21	PIK3R1
GAS5	hsa-mir-21	PIK3R1
PVT1	hsa-mir-21	PIK3R1
GRM5-AS1	hsa-mir-21	PIK3R1
SNHG1	hsa-mir-21	PIK3R1
RERG-IT1	hsa-mir-21	PIK3R1
C9orf163	hsa-mir-21	EPM2A
LINC00336	hsa-mir-21	EPM2A
FAM201A	hsa-mir-21	EPM2A
CLLU1	hsa-mir-21	EPM2A
LINC00466	hsa-mir-21	EPM2A
FAM66C	hsa-mir-21	EPM2A
HOTAIR	hsa-mir-21	EPM2A
HS1BP3-IT1	hsa-mir-21	EPM2A
GAS5	hsa-mir-21	EPM2A
PVT1	hsa-mir-21	EPM2A
GRM5-AS1	hsa-mir-21	EPM2A
SNHG1	hsa-mir-21	EPM2A
RERG-IT1	hsa-mir-21	EPM2A
KIAA0087	hsa-mir-211	SLC43A1
C2orf48	hsa-mir-211	SLC43A1
AC127496.1	hsa-mir-211	SLC43A1
C10orf91	hsa-mir-211	SLC43A1
LINC00313	hsa-mir-211	SLC43A1
PSORS1C3	hsa-mir-211	SLC43A1
CLLU1	hsa-mir-211	SLC43A1
AC092117.1	hsa-mir-211	SLC43A1
AL137145.1	hsa-mir-211	SLC43A1
AL021068.1	hsa-mir-211	SLC43A1
AC010336.2	hsa-mir-211	SLC43A1
AC011481.1	hsa-mir-211	SLC43A1
LINC00466	hsa-mir-211	SLC43A1
THRB-IT1	hsa-mir-211	SLC43A1
SFTA1P	hsa-mir-211	SLC43A1
BOLA3-AS1	hsa-mir-211	SLC43A1
DBH-AS1	hsa-mir-211	SLC43A1
AL356356.1	hsa-mir-211	SLC43A1
LINC00323	hsa-mir-211	SLC43A1
FAM66C	hsa-mir-211	SLC43A1
HOTAIR	hsa-mir-211	SLC43A1
LINC00242	hsa-mir-211	SLC43A1
LRRC3-AS1	hsa-mir-211	SLC43A1
UBE2Q1-AS1	hsa-mir-211	SLC43A1
LINC00443	hsa-mir-211	SLC43A1
AL161645.1	hsa-mir-211	SLC43A1
DLX6-AS1	hsa-mir-211	SLC43A1
AC110491.1	hsa-mir-211	SLC43A1

MAGI2-AS3	hsa-mir-211	SLC43A1
LINC00402	hsa-mir-211	SLC43A1
SAPCD1-AS1	hsa-mir-211	SLC43A1
NEXN-AS1	hsa-mir-211	SLC43A1
AC015987.1	hsa-mir-211	SLC43A1
AL137798.1	hsa-mir-211	SLC43A1
CLRN1-AS1	hsa-mir-211	SLC43A1
ITIH4-AS1	hsa-mir-211	SLC43A1
HOTTIP	hsa-mir-211	SLC43A1
AP001781.1	hsa-mir-211	SLC43A1
SNHG1	hsa-mir-211	SLC43A1
LINC00261	hsa-mir-211	SLC43A1
KIAA0087	hsa-mir-211	TMTC2
C2orf48	hsa-mir-211	TMTC2
AC127496.1	hsa-mir-211	TMTC2
C10orf91	hsa-mir-211	TMTC2
LINC00313	hsa-mir-211	TMTC2
PSORS1C3	hsa-mir-211	TMTC2
CLLU1	hsa-mir-211	TMTC2
AC092117.1	hsa-mir-211	TMTC2
AL137145.1	hsa-mir-211	TMTC2
AL021068.1	hsa-mir-211	TMTC2
AC010336.2	hsa-mir-211	TMTC2
AC011481.1	hsa-mir-211	TMTC2
LINC00466	hsa-mir-211	TMTC2
THRB-IT1	hsa-mir-211	TMTC2
SFTA1P	hsa-mir-211	TMTC2
BOLA3-AS1	hsa-mir-211	TMTC2
DBH-AS1	hsa-mir-211	TMTC2
AL356356.1	hsa-mir-211	TMTC2
LINC00323	hsa-mir-211	TMTC2
FAM66C	hsa-mir-211	TMTC2
HOTAIR	hsa-mir-211	TMTC2
LINC00242	hsa-mir-211	TMTC2
LRRC3-AS1	hsa-mir-211	TMTC2
UBE2Q1-AS1	hsa-mir-211	TMTC2
LINC00443	hsa-mir-211	TMTC2
AL161645.1	hsa-mir-211	TMTC2
DLX6-AS1	hsa-mir-211	TMTC2
AC110491.1	hsa-mir-211	TMTC2
MAGI2-AS3	hsa-mir-211	TMTC2
LINC00402	hsa-mir-211	TMTC2
SAPCD1-AS1	hsa-mir-211	TMTC2
NEXN-AS1	hsa-mir-211	TMTC2
AC015987.1	hsa-mir-211	TMTC2
AL137798.1	hsa-mir-211	TMTC2
CLRN1-AS1	hsa-mir-211	TMTC2

ITIH4-AS1	hsa-mir-211	TMTC2
HOTTIP	hsa-mir-211	TMTC2
AP001781.1	hsa-mir-211	TMTC2
SNHG1	hsa-mir-211	TMTC2
LINC00261	hsa-mir-211	TMTC2
KIAA0087	hsa-mir-211	SAMD5
C2orf48	hsa-mir-211	SAMD5
AC127496.1	hsa-mir-211	SAMD5
C10orf91	hsa-mir-211	SAMD5
LINC00313	hsa-mir-211	SAMD5
PSORS1C3	hsa-mir-211	SAMD5
CLLU1	hsa-mir-211	SAMD5
AC092117.1	hsa-mir-211	SAMD5
AL137145.1	hsa-mir-211	SAMD5
AL021068.1	hsa-mir-211	SAMD5
AC010336.2	hsa-mir-211	SAMD5
AC011481.1	hsa-mir-211	SAMD5
LINC00466	hsa-mir-211	SAMD5
THRB-IT1	hsa-mir-211	SAMD5
SFTA1P	hsa-mir-211	SAMD5
BOLA3-AS1	hsa-mir-211	SAMD5
DBH-AS1	hsa-mir-211	SAMD5
AL356356.1	hsa-mir-211	SAMD5
LINC00323	hsa-mir-211	SAMD5
FAM66C	hsa-mir-211	SAMD5
HOTAIR	hsa-mir-211	SAMD5
LINC00242	hsa-mir-211	SAMD5
LRRC3-AS1	hsa-mir-211	SAMD5
UBE2Q1-AS1	hsa-mir-211	SAMD5
LINC00443	hsa-mir-211	SAMD5
AL161645.1	hsa-mir-211	SAMD5
DLX6-AS1	hsa-mir-211	SAMD5
AC110491.1	hsa-mir-211	SAMD5
MAGI2-AS3	hsa-mir-211	SAMD5
LINC00402	hsa-mir-211	SAMD5
SAPCD1-AS1	hsa-mir-211	SAMD5
NEXN-AS1	hsa-mir-211	SAMD5
AC015987.1	hsa-mir-211	SAMD5
AL137798.1	hsa-mir-211	SAMD5
CLRN1-AS1	hsa-mir-211	SAMD5
ITIH4-AS1	hsa-mir-211	SAMD5
HOTTIP	hsa-mir-211	SAMD5
AP001781.1	hsa-mir-211	SAMD5
SNHG1	hsa-mir-211	SAMD5
LINC00261	hsa-mir-211	SAMD5
KIAA0087	hsa-mir-211	ZCCHC24
C2orf48	hsa-mir-211	ZCCHC24

AC127496.1	hsa-mir-211	ZCCHC24
C10orf91	hsa-mir-211	ZCCHC24
LINC00313	hsa-mir-211	ZCCHC24
PSORS1C3	hsa-mir-211	ZCCHC24
CLLU1	hsa-mir-211	ZCCHC24
AC092117.1	hsa-mir-211	ZCCHC24
AL137145.1	hsa-mir-211	ZCCHC24
AL021068.1	hsa-mir-211	ZCCHC24
AC010336.2	hsa-mir-211	ZCCHC24
AC011481.1	hsa-mir-211	ZCCHC24
LINC00466	hsa-mir-211	ZCCHC24
THRB-IT1	hsa-mir-211	ZCCHC24
SFTA1P	hsa-mir-211	ZCCHC24
BOLA3-AS1	hsa-mir-211	ZCCHC24
DBH-AS1	hsa-mir-211	ZCCHC24
AL356356.1	hsa-mir-211	ZCCHC24
LINC00323	hsa-mir-211	ZCCHC24
FAM66C	hsa-mir-211	ZCCHC24
HOTAIR	hsa-mir-211	ZCCHC24
LINC00242	hsa-mir-211	ZCCHC24
LRRC3-AS1	hsa-mir-211	ZCCHC24
UBE2Q1-AS1	hsa-mir-211	ZCCHC24
LINC00443	hsa-mir-211	ZCCHC24
AL161645.1	hsa-mir-211	ZCCHC24
DLX6-AS1	hsa-mir-211	ZCCHC24
AC110491.1	hsa-mir-211	ZCCHC24
MAGI2-AS3	hsa-mir-211	ZCCHC24
LINC00402	hsa-mir-211	ZCCHC24
SAPCD1-AS1	hsa-mir-211	ZCCHC24
NEXN-AS1	hsa-mir-211	ZCCHC24
AC015987.1	hsa-mir-211	ZCCHC24
AL137798.1	hsa-mir-211	ZCCHC24
CLRN1-AS1	hsa-mir-211	ZCCHC24
ITIH4-AS1	hsa-mir-211	ZCCHC24
HOTTIP	hsa-mir-211	ZCCHC24
AP001781.1	hsa-mir-211	ZCCHC24
SNHG1	hsa-mir-211	ZCCHC24
LINC00261	hsa-mir-211	ZCCHC24
KIAA0087	hsa-mir-211	ELOVL6
C2orf48	hsa-mir-211	ELOVL6
AC127496.1	hsa-mir-211	ELOVL6
C10orf91	hsa-mir-211	ELOVL6
LINC00313	hsa-mir-211	ELOVL6
PSORS1C3	hsa-mir-211	ELOVL6
CLLU1	hsa-mir-211	ELOVL6
AC092117.1	hsa-mir-211	ELOVL6
AL137145.1	hsa-mir-211	ELOVL6

AL021068.1	hsa-mir-211	ELOVL6
AC010336.2	hsa-mir-211	ELOVL6
AC011481.1	hsa-mir-211	ELOVL6
LINC00466	hsa-mir-211	ELOVL6
THRB-IT1	hsa-mir-211	ELOVL6
SFTA1P	hsa-mir-211	ELOVL6
BOLA3-AS1	hsa-mir-211	ELOVL6
DBH-AS1	hsa-mir-211	ELOVL6
AL356356.1	hsa-mir-211	ELOVL6
LINC00323	hsa-mir-211	ELOVL6
FAM66C	hsa-mir-211	ELOVL6
HOTAIR	hsa-mir-211	ELOVL6
LINC00242	hsa-mir-211	ELOVL6
LRRC3-AS1	hsa-mir-211	ELOVL6
UBE2Q1-AS1	hsa-mir-211	ELOVL6
LINC00443	hsa-mir-211	ELOVL6
AL161645.1	hsa-mir-211	ELOVL6
DLX6-AS1	hsa-mir-211	ELOVL6
AC110491.1	hsa-mir-211	ELOVL6
MAGI2-AS3	hsa-mir-211	ELOVL6
LINC00402	hsa-mir-211	ELOVL6
SAPCD1-AS1	hsa-mir-211	ELOVL6
NEXN-AS1	hsa-mir-211	ELOVL6
AC015987.1	hsa-mir-211	ELOVL6
AL137798.1	hsa-mir-211	ELOVL6
CLRN1-AS1	hsa-mir-211	ELOVL6
ITIH4-AS1	hsa-mir-211	ELOVL6
HOTTIP	hsa-mir-211	ELOVL6
AP001781.1	hsa-mir-211	ELOVL6
SNHG1	hsa-mir-211	ELOVL6
LINC00261	hsa-mir-211	ELOVL6
KIAA0087	hsa-mir-211	IL11
C2orf48	hsa-mir-211	IL11
AC127496.1	hsa-mir-211	IL11
C10orf91	hsa-mir-211	IL11
LINC00313	hsa-mir-211	IL11
PSORS1C3	hsa-mir-211	IL11
CLLU1	hsa-mir-211	IL11
AC092117.1	hsa-mir-211	IL11
AL137145.1	hsa-mir-211	IL11
AL021068.1	hsa-mir-211	IL11
AC010336.2	hsa-mir-211	IL11
AC011481.1	hsa-mir-211	IL11
LINC00466	hsa-mir-211	IL11
THRB-IT1	hsa-mir-211	IL11
SFTA1P	hsa-mir-211	IL11
BOLA3-AS1	hsa-mir-211	IL11

DBH-AS1	hsa-mir-211	IL11
AL356356.1	hsa-mir-211	IL11
LINC00323	hsa-mir-211	IL11
FAM66C	hsa-mir-211	IL11
HOTAIR	hsa-mir-211	IL11
LINC00242	hsa-mir-211	IL11
LRRC3-AS1	hsa-mir-211	IL11
UBE2Q1-AS1	hsa-mir-211	IL11
LINC00443	hsa-mir-211	IL11
AL161645.1	hsa-mir-211	IL11
DLX6-AS1	hsa-mir-211	IL11
AC110491.1	hsa-mir-211	IL11
MAGI2-AS3	hsa-mir-211	IL11
LINC00402	hsa-mir-211	IL11
SAPCD1-AS1	hsa-mir-211	IL11
NEXN-AS1	hsa-mir-211	IL11
AC015987.1	hsa-mir-211	IL11
AL137798.1	hsa-mir-211	IL11
CLRN1-AS1	hsa-mir-211	IL11
ITIH4-AS1	hsa-mir-211	IL11
HOTTIP	hsa-mir-211	IL11
AP001781.1	hsa-mir-211	IL11
SNHG1	hsa-mir-211	IL11
LINC00261	hsa-mir-211	IL11
KIAA0087	hsa-mir-211	NPTX1
C2orf48	hsa-mir-211	NPTX1
AC127496.1	hsa-mir-211	NPTX1
C10orf91	hsa-mir-211	NPTX1
LINC00313	hsa-mir-211	NPTX1
PSORS1C3	hsa-mir-211	NPTX1
CLLU1	hsa-mir-211	NPTX1
AC092117.1	hsa-mir-211	NPTX1
AL137145.1	hsa-mir-211	NPTX1
AL021068.1	hsa-mir-211	NPTX1
AC010336.2	hsa-mir-211	NPTX1
AC011481.1	hsa-mir-211	NPTX1
LINC00466	hsa-mir-211	NPTX1
THRB-IT1	hsa-mir-211	NPTX1
SFTA1P	hsa-mir-211	NPTX1
BOLA3-AS1	hsa-mir-211	NPTX1
DBH-AS1	hsa-mir-211	NPTX1
AL356356.1	hsa-mir-211	NPTX1
LINC00323	hsa-mir-211	NPTX1
FAM66C	hsa-mir-211	NPTX1
HOTAIR	hsa-mir-211	NPTX1
LINC00242	hsa-mir-211	NPTX1
LRRC3-AS1	hsa-mir-211	NPTX1

UBE2Q1-AS1	hsa-mir-211	NPTX1
LINC00443	hsa-mir-211	NPTX1
AL161645.1	hsa-mir-211	NPTX1
DLX6-AS1	hsa-mir-211	NPTX1
AC110491.1	hsa-mir-211	NPTX1
MAGI2-AS3	hsa-mir-211	NPTX1
LINC00402	hsa-mir-211	NPTX1
SAPCD1-AS1	hsa-mir-211	NPTX1
NEXN-AS1	hsa-mir-211	NPTX1
AC015987.1	hsa-mir-211	NPTX1
AL137798.1	hsa-mir-211	NPTX1
CLRN1-AS1	hsa-mir-211	NPTX1
ITIH4-AS1	hsa-mir-211	NPTX1
HOTTIP	hsa-mir-211	NPTX1
AP001781.1	hsa-mir-211	NPTX1
SNHG1	hsa-mir-211	NPTX1
LINC00261	hsa-mir-211	NPTX1
KIAA0087	hsa-mir-211	CREB5
C2orf48	hsa-mir-211	CREB5
AC127496.1	hsa-mir-211	CREB5
C10orf91	hsa-mir-211	CREB5
LINC00313	hsa-mir-211	CREB5
PSORS1C3	hsa-mir-211	CREB5
CLLU1	hsa-mir-211	CREB5
AC092117.1	hsa-mir-211	CREB5
AL137145.1	hsa-mir-211	CREB5
AL021068.1	hsa-mir-211	CREB5
AC010336.2	hsa-mir-211	CREB5
AC011481.1	hsa-mir-211	CREB5
LINC00466	hsa-mir-211	CREB5
THRB-IT1	hsa-mir-211	CREB5
SFTA1P	hsa-mir-211	CREB5
BOLA3-AS1	hsa-mir-211	CREB5
DBH-AS1	hsa-mir-211	CREB5
AL356356.1	hsa-mir-211	CREB5
LINC00323	hsa-mir-211	CREB5
FAM66C	hsa-mir-211	CREB5
HOTAIR	hsa-mir-211	CREB5
LINC00242	hsa-mir-211	CREB5
LRRC3-AS1	hsa-mir-211	CREB5
UBE2Q1-AS1	hsa-mir-211	CREB5
LINC00443	hsa-mir-211	CREB5
AL161645.1	hsa-mir-211	CREB5
DLX6-AS1	hsa-mir-211	CREB5
AC110491.1	hsa-mir-211	CREB5
MAGI2-AS3	hsa-mir-211	CREB5
LINC00402	hsa-mir-211	CREB5

SAPCD1-AS1	hsa-mir-211	CREB5
NEXN-AS1	hsa-mir-211	CREB5
AC015987.1	hsa-mir-211	CREB5
AL137798.1	hsa-mir-211	CREB5
CLRN1-AS1	hsa-mir-211	CREB5
ITIH4-AS1	hsa-mir-211	CREB5
HOTTIP	hsa-mir-211	CREB5
AP001781.1	hsa-mir-211	CREB5
SNHG1	hsa-mir-211	CREB5
LINC00261	hsa-mir-211	CREB5
KIAA0087	hsa-mir-211	HOXC8
C2orf48	hsa-mir-211	HOXC8
AC127496.1	hsa-mir-211	HOXC8
C10orf91	hsa-mir-211	HOXC8
LINC00313	hsa-mir-211	HOXC8
PSORS1C3	hsa-mir-211	HOXC8
CLLU1	hsa-mir-211	HOXC8
AC092117.1	hsa-mir-211	HOXC8
AL137145.1	hsa-mir-211	HOXC8
AL021068.1	hsa-mir-211	HOXC8
AC010336.2	hsa-mir-211	HOXC8
AC011481.1	hsa-mir-211	HOXC8
LINC00466	hsa-mir-211	HOXC8
THRB-IT1	hsa-mir-211	HOXC8
SFTA1P	hsa-mir-211	HOXC8
BOLA3-AS1	hsa-mir-211	HOXC8
DBH-AS1	hsa-mir-211	HOXC8
AL356356.1	hsa-mir-211	HOXC8
LINC00323	hsa-mir-211	HOXC8
FAM66C	hsa-mir-211	HOXC8
HOTAIR	hsa-mir-211	HOXC8
LINC00242	hsa-mir-211	HOXC8
LRRC3-AS1	hsa-mir-211	HOXC8
UBE2Q1-AS1	hsa-mir-211	HOXC8
LINC00443	hsa-mir-211	HOXC8
AL161645.1	hsa-mir-211	HOXC8
DLX6-AS1	hsa-mir-211	HOXC8
AC110491.1	hsa-mir-211	HOXC8
MAGI2-AS3	hsa-mir-211	HOXC8
LINC00402	hsa-mir-211	HOXC8
SAPCD1-AS1	hsa-mir-211	HOXC8
NEXN-AS1	hsa-mir-211	HOXC8
AC015987.1	hsa-mir-211	HOXC8
AL137798.1	hsa-mir-211	HOXC8
CLRN1-AS1	hsa-mir-211	HOXC8
ITIH4-AS1	hsa-mir-211	HOXC8
HOTTIP	hsa-mir-211	HOXC8

AP001781.1	hsa-mir-211	HOXC8
SNHG1	hsa-mir-211	HOXC8
LINC00261	hsa-mir-211	HOXC8
LINC00304	hsa-mir-222	FOS
FAM87A	hsa-mir-222	FOS
AC087392.1	hsa-mir-222	FOS
FAM201A	hsa-mir-222	FOS
CLLU1	hsa-mir-222	FOS
AL137145.1	hsa-mir-222	FOS
CLDN10-AS1	hsa-mir-222	FOS
SFTA1P	hsa-mir-222	FOS
FAM66C	hsa-mir-222	FOS
HOTAIR	hsa-mir-222	FOS
LINC00242	hsa-mir-222	FOS
DLG3-AS1	hsa-mir-222	FOS
AC037487.1	hsa-mir-222	FOS
AC110491.1	hsa-mir-222	FOS
LINC00460	hsa-mir-222	FOS
GAS5	hsa-mir-222	FOS
MLIP-AS1	hsa-mir-222	FOS
LINC00494	hsa-mir-222	FOS
CLRN1-AS1	hsa-mir-222	FOS
CRNDE	hsa-mir-222	FOS
PVT1	hsa-mir-222	FOS
AC147651.1	hsa-mir-222	FOS
FAM66D	hsa-mir-222	FOS
AP006285.1	hsa-mir-222	FOS
LINC00304	hsa-mir-222	GALNT3
FAM87A	hsa-mir-222	GALNT3
AC087392.1	hsa-mir-222	GALNT3
FAM201A	hsa-mir-222	GALNT3
CLLU1	hsa-mir-222	GALNT3
AL137145.1	hsa-mir-222	GALNT3
CLDN10-AS1	hsa-mir-222	GALNT3
SFTA1P	hsa-mir-222	GALNT3
FAM66C	hsa-mir-222	GALNT3
HOTAIR	hsa-mir-222	GALNT3
LINC00242	hsa-mir-222	GALNT3
DLG3-AS1	hsa-mir-222	GALNT3
AC037487.1	hsa-mir-222	GALNT3
AC110491.1	hsa-mir-222	GALNT3
LINC00460	hsa-mir-222	GALNT3
GAS5	hsa-mir-222	GALNT3
MLIP-AS1	hsa-mir-222	GALNT3
LINC00494	hsa-mir-222	GALNT3
CLRN1-AS1	hsa-mir-222	GALNT3
CRNDE	hsa-mir-222	GALNT3

PVT1	hsa-mir-222	GALNT3
AC147651.1	hsa-mir-222	GALNT3
FAM66D	hsa-mir-222	GALNT3
AP006285.1	hsa-mir-222	GALNT3
FAM87A	hsa-mir-31	HOXC13
LINC00313	hsa-mir-31	HOXC13
AC116351.1	hsa-mir-31	HOXC13
CLLU1	hsa-mir-31	HOXC13
AP000525.1	hsa-mir-31	HOXC13
AL137145.1	hsa-mir-31	HOXC13
AC010336.2	hsa-mir-31	HOXC13
AL137145.2	hsa-mir-31	HOXC13
AC016773.1	hsa-mir-31	HOXC13
LINC00184	hsa-mir-31	HOXC13
LINC00242	hsa-mir-31	HOXC13
HM13-AS1	hsa-mir-31	HOXC13
DLX6-AS1	hsa-mir-31	HOXC13
FAM215B	hsa-mir-31	HOXC13
MAGI2-AS3	hsa-mir-31	HOXC13
GAS5	hsa-mir-31	HOXC13
LINC00494	hsa-mir-31	HOXC13
HOTTIP	hsa-mir-31	HOXC13
CRNDE	hsa-mir-31	HOXC13
PVT1	hsa-mir-31	HOXC13
GRM5-AS1	hsa-mir-31	HOXC13
AP001029.2	hsa-mir-31	HOXC13
LINC00261	hsa-mir-31	HOXC13
FAM87A	hsa-mir-31	FOXD4
LINC00313	hsa-mir-31	FOXD4
AC116351.1	hsa-mir-31	FOXD4
CLLU1	hsa-mir-31	FOXD4
AP000525.1	hsa-mir-31	FOXD4
AL137145.1	hsa-mir-31	FOXD4
AC010336.2	hsa-mir-31	FOXD4
AL137145.2	hsa-mir-31	FOXD4
AC016773.1	hsa-mir-31	FOXD4
LINC00184	hsa-mir-31	FOXD4
LINC00242	hsa-mir-31	FOXD4
HM13-AS1	hsa-mir-31	FOXD4
DLX6-AS1	hsa-mir-31	FOXD4
FAM215B	hsa-mir-31	FOXD4
MAGI2-AS3	hsa-mir-31	FOXD4
GAS5	hsa-mir-31	FOXD4
LINC00494	hsa-mir-31	FOXD4
HOTTIP	hsa-mir-31	FOXD4
CRNDE	hsa-mir-31	FOXD4
PVT1	hsa-mir-31	FOXD4

GRM5-AS1	hsa-mir-31	FOXD4
AP001029.2	hsa-mir-31	FOXD4
LINC00261	hsa-mir-31	FOXD4
KIAA0087	hsa-mir-429	PMAIP1
C10orf91	hsa-mir-429	PMAIP1
AC087392.1	hsa-mir-429	PMAIP1
CLLU1	hsa-mir-429	PMAIP1
AL021068.1	hsa-mir-429	PMAIP1
AC010336.2	hsa-mir-429	PMAIP1
AL137145.2	hsa-mir-429	PMAIP1
AL354984.1	hsa-mir-429	PMAIP1
AC080037.1	hsa-mir-429	PMAIP1
LINC00466	hsa-mir-429	PMAIP1
THRB-IT1	hsa-mir-429	PMAIP1
BOLA3-AS1	hsa-mir-429	PMAIP1
FAM66C	hsa-mir-429	PMAIP1
DLX6-AS1	hsa-mir-429	PMAIP1
AC110491.1	hsa-mir-429	PMAIP1
LINC00460	hsa-mir-429	PMAIP1
MAGI2-AS3	hsa-mir-429	PMAIP1
GAS5	hsa-mir-429	PMAIP1
LINC00402	hsa-mir-429	PMAIP1
AC015987.1	hsa-mir-429	PMAIP1
CLRN1-AS1	hsa-mir-429	PMAIP1
GK-AS1	hsa-mir-429	PMAIP1
GRM5-AS1	hsa-mir-429	PMAIP1
LINC00261	hsa-mir-429	PMAIP1
AL591845.1	hsa-mir-96	TSKU
KIAA0087	hsa-mir-96	TSKU
AC005280.1	hsa-mir-96	TSKU
AC127496.1	hsa-mir-96	TSKU
FAM87A	hsa-mir-96	TSKU
LINC00336	hsa-mir-96	TSKU
FAM201A	hsa-mir-96	TSKU
UCA1	hsa-mir-96	TSKU
AC096642.1	hsa-mir-96	TSKU
LINC00466	hsa-mir-96	TSKU
LINC00323	hsa-mir-96	TSKU
LINC00242	hsa-mir-96	TSKU
LRRC3-AS1	hsa-mir-96	TSKU
UBE2Q1-AS1	hsa-mir-96	TSKU
AL161645.1	hsa-mir-96	TSKU
AC092171.1	hsa-mir-96	TSKU
GAS5	hsa-mir-96	TSKU
SYNPR-AS1	hsa-mir-96	TSKU
KLHL6-AS1	hsa-mir-96	TSKU
AC073352.1	hsa-mir-96	TSKU

GRM5-AS1	hsa-mir-96	TSKU
AL591845.1	hsa-mir-96	PRDM16
KIAA0087	hsa-mir-96	PRDM16
AC005280.1	hsa-mir-96	PRDM16
AC127496.1	hsa-mir-96	PRDM16
FAM87A	hsa-mir-96	PRDM16
LINC00336	hsa-mir-96	PRDM16
FAM201A	hsa-mir-96	PRDM16
UCA1	hsa-mir-96	PRDM16
AC096642.1	hsa-mir-96	PRDM16
LINC00466	hsa-mir-96	PRDM16
LINC00323	hsa-mir-96	PRDM16
LINC00242	hsa-mir-96	PRDM16
LRRC3-AS1	hsa-mir-96	PRDM16
UBE2Q1-AS1	hsa-mir-96	PRDM16
AL161645.1	hsa-mir-96	PRDM16
AC092171.1	hsa-mir-96	PRDM16
GAS5	hsa-mir-96	PRDM16
SYNPR-AS1	hsa-mir-96	PRDM16
KLHL6-AS1	hsa-mir-96	PRDM16
AC073352.1	hsa-mir-96	PRDM16
GRM5-AS1	hsa-mir-96	PRDM16
AL591845.1	hsa-mir-96	SCARB1
KIAA0087	hsa-mir-96	SCARB1
AC005280.1	hsa-mir-96	SCARB1
AC127496.1	hsa-mir-96	SCARB1
FAM87A	hsa-mir-96	SCARB1
LINC00336	hsa-mir-96	SCARB1
FAM201A	hsa-mir-96	SCARB1
UCA1	hsa-mir-96	SCARB1
AC096642.1	hsa-mir-96	SCARB1
LINC00466	hsa-mir-96	SCARB1
LINC00323	hsa-mir-96	SCARB1
LINC00242	hsa-mir-96	SCARB1
LRRC3-AS1	hsa-mir-96	SCARB1
UBE2Q1-AS1	hsa-mir-96	SCARB1
AL161645.1	hsa-mir-96	SCARB1
AC092171.1	hsa-mir-96	SCARB1
GAS5	hsa-mir-96	SCARB1
SYNPR-AS1	hsa-mir-96	SCARB1
KLHL6-AS1	hsa-mir-96	SCARB1
AC073352.1	hsa-mir-96	SCARB1
GRM5-AS1	hsa-mir-96	SCARB1
AL591845.1	hsa-mir-96	SLC25A25
KIAA0087	hsa-mir-96	SLC25A25
AC005280.1	hsa-mir-96	SLC25A25
AC127496.1	hsa-mir-96	SLC25A25

FAM87A	hsa-mir-96	SLC25A25
LINC00336	hsa-mir-96	SLC25A25
FAM201A	hsa-mir-96	SLC25A25
UCA1	hsa-mir-96	SLC25A25
AC096642.1	hsa-mir-96	SLC25A25
LINC00466	hsa-mir-96	SLC25A25
LINC00323	hsa-mir-96	SLC25A25
LINC00242	hsa-mir-96	SLC25A25
LRRC3-AS1	hsa-mir-96	SLC25A25
UBE2Q1-AS1	hsa-mir-96	SLC25A25
AL161645.1	hsa-mir-96	SLC25A25
AC092171.1	hsa-mir-96	SLC25A25
GAS5	hsa-mir-96	SLC25A25
SYNPR-AS1	hsa-mir-96	SLC25A25
KLHL6-AS1	hsa-mir-96	SLC25A25
AC073352.1	hsa-mir-96	SLC25A25
GRM5-AS1	hsa-mir-96	SLC25A25

Table S6. The connection degree of each gene in the ceRNA network.

Gene	Type	Connection degree
KIAA0087	lncRNA	8
CLLU1	lncRNA	8
AC010336.2	lncRNA	8
LINC00466	lncRNA	8
DLX6-AS1	lncRNA	8
SNHG1	lncRNA	8
MAGI2-AS3	lncRNA	7
GAS5	lncRNA	7
GRM5-AS1	lncRNA	7
FAM66C	lncRNA	6
LINC00242	lncRNA	6
AC110491.1	lncRNA	6
LINC00402	lncRNA	6
LINC00261	lncRNA	6
AC127496.1	lncRNA	5
FAM87A	lncRNA	5
FAM201A	lncRNA	5
BOLA3-AS1	lncRNA	5
LINC00443	lncRNA	5
PVT1	lncRNA	5
AL591845.1	lncRNA	4
C15orf56	lncRNA	4
UCA1	lncRNA	4
AL137145.2	lncRNA	4
SFTA1P	lncRNA	4
AL161645.1	lncRNA	4
LINC00494	lncRNA	4

HOTTIP	lncRNA	4
CRNDE	lncRNA	4
NRG1-IT1	lncRNA	4
C2orf48	lncRNA	3
C10orf91	lncRNA	3
LINC00313	lncRNA	3
AC087392.1	lncRNA	3
AC092117.1	lncRNA	3
AL137145.1	lncRNA	3
AL021068.1	lncRNA	3
AL356356.1	lncRNA	3
LINC00323	lncRNA	3
LINC00348	lncRNA	3
HOTAIR	lncRNA	3
LRRC3-AS1	lncRNA	3
UBE2Q1-AS1	lncRNA	3
AC092171.1	lncRNA	3
MLIP-AS1	lncRNA	3
AC015987.1	lncRNA	3
CLRN1-AS1	lncRNA	3
SYNPR-AS1	lncRNA	3
FAM66D	lncRNA	3
FBXL19-AS1	lncRNA	3
H19	lncRNA	2
AC005280.1	lncRNA	2
LINC00304	lncRNA	2
LINC00336	lncRNA	2
AL359878.1	lncRNA	2
AC108134.1	lncRNA	2
AC016773.1	lncRNA	2
THRB-IT1	lncRNA	2
LINC00337	lncRNA	2
LINC00460	lncRNA	2
AL137798.1	lncRNA	2
AC009121.1	lncRNA	2
ITIH4-AS1	lncRNA	2
AC080129.1	lncRNA	2
TM4SF1-AS1	lncRNA	2
KLHL6-AS1	lncRNA	2
AC073352.1	lncRNA	2
AL139147.1	lncRNA	2
AP006285.1	lncRNA	2
RERG-IT1	lncRNA	2
AC012074.1	lncRNA	1
LINC00315	lncRNA	1
C9orf163	lncRNA	1
AC116351.1	lncRNA	1

PSORS1C3	lncRNA	1
TTLL10-AS1	lncRNA	1
FAM99A	lncRNA	1
AP000525.1	lncRNA	1
AC011481.1	lncRNA	1
AL354984.1	lncRNA	1
AC090150.1	lncRNA	1
AC080037.1	lncRNA	1
AC096642.1	lncRNA	1
CLDN10-AS1	lncRNA	1
POU6F2-AS1	lncRNA	1
WARS2-IT1	lncRNA	1
LINC00184	lncRNA	1
DBH-AS1	lncRNA	1
SZT2-AS1	lncRNA	1
HM13-AS1	lncRNA	1
DLG3-AS1	lncRNA	1
F10-AS1	lncRNA	1
HS1BP3-IT1	lncRNA	1
SLC6A1-AS1	lncRNA	1
FAM215B	lncRNA	1
AC037487.1	lncRNA	1
TPRG1-AS1	lncRNA	1
SAPCD1-AS1	lncRNA	1
NEXN-AS1	lncRNA	1
LINC00316	lncRNA	1
AL391832.1	lncRNA	1
AP004609.1	lncRNA	1
AL118511.1	lncRNA	1
ARHGAP31-AS1	lncRNA	1
AL359541.1	lncRNA	1
GK-AS1	lncRNA	1
AL133367.1	lncRNA	1
AL445228.2	lncRNA	1
AP001781.1	lncRNA	1
AC147651.1	lncRNA	1
AC110619.1	lncRNA	1
AL139385.1	lncRNA	1
AC093734.1	lncRNA	1
AP001029.2	lncRNA	1
AL021707.2	lncRNA	1
DNM1P35	lncRNA	1
hsa-mir-211	miRNA	50
hsa-mir-182	miRNA	38
hsa-mir-122	miRNA	36
hsa-mir-141	miRNA	30
hsa-mir-200a	miRNA	30

hsa-mir-144	miRNA	27
hsa-mir-222	miRNA	27
hsa-mir-96	miRNA	26
hsa-mir-383	miRNA	26
hsa-mir-429	miRNA	26
hsa-mir-31	miRNA	26
hsa-mir-21	miRNA	24
hsa-mir-183	miRNA	17
KIAA1549	mRNA	2
SCD5	mRNA	2
NPTX1	mRNA	2
SLC35D1	mRNA	2
GALNT3	mRNA	2
HOXB5	mRNA	2
MACC1	mRNA	2
EPHA7	mRNA	2
FOXC1	mRNA	1
DIO1	mRNA	1
MTSS1	mRNA	1
FOXD4L1	mRNA	1
SLC43A1	mRNA	1
EDIL3	mRNA	1
SLC52A2	mRNA	1
SAMD5	mRNA	1
RARG	mRNA	1
ESR1	mRNA	1
FAM46A	mRNA	1
ELOVL7	mRNA	1
ELL2	mRNA	1
ELOVL6	mRNA	1
MITF	mRNA	1
HOXC8	mRNA	1
FZD6	mRNA	1
BDNF	mRNA	1
SCRN1	mRNA	1
TMTC2	mRNA	1
KIF5C	mRNA	1
SHCBP1	mRNA	1
NTF3	mRNA	1
SLC7A1	mRNA	1
ZCCHC24	mRNA	1
HOXC13	mRNA	1
IL11	mRNA	1
TMEM170B	mRNA	1
MFSD6	mRNA	1
TSKU	mRNA	1
PKM	mRNA	1

PIK3R1	mRNA	1
CPEB3	mRNA	1
ALDOA	mRNA	1
PTPN14	mRNA	1
FGF9	mRNA	1
CREB5	mRNA	1
FOS	mRNA	1
PRDM16	mRNA	1
UBASH3B	mRNA	1
TGFB2	mRNA	1
JAG1	mRNA	1
CCNB1	mRNA	1
FOXD4	mRNA	1
PMAIP1	mRNA	1
SCARB1	mRNA	1
FOXF2	mRNA	1
HOXA10	mRNA	1
SLC25A25	mRNA	1
PANK1	mRNA	1
EPM2A	mRNA	1
JPH1	mRNA	1

Table S7. Functional enrichment analysis of the sixty DEmRNAs.

Category	Gene function	Gene count	Genes	P-value
GOTERM_MF_FAT	Sequence-specific DNA binding	14	RARG, MITF, ESR1, CREB5, PRDM16, FOS, HOXC8, FOXD4L1, HOXC13, HOXB5, FOXF2, HOXA10, FOXC1, FOXD4	6.10562369768206E-8
GOTERM_BP_FAT	Sensory organ development	9	BDNF, RARG, HOXC13, FGF9, MITF, FOXC1, JAG1, FZD6, TGFB2	1.0623515278449677E-6
GOTERM_MF_FAT	Growth factor activity	7	BDNF, NTF3, FGF9, JAG1, MACC1, IL11, TGFB2	1.5837257321808756E-5
GOTERM_MF_FAT	Transcription factor activity	13	RARG, MITF, ESR1, CREB5, FOS, HOXC8, FOXD4L1, HOXC13, HOXB5, FOXF2, HOXA10, FOXC1, FOXD4	6.671669448676301E-5
GOTERM_BP_FAT	Positive regulation of macromolecule metabolic processes	12	CCNB1, FOS, RARG, NTF3, MITF, FOXF2, ESR1, CREB5, FOXC1, PRDM16, IL11, TGFB2	1.308873087185182E-4
GOTERM_BP_FAT	Positive regulation of gene expression	10	FOS, RARG, NTF3, MITF, FOXF2, ESR1, CREB5, FOXC1, PRDM16, IL11	1.4745875098500156E-4
GOTERM_BP_FAT	Positive regulation of transcription, DNA-dependent	9	FOS, RARG, NTF3, MITF, FOXF2, CREB5, FOXC1, PRDM16, IL11	2.1100888181966603E-4
GOTERM_BP_FAT	Positive regulation of RNA metabolic processes	9	FOS, RARG, NTF3, MITF, FOXF2, CREB5, FOXC1, PRDM16, IL11	2.2336005273247087E-4
GOTERM_BP_FAT	Regulation of neuronal apoptosis	5	EPHA7, BDNF, NTF3, ESR1, TGFB2	2.6258448452550056E-4

GOTERM_MF_FAT	Transcription activity regulator	15	RARG, MITF, ESR1, CREB5, PRDM16, ELL2, FOS, HOXC8, FOXD4L1, HOXC13, HOXB5, FOXF2, HOXA10, FOXC1, FOXD4	3.090513433354426E-4
GOTERM_BP_FAT	Positive regulation of macromolecule biosynthetic processes	10	FOS, RARG, NTF3, MITF, FOXF2, CREB5, FOXC1, PRDM16, IL11, TGFB2	3.565900962840298E-4
GOTERM_BP_FAT	Positive regulation of biosynthetic processes	10	FOS, RARG, NTF3, MITF, FOXF2, CREB5, FOXC1, PRDM16, IL11, TGFB2	5.559481834225973E-4
GOTERM_BP_FAT	Positive regulation of transcription	9	FOS, RARG, NTF3, MITF, FOXF2, CREB5, FOXC1, PRDM16, IL11	6.484613226112572E-4
GOTERM_BP_FAT	Regulation of transcription, DNA-dependent	16	RARG, NTF3, MITF, ESR1, CREB5, PRDM16, IL11, FOS, HOXC8, FOXD4L1, HOXC13, HOXB5, FOXF2, HOXA10, FOXC1, FOXD4	6.684090192923358E-4
GOTERM_BP_FAT	Regulation of RNA metabolic processes	16	RARG, NTF3, MITF, ESR1, CREB5, PRDM16, IL11, FOS, HOXC8, FOXD4L1, HOXC13, HOXB5, FOXF2, HOXA10, FOXC1, FOXD4	8.472023318856754E-4

Table S8. Pathway enrichment analysis of the sixty DEmRNAs.

ID	Description	Gene count	Gene	P-value
hsa01522	Endocrine resistance	4	JAG1/FOS/PIK3R1/ESR1	1.7E-05
hsa04915	Estrogen signaling pathway	4	FOS/PIK3R1/CREB5/ESR1	1.84E-05
hsa04668	TNF signaling pathway	4	JAG1/FOS/PIK3R1/CREB5	2.75E-05
hsa05200	Pathways in cancer	6	MITF/PIK3R1/TGFB2/FOS/FGF9/FZD6	3.27E-05
hsa04010	MAPK signaling pathway	5	NTF3/TGFB2/FGF9/BDNF/FOS	4.65E-05
hsa04380	Osteoclast differentiation	4	FOS/PIK3R1/MITF/TGFB2	5.47E-05
hsa05161	Hepatitis B	4	FOS/PIK3R1/CREB5/TGFB2	8.01E-05

Table S9. Twenty-six DElncRNAs associated with overall survival in cholangiocarcinoma.

Gene	Group	Expression level	Number of patients	Mean survival time	P-value
C2orf48	high	>23.9185162419751	9	1.319721679	0.000759798
	low	<=23.9185162419751	27	3.686147906	
LINC00337	high	>7.09559093952817	8	1.106164384	0.00114019
	low	<=7.09559093952817	28	3.593247428	
AL359878.1	high	>13.1461991619369	29	3.515814804	0.001628222
	low	<=13.1461991619369	7	0.997260274	
NEXN-AS1	high	>3.29364698838757	29	3.578851649	0.0028923
	low	<=3.29364698838757	7	1.383405088	
AL021068.1	high	>17.1330942097468	9	5.41369863	0.003940705
	low	<=17.1330942097468	27	2.502455603	
AL137145.1	high	>12.2716490155312	30	3.419460342	0.006141131
	low	<=12.2716490155312	6	0.996712329	
AC092117.1	high	>64.6059813416698	14	4.43569863	0.007079418
	low	<=64.6059813416698	22	2.071490838	
SLC6A1-AS1	high	>0.778775094738471	25	3.719268922	0.007123007
	low	<=0.778775094738471	11	1.892851806	
HOTAIR	high	>2.05736815519913	10	1.48630137	0.007353879
	low	<=2.05736815519913	26	3.607402548	

ARHGAP31-AS1	high	>20.7422445876976	7	5.363013699	0.00857414
	low	<=20.7422445876976	29	2.314640313	
SFTA1P	high	>1.03681837130166	24	3.892271539	0.008908292
	low	<=1.03681837130166	12	1.823561644	
AC015987.1	high	>23.4263766153645	5	1.145753425	0.012045186
	low	<=23.4263766153645	31	3.393241787	
AC147651.1	high	>57.4231114932046	27	3.533732795	0.013078154
	low	<=57.4231114932046	9	1.37564688	
SYNPR-AS1	high	>0.00705524554364256	26	3.695928727	0.013999695
	low	<=0.00705524554364256	10	2.016164384	
LINC00315	high	>1.93798374022249	33	3.340576672	0.015899241
	low	<=1.93798374022249	3	1.042922374	
AL356356.1	high	>8.44173869146235	33	3.326028031	0.016811514
	low	<=8.44173869146235	3	1.141552511	
DBH-AS1	high	>229.817972061259	4	0.935616438	0.025543002
	low	<=229.817972061259	32	3.331929482	
FAM87A	high	>0.541359588507206	31	3.447344893	0.027561558
	low	<=0.541359588507206	5	1.664657534	
CLRN1-AS1	high	>4.15035091132941	4	5.41369863	0.033069536
	low	<=4.15035091132941	32	2.78102961	
AC005280.1	high	>4.72137589949119	10	2.017424658	0.037898096
	low	<=4.72137589949119	26	3.563174362	
CLLU1	high	>7.83843904390199	6	3.969863014	0.040393846
	low	<=7.83843904390199	30	2.756645396	
AP001029.2	high	>8.68798723540598	25	3.618431617	0.041730852
	low	<=8.68798723540598	11	1.892794876	
CRNDE	high	>131.15146645312	19	2.47121415	0.046034955
	low	<=131.15146645312	17	3.383958904	
AP001781.1	high	>0.761590767502108	31	2.776581018	0.047604434
	low	<=0.761590767502108	5	4.421917808	
AL445228.2	high	>9.78008009238626	16	2.406946558	0.048286692
	low	<=9.78008009238626	20	3.780072591	
LINC00494	high	>550.523670211504	13	3.492952816	0.048944575
	low	<=550.523670211504	23	2.5164266	

Table S10. Three DEmiRNAs associated with overall survival in cholangiocarcinoma.

Gene	Group	Expression level	Number of patients	Mean survival time	P-value
hsa-mir-383	high	>0.013101631352411	29	3.49985	0.021865
	low	<=0.013101631352411	7	1.542466	
hsa-mir-144	high	>52.6472519026576	31	3.380841	0.023828
	low	<=52.6472519026576	5	1.281096	
hsa-mir-141	high	>37.3214082654265	26	2.615831	0.038067
	low	<=37.3214082654265	10	4.272534	

Table S11. Thirteen DEmRNAs associated with overall survival in cholangiocarcinoma.

Gene	Group	Expression level	Number of patients	Mean survival time	P-value
HOXC13	high	>48.1503713897389	4	0.576255708	6.99816E-05
	low	<=48.1503713897389	32	3.36263421	
FAM46A	high	>1088.71219092439	5	0.591232877	9.56482E-05
	low	<=1088.71219092439	31	3.462194503	
HOXA10	high	>0.688276286730929	29	3.501482176	0.00241661
	low	<=0.688276286730929	7	0.9543379	
SCRN1	high	>3506.98153181131	17	4.053332192	0.003445418
	low	<=3506.98153181131	19	1.911660506	
EDIL3	high	>1331.4211476488	4	0.838356164	0.012489182
	low	<=1331.4211476488	32	3.330720313	
SLC25A25	high	>1295.51078573059	8	1.696986301	0.015308753
	low	<=1295.51078573059	28	3.603969161	
JPH1	high	>8.55485029985959	30	3.468397514	0.015666656
	low	<=8.55485029985959	6	1.277625571	
PIK3R1	high	>1983.99420182331	13	4.556164384	0.020283734
	low	<=1983.99420182331	23	2.528714558	
TMTC2	high	>630.350776292282	7	4.421917808	0.028364539
	low	<=630.350776292282	29	2.712478378	
KIF5C	high	>15.6811663838079	33	3.26574812	0.030429196
	low	<=15.6811663838079	3	0.654794521	
TSKU	high	>1849.82408356	9	1.682597666	0.030674744
	low	<=1849.82408356	27	3.605141906	
FOXC1	high	>73.8802257655522	32	3.401221723	0.034297632
	low	<=73.8802257655522	4	1.442465753	
TGFB2	high	>1151.28842822554	19	2.133416153	0.039994177
	low	<=1151.28842822554	17	3.855322631	

Table S12. Linear regression analysis between the DElncRNAs and DEmRNAs targeted by hsa-mir-211.

DElncRNA	DEmiRNA	DEmRNA	Pearson correlation coefficient	P-value
LINC00261	hsa-mir-211	SLC43A1	0.652982841	1.58E-05
HOTAIR	hsa-mir-211	HOXC8	0.567688223	0.000304998
LINC00242	hsa-mir-211	ELOVL6	0.557404976	0.000413143
SAPCD1-AS1	hsa-mir-211	SLC43A1	0.520220801	0.00114501
HOTTIP	hsa-mir-211	HOXC8	0.496807315	0.002054833
AL137145.1	hsa-mir-211	SLC43A1	0.482413155	0.00288583
AP001781.1	hsa-mir-211	ELOVL6	0.477889125	0.003201379
HOTTIP	hsa-mir-211	SAMD5	0.471874751	0.003667087
HOTAIR	hsa-mir-211	FOXC1	0.469358319	0.00387874
MAGI2-AS3	hsa-mir-211	ZCCHC24	0.458183431	0.004951668
SFTA1P	hsa-mir-211	ZCCHC24	0.438255757	0.007506917
AL021068.1	hsa-mir-211	TMTC2	0.426343256	0.009518485
BOLA3-AS1	hsa-mir-211	IL11	0.422310504	0.010296024
LINC00261	hsa-mir-211	CREB5	0.419398628	0.010890437
AL356356.1	hsa-mir-211	SLC43A1	0.413710363	0.01213588

MAGI2-AS3	hsa-mir-211	TMTC2	0.405569172	0.014126605
MAGI2-AS3	hsa-mir-211	ELOVL6	0.404356263	0.014445512
MAGI2-AS3	hsa-mir-211	NPTX1	0.401434518	0.015238686

Table S13. Gene enrichment in the high SLC43A1 expression group for patients with cholangiocarcinoma.

Name	Size	ES	NES	NOM p-value	FDR q-value	FWER p-value	Rank at max	Leading edge
KEGG_PARKINSONS_DISEASE	97	0.745182	3.530183	0	0	0	1619	tags=63%, list=9%, signal=69%
KEGG_OXIDATIVE_PHOSPHORYLATION	98	0.731244	3.446973	0	0	0	2002	tags=66%, list=11%, signal=74%
KEGG_HUNTINGTONS_DISEASE	154	0.596038	3.01596	0	0	0	1688	tags=46%, list=10%, signal=51%
KEGG_ALZHEIMERS_DISEASE	142	0.60436	3.000241	0	0	0	1619	tags=44%, list=9%, signal=48%
KEGG_PROTEASOME	42	0.608047	2.423091	0	0	0	4210	tags=67%, list=24%, signal=87%
KEGG_SYSTEMIC_LUPUS_ERYTHEMATOSUS	112	0.4741	2.275453	0	0.000153	0.001	2804	tags=39%, list=16%, signal=46%
KEGG_CITRATE_CYCLE_TCA_CYCLE	29	0.608744	2.215968	0	0.000254	0.002	4456	tags=66%, list=25%, signal=87%
KEGG_PEROXISOME	78	0.465569	2.101058	0	0.000851	0.008	4204	tags=49%, list=24%, signal=64%
KEGG_CARDIAC_MUSCLE_CONTRACTION	70	0.461038	2.045029	0	0.001236	0.013	2365	tags=39%, list=13%, signal=44%
KEGG_PORPHYRIN_AND_CHLOROPHYLL_METABOLISM	40	0.522917	2.031088	0	0.0012	0.014	5434	tags=58%, list=31%,

								signal=83 %
KEGG_FATTY_ACID_METABOLISM	42	0.504066	2.013674	0	0.001243	0.016	6314	tags=69%, list=36%, signal=107 %
KEGG_VALINE_LEUCINE_AND_ISOLEUCINE_DEGRADATION	44	0.469853	1.90813	0	0.003763	0.051	4677	tags=52%, list=26%, signal=71 %
KEGG_PROPANOATE_METABOLISM	32	0.508421	1.878955	0	0.005093	0.074	4482	tags=50%, list=25%, signal=67 %
KEGG_PROTEIN_EXPORT	22	0.546787	1.878577	0.006036	0.004788	0.075	4309	tags=59%, list=24%, signal=78 %
KEGG_PROXIMAL_TUBULE_BICARBONATE_RECLAMATION	22	0.566718	1.868255	0.003945	0.004821	0.081	3587	tags=64%, list=20%, signal=80 %
KEGG_ASCORBATE_AND_ALDARATE_METABOLISM	25	0.529109	1.854806	0.001931	0.005254	0.094	5157	tags=60%, list=29%, signal=85 %
KEGG_GLUTATHIONE_METABOLISM	43	0.455868	1.812007	0	0.007439	0.138	2451	tags=30%, list=14%, signal=35 %
KEGG_STEROID_HORMONE_BIOSYNTHESIS	53	0.43136	1.810883	0.001984	0.007025	0.138	5157	tags=55%, list=29%, signal=77 %
KEGG_GLYCINE_SERINE_AND_THREONINE_METABOLISM	31	0.495387	1.809049	0.004016	0.006834	0.142	3653	tags=42%, list=21%, signal=53 %
KEGG_BUTANOATE_METABOLISM	33	0.479206	1.750083	0.002024	0.012037	0.247	4482	tags=52%, list=25%, signal=69 %
KEGG_ALANINE_ASPARTATE_AND_GLUTAMATE_METABOLISM	32	0.466045	1.741268	0.00396	0.012386	0.264	4278	tags=53%, list=24%, signal=70 %
KEGG_PYRUVATE_METABOLISM	38	0.453011	1.740204	0.004032	0.011941	0.267	4674	tags=50%, list=26%,

								signal=68 %
KEGG_PRIMARY_BILE_ACID_BIOSYNTHESIS	16	0.548729	1.732242	0.011788	0.012157	0.28	4803	tags=56%, list=27%, signal=77 %
KEGG_ARGININE_AND_PROLINE_METABOLISM	51	0.423602	1.729514	0.001961	0.011907	0.285	4407	tags=47%, list=25%, signal=62 %
KEGG_SPLICEOSOME	123	0.34271	1.68055	0.001949	0.018244	0.417	5782	tags=46%, list=33%, signal=68 %
KEGG_BIOSYNTHESIS_OF_UNSATURATED_FATTY_ACIDS	20	0.512913	1.677651	0.020408	0.018045	0.427	4750	tags=50%, list=27%, signal=68 %
KEGG_METABOLISM_OF_XENOBIOTICS_BY_CYTOCHROME_P450	66	0.365879	1.617282	0.01	0.028976	0.614	5489	tags=50%, list=31%, signal=72 %
KEGG_DRUG_METABOLISM_OTHER_ENZYMES	51	0.37665	1.570707	0.006135	0.040811	0.75	5584	tags=51%, list=32%, signal=74 %
KEGG_RETINOL_METABOLISM	63	0.359605	1.537154	0.017308	0.051227	0.836	5489	tags=49%, list=31%, signal=71 %
KEGG_AMINOACYL_TRNA_BIOSYNTHESIS	41	0.388675	1.523393	0.02549	0.054648	0.874	2915	tags=29%, list=16%, signal=35 %
KEGG_GLYCOSYLPHOSPHATIDYLINOSITOL_GPI_ANCHOR_BIOSYNTHESIS	24	0.438903	1.522057	0.03006	0.053356	0.878	2900	tags=38%, list=16%, signal=45 %
KEGG_PYRIMIDINE_METABOLISM	95	0.323198	1.512775	0.011696	0.055803	0.893	3224	tags=29%, list=18%, signal=36 %
KEGG_N_GLYCAN_BIOSYNTHESIS	46	0.369218	1.490087	0.036145	0.062187	0.932	3302	tags=35%, list=19%, signal=43 %
KEGG_DRUG_METABOLISM_CYTOCHROME_P450	68	0.336395	1.461512	0.02444	0.073307	0.954	5489	tags=49%, list=31%,

								signal=70 %
KEGG_TRYPTOPHAN_METABOLISM	38	0.372202	1.44707	0.033138	0.079649	0.965	1781	tags=21%, list=10%, signal=23 %

Table S14. Gene enrichment in the high HOXC8 expression group for patients with cholangiocarcinoma.

Name	Size	ES	NES	NOM p-value	FDR q-value	FWER p-value	Rank at max	Leading edge
KEGG_PRIMARY_IMMUNODEFICIENCY	34	0.582759	2.136621	0	0.002018	0.003	5058	tags=68 %, list=29%, signal=95%
KEGG_TASTE_TRANSDUCTION	34	0.515663	1.863118	0.001733	0.028951	0.079	4867	tags=59 %, list=28%, signal=81%
KEGG_HEMATOPOIETIC_CELL_LINEAGE	81	0.422977	1.843209	0	0.024164	0.096	5777	tags=56 %, list=33%, signal=82%
KEGG_INTESTINAL_IMMUNE_NETWORK_FOR_IGA_PRODUCTION	45	0.434887	1.686596	0.005226	0.094873	0.424	5344	tags=58 %, list=30%, signal=83%
KEGG_BASAL_CELL_CARCINOMA	54	0.405306	1.668061	0.013201	0.090246	0.481	4262	tags=46 %, list=24%, signal=61%
KEGG_CYTOKINE_CYTOKINE_RECEPTOR_INTERACTION	232	0.314857	1.630173	0	0.102692	0.598	4564	tags=38 %, list=26%, signal=51%
KEGG_ASTHMA	26	0.469814	1.5902	0.013793	0.125003	0.721	5080	tags=50 %, list=29%, signal=70%
KEGG_HEDGEHOG_SIGNALING_PATHWAY	53	0.387806	1.585679	0.014876	0.112885	0.733	5245	tags=55 %,

								list=30%, signal=78%
KEGG_NOTCH_SIGNALING_PATHWAY	47	0.392014	1.554372	0.02418	0.132222	0.817	6445	tags=53%, list=36%, signal=83%
KEGG_CELL_ADHESION_MOLECULES_CAMS	126	0.324596	1.547687	0.007813	0.125723	0.834	5444	tags=47%, list=31%, signal=67%
KEGG_NEUROACTIVE_LIGAND_RECEPTOR_INTERACTION	232	0.296876	1.532032	0.001484	0.129439	0.867	3517	tags=30%, list=20%, signal=37%
KEGG_ARRHYTHMOGENIC_RIGHT_VENTRICULAR_CARDIOMYOPATHY_ARVC	73	0.338712	1.46767	0.025559	0.17971	0.957	4651	tags=40%, list=26%, signal=54%
KEGG_DILATED_CARDIOMYOPATHY	88	0.317172	1.403275	0.038397	0.26484	0.995	5451	tags=45%, list=31%, signal=65%
KEGG_VASCULAR_SMOOTH_MUSCLE_CONTRACTION	111	0.29953	1.389262	0.035427	0.273294	0.998	3575	tags=33%, list=20%, signal=42%
KEGG_CHEMOKINE_SIGNALING_PATHWAY	181	0.273851	1.380198	0.014837	0.27309	1	5690	tags=44%, list=32%, signal=64%
KEGG_SYSTEMIC_LUPUS_ERYTHEMATOSUS	112	0.293821	1.372502	0.04918	0.272304	1	4793	tags=38%, list=27%, signal=52%
KEGG_HYPERTROPHIC_CARDIOMYOPATHY_HCM	82	0.308653	1.361046	0.044944	0.262575	1	5182	tags=41%, list=29%, signal=58%

KEGG_NATURAL_KILLER_CELL_MEDIATED_CYTOTOXICITY	115	0.285154	1.329403	0.040453	0.290701	1	4124	tags=32%, list=23%, signal=42%
KEGG_CALCIUM_SIGNALING_PATHWAY	167	0.264522	1.314872	0.031484	0.290082	1	3059	tags=27%, list=17%, signal=32%

Table S15. Gene enrichment in the high ELOVL6 expression group for patients with cholangiocarcinoma.

Name	Size	ES	NES	NOM p-value	FDR q-value	FWER p-value	Rank at max	Leading edge
KEGG_COMPLEMENT_AND_COAGULATION_CASCADES	69	0.58806	2.740239	0	0	0	4209	tags=58%, list=24%, signal=76%
KEGG_PPAR_SIGNALING_PATHWAY	66	0.557117	2.493709	0	0	0	2759	tags=53%, list=16%, signal=63%
KEGG_ECM_RECEPTOR_INTERACTION	82	0.520671	2.481318	0	0	0	4883	tags=59%, list=28%, signal=80%
KEGG_GLYCINE_SERINE_AND_THREONINE_METABOLISM	31	0.599362	2.257494	0	0.000369	0.001	2979	tags=42%, list=17%, signal=50%
KEGG_RETINOL_METABOLISM	63	0.489359	2.205783	0	0.000296	0.001	4272	tags=48%, list=24%, signal=63%
KEGG_PRIMARY_BILE_ACID_BIOSYNTHESIS	16	0.676062	2.135889	0.002105	0.000721	0.003	2177	tags=69%, list=12%, signal=78%
KEGG_FATTY_ACID_METABOLISM	42	0.512704	2.129147	0	0.000618	0.003	3566	tags=43%,

								list=20%, signal=54%
KEGG_MATURITY_ONSET_DIABETES_OF_THE_YOUNG	25	0.587399	2.063265	0	0.001686	0.009	4299	tags=56%, list=24%, signal=74%
KEGG_DRUG_METABOLISM_CYTOCHROME_P450	68	0.445317	2.02261	0	0.002495	0.015	4272	tags=50%, list=24%, signal=66%
KEGG_ABC_TRANSPORTERS	44	0.498683	2.017387	0	0.002246	0.015	3225	tags=41%, list=18%, signal=50%
KEGG_VALINE_LEUCINE_AND_ISOLEUCINE_DEGRADATION	44	0.490323	1.993949	0.002387	0.002693	0.02	3242	tags=41%, list=18%, signal=50%
KEGG_BETA_ALANINE_METABOLISM	22	0.563249	1.933112	0	0.00451	0.037	2422	tags=45%, list=14%, signal=53%
KEGG_LYSINE_DEGRADATION	39	0.469142	1.862746	0.002439	0.010252	0.09	3558	tags=46%, list=20%, signal=58%
KEGG_PENTOSE_AND_GLUCURONATE_INTERCONVERSIONS	27	0.483038	1.791285	0.002364	0.01822	0.16	1273	tags=30%, list=7%, signal=32%
KEGG_STARCH_AND_SUCROSE_METABOLISM	48	0.41716	1.790372	0	0.017198	0.162	2883	tags=35%, list=16%, signal=42%
KEGG_FOCAL_ADHESION	197	0.324637	1.780355	0	0.01787	0.182	3630	tags=37%, list=21%, signal=46%

KEGG_ARRHYTHMOGENIC_RIGHT_VENTRICULAR_CARDIOMYOPATHY_ARVC	73	0.387852	1.778477	0	0.016971	0.184	5444	tags=53%, list=31%, signal=77%
KEGG_DRUG_METABOLISM_OTHER_ENZYMES	51	0.412529	1.773664	0	0.016948	0.194	2176	tags=35%, list=12%, signal=40%
KEGG_PEROXISOME	78	0.3624	1.700577	0	0.030142	0.343	3787	tags=37%, list=21%, signal=47%
KEGG_PRION_DISEASES	34	0.441455	1.695242	0.005013	0.0299	0.358	3494	tags=41%, list=20%, signal=51%
KEGG_NITROGEN_METABOLISM	22	0.483635	1.679559	0.007576	0.031711	0.391	4450	tags=50%, list=25%, signal=67%
KEGG_LINOLEIC_ACID_METABOLISM	28	0.444488	1.672835	0.018223	0.03214	0.41	3760	tags=50%, list=21%, signal=63%
KEGG_ARGININE_AND_PROLINE_METABOLISM	51	0.383247	1.653859	0.007813	0.036304	0.464	3787	tags=37%, list=21%, signal=47%
KEGG_BUTANOATE_METABOLISM	33	0.417207	1.610563	0.018265	0.04759	0.574	3489	tags=39%, list=20%, signal=49%
KEGG_TGF_BETA_SIGNALING_PATHWAY	85	0.332454	1.590555	0.002849	0.053886	0.633	5602	tags=48%, list=32%, signal=70%
KEGG_ALANINE_ASPARTATE_AND_GLUTAMATE_METABOLISM	32	0.418188	1.576543	0.006977	0.057575	0.666	2060	tags=28%, list=12%,

								signal=32%
KEGG_STEROID_HORMONE_BIOSYNTHESIS	53	0.360886	1.561224	0.023377	0.06128	0.704	3023	tags=34%, list=17%, signal=41%
KEGG_GLYCOLYSIS_GLUCONEOGENESIS	58	0.346823	1.557295	0.019022	0.06115	0.722	4435	tags=40%, list=25%, signal=53%
KEGG_METABOLISM_OF_XENOBIOTICS_BY_CYTOCHROME_P450	66	0.334517	1.511922	0.02551	0.082295	0.819	4272	tags=41%, list=24%, signal=54%
KEGG_HYPERTROPHIC_CARDIOMYOPATHY_HCM	82	0.310373	1.492306	0.01039	0.091784	0.856	5077	tags=44%, list=29%, signal=61%
KEGG_AMINO_SUGAR_AND_NUCLEOTIDE_SUGAR_METABOLISM	44	0.358431	1.47749	0.033573	0.098685	0.884	4113	tags=41%, list=23%, signal=53%
KEGG_SMALL_CELL_LUNG_CANCER	84	0.303803	1.451052	0.01269	0.110551	0.927	2440	tags=26%, list=14%, signal=30%
KEGG_RENAL_CELL_CARCINOMA	66	0.313127	1.421423	0.033679	0.130448	0.963	3367	tags=36%, list=19%, signal=45%
KEGG_DILATED_CARDIOMYOPATHY	88	0.27956	1.347271	0.034667	0.187101	0.992	5077	tags=42%, list=29%, signal=59%
KEGG_PROSTATE_CANCER	89	0.275545	1.334022	0.041885	0.188399	0.996	3784	tags=36%, list=21%, signal=46%

KEGG_REGULATION_OF_ACTIN_CYTOSKELETON	207	0.23301	1.28435	0.032934	0.248016	0.998	4165	tags=31%, list=24%, signal=41%
KEGG_PATHWAYS_IN_CANCER	318	0.211607	1.232495	0.023256	0.305505	1	4170	tags=31%, list=24%, signal=39%
